# A ‘click’ chemistry approach to novel entinostat (MS-275) based class I histone deacetylase proteolysis targeting chimeras†

**DOI:** 10.1039/d2md00199c

**Published:** 2022-11-01

**Authors:** Jasmine M. Cross, Megan E. Coulson, Joshua P. Smalley, Wiktoria A. Pytel, Ozair Ismail, Justin S. Trory, Shaun M. Cowley, James T. Hodgkinson

**Affiliations:** Leicester Institute of Structural and Chemical Biology, School of Chemistry, University of Leicester Leicester LE1 7RH UK JTHodgkinson@le.ac.uk; Department of Molecular and Cell Biology, University of Leicester Leicester LE1 7RH UK smc57@leicester.ac.uk

## Abstract

Click chemistry was utilised to prepare a library of PROTACs based on entinostat a class I histone deacetylase (HDAC) inhibitor in clinical trials. A novel PROTAC JMC-137 was identified as a HDAC1/2 and HDAC3 degrader in HCT116 cells. However, potency was compromised compared to previously identified class I HDAC PROTACs highlighting the importance in the choice of HDAC ligand, functional group for linker attachment and positioning in PROTAC design.

## Abstract 2

Click chemistry was utilised to prepare a library of PROTACs based on entinostat a class I histone deacetylase (HDAC) inhibitor in clinical trials.

## Graphical abstract

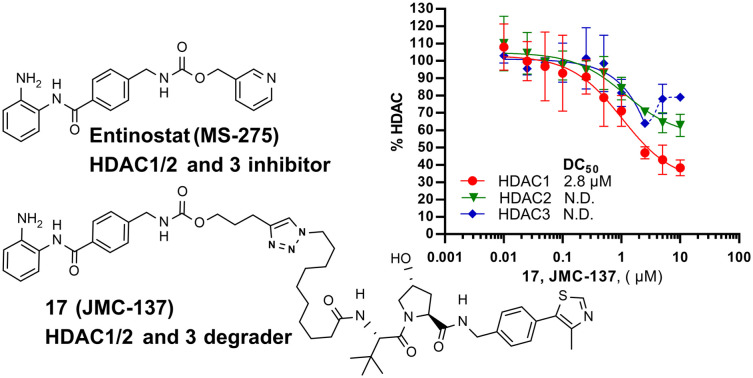

Histone deacetylase (HDAC) enzymes catalyse the hydrolysis of acetyl groups in *N*-ε-acetyl-l-lysine residues in histone proteins and non-histone proteins.^1,2^ There are eighteen HDAC isoforms present in humans, eleven of which are zinc dependent and seven nicotinamide adenine dinucleotide (NAD^+^) dependent.^1,2^ The zinc dependent class I HDACs 1, 2 & 3 exist as catalytic subunits in large multiprotein complexes localised in the nucleus catalysing the removal of acetyl groups in histone proteins.^3^ These HDACs and their associated complexes play an important role in chromatin structure and gene transcription.^4^

The HDAC inhibitors vorinostat, belinostat, panobinostat and romidepsin have been approved for use in the clinic, most commonly used in the treatment of haematological cancers. However, these drugs exhibit poor selectivity between the eleven zinc dependent HDAC enzymes and can be associated with debilitating side effects.^3,5^ Studies have demonstrated that the selective targeting of HDAC1/2 or HDAC3 may result in enhanced therapeutic benefits with reduced side effects in certain cancers.^6–8^

Entinostat (MS-275) is a selective class I HDAC1/2 & 3 inhibitor that has been in clinical trials for treating solid tumours and haematological cancers (Fig. 1).^9^ More recently, in further clinical trial studies, entinostat has demonstrated promise as a combination therapy with immunotherapy treatments to treat advanced tumours.^10^ Chidamide, structurally similar to entinostat, has also been approved by the China FDA for the treatment of peripheral T-cell lymphoma.^11^

**Fig. 1:**
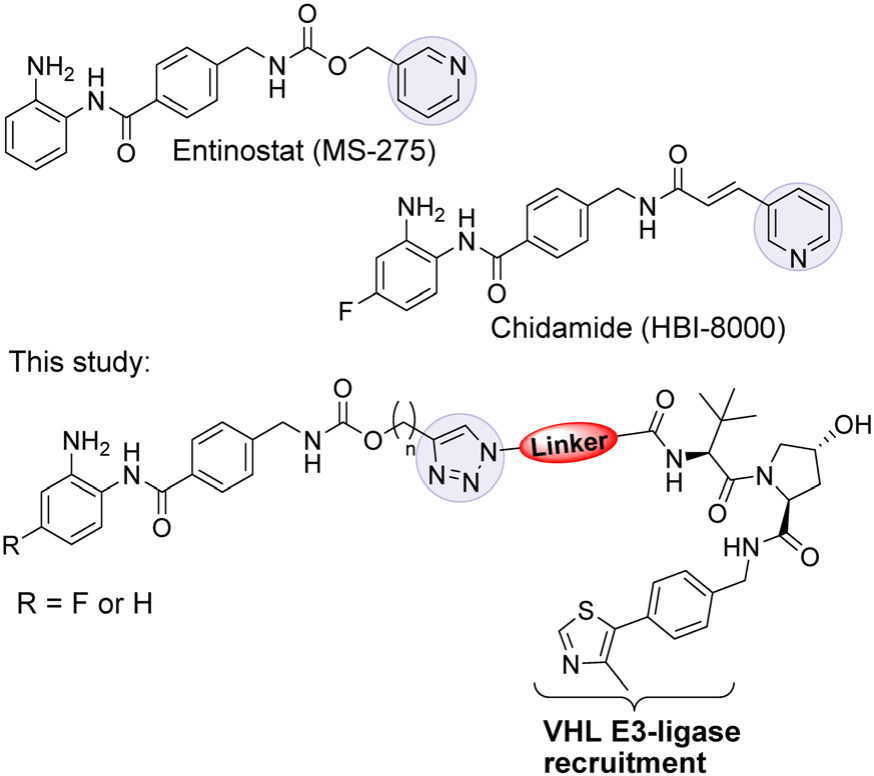
Entinostat (MS-275) is a class I HDAC 1, 2 & 3 inhibitor in clinical trials. Chidamide has been approved for the treatment of peripheral T-cell lymphoma in China. In this study we investigated class I HDAC PROTACs based on entinostat.

We and others have reported P̲r̲o̲teolysis T̲a̲rgeting C̲himeras (PROTACs) capable of degrading class I HDACs 1, 2 & 3.^12–15^ PROTACs utilise the cell's ubiquitination machinery to degrade the desired protein of interest *via* the proteasome.^16–18^ PROTACs contain a ligand for the protein of interest, a linker, and an E3 ligase recruiting ligand.^19^ We were inspired to synthesise a small library of PROTACs whereby the HDAC binding component closely resembles the clinical candidate entinostat. In previous studies utilising the class I HDAC inhibitor CI-994 in PROTAC design we discovered linker lengths of approximately 12 atoms were necessary for degradation.^12,15^ In this study, we envisaged that the incorporation of the carbamate group and heterocycle present in entinostat may facilitate shorter linker lengths, perhaps making such PROTACs more amenable towards drug discovery efforts. We were further motivated by the successful progression of PROTACs ARV-110 and ARV-471 in clinical trials for metastatic castration resistant prostate cancer and metastatic ER positive/HER2 negative breast cancer respectively.^20,21^

In our PROTAC design we hypothesised that the pyridine of entinostat which is not directly involved in binding in the HDAC catalytic active site but solvent exposed could be substituted with a triazole motif.^22,23^ This would facilitate the rapid access to a library of entinostat based PROTACs utilising copper(i)-catalysed azide/alkyne cycloaddition (CuAAC) chemistry, referred as ‘click chemistry’ (Scheme 1).

**Scheme 1:**
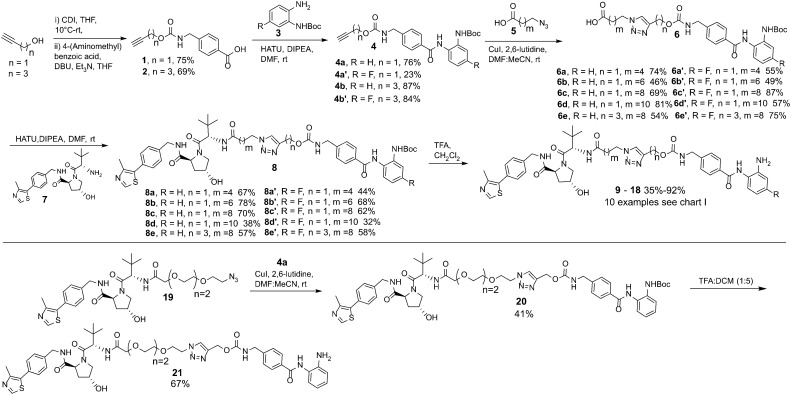
Synthetic route to entinostat based PROTACs using copper(i)-catalysed azide/alkyne cycloaddition (CuAAC) chemistry.

The VHL Von Hippel–Lindau (VHL) ligand was chosen as the E3-ligase ligand as we and others have previously found it to be one of the more effective E3-ligands in degrading class I HDACs.^12–15^ We also wanted to prepare entinostat analogues incorporating a fluorine atom on the benzamide ring, structurally similar to chidamide and also reported to infer selectivity for HDAC3.^24^

In our synthetic route, analogue 21 aside, we decided to functionalise the entinostat HDAC ligand with linkers of various lengths rather that functionalising the VHL ligand with linkers (Scheme 1). Although VHL analogues functionalised with linkers are now commercially available they are typically expensive for tens of milligrams quantities, while the starting materials for the entinostat functionalised linkers of 6 are inexpensive and can be prepared in fewer steps.

Propargyl alcohol and 4-pentyn-1-ol were reacted in parallel with carbonyldiimidazole (CDI) to generate imidazole containing carbamates *in situ*. 4-(Aminomethyl) benzoic acid was then added to this reaction to yield 1 & 2 containing the carbamate group present in entinostat and an alkyne group for click chemistry in latter synthetic steps. 1 & 2 were then reacted with Boc-protected, fluorine or non-fluorine substituted, *o*-phenylenediamines 3*via* HATU promoted amide coupling to generate Boc-protected entinostat analogues of substructure 4.

The copper(i)-catalysed azide/alkyne cycloaddition reaction was carried out with analogues of 4 and azide functionalised carboxylic acids of substructure 5, with the azides prepared or purchased. Amide coupling *via* HATU with commercially available VHL ligand, 7, and linker functionalised entinostat analogues 6 gave Boc-protected analogues of substructure 8. The Boc protecting group was removed by stirring in DCM/TFA, with any remaining residual TFA was removed using a carbonate based solid phase resin to yield ten examples of entinostat based heterobifunctional molecules 9–18 (Chart 1).

**Chart 1:**
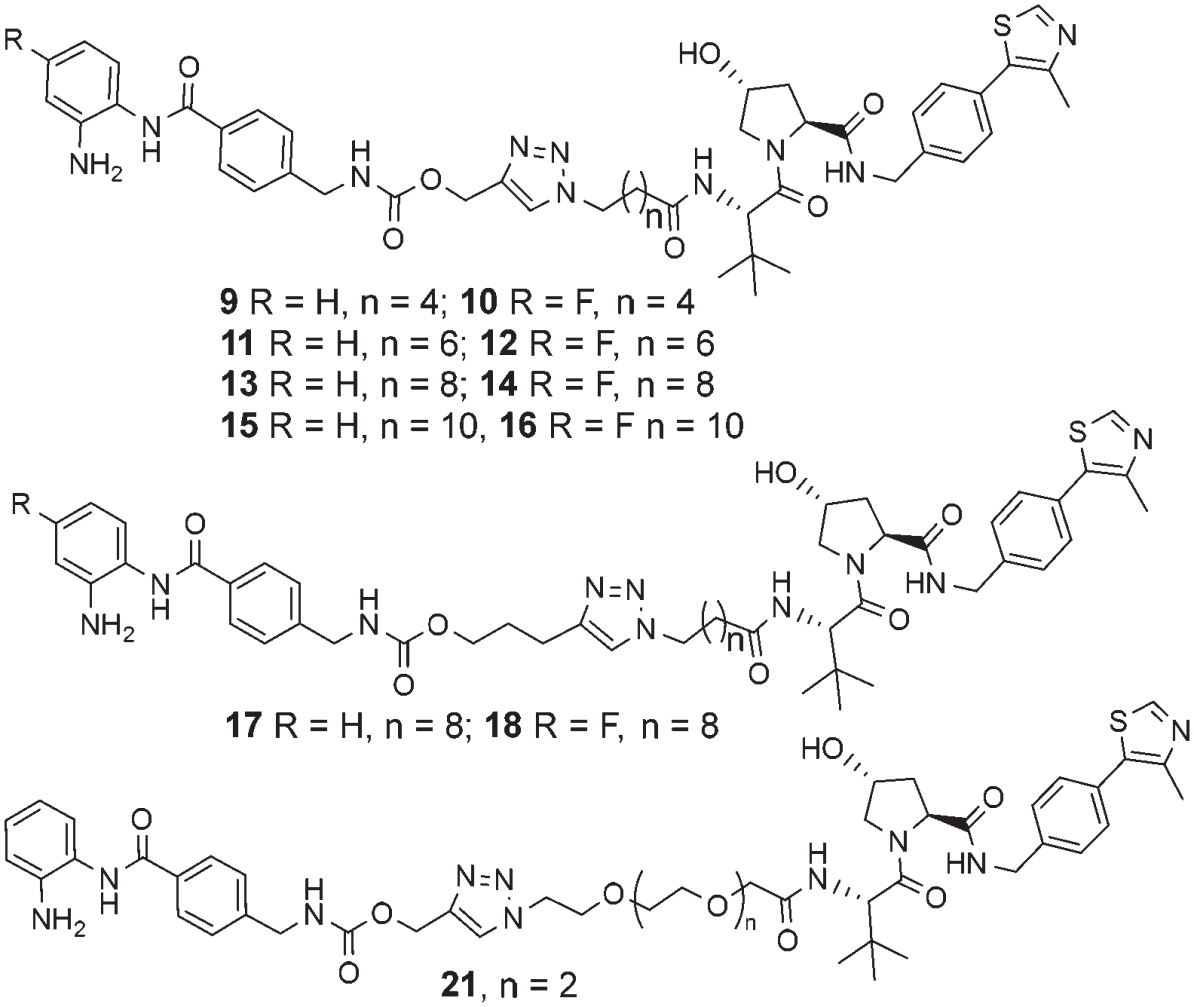
Library of entinostat based heterobifunctional molecules of varying linker lengths for biological evaluation.

We also prepared a polyethylene glycol (PEG) linker analogue 21, commonly incorporated in PROTACs. For this example we decided to purchase VHL functionalised with a PEG linker, 19, to determine if this could undergo click chemistry directly with 4a. The click chemistry to synthesise 20 proceeded in moderate yield, this was again followed by Boc removal to generate the PEG linker analogue 21. This demonstrates the feasibility of two alternative synthetic routes to synthesise these heterobifunctional molecules. The latter may be the preferred option if VHL functionalised linkers with azides are readily at hand.

We next proceeded to the biological evaluation of our library, with the aim of the first screen to identify active degrader molecules. To achieve this each compound was added to a HCT116 cell line at concentrations of 10 μM, 1 μM and 0.1 μM for 24 hours and the relative abundance of HDAC1/2 & HDAC3 was determined by quantitative fluorescent western blotting with specific antibodies for HDAC1, HDAC2, and HDAC3 (for blots see ESI†). The 24 hour time point was chosen as we previously observed that *D*_max_ values with analogous benzamide VHL PROTACs generally plateau at 24 hours, with only very marginal increases in *D*_max_ observed after 24 hours.^15^ This was performed side-by-side with our previously identified class I HDAC degrader at 10 μM referred to as JPS004 as a positive control, and the analogous benzamide class I HDAC inhibitor CI-994 at 10 μM. JPS004 exhibits HDAC1/2 degradation in a dose dependent manner reaching maximum HDAC1/2 degradation at 10 μM, while we recently discovered such analogues, although effective HDAC3 degraders exhibit a hook effect whereby HDAC3 degradation is compromised at concentrations greater than 1 μM and little HDAC3 degradation observed at 10 μM.^15^

Throughout the library the trend in HDAC1 degradation matched that of HDAC2, except overall the degradation of HDAC1 was more prominent than HDAC2 (Fig. 2). There was a clear dependence on linker length with 13 (10 atom linker) exhibiting 52% HDAC1 degradation and 40% HDAC2 degradation at 10 μM, while compounds 15 and 17 (both 12 atoms in linker length, 17 containing an extra two methylene groups before the triazole) exhibited the most prominent HDAC1/2 degradation of this series of compounds. This is consistent with what we have previously observed in other studies,^12,15^ however we were hoping incorporation of the traizole and carbamate moiety in these PROTACs would reduce the length of the alkyl linker required for degradation, based on these results this was not the case. Regarding HDAC3 degradation intriguingly the analogue 11 exhibited 70% HDAC3 degradation at 10 μM, however this was not maintained at 1 μM. Compounds 15 and 16 exhibited greater than 50% HDAC3 degradation, while 17, containing the triazole group positioned further away from the HDAC inhibitor seemed to exhibit preferential HDAC1/2 degradation over HDAC3 degradation.

**Fig. 2:**
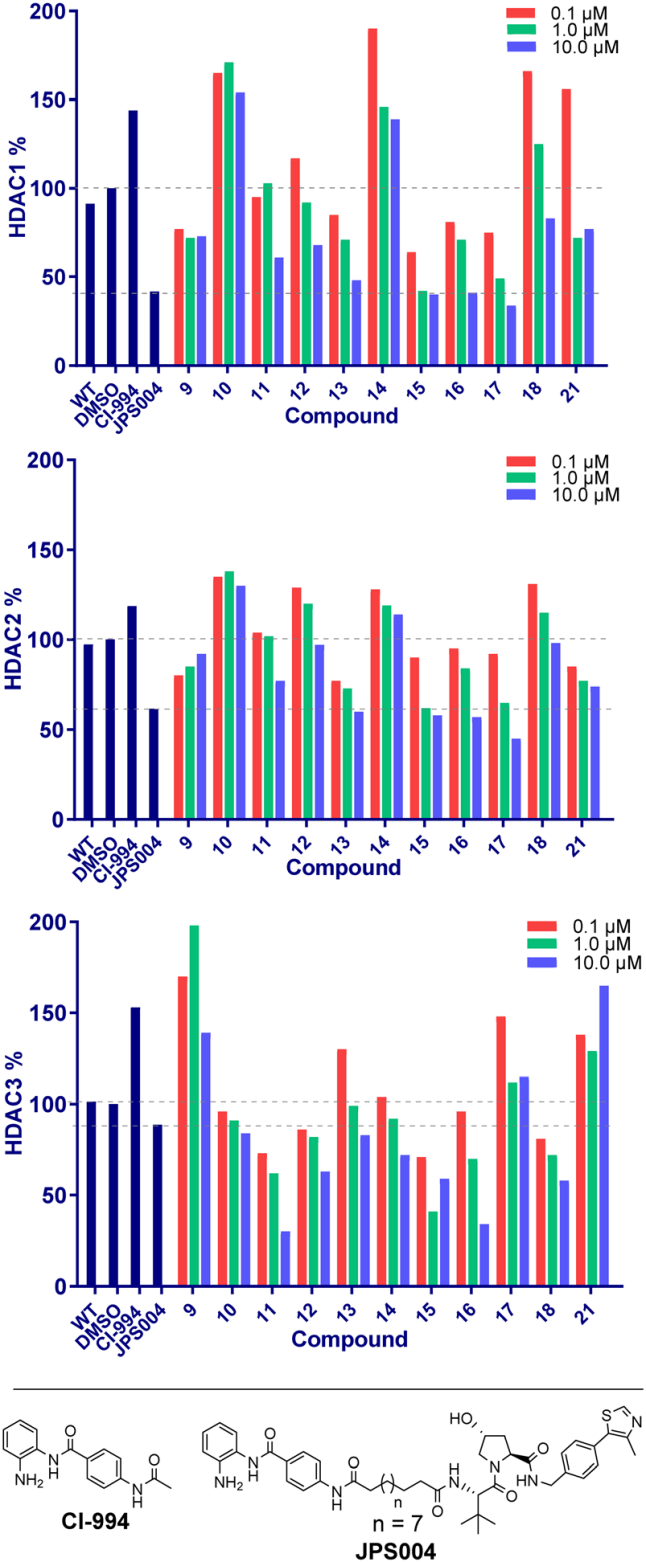
Compounds were screened at 0.1, 1.0 and 10 μM and HDAC1/2 and 3 abundance quantified by fluorescence western blotting relative to DMSO. CI-994 is a class I selective HDAC inhibitor and JPS004 a previously identified class I HDAC degrader.

We next investigated the ability of the compounds to increase histone 3 lysine 56 (H3K56ac) acetylation levels. H3K56ac was chosen as it is an established substrate and biomarker for class I HDACs,^25,26^ and we would expect an increase in H3K56ac levels resulting from the inhibition or degradation of class I HDACs.^12^ After 24 hours treatment with each compound at 10 μM, 1 μM and 0.1 μM nucleus extracts were quantified for H3K56 acetylation.

We were pleased to see a good correlation between the more active HDAC1/2 degraders and increased H3K56 acetylation levels (see ESI† for full blots). Only compounds 13, 15 & 17, the most active HDAC1/2 degraders and non-fluorinated analogues significantly increased H3K56ac levels greater than DMSO controls. Of these three compounds 17 resulted in the greatest fold change in H3K56ac levels (Fig. 3). However, we note these PROTACs, including 17, did not increase H3K56ac levels to the same levels as CI-994 or JPS004, suggesting these compounds are less potent at engaging class I HDACs in the cell and increasing H3K56 acetylation levels. We next sought to determine the DC_50_ values of active degraders identified in the first screen. Compounds 13 and 15 exhibited HDAC1/2 & 3 degradation in a dose dependent manner (see ESI†), however maximum degradation generally plateaued at approximately 50% or less at 10 μM. Compound 17 was the only PROTAC to infer greater than 50% HDAC1 degradation at 10 μM. Compound 17, herein also referred to as JMC-137, exhibited a dose dependent degradation of HDAC1/2. The DC_50_ was determined as 2.84 ± 0.12 μM for HDAC1, not determined for HDAC2 (<50% degradation at 10 μM), with the maximal degradation values (*D*_max_) for HDAC1 and HDAC2 reaching 62% and 37% respectively (average of three independent biological replicates). Regards HDAC3 degradation maximum degradation is achieved at 2.5 μM, however at concentrations greater than 2.5 μM HDAC3 levels increase, suggestive of a hook effect that we have previously observed in other PROTACs with the linker bonded to the l-*tert*-leucine residue of VHL.^15^ However, this hook effect is less pronounced with 17, and this is also the least HDAC3 degradation we have observed with class I HDAC PROTACs suggesting that modifications to the linker may be a possible strategy to infer HDAC1/2 degradation over HDAC3 degradation. To confirm degradation was occurring *via* the VHL E3-ligase ligand we also synthesised 22 (see ESI† for synthesis), with the stereochemistry of the hydroxyl group inverted reducing affinity for the VHL E3-ligase which should result in a reduction in degradation if degradation is occurring *via* the VHL E3-ligase. We were pleased to see that HDAC1/2 degradation was compromised with 22 at 10, 5 and 2.5 μM compared to 17 (JMC-137) providing evidence that JMC-137 is recruiting the VHL E3-ligase for degradation (Fig. 4C).

**Fig. 3:**
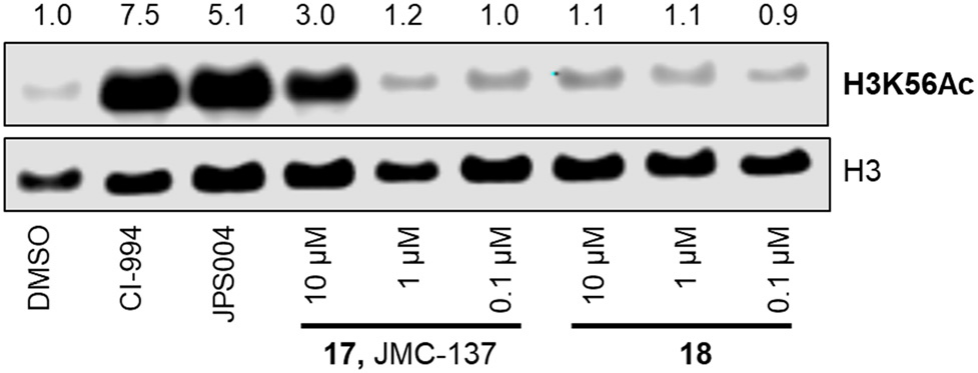
H3K56 acetylation levels and fold change in the presence of CI-994, JPS004, 17 and 18.

**Fig. 4:**
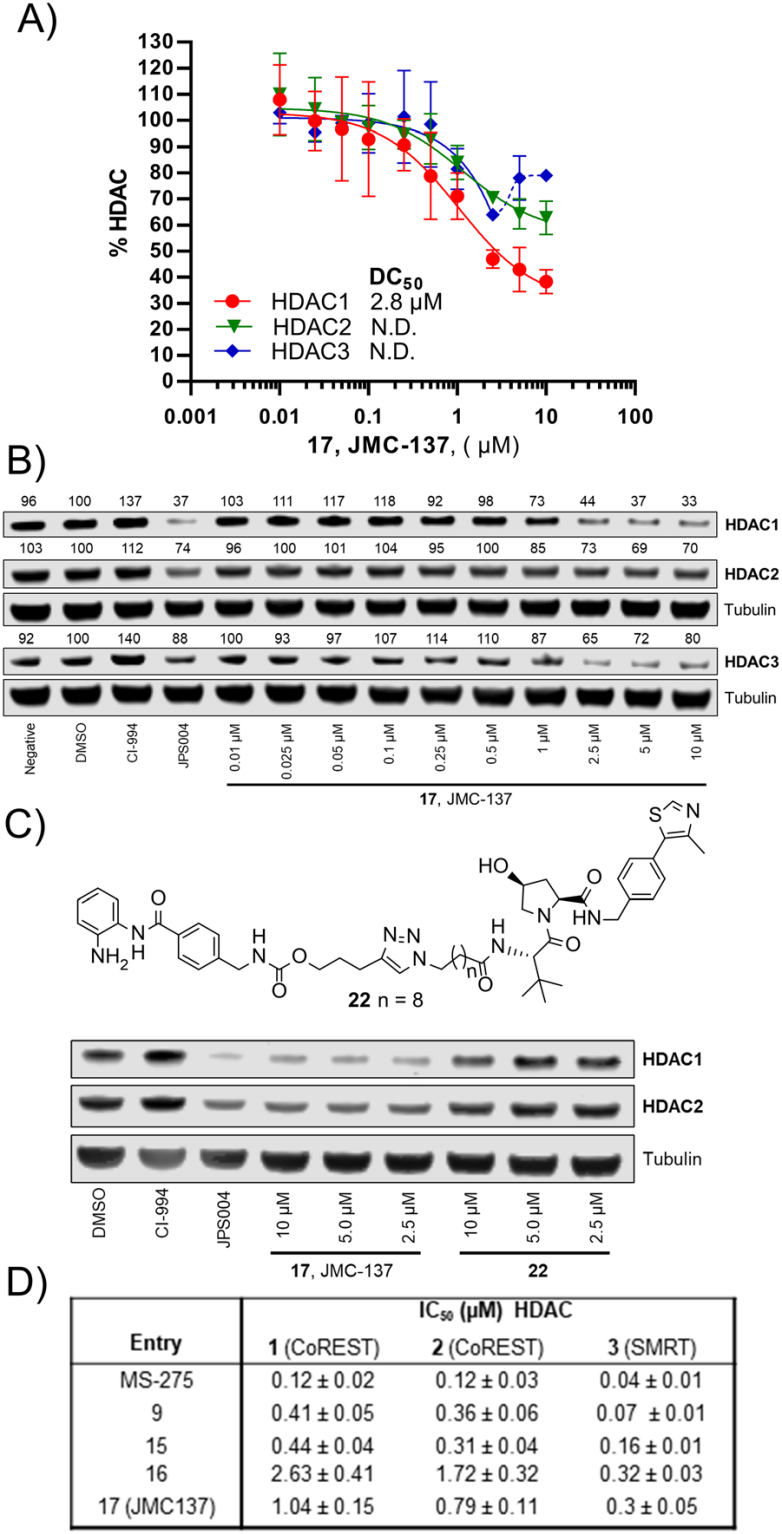
A) DC_50_ dose response curve for JMC-137, error bars representing the average of three independent biological replicates for HDAC1 and HDAC2 and two independent biological replicates for HDAC3. B) Representative quantitative fluorescence western blot showing HDAC1, HDAC2 and HDAC3 degradation by 17 (JMC-137). C) Western blot comparing HDAC1/2 degradation with JMC-137 and negative control VHL epimer 22. D) *in vitro* HDAC inhibition assay IC_50_ values in the presence of HDAC1-CoREST-LSD1, HDAC2-CoREST-LSD1, and the HDAC3-SMRT complex, see ESI† for full dose response curves.

Finally, we performed *in vitro* HDAC inhibition assays as previously reported with HDAC1-CoREST-LSD1, HDAC2-CoREST-LSD1, and the HDAC3-SMRT complex (Fig. 4D).^15^ These assays were performed in the presence of MS-275, 9, 15, 16, and 17 (JMC-137). These subset of molecules were chosen to determine the different effects on HDAC inhibition with differing linker length, triazole position, and incorporation of the fluorine atom on the benzamide HDAC ligand. MS-275 exhibited submicromolar inhibition of all three HDAC containing complexes as expected, with marginal selectivity for the HDAC3-SMRT complex. Replacement of the pyridine in MS-275 with the triazole, six carbon linker and VHL ligand present in 9 resulted in an approximately 3–4 fold loss in IC_50_ values with the HDAC1 and HDAC2 containing complexes compared to MS-275, however submicromolar inhibition was still maintained. Hence, 9 is a submicromolar class I HDAC inhibitor *in vitro*, however this heterobifunctional molecule with a shorter linker does not degrade class I HDACs in cells, similar to what we have observed previously, again highlighting the importance in linker length for degradation in cells.^12^ Increasing the linker length to 12 atoms in 15 resulted in little change in IC_50_ values compared to 9, yet 15 did act as a class I HDAC degrader in cells (Fig. 2), further reinstating the importance of the linker length. Incorporating the fluorine atom in the benzamide 16 as expected resulted in enhanced HDAC3-SMRT inhibition over the HDAC1 and HDAC2 containing complexes. Interestingly, 17 (JMC-137) resulted in a 2–2.5 fold reduction in HDAC inhibition *in vitro* compared to 15 and 9, yet 17 was the most effective HDAC1/2 degrader identified in the library. This highlights that more potent inhibition does not necessarily correlate with more potent degradation, 17 only differs from 15 in the positon of the triazole in the linker.

For the first time we have designed and synthesised class I HDAC PROTACs based on the inhibitor entinostat (MS-275), a class I HDAC inhibitor currently in clinical trials, utilising click chemistry. This strategy successfully yielded class I HDAC1/2 and HDAC3 degraders, however potency seems to be compromised, as in previous studies utilising the inhibitor CI-994 in PROTAC design we were able to identify submicromolar degraders of HDAC1 and HDAC3, and low micromolar degraders of HDAC2.^15^ Unfortunately, incorporating the carbamate and heterocycle groups of entinostat into PROTACs did not facilitate the use of shorter linker lengths less than 12 atoms. It is tempting to speculate that the necessity for the 12 atom linker, in combination with the additional carbamate and triazole functional groups, significantly enhances the molecular weight (molecular weights close to 1000) effecting cell permeability. This would reflect the reduced increase in H3K56 acetylation levels compared to CI-994 and JPS004 observed. Compounds 9, 15 and 17 are also more potent class I HDAC inhibitors *in vitro* than class I HDAC degraders in cells, which also may suggest limited cell permeability of this series of molecules. Additionally, in select PROTAC studies incorporation of the triazole negatively effects cell permeability of the PROTAC,^27,28^ which we also cannot rule out.

The presence of the carbamate and triazole moiety may also effect ternary complex formation, if the linker is not perturbed in an orientation favourable for ternary complex formation then degradation may be compromised, and the longer linker lengths would still be required as observed in this study. This may reflect the observation that the more effective HDAC1/2 degrader 17 JMC-137 of the series contains the triazole group positioned further away from the carbamate moiety and HDAC ligand than the rest of the compounds in the library, 15 only differs to 17 in the position of the triazole. Interestingly, 17 is a less potent class I HDAC inhibitor *in vitro* than 15, yet 17 is a more potent degrader than 15 in cells which would also support this theory. It is also important to note that in the future development of class I HDAC1/2 degraders that exhibit selectivity for specific HDAC1/2 containing complexes it would perhaps be expected to see more modest effects on class I HDAC degradation in terms of HDAC1 and HDAC2 maximum degradation values, and more modest increases in histone acetylation levels.

## Conflicts of interest

The authors declare the following competing financial interest(s): J. M. C., J. P. S., S. M. C., and J. T. H. are inventors on the PCT patent application WO2021148811A1, HDAC Degrader.

## Supplementary Material

MD-013-D2MD00199C-s001
