# Supplementary material for: A ‘click’ chemistry approach to novel entinostat (MS-275) based class I histone deacetylase proteolysis targeting chimeras
Source: RSC Med Chem. 2022 Nov 1;13(12):1634–9. doi: 10.1039/d2md00199c (PMC9749924; doi:10.1039/d2md00199c)
Supplement: MD-013-D2MD00199C-s001 [file MD-013-D2MD00199C-s001.pdf]

Electronic Supplementary Material (ESI) for RSC Medicinal Chemistry.
This journal is © The Royal Society of Chemistry 2022

A 'Click' Chemistry Approach to Novel Entinostat (MS-275) based Class I Histone Deacetylase Proteolysis Targeting Chimeras

Jasmine M. Cross,[a] Megan E. Coulson,[a,b] Joshua P. Smalley,[a] Wiktoria A. Pytel,[a.b] Ozair Ismail,[a] Justin S. Trory,[a] Shaun M. Cowley,[b*] and James T. Hodgkinson[a*]

a. Leicester Institute of Structural and Chemical Biology, School of Chemistry, University of Leicester, Leicester, LE1 7RH, UK

b. Department of Molecular and Cell Biology, University of Leicester, Leicester, LE1 7RH, UK

Contents

Supplementary Information: Biology 3

1. Cell Lines and Materials 3
2. Western Blotting 3
   2.1. Western Blots for Three-Point Dilution Screening Assay 4
3. In vitro HDAC assays 11

Supplementary Information: Chemistry 14

4. General Methods 14
5. Compound Characterisation 15

References 48

Appendix: ^{1}H NMR and ^{13}C NMR of Novel Compounds 49

Supplementary Information: Biology

1. Cell Lines and Materials

HCT116 human colon carcinoma cells were grown in Dulbecco's Modified Eagle Medium (DMEM) (GIBCO, 41965-039) supplemented with 10% Fetal Bovine Serum (FBS) (Sigma) and 1X glutamine/penicillin/streptomycin (GIBCO, 10378-016). HCT116 cells were incubated at 37 ˚C with 5% CO_2. Cells were treated with PROTACs (0.01-10 µM) alongside HDAC inhibitors CI-994 (10 µM) and JPS004 (10 µM) as controls.

2. Western Blotting

HCT116 cells were treated 24 hours after seeding. 24 hours post treatment, cells were harvested, lysed in lysis buffer (50mM Tris-HCl, 150 mM NaCl, 0.5% NP-40, 0.5% Triton X-100) with protease inhibitor (Sigma, P8340), then incubated on ice for 30 minutes, before being centrifuged (14,000 rpm, 15 minutes, 4 ˚C). The supernatant was collected, and protein concentrations quantified via Bradford Assay using Protein Assay Dye Reagent Concentrate (BIO-RAD). For histone extraction, 75 % volume of 0.4 N H2SO4 was added to the pellets and the extracts placed at 4 ˚C overnight. Following overnight incubation, the tubes were centrifuged (14,000 rpm, 15 minutes, 4 ˚C) and then the supernatant (histone extract) collected.

Western blots were run on NuPAGETM 4-12% Bis-Tris gels with 20-30 µg of protein or 10 µL of acid-extracted histone loaded per lane, using NuPAGETM LDS Sample Buffer (4X). PageRulerTM Plus Prestained Ladder was used for size standards. After gel electrophoresis at 140V for 90 minutes the separated proteins were transferred onto nitrocellulose membrane at 30V for 60 minutes. The membranes were probed with primary antibodies (listed below) for 60 minutes. Blots were developed with complimentary IRDye conjugated secondary antibodies and the bands visualised using the Odyssey Infrared Imaging System. Image processing and band intensity quantification was performed using Image Studio Lite. DC_{50} values were obtained by fitting D_{max} values to a variable slope dose–response model in GraphPad Prism.

Antibody Information

Primary Antibodies;

α-tubulin - Sigma, t5168 (1:10,000 dilution)

HDAC1 - Abcam, 109411 (1:2,000 dilution)

HDAC2 - Merck Millipore, 05-814 (1:2,000 dilution)

HDAC3 - Abcam, 32369 (1:2,000 dilution)

H3 - Merck Millipore, 05-499 (1:1,000 dilution)

H3K56Ac - Active Motif, 39281 (1:1,000 dilution)

Secondary Antibodies;

IRDye® 680LT - LI-COR Biosciences, 926-68023 (1:10,000 dilution)

IRDye® 800CW - LI-COR Biosciences, 926-32210 (1:10,000 dilution)

2.1. Western Blots for Three-Point Dilution Screening Assay

Figure S1: Immunoblots with HDAC 1, 2, and 3 antibodies after 24h in HCT116 cell line. Numerical value represents percentage of protein compared to DMSO control = 100%. CI-994 = 10µM, JPS004 = 10 µM. PROTACs dosed at three-point dilutions (10, 1.0 and 0.1 µM)

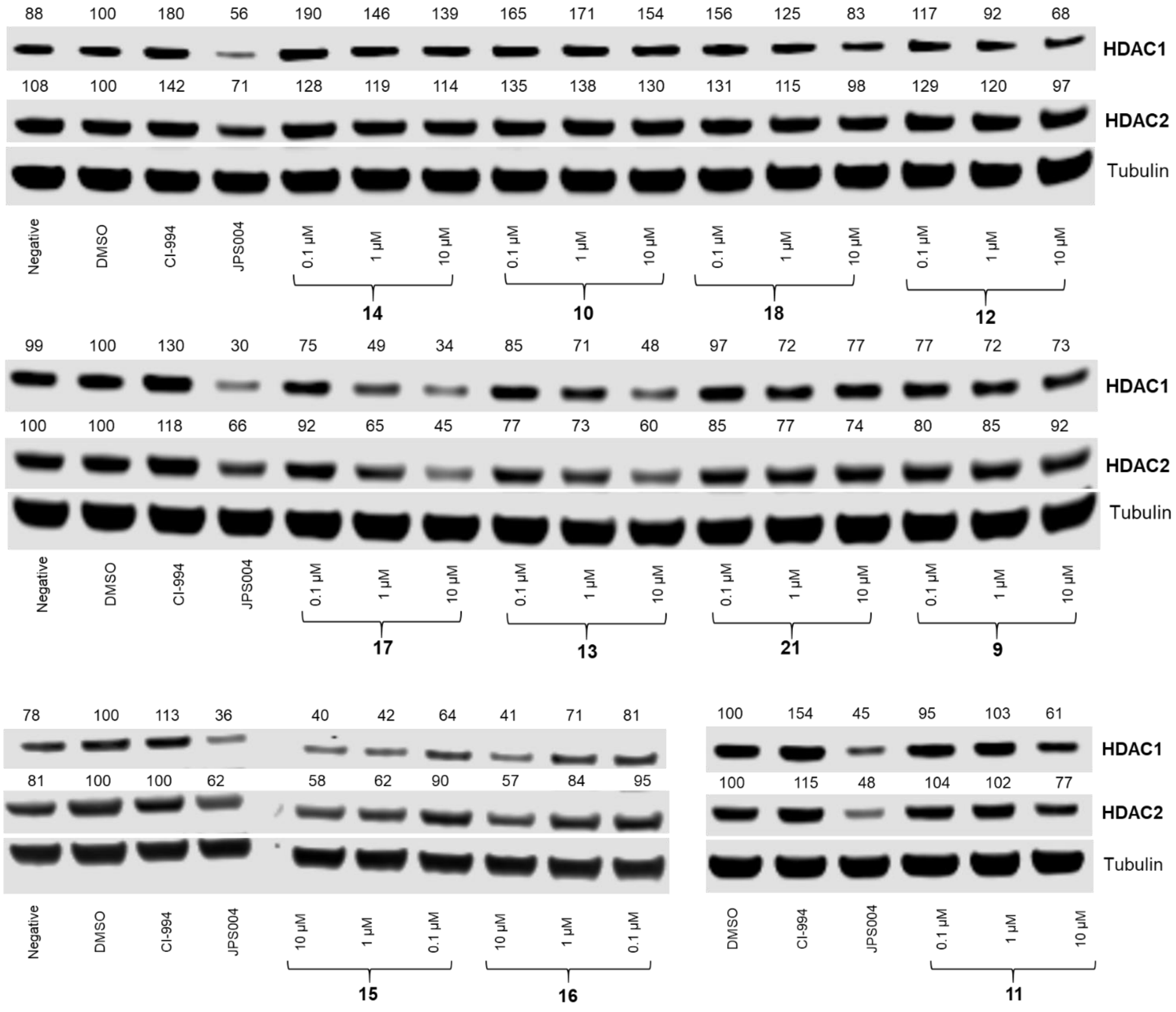

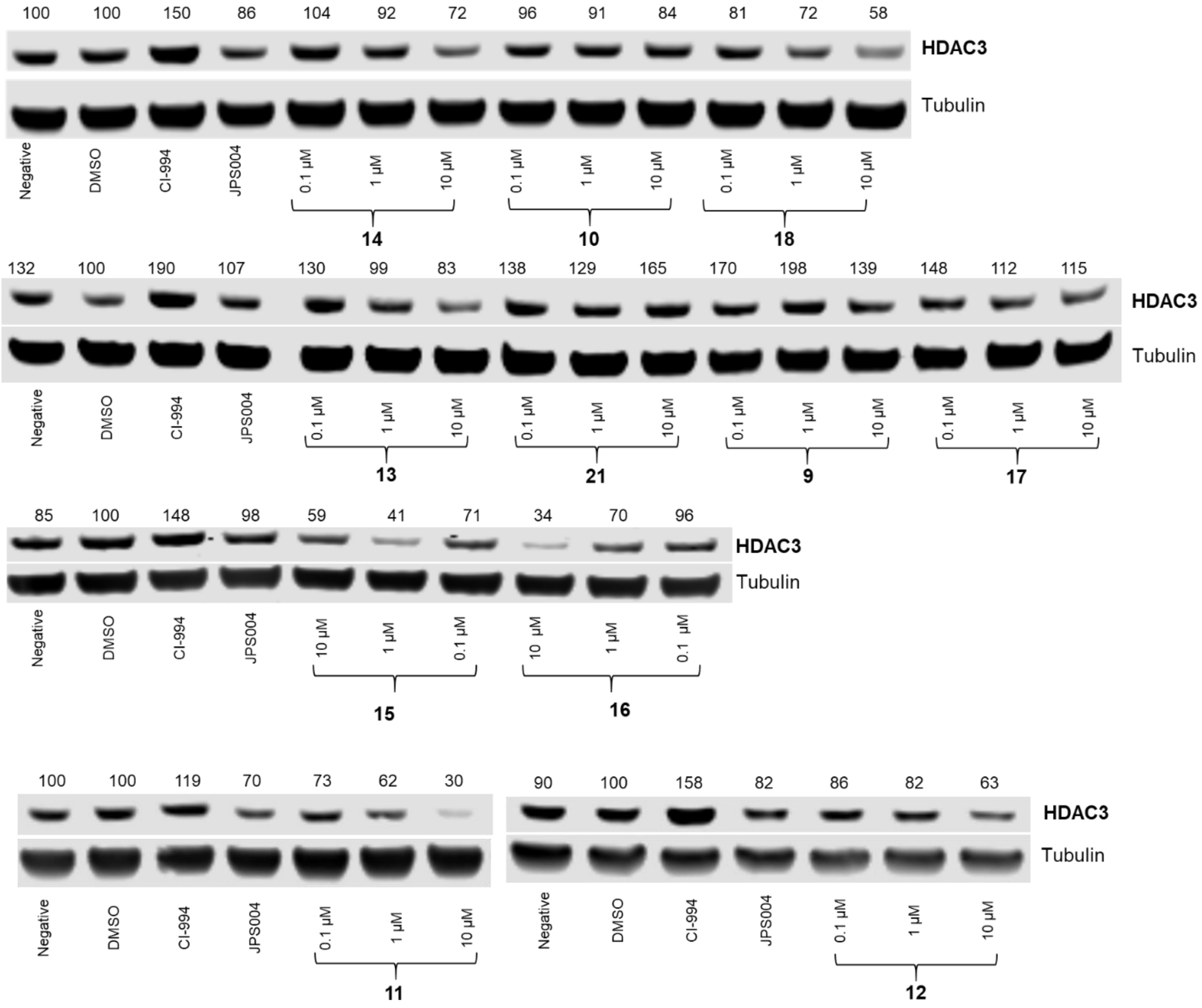
100 100 150 86 104 92 72 96 91 84 81 72 58
HDAC3
Tubulin
Negative DMSO CI-994 JPS004 0.1 µM 1 µM 10 µM 0.1 µM 1 µM 10 µM 0.1 µM 1 µM 10 µM
14
10
18
132 100 190 107 130 99 83 138 129 165 170 198 139 148 112 115
HDAC3
Tubulin
Negative DMSO CI-994 JPS004 0.1 µM 1 µM 10 µM 0.1 µM 1 µM 10 µM 0.1 µM 1 µM 10 µM 0.1 µM 1 µM 10 µM
13
21
9
17
85 100 148 98 59 41 71 34 70 96
HDAC3
Tubulin
Negative DMSO CI-994 JPS004 10 µM 1 µM 0.1 µM 10 µM 1 µM 0.1 µM
15
16
100 100 119 70 73 62 30
Negative DMSO CI-994 JPS004 0.1 µM 1 µM 10 µM
11
90 100 158 82 86 82 63
HDAC3
Tubulin
Negative DMSO CI-994 JPS004 0.1 µM 1 µM 10 µM
12

Figure S2: Histone 3 lysine 56 acetylation (H3K56Ac) levels in HCT116 cells after 24h, histone 3 (H3) levels measured as a control; A) CI-994 = 10 µM, JPS004 = 10 µM, PROTACs **9**, **10**, **21**, **11** and **12**. B) Fold change in H3K56Ac levels shown.

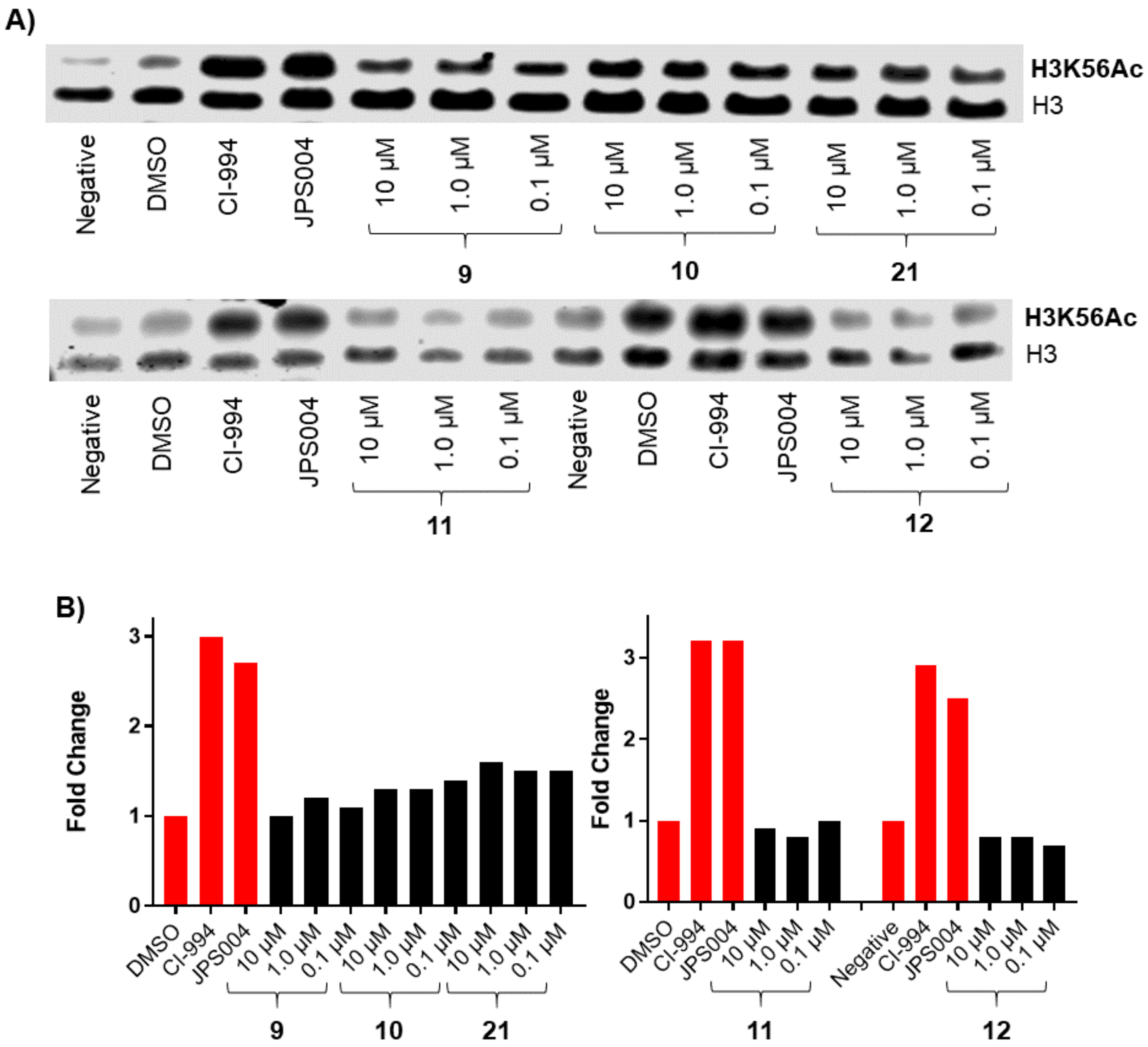

Figure S3: Histone 3 lysine 56 acetylation (H3K56Ac) levels in HCT116 cells after 24h, histone 3 (H3) levels measured as a control; A) CI-994 = 10 μM, JPS004 = 10 μM, PROTACs **13**, **14**, **15** and **16**. B) Fold change in H3K56Ac levels shown.

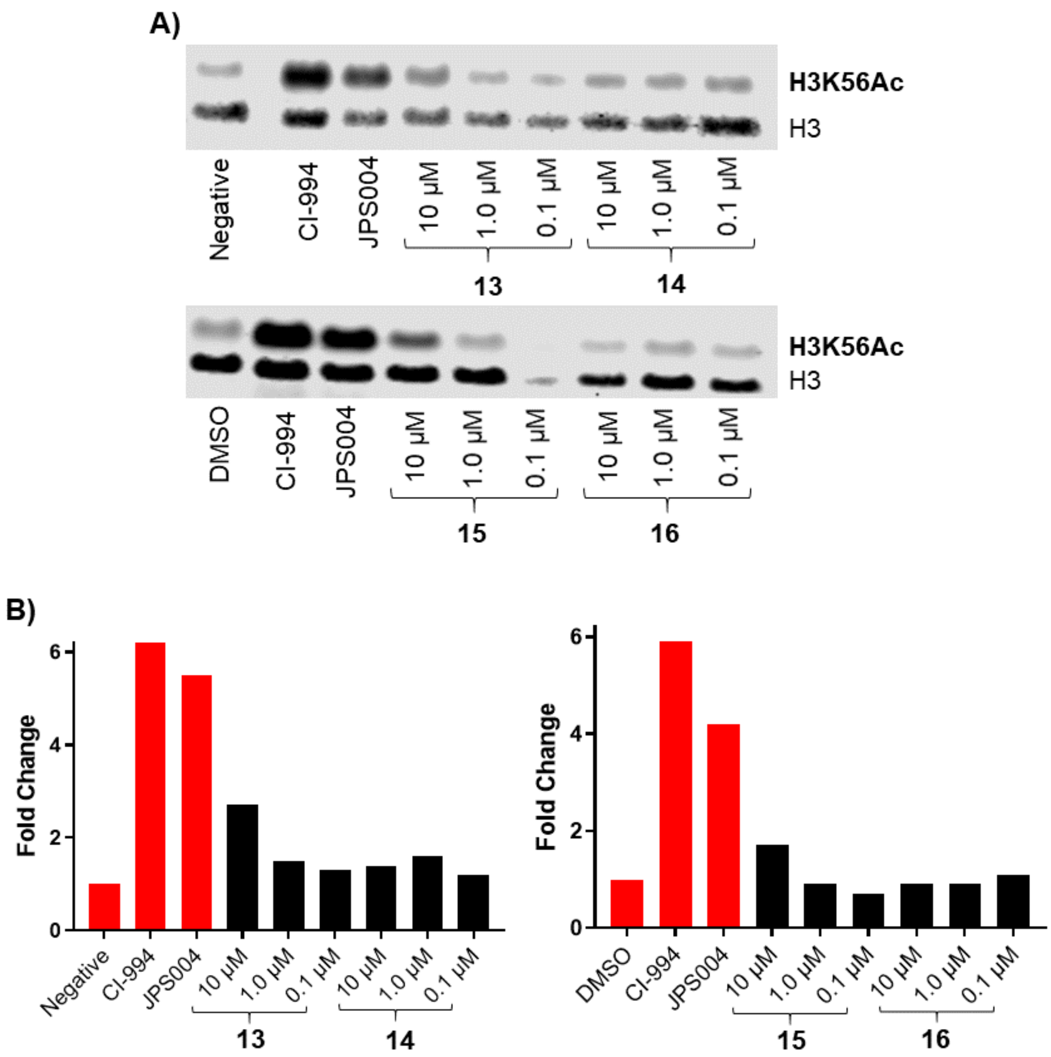

Figure S4: Histone 3 lysine 56 acetylation (H3K56Ac) levels in HCT116 cells after 24h, histone 3 (H3) levels measured as a control; A) CI-994 = 10 µM, JPS004 = 10 µM, PROTACs **17** and **18**. B) Fold change in H3K56Ac levels shown.

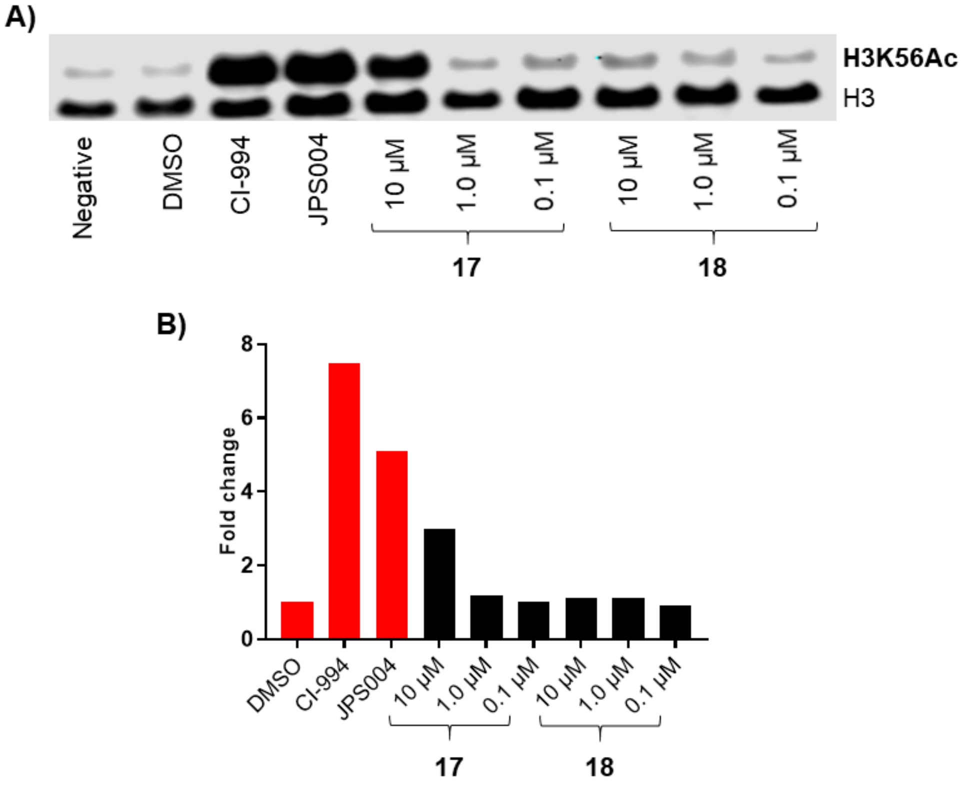

Figure S5: A) Immunoblot with HDAC 1, 2 & 3 antibodies after 24h in HCT116 cell line. Numerical value represents percentage of protein compared to DMSO control = 100%. CI-994 = 10µM, JPS004 = 10 µM. PROTAC **13** was dosed in a ten-point dilution range from 10-0.01 µM.

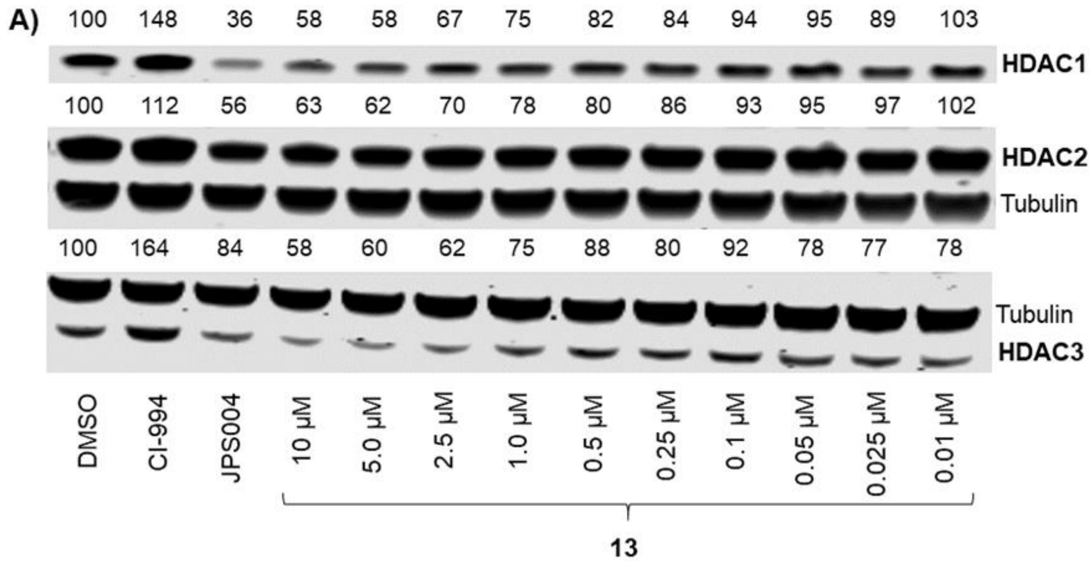

Figure S6: A) Immunoblot with HDAC 1, 2 & 3 antibodies after 24h in HCT116 cell line. Numerical value represents percentage of protein compared to DMSO control = 100%. CI-994 = 10µM, JPS004 = 10 µM. PROTAC **15** was dosed in a ten-point dilution range from 10-0.01 µM.

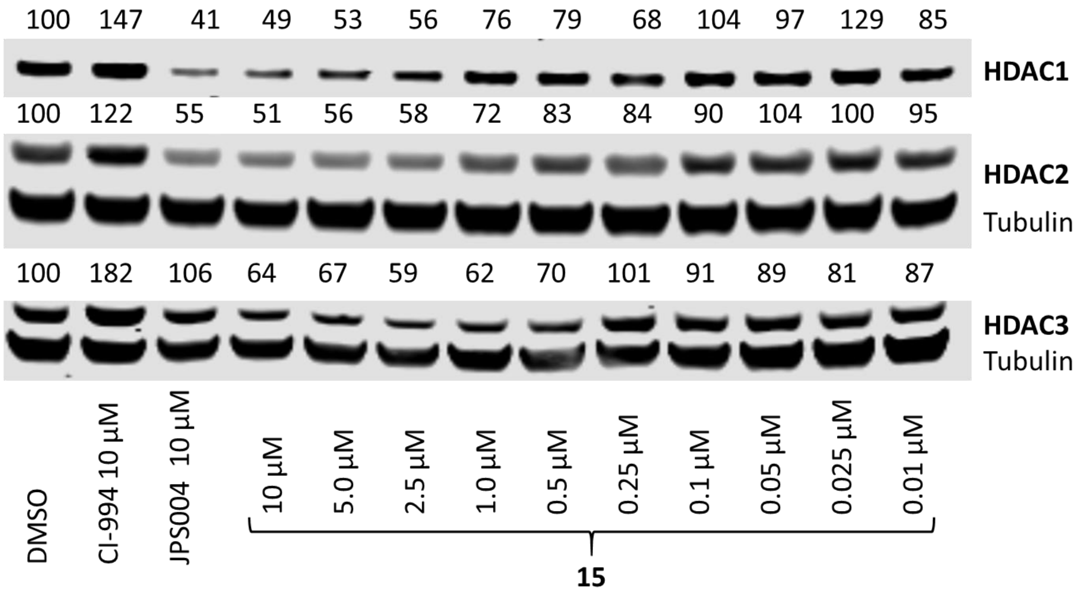

Figure S7: A) Immunoblot with HDAC 1, 2 & 3 antibodies after 24h in HCT116 cell line. Numerical value represents percentage of protein compared to DMSO control = 100%. CI-994 = 10µM, JPS004 = 10 µM. PROTAC **17** (JMC-137)was dosed in a ten-point dilution range from 10-0.01 µM.

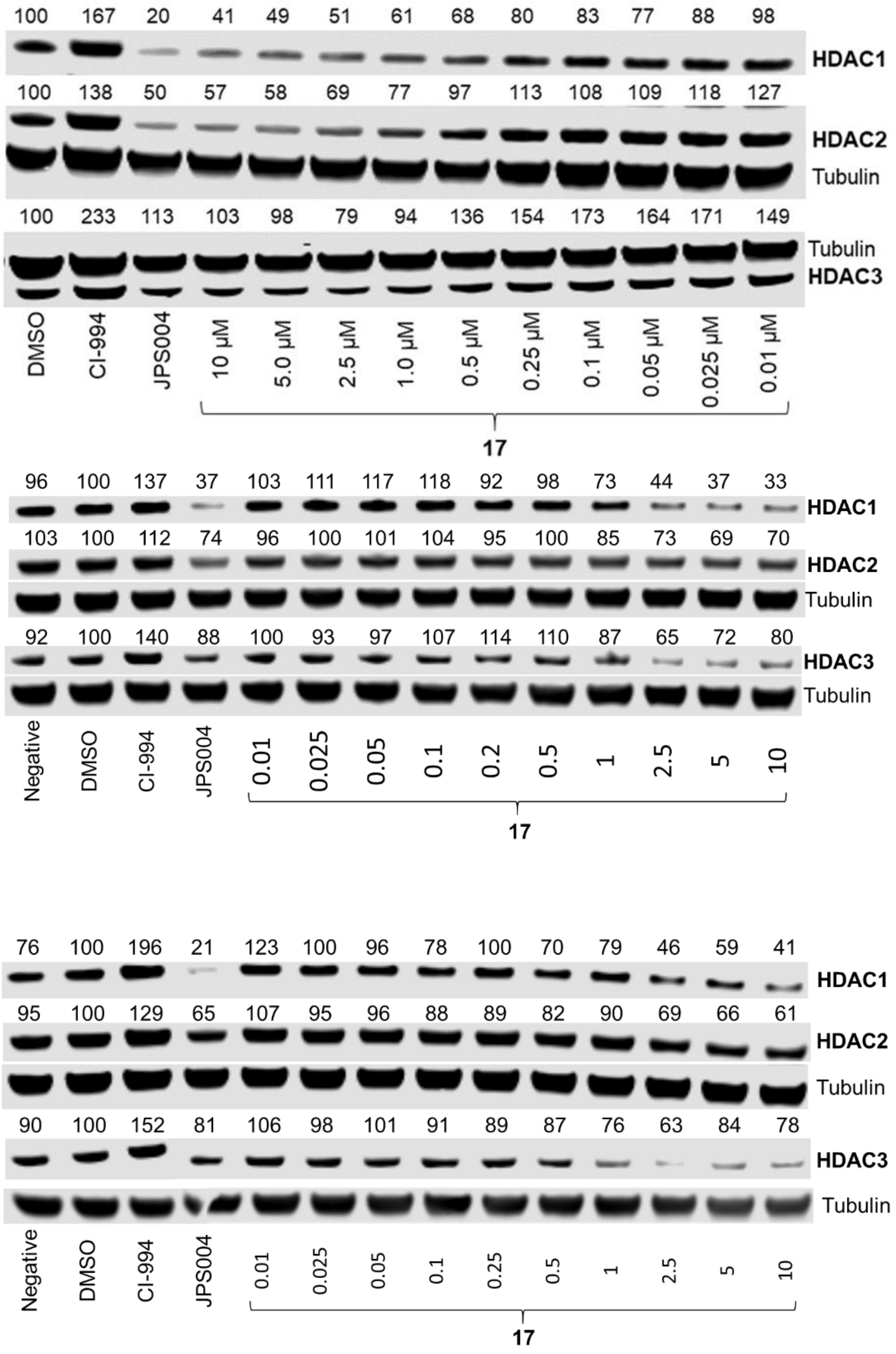

3. In vitro HDAC assays

In vitro inhibition assays with HDAC1-CoREST-LSD1, HDAC2-CoREST-LSD1 and HDAC3-SMRT-DAD were performed using a fluorescent HDAC assay. The assays were performed on black 96-well plates (Corning) with a reaction volume of 50 µL. HDAC complexes were expressed and purified as described previously.[1,2] 100 µM of Boc- (Ac)Lys-AMC was used as the substrate. All determinations were performed in triplicate. The compounds were dissolved at 50 mM in DMSO, and then diluted again with 10% DMSO HDAC assay buffer (10% DMSO, 50 mM Tris pH 7.5, 50 mM NaCl, 0.1 mg/mL BSA) to micromolar concentrations. 1:3 serial dilutions were then carried out using 10% DMSO HDAC assay buffer to afford range of concentrations. 10 µL of each of these solutions was then added to individual wells, followed by the addition of 30 µL of the HDAC complex (12.5 nM) in assay buffer (50 mM Tris pH 7.5, 50 mM NaCl, 0.1 mg/mL BSA). The plate was then incubated at 20 ˚C, 100 RPM for 1 hour. After incubation, 10 µL of the Boc-(Ac)Lys-AMC substrate, dissolved in HDAC assay buffer, was added to each well. The plate was incubated at 30 ˚C, 100 RPM for 1 hour, followed by addition of 50 µL of a developer buffer (50 mM Tris pH 7.5, 100 mM NaCl, 10 mg/mL trypsin) to stop the reaction. The reaction was allowed to develop for 10 minutes at 30˚C, 100 RPM. Fluorescence intensity was determined with the Victor X5 plate reader (Perkin Elmer, λex = 335 nm, λem = 460 nm). IC50 values were calculated through the GraphPad Prism 7 software by non-linear regression, log (inhibitor) vs. response – variable slope (four parameters).

MS-275

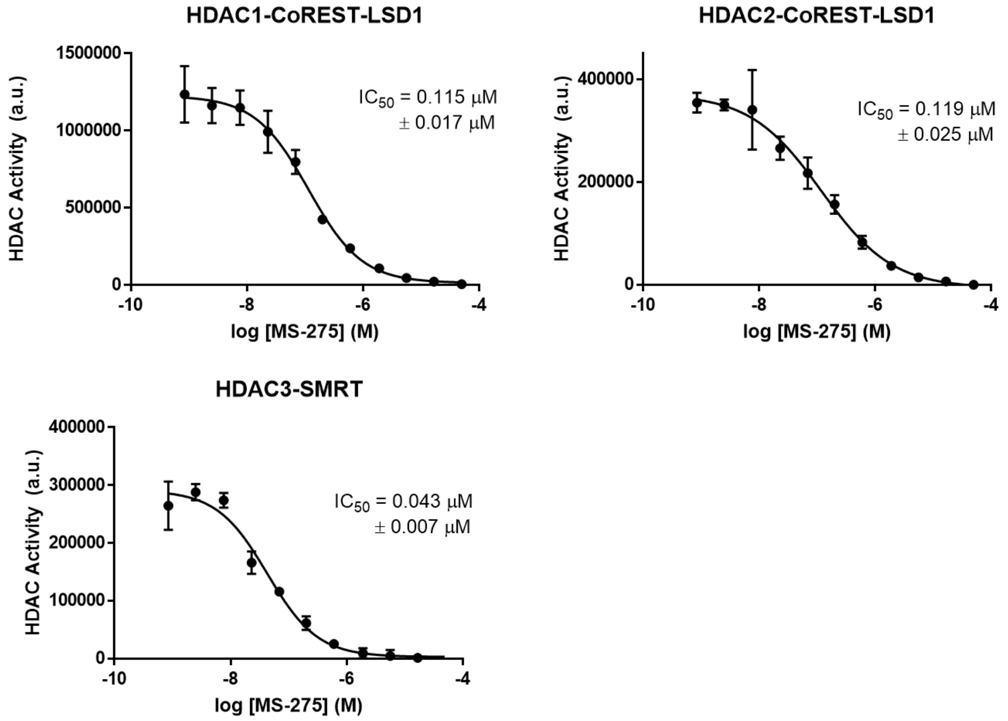

9

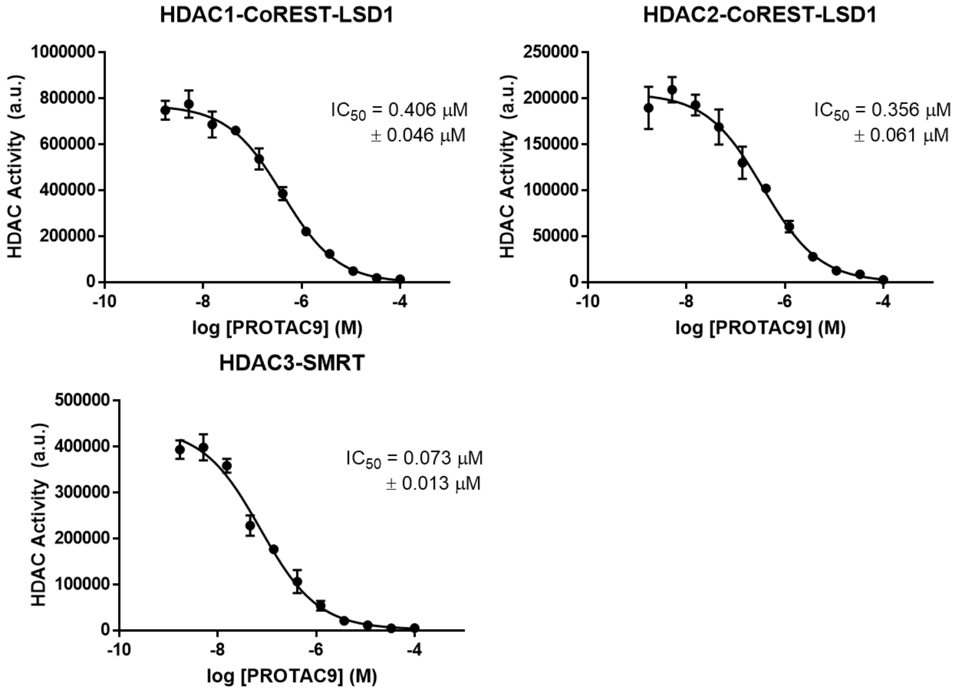
HDAC1-CoREST-LSD1
HDAC Activity (a.u.)
log [PROTAC9] (M)
IC50 = 0.406 µM
± 0.046 µM
HDAC2-CoREST-LSD1
HDAC Activity (a.u.)
log [PROTAC9] (M)
IC50 = 0.356 µM
± 0.061 µM
HDAC3-SMRT
HDAC Activity (a.u.)
log [PROTAC9] (M)
IC50 = 0.073 µM
± 0.013 µM

15

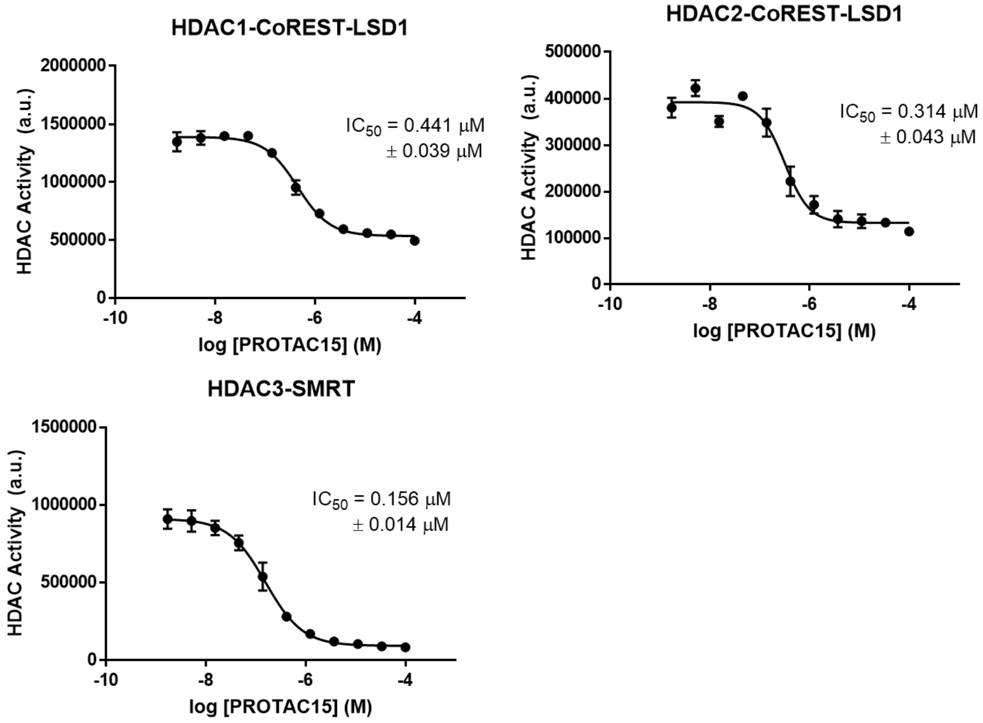
HDAC1-CoREST-LSD1
HDAC Activity (a.u.)
log [PROTAC15] (M)
IC50 = 0.441 µM
± 0.039 µM
HDAC2-CoREST-LSD1
HDAC Activity (a.u.)
log [PROTAC15] (M)
IC50 = 0.314 µM
± 0.043 µM
HDAC3-SMRT
HDAC Activity (a.u.)
log [PROTAC15] (M)
IC50 = 0.156 µM
± 0.014 µM

16

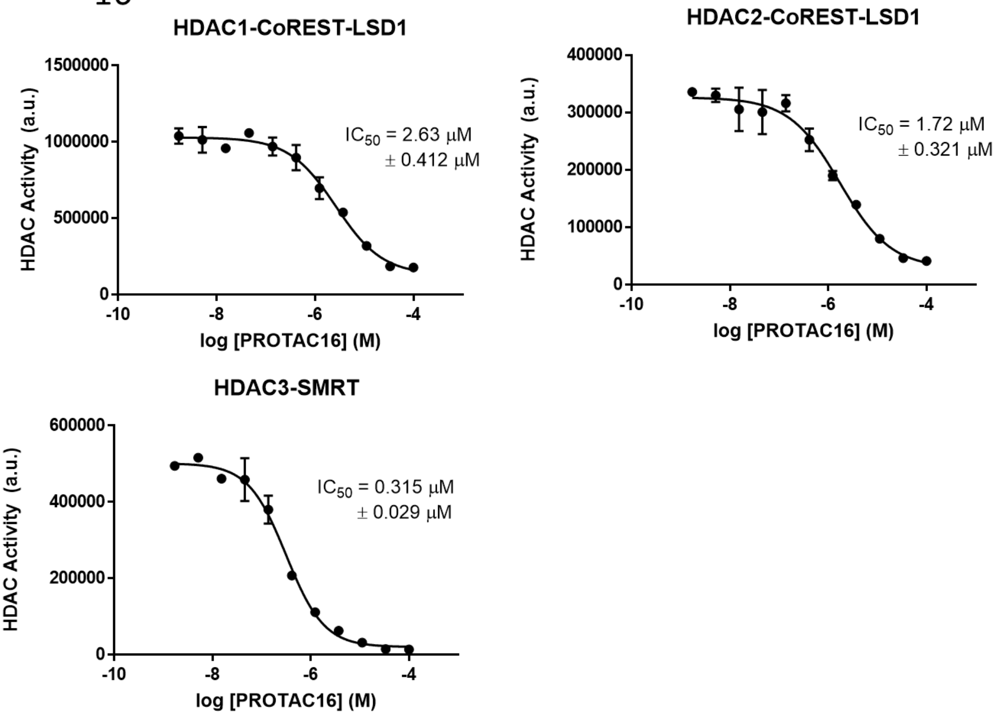
HDAC1-CoREST-LSD1
HDAC Activity (a.u.)
log [PROTAC16] (M)
IC$_{50}$ = 2.63 μM
± 0.412 μM
HDAC2-CoREST-LSD1
HDAC Activity (a.u.)
log [PROTAC16] (M)
IC$_{50}$ = 1.72 μM
± 0.321 μM
HDAC3-SMRT
HDAC Activity (a.u.)
log [PROTAC16] (M)
IC$_{50}$ = 0.315 μM
± 0.029 μM

17 (JMC-137)

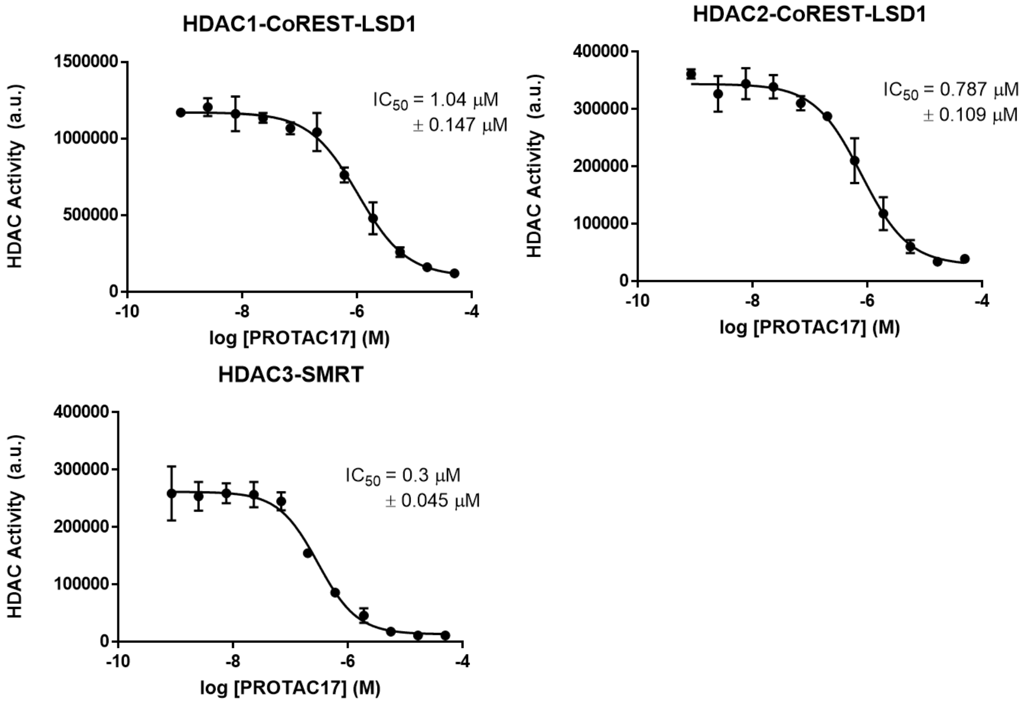
HDAC1-CoREST-LSD1
HDAC Activity (a.u.)
log [PROTAC17] (M)
IC$_{50}$ = 1.04 μM
± 0.147 μM
HDAC2-CoREST-LSD1
HDAC Activity (a.u.)
log [PROTAC17] (M)
IC$_{50}$ = 0.787 μM
± 0.109 μM
HDAC3-SMRT
HDAC Activity (a.u.)
log [PROTAC17] (M)
IC$_{50}$ = 0.3 μM
± 0.045 μM

Supplementary Information: Chemistry

4. General Methods

All reagents and solvents were obtained from Sigma Aldrich, Acros Organics, Apollo Scientific, Fluorochem, Fisher Scientific and were used as supplied unless stated otherwise. The active VHL ligand (4*R*)-3-Methyl-L-valyl-4-hydroxy-*N*-[[4-(4-methyl-5-thiazolyl)phenyl]methyl]-L-prolinamide hydrochloride were purchased from MedChemExpress® and inactive (negative control) VHL ligand (2*S*,4*S*)-1-[(2*S*)-2-Amino-3,3-dimethyl-butanoyl]-4-hydroxy-*N*-[[4-(4-methylthiazol-5-yl)phenyl]methyl]pyrrolidine-2-carboxamide dihydrochloride purchased from TOCRIS. Aniline derivatives of substructure **3** were synthesised using previously undertaken methods.[3] Azide compounds for subsequent Click reactions were synthesised using known literature methods, with the exception of the readily available 6-azidohexanoic acid.[4,5] Biotage® Macroporous polystyrene-co-divinylbenzene (MP) carbonate resin (3.02 mmol/g loading capacity) was used for neutralizing amine TFA salts and scavenging excess TFA during tert-butoxycarbonyl deprotection reactions. Glassware was dried in oven at 100 °C for 16 hours for moisture sensitive reactions. Unless otherwise stated reactions were performed under nitrogen using anhydrous solvents. Dried THF and DCM were dried using an Innovative Technology inc. PureSolv solvent purification system. Room temperature refers to ambient temperature. Temperatures of 0 °C were maintained using an ice-water bath. The reactions were monitored by thin-layer chromatography (TLC) on aluminium backed silica gel. Unless otherwise stated Flash column chromatography was carried out with Silica Gel 60 using commercial solvents. All evaporations *in vacuo* were performed under reduced pressure using a Büchi rotary evaporator. All chemical names have been generated using ChemDraw Professional. Preparative column chromatography and flash column chromatography using a Biotage Isolera purification system was perfomed using silica gel 60 (230-400 mesh).

Analytical and semi-preparative HPLC were performed on a ThermoFisher Ultimate 3000 system with Chromeleon software on a Phenomenex Luna 5 µM C18 column. Method 1, A= H_2O, B= MeCN, 5-95% B, 10 mL/min flow, 30 min gradient. Method 2, A= H_2O, B= MeCN, 30-100% B, 10 mL/min flow, 30 min gradient. Method 3, A= H_2O, B= MeCN, 50-100% B, 10 mL/min flow, 30 min gradient. All methods measured absorbance at 214 nm. Solutions were made up in HPLC Grade acetonitrile (MeCN) and deionised water (1:1). Where semi-preparative HPLC purification has not been used, purity (>95%) of final compounds was determined by analytical HPLC.

Nuclear magnetic resonance (NMR) spectra were acquired using a Bruker 400 (^{1}H 400 MHz; ^{13}C 100 MHz, ^{19}F 376 MHz) instrument at ambient temperature using deuterated solvent as reference - $CDCl_3$

(δ_H = 7.26 ppm, δ_C = 77.00 ppm), DMSO-d_6 (δ_H = 2.50 ppm, δ_C = 39.51 ppm) or CD_3OD (δ_H = 3.31 ppm, δ_C = 49.15 ppm). ^{1}H NMR data are reported as: chemical shift, multiplicity [b, broad; s, singlet; d, doublet; t, triplet; q, quartet; quin, m for multiplet; or as a combination (e.g., dd, dt, etc.)], coupling constant(s) and integration. ^{13}C NMR spectra were recorded by broadband proton decoupling. ^{13}C NMR chemical shifts (δ) are quoted to the nearest 0.1 ppm and referenced to the residual non-deuterated solvent peak. Where ^{1}H and ^{13}C NMR's have been fully assigned, 2D NMR including ^{1}H-^{1}H COSY (correlated spectroscopy), ^{1}H-^{13}C HSQC (heteronuclear single quantum coherence) and ^{1}H-^{13}C HMBC (heteronuclear multiple bond coherence) were used to aid assignment. TopSpin 4.0 software (Chemsketch and Spectrus Processor) was used for peak picking, integration and calculating coupling constants. High resolution mass spectra (HRMS) were recorded on a Water Aquity XEVO Q ToF machine and measured in m/z.

5. Compound Characterisation

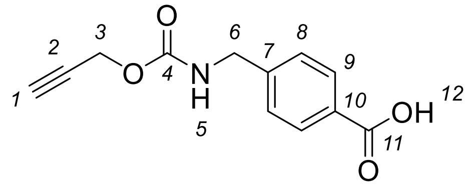

4-({[(Prop-2-yn-1-yloxy)carbonyl]amino}methyl)benzoic acid (1)

Propargyl alcohol (0.12 mL, 1.98 mmol) and carbonyldiimidazole (0.32 g, 1.98 mmol) were dissolved in THF (5 mL) and stirred at 10 °C for 1 h. 4-(Aminomethyl)benzoic acid (0.30 g, 1.98 mmol), DBU (0.29 mL, 1.98 mmol) and triethylamine (0.27 mL, 1.98 mmol) were suspended in THF (5 mL) and combined with the CDI-intermediate solution and the reaction stirred at room temperature for 16 h. The solvent was removed under reduced pressure and resuspended in H_2O (10 mL) and the solution was acidified with 1M HCl solution to pH 5. The resulting precipitate was collected by gravity filtration and air dried to provide the *title compound* as a white powder (0.35 g, 75%). ^{1}H NMR (400 MHz, DMSO-d_6) δ_H ppm 12.8 (1H, s, H^{12}), 7.99 (1H, t, J = 6.0 Hz, H^5), 7.90 (2H, d, J = 8.0 Hz, H^8), 7.36 (2H, d, J = 8.0 Hz, H^9), 4.65 (2H, d, J = 2.5 Hz, H^3), 4.27 (2H, d, J = 6.0 Hz, H^6), 3.50 (1H, t, J = 2.5 Hz, H^1); ^{13}C NMR (101 MHz, DMSO-d_6) δ_C ppm 101 MHz) 167.6 (C^{11}), 156.0 (C^4), 145.2 (C^7), 129.9 (C^{10}), 129.8 (C^8), 127.5 (C^9), 79.7 (C^2), 77.6 (C^1), 52.2 (C^3), 44.1 (C^6); *m/z* (HRMS$^+$) 234.0764 $[M+H]^+$ ($C_{12}H_{12}NO_4$ requires 234.0766).

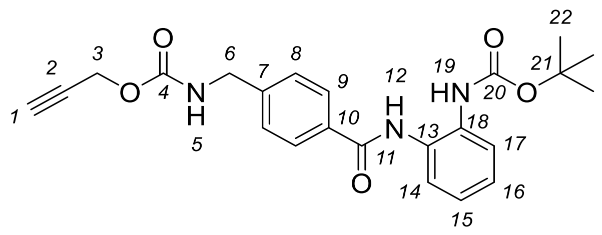

Tert-butyl N-{2-[4-({[(prop-2-yn-1-yloxy)carbonyl]amino}methyl)benzamido]phenyl}carbamate (4a)

4-({[(Prop-2-yn-1-yloxy)carbonyl]amino}methyl)benzoic acid (0.20 g, 0.85 mmol) and HATU (0.49 g, 1.28 mmol) were dissolved in anhydrous DMF (5 mL) and stirred at room temperature for 1 h. Tert-butyl N-(2-aminophenyl)carbamate (0.18 g, 0.85 mmol) and diisopropylethylamine (0.30 mL, 1.71 mmol) were added and the reaction stirred at room temperature for 16 h. The solvent was removed under reduced pressure and suspended in EtOAc (20 mL). The organic solution was washed with sat. $NaHCO_3$ (2 x 20 mL), brine (20 mL), the organic layers combined, dried over $MgSO_4$, filtered and the solvent removed under reduced pressure. The crude oil was purified by flash chromatography on silica (Hexane:EtOAc 30:70-50:50 gradient) to provide the *title compound* as a pale pink oil (0.28 g, 76%). ^1H NMR (400 MHz, $CDCl_3$) δ_H ppm 9.16 (1H, s, H^{12}), 7.93 (2H, d, J = 8.0 Hz, H^9), 7.80 (1H, d, J = 7.5 Hz, H^{14}), 7.37 (2H, d, J = 8.0 Hz, H^8), 7.26-7.21 (2H, m, $H^{15, 17}$), 7.16 (1H, ddd, J = 7.5 Hz 7.0 Hz 1.5 Hz, H^{16}), 6.77 (1H, s, H^{19}), 5.22 (1H, t, J = 6.0 Hz, H^5), 4.73 (2H, d, J = 2.5 Hz, H^3), 4.45 (2H, d, J = 6.0 Hz, H^6), 2.49 (1H, t, J = 2.5 Hz, H^1), 1.52 (9H, s, H^{22}); ^{13}C NMR (101 MHz, $CDCl_3$) δ_C ppm 165.2 (C^{11}), 155.5 (C^4), 154.6 (C^{20}), 142.1 (C^7), 133.6 (C^{10}), 131.0 (C^{18}), 129.7 (C^{13}), 127.8 (C^9), 127.5 (C^8), 126.1 (C^{17}), 125.9 (C^{16}), 125.7 (C^{14}), 124.5 (C^{15}), 81.5 (C^{21}), 77.2 (C^2), 74.8 (C^1), 52.7 (C^3), 44.8 (C^6), 28.2 (C^{22}); *m/z* ($HRMS^+$) 424.1871 $[M+H]^+$ ($C_{23}H_{26}N_3O_5$ requires 424.1872); R_f = 0.52 (70:30 EtOAc:Hexane).

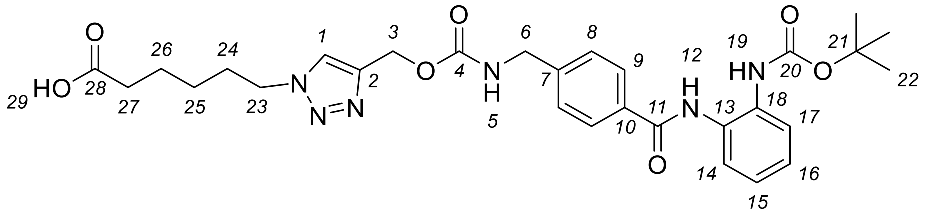

6-(4-((((4-((2-((Tert-butoxycarbonyl)amino)phenyl)carbamoyl)benzyl)carbamoyl)oxy)methyl)-1H-1,2,3-triazol-1-yl)hexanoic acid (6a)

Tert-butyl-*N*-{2-[4-({[(prop-2-yn-1-yloxy)carbonyl]amino}methyl)benzamido]phenyl}carbamate (0.10 g, 0.23 mmol), 6-azidooctanoic acid (0.04 g, 0.23 mmol) and CuI (0.01 g, 0.08 mmol) were suspended in degassed DMF:MeCN (5 mL, 2:1) under an inert atmosphere. 2,6-Lutidine (0.04 mL, 0.35 mmol) was added and the reaction stirred at room temperature for 16 h. The solvent was removed under reduced pressure and suspended in EtOAc (10 mL), washed with 1 M HCl (1 x 10 mL), sat. $NaHCO_3$ (2 x 10 mL), brine (2 x 10 mL), the organic layers combined, dried over $MgSO_4$, filtered and the solvent removed under reduced pressure. The crude oil was purified by flash chromatography on silica (3-5% MeOH:DCM solvent gradient) to provide the *title compound* as an off white solid (0.10 g, 74%). ^1H NMR (400 MHz, $CDCl_3$) δ_H ppm 9.20 (1H, bs, H^{12}), 7.81 (2H, d, J = 8.0 Hz, H^9), 7.66 (1H, d, J = 7.0 Hz, H^{14}), 7.56 (1H, s, H^1), 7.23-7.19 (3H, m, $H^{8, 16}$), 7.14-7.05 (2H, m, $H^{15, 17}$), 5.46 (1H, t, J = 5.5 Hz, H^5), 5.13 (2H, s, H^3), 4.33 (2H, d, J = 5.5 Hz, H^6), 4.26 (2H, t, J = 7.5 Hz, H^{23}), 2.22 (2H, t, J = 7.5,

H^{27}), 1.83 (2H, quin, J = 7.5 Hz, H^{24}), 1.55 (2H, quin, J = 7.5 Hz, H^{26}), 1.42 (9H, s, H^{22}), 1.26-1.19 (2H, m, H^{25}); ^{13}C NMR (101 MHz, $CDCl_3$) δ_C ppm 177.0 (C^{28}), 165.6 (C^{11}), 156.6 (C^{4}, 143.7 (C^{20}), 143.4 (C^{2}), 142.5 (C^{7}), 133.3 (C^{10}), 130.7 (C^{18}), 130.1 (C^{13}), 127.8 (C^{9}), 127.3 (C^{8}), 126.0 (C^{15}), 125.9 (C^{17}), 125.7 (C^{14}), 124.6 (C^{16}), 123.9 (C^{1}), 81.3 (C^{21}), 58.1 (C^{3}), 49.9 (C^{23}), 44.5 (C^{6}), 33.5 (C^{27}), 29.6 (C^{24}), 28.3 (C^{22}), 25.5 (C^{25}), 23.9 (C^{26}); R_f = 0.42 (10% MeOH:DCM).

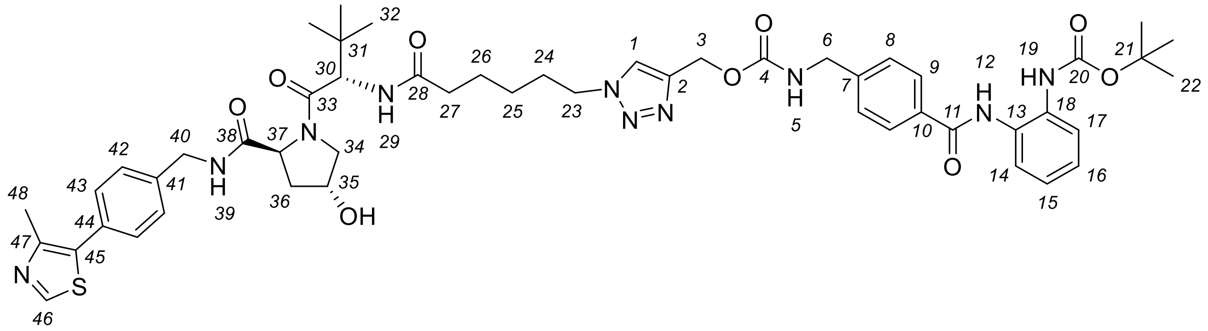

Tert-butyl (2-(4-(((((1-(6-(((S)-1-((2S,4R)-4-hydroxy-2-((4-(4-methylthiazol-5-yl)benzyl)carbamoyl) pyrrolidin-1-yl)-3,3-dimethyl-1-oxobutan-2-yl)amino)-6-oxohexyl)-1H-1,2,3-triazol-4-yl)methoxy) carbonyl)amino)methyl)benzamido)phenyl)carbamate (8a)

6-(4-((((4-((2-((Tert-butoxycarbonyl)amino)phenyl)carbamoyl)benzyl)carbamoyl)oxy)methyl)-1H-1,2,3-triazol-1-yl)hexanoic acid (0.054 g, 0.09 mmol) and HATU (0.04 g, 0.11 mmol) were charged to a flask, dissolved in anhydrous DMF (2 mL) under an inert atmosphere and the solution cooled to 0 °C. Diisopropylethylamine (0.04 mL, 0.25 mmol) was added and the reaction stirred for 15 mins. (4*R*)-3-Methyl-L-valyl-4-hydroxy-*N*-[[4-(4-methyl-5-thiazolyl)phenyl]methyl]-L-prolinamide hydrochloride (0.04 g, 0.08 mmol) was dissolved in anhydrous DMF (1 mL) and added, the reaction allowed to warm to room temperature and stirred for 16 h. Solvent was removed under reduced pressure and redissolved in EtOAc (10 mL), extracted with saturated $NaHCO_3$ solution (2 x 10 mL), brine (2 x 10 mL), organic layers combined, dried over $MgSO_4$, filtered and solvent removed under reduced pressure. The crude oil was purified by flash chromatography on silica (2-10% MeOH:DCM solvent gradient) to provide the *title compound* as an off-white crystalline solid (0.062 g, 67%). ^{1}H NMR (400 MHz, MeOD-d_4) δ_H ppm 8.75 (1H, s, H^{46}), 7.85 (1H, s, H^{1}), 7.80 (2H, d, J = 8.0 Hz, H^{9}), 7.48 (1H, dd, J = 8.0 Hz 3.0 Hz, H^{14}), 7.34-7.26 (7H, m, $H^{8, 16, 42, 43}$), 7.13-7.06 (2H, m, $H^{15, 17}$), 5.05 (2H, s, H^{3}), 4.50 (1H, s, H^{30}), 4.48-4.36 (3H, m, $H^{35, 37, 40}$), 4.28-4.20 (5H, m, $H^{6, 23, 40'}$), 3.79-3.76 (1H, m, H^{34}), 3.68-3.64 (1H, m, $H^{34'}$), 2.35 (3H, s, H^{48}), 2.17-2.07 (3H, m, $H^{27, 36}$), 1.99-1.93 (1H, m, $H^{36'}$), 1.81-1.75 (2H, m, H^{24}), 1.55-1.47 (2H, m, H^{26}), 1.37 (9H, s, H^{22}), 1.22-1.14 (2H, m, H^{25}), 0.90 (9H, s, H^{32}); ^{13}C NMR (101 MHz, MeOD-d_4) δ_C ppm 174.2 (C^{28}), 173.0 (C^{38}), 170.9 (C^{33}), 166.7 (C^{11}), 157.4 (C^{4}), 155.0 (C^{20}), 151.6 (C^{47}), 151.4 (C^{46}), 147.7 (C^{45}), 144.0 (C^{7}), 143.1 (C^{2}), 138.9 (C^{41}), 132.8 (C^{10}), 132.0 (C^{44}), 131.6 (C^{13}), 130.2 (C^{18}), 128.9 (C^{16}), 127.5 (C^{42}), 127.4 (C^{9}), 127.0 (C^{43}), 126.0 (C^{15}), 125.8 (C^{14}), 124.8 (C^{17}), 124.3 (C^{8}), 124.2 (C^{1}), 80.4 (C^{21}), 69.7 (C^{35}), 59.4 (C^{37}), 57.6 (C^{30}), 57.3 (C^{3}), 56.7 (C^{34}), 49.8 (C^{23}), 43.7 (C^{6}), 42.3 (C^{40}),

37.5 (C^{36}), 35.2 (C^{31}), 34.8 (C^{27}), 29.5 (C^{24}), 27.2 (C^{22}), 25.6 (C^{32}), 25.5 (C^{25}), 24.8 (C^{26}), 14.5 (C^{48}); *m/z* (HRMS$^+$) 1015.4434 $[M+Na]^+$ ($C_{51}H_{64}N_{10}O_9SNa$ requires 1015.4476); R_f = 0.25 (10% MeOH:DCM).

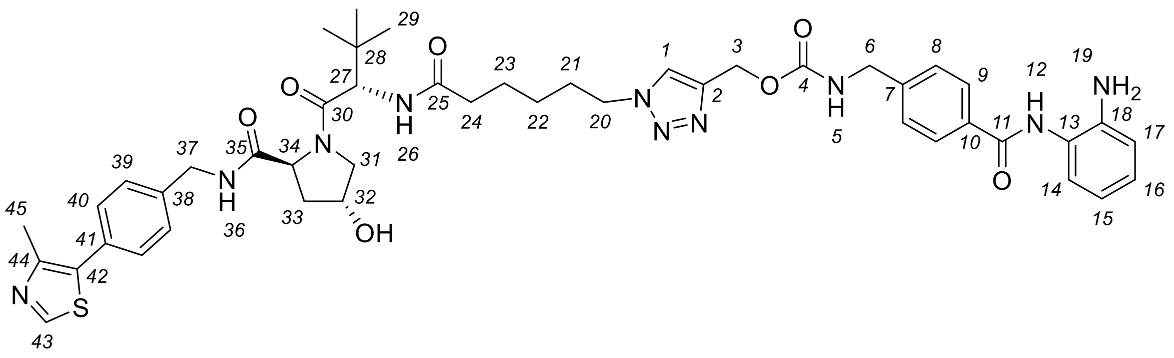

(1-(6-(((S)-1-((2S,4R)-4-Hydroxy-2-((4-(4-methylthiazol-5-yl)benzyl)carbamoyl)pyrrolidin-1-yl)-3,3-dimethyl-1-oxobutan-2-yl)amino)-6-oxohexyl)-1H-1,2,3-triazol-4-yl)methyl(4-((2-aminophenyl) carbamoyl)benzyl)carbamate (9)

TFA (0.2 mL) was added to a stirring solution of tert-butyl (2-(4-(((((1-(6-(((S)-1-((2S,4R)-4-hydroxy-2-((4-(4-methylthiazol-5-yl)benzyl)carbamoyl)pyrrolidin-1-yl)-3,3-dimethyl-1-oxobutan-2-yl)amino)-6-oxohexyl)-1H-1,2,3-triazol-4-yl)methoxy)carbonyl)amino)methyl)benzamido)phenyl) carbamate (0.06 g, 0.06 mmol) in DCM (3 mL) and the solution stirred at room temperature for 7 h. The reaction mixture was concentrated under reduced pressure and dissolved in MeOH (2 mL), agitated in MP-carbonate resin (3.02 mmol/g loading capacity, 200 mg) for 4 h. Prior to biological evaluation the product was further purified by semi-preparative HPLC (Method 1), the solution lyophilised to provide the *title compound* as an off-white solid (0.05 g, 70%). ^{1}H NMR (400 MHz, MeOD-d_4) δ_H ppm 8.75 (1H, s, H^{43}), 7.86 (1H, s, H^{1}), 7.82 (2H, d, J = 7.0 Hz, H^9), 7.35-7.27 (6H, m, $H^{8, 39, 40}$), 7.07 (1H, dd, J = 7.5 Hz 1.5 Hz, H^{14}), 6.96 (1H, ddd, J = 8.0 Hz 8.0 Hz 1.5 Hz, H^{16}), 6.79 (1H, dd, J = 8.0 Hz 1.5 Hz, H^{17}), 6.65 (1H, ddd, J = 8.0 Hz 8.0 Hz 1.5 Hz, H^{15}), 5.05 (2H, s, H^3), 4.50 (1H, s, H^{27}), 4.48-4.36 (3H, m, $H^{32, 34, 37}$), 4.29-4.21 (5H, m, $H^{6, 20, 37'}$), 3.79-3.76 (1H, m, H^{31}), 3.69-3.65 (1H, m, $H^{31'}$), 2.35 (3H, s, H^{45}), 2.16-2.06 (3H, m, $H^{24, 33}$), 1.99-1.92 (1H, m, $H^{33'}$), 1.81-1.74 (2H, m, H^{21}), 1.56-1.48 (2H, m, H^{23}), 1.22-1.16 (2H, m, H^{22}), 0.91 (9H, s, H^{29}); ^{13}C NMR (101 MHz, MeOD-d_4) δ_C ppm 174.3 (C^{25}), 173.1 (C^{35}), 171.1 (C^{30}), 167.3 (C^{11}), 157.5 (C^4), 151.4 (C^{43}), 147.6 (C^{44}), 143.3 (C^2), 143.2 (C^7), 142.4 (C^{18}), 138.7 (C^{38}), 132.9 (C^{10}), 132.1 (C^{42}), 132.0 (C^{41}), 128.9 (C^{39}), 127.6 (C^9), 127.5 (C^{40}),127.2 (C^{16}), 126.9 (C^8), 126.2 (C^{14}), 124.2 (C^1), 123.9 (C^{13}), 118.3 (C^{15}), 117.3 (C^{17}), 69.7 (C^{32}), 59.5 (C^{34}), 57.6 (C^{27}), 57.3 (C^3), 56.7 (C^{31}), 49.7 (C^{20}), 43.7 (C^6), 42.2 (C^{37}), 37.6 (C^{33}), 35.3 (C^{28}), 34.8 (C^{24}), 29.4 (C^{21}), 25.6 (C^{29}), 25.5 (C^{22}), 24.8 (C^{23}), 14.6 (C^{45}); *m/z* (HRMS$^+$) 893.4129 $[M+H]^+$ ($C_{46}H_{57}N_{10}O_7S$ requires 893.4132).

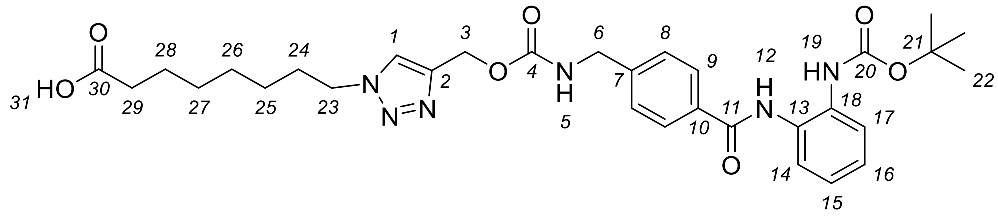

8-(4-((((4-((2-((Tert-butoxycarbonyl)amino)phenyl)carbamoyl)benzyl)carbamoyl)oxy)methyl)-1H-1,2,3-triazol-1-yl)octanoic acid (6b)

Tert-butyl-*N*-{2-[4-({[(prop-2-yn-1-yloxy)carbonyl]amino}methyl)benzamido]phenyl}carbamate (0.13 g, 0.29 mmol), 8-azidooctanoic acid (0.05 g, 0.29 mmol) and CuI (0.01 g, 0.10 mmol) were suspended in degassed DMF:MeCN (5 mL, 2:1) under an inert atmosphere. 2,6-Lutidine (0.06 g, 0.52 mmol) was added and the reaction stirred at room temperature for 16 h. The solvent was removed under reduced pressure and suspended in EtOAc (10 mL), washed with sat. $NaHCO_3$ (2 x 10 mL), brine (2 x 10 mL), the organic layers combined, dried over $MgSO_4$, filtered and the solvent removed under reduced pressure. The crude oil was purified by flash chromatography on silica (3-5% MeOH:DCM solvent gradient) to provide the *title compound* as an off white solid (0.08 g, 46%). ^{1}H NMR (400 MHz, $CDCl_3$) δ_H ppm 9.35 (1H, bs, H^{12}), 7.83 (2H, d, *J* = 8.0 Hz, H^{9}), 7.64-7.60 (2H, m, $H^{1, 14}$), 7.40 (1H, bs, H^{19}), 7.31-7.29 (1H, m, H^{16}), 7.26 (2H, d, *J* = 8.0 Hz, H^{8}), 7.11-7.09 (2H, m, $H^{15, 17}$), 5.85 (1H, bs, H^{5}), 5.16 (2H, s, H^{3}), 4.34 (2H, m, H^{6}), 4.27 (2H, m, H^{23}), 2.28-2.25 (2H, m, H^{29}), 1.85-1.82 (2H, m, H^{24}), 1.57-1.53 (2H, m, H^{28}), 1.46 (9H, s, H^{22}), 1.31-1.25 (6H, m, $H^{25, 26, 27}$); ^{13}C NMR (101 MHz, $CDCl_3$) δ_C ppm 177.8 (C^{30}), 165.8 (C^{11}), 156.6 (C^{4}), 154.7 (C^{20}), 143.1 (C^{2}), 142.7 (C^{7}), 133.0 (C^{10}), 130.5 (C^{13}), 130.4 (C^{18}), 127.7 (2C, C^{9}), 127.3 (C^{8}), 126.1 (C^{15}), 125.7 (C^{14}), 125.6 (C^{17}), 124.5 (C^{16}), 123.8 (C^{1}), 81.2 (C^{21}), 58.2 (C^{3}), 50.3 (C^{23}), 44.5 (C^{6}), 33.9 (C^{29}), 29.9 (C^{24}), 28.4 (C^{27}), 28.3 (C^{22}), 26.1 (C^{25}), 24.5 (C^{28}), 22.9 (C^{26}); *m/z* ($HRMS^{+}$) 631.2856 $[M+Na]^{+}$ ($C_{31}H_{40}N_6O_7Na$ requires 631.2856); R_f = 0.23 (7% MeOH:DCM).

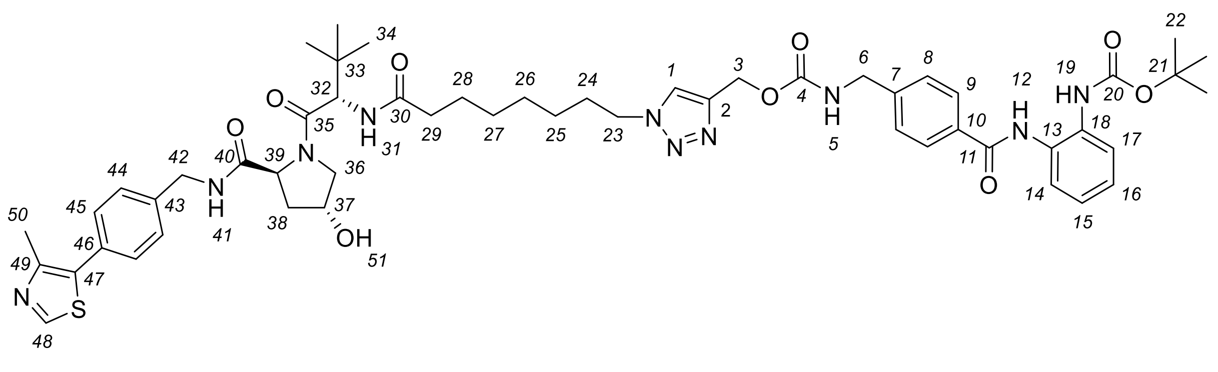

(1-(8-(((S)-1-((2S,4R)-4-Hydroxy-2-((4-(4-methylthiazol-5-yl)benzyl)carbamoyl)pyrrolidin-1-yl)-3,3-dimethyl-1-oxobutan-2-yl)amino)-8-oxooctyl)-1H-1,2,3-triazol-4-yl)methyl(4-((2-((tert-butoxy carbonyl)amino)phenyl)carbamoyl)benzyl)carbamate (8b)

8-(4-((((4-((2-((Tert-butoxycarbonyl)amino)phenyl)carbamoyl)benzyl)carbamoyl)oxy)methyl)-1H-1,2,3-triazol-1-yl)octanoic acid (0.075 g, 0.12 mmol) and HATU (0.05 g, 0.13 mmol) were charged to a flask, dissolved in anhydrous DMF (2 mL) under an inert atmosphere and the solution cooled to 0 °C. Diisopropylethylamine (0.05 mL, 0.29 mmol) was added and the reaction stirred for 15 mins. (4*R*)-3-Methyl-L-valyl-4-hydroxy-*N*-[[4-(4-methyl-5-thiazolyl)phenyl]methyl]-L-prolinamide hydrochloride (0.05 g, 0.10 mmol) was dissolved in anhydrous DMF (1 mL) and added, the reaction allowed to warm to room temperature and stirred for 16 h. Solvent was removed under reduced pressure and redissolved in EtOAc (10 mL), extracted with saturated $NaHCO_3$ solution (2 x 10 mL), brine (2 x 10 mL), organic layers combined, dried over $MgSO_4$, filtered and solvent removed under reduced pressure. The crude oil was purified by flash chromatography on silica (2-7% MeOH:DCM solvent gradient) to provide the *title compound* as an off-white crystalline solid (0.08 g, 78%). ^{1}H NMR (400 MHz, MeOD-d_4) δ_H ppm 8.86 (1H, s, H^{48}), 7.89 (1H, s, H^{1}), 7.81 (2H, d, J = 9.0 Hz, H^{9}), 7.67 (1H, d, J = 9.0 Hz, H^{31}), 7.51-7.48 (1H, m, H^{14}), 7.36-7.28 (7H, m, $H^{8, 17, 44, 45}$), 7.13-7.07 (2H, m, $H^{15, 16}$), 5.06 (2H, s, H^{3}), 4.53-4.51 (1H, m, H^{32}), 4.48-4.40 (2H, m, $H^{39, 42}$), 4.38-4.37 (1H, m, H^{37}), 4.30-4.22 (5H, m, $H^{6, 23, 42'}$), 3.80-3.77 (1H, m, H^{36}), 3.69-3.66 (1H, m, $H^{36'}$), 2.36 (3H, s, H^{50}), 2.16-2.07 (3H, m, $H^{29, 38}$), 1.99-1.93 (1H, m, $H^{38'}$), 1.77 (2H, m, H^{24}), 1.49-1.42 (2H, m, H^{28}), 1.37 (9H, s, H^{22}), 1.24-1.16 (6H, m, $H^{25, 26, 27}$), 0.92 (9H, s, H^{34}); ^{13}C NMR (101 MHz, MeOD-d_4) δ_C ppm 174.6 (C^{30}), 173.1 (C^{35}),170.9 (C^{40}), 166.6 (C^{11}), 157.2 (C^{4}), 154.9 (C^{20}), 151.4 (C^{48}), 147.6 (C^{49}), 143.8 (C^{10}), 143.1 (C^{2}), 138.8 (C^{7}), 132.7 (C^{43}), 132.0 (C^{47}), 131.6 (C^{13}), 130.2 (C^{18}), 130.1 (C^{46}), 128.9 (C^{45}), 127.5 (C^{8}), 127.4 (C^{9}), 127.0 (C^{44}), 126.0 (C^{15}), 125.7 (C^{14}), 124.8 (C^{16}), 124.3 (C^{1}), 124.1 (C^{17}), 80.3 (C^{21}), 69.6 (C^{37}), 59.4 (C^{39}), 57.5 (C^{32}), 57.3 (C^{3}), 56.6 (C^{36}), 49.9 (C^{23}), 43.7 (C^{6}), 42.3 (C^{42}), 37.5 (C^{38}), 35.1 (C^{29}), 29.7 (C^{24}), 29.3 (C^{33}), 28.5 (C^{25}), 28.2 (C^{27}), 27.3 (C^{22}), 25.8 (C^{26}), 25.6 (C^{34}), 25.3 (C^{28}), 14.4 (C^{50}); *m/z* ($HRMS^+$) 1043.4758 $[M+Na]^+$ ($C_{53}H_{68}N_{10}O_9SNa$ requires 1043.4789); R_f = 0.15 (7% MeOH:DCM).

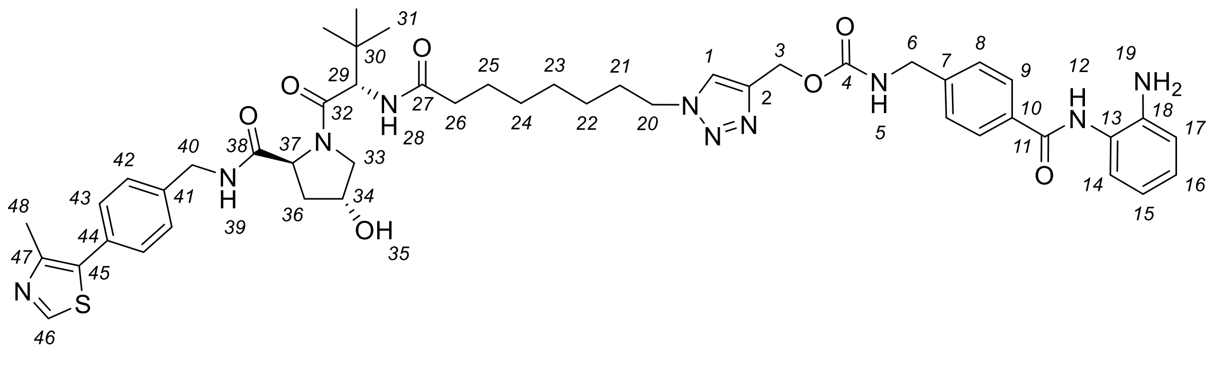

(1-(8-(((S)-1-((2S,4R)-4-Hydroxy-2-((4-(4-methylthiazol-5-yl)benzyl)carbamoyl)pyrrolidin-1-yl)-3,3-dimethyl-1-oxobutan-2-yl)amino)-8-oxooctyl)-1H-1,2,3-triazol-4-yl)methyl (4-((2-aminophenyl) carbamoyl)benzyl)carbamate (11)

TFA (0.2 mL) was added to a stirring solution of (1-(8-(((*S*)-1-((2*S*,4*R*)-4-hydroxy-2-((4-(4-methylthiazol-5-yl)benzyl)carbamoyl)pyrrolidin-1-yl)-3,3-dimethyl-1-oxobutan-2-yl)amino)-8-

oxooctyl)-1H-1,2,3-triazol-4-yl)methyl(4-((2-((tert-butoxycarbonyl)amino)phenyl)carbamoyl)benzyl) carbamate (0.08 g, 0.08 mmol) in DCM (3 mL) and the solution stirred at room temperature for 7 h. The reaction mixture was concentrated under reduced pressure and dissolved in MeOH (2 mL), agitated in MP-carbonate resin (3.02 mmol/g loading capacity, 210 mg) for 4 h. The filtrate was concentrated under reduced pressure to provide a yellow oil. The crude oil was dissolved in MeCN:H_2O (1:1, 2 mL) and lyophilised to remove residual TFA impurities to provide the *title compound* as an off-white solid (0.06 g, 84%). ^{1}H NMR (400 MHz, MeOD-d_4) δ_H ppm 8.75 (1H, s, H^{46}), 7.87 (1H, s, H^{1}), 7.82 (2H, m, H^{9}), 7.35-7.33 (2H, m, H^{43}), 7.31-7.27 (4H, m, $H^{8, 42}$), 7.08-7.06 (1H, m H^{14}), 6.98-6.94 (1H, m, H^{16}), 6.80-6.77 (1H, m, H^{17}), 6.67-6.63 (1H, m, H^{15}), 5.05 (2H, s, H^{3}), 4.51 (1H, s, H^{29}), 4.47-4.39 (2H, m, $H^{37, 40}$), 4.38-4.36 (1H, m, H^{34}), 4.28-4.19 (3H, m, $H^{20,40'}$), 4.25 (2H, s, H^{6}), 3.79-3.76 (1H, m, H^{33}), 3.69-3.65 (1H, m, $H^{33'}$), 2.35 (3H, s, H^{48}), 2.17-2.06 (3H, m, $H^{26,36}$), 1.99-1.92 (1H, m, $H^{36'}$), 1.80-1.73 (2H, m, H^{21}), 1.49-1.42 (2H, m, H^{25}), 1.23-1.14 (6H, m, $H^{22, 23, 24}$), 0.91 (9H, s, H^{31}); ^{13}C NMR (101 MHz, MeOD-d_4) δ_C ppm 174.6 (C^{27}), 173.0 (C^{38}), 170.9 (C^{32}), 167.2 (C^{11}), 157.3 (C^{4}), 151.5 (C^{45}), 147.6 (C^{46}), 145.0 (C^{47}), 143.4 (C^{7}), 143.1 (C^{2}), 142.3 (C^{13}), 138.9 (C^{44}), 132.9 (C^{40}), 130.1 (C^{43}), 129.1 (C^{10}), 128.9 (C^{42}), 128.3 (C^{15}), 127.6 (C^{8}), 127.5 (C^{9}), 127.1 (C^{44}), 126.2 (C^{14}), 124.2 (C^{1}), 123.9 (C^{18}), 117.4 (C^{17}), 69.6 (C^{34}), 59.4 (C^{37}), 57.5 (C^{29}), 57.3 (C^{3}), 56.6 (C^{33}), 49.9 (C^{20}), 43.7 (C^{6}), 42.3 (C^{40}), 37.6 (C^{36}), 35.2 (C^{30}), 35.1 (C^{26}), 29.7 (C^{21}), 28.5 (C^{24}), 28.2 (C^{23}), 25.6 (C^{31}), 25.8 (C^{22}), 25.3 (C^{25}), 14.5 (C^{48}); *m/z* ($HRMS^+$) 943.4246 $[M+Na]^+$ ($C_{48}H_{60}N_{10}O_7SNa$ requires 943.4265).

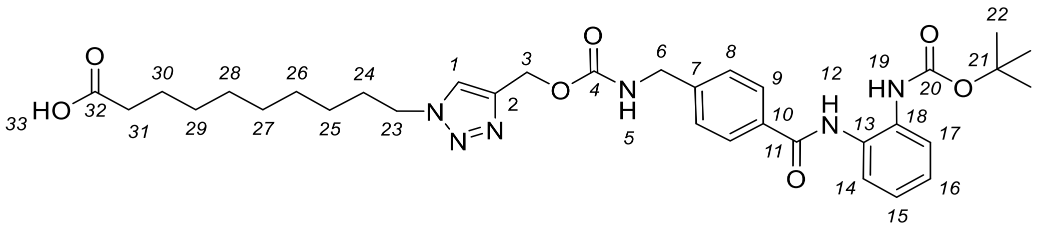

10-(4-((((4-((2-((Tert-butoxycarbonyl)amino)phenyl)carbamoyl)benzyl)carbamoyl)oxy)methyl)-1H-1,2,3-triazol-1-yl)decanoic acid (6c)

Tert-butyl-*N*-{2-[4-({[(prop-2-yn-1-yloxy)carbonyl]amino}methyl)benzamido]phenyl}carbamate (0.20 g, 0.47 mmol), 10-azidodecanoic acid (0.12 g, 0.56 mmol) and CuI (0.02 g, 0.14 mmol) were suspended in degassed DMF:MeCN (5 mL, 2:1) under an inert atmosphere. 2,6-Lutidine (0.08 mL, 0.71 mmol) was added and the reaction stirred at room temperature for 16 h. The solvent was removed under reduced pressure and suspended in EtOAc (10 mL), washed with sat. $NaHCO_3$ (2 x 10 mL), brine (2 x 10 mL), the organic layers combined, dried over $MgSO_4$, filtered and the solvent removed under reduced pressure. The crude oil was purified by flash chromatography on silica (4-7% MeOH:DCM solvent gradient) to provide the *title compound* as an off white solid (0.21 g, 69%). ^{1}H NMR (400 MHz, MeOD-d_4) δ_H ppm 7.98 (1H, s, H^{1}), 7.94 (2H, d, J = 8.0 Hz, H^{9}), 7.62 (1H, dd, J = 6.5 Hz 2.0 Hz, H^{14}), 7.46-7.43 (3H, m, $H^{8, 17}$), 7.27-7.21 (2H, m, $H^{15, 16}$), 5.19 (2H, s, H^{3}), 4.42-4.39 (4H, m, $H^{6, 23}$), 2.27 (2H, t, J = 7.5 Hz, H^{31}), 1.91 (2H, m, H^{24}), 1.59 (9H, s, H^{22}), 1.58 (2H, m, H^{30}), 1.36-1.27 (10H, m, H^{25-29});

^{13}C NMR (101 MHz, MeOD-d_4) δ_C ppm 176.3 (C^{32}), 166.7 (C^{11}), 157.2 (C^4), 154.9 (C^{20}), 143.8 (C^2), 132.8 (C^7), 131.6 (C^{10}), 130.3 (C^{13}), 130.2 (C^{18}), 127.4 (C^9), 126.9 (C^8), 126.0 (C^{15}), 125.7 (C^{14}), 124.8 (C^{16}), 124.2 (C^1), 124.1 (C^{17}), 80.3 (C^{21}), 57.3 (C^3), 49.9 (C^{23}), 43.7 (C^6), 33.5 (C^{31}), 29.8 (C^{24}), 28.9-28.5 (C^{26-29}), 27.2 (C^{22}), 25.9 (C^{25}), 24.6 (C^{30}); *m/z* (HRMS$^+$) 659.3163 $[M+Na]^+$ ($C_{33}H_{44}N_6O_7Na$ requires 659.3169); R_f = 0.16 (7% MeOH:DCM)

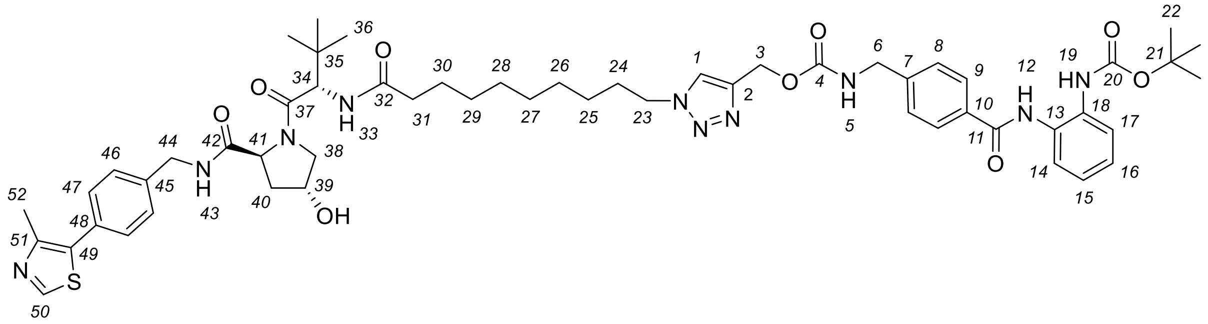

(1-(10-(((S)-1-((2S,4R)-4-Hydroxy-2-((4-(4-methylthiazol-5-yl)benzyl)carbamoyl)pyrrolidin-1-yl)-3,3-dimethyl-1-oxobutan-2-yl)amino)-10-oxodecyl)-1H-1,2,3-triazol-4-yl)methyl(4-((2-((tert-butoxy carbonyl)amino)phenyl)carbamoyl)benzyl)carbamate (8c)

10-(4-((((4-((2-((Tert-butoxycarbonyl)amino)phenyl)carbamoyl)benzyl)carbamoyl)oxy)methyl)-1H-1,2,3-triazol-1-yl)decanoic acid (0.07 g, 0.10 mmol) and HATU (0.05 g, 0.13 mmol) were charged to a flask, dissolved in anhydrous DMF (2 mL) under an inert atmosphere and the solution cooled to 0 °C. Diisopropylethylamine (0.05 mL, 0.29 mmol) was added and the reaction stirred for 15 mins. (4*R*)-3-Methyl-L-valyl-4-hydroxy-*N*-[[4-(4-methyl-5-thiazolyl)phenyl]methyl]-L-prolinamide hydrochloride (0.045 g, 0.09 mmol) was dissolved in anhydrous DMF (1 mL) and added, the reaction allowed to warm to room temperature and stirred for 16 h. Solvent was removed under reduced pressure and redissolved in EtOAc (10 mL), extracted with saturated $NaHCO_3$ solution (2 x 10 mL), brine (2 x 10 mL), organic layers combined, dried over $MgSO_4$, filtered and solvent removed under reduced pressure. The crude oil was purified by flash chromatography on silica (2-7% MeOH:DCM solvent gradient) to provide the *title compound* as a bright-yellow crystalline solid (0.07 g, 70%). ^1H NMR (400 MHz, MeOD-d_4) δ_H ppm 8.87 (1H, s, H^{50}), 7.97 (1H, s, H^1), 7.93 (2H, d, J = 9.0 Hz, H^9), 7.60 (1H, m, H^{14}), 7.45-7.38 (7H, m, $H^{8, 17, 46, 47}$), 7.24-7.17 (2H, m, $H^{15, 16}$), 5.17 (2H, s, H^3), 4.64 (1H, s, H^{34}), 4.59-4.52 (2H, m, $H^{41, 44}$), 4.50-4.49 (1H, m, H^{39}), 4.49-4.46 (3H, m, $H^{5, 23}$), 4.38-4.32 (1H, m, $H^{44'}$), 4.36 (2H, s, H^6), 3.90-3.87 (1H, m, H^{38}), 3.80-3.76 (1H, m, $H^{38'}$), 2.48 (3H, s, H^{52}), 2.29-2.16 (3H, m, $H^{31, 40}$), 2.10-2.03 (1H, m, $H^{40'}$), 1.90-1.83 (2H, m, H^{24}), 1.60-1.52 (2H, m, H^{30}), 1.47 (9H, s, H^{22}), 1.31-1.23 (10H, m, H^{25-29}), 1.02 (9H, s, H^{36}); ^{13}C NMR (101 MHz, MeOD-d_4) δ_C ppm 174.6 (C^{32}), 173.0 (C^{37}), 171.5 (C^{42}), 166.6 (C^{11}), 157.2 (C^4). 154.8 (C^{20}), 151.4 (C^{50}), 147.6 (C^{51}), 143.7 (C^{10}), 143.1 (C^2), 138.8 (C^7), 132.7 (C^{45}), 132.0 (C^{49}), 131.6 (C^{13}), 130.2 (C^{18}), 130.1 (C^{48}), 128.9 (C^{47}), 127.5 (C^8), 127.4 (C^9), 127.0 (C^{46}), 126.0 (C^{15}), 125.7 (C^{14}), 124.8 (C^{16}), 124.3 (C^1), 124.1 (C^{17}), 80.3 (C^{21}), 69.6 (C^{39}), 59.4 (C^{41}), 57.5 (C^{34}), 57.3 (C^3), 56.6 (C^{38}), 49.9 (C^{23}), 43.7 (C^6), 42.3 (C^{44}), 37.5 (C^{40}), 35.1 (C^{31}), 29.8 (C^{24}), 29.3 (C^{35}), 28.9-28.5 (C^{25-}

[29]), 27.2 (C^{22}), 25.6 (C^{36}), 25.5 (C^{30}), 14.4 (C^{52}); *m/z* ($HRMS^+$) 1049.5237 $[M+H]^+$ ($C_{55}H_{73}N_{10}O_9S$ requires 1049.5283); R_f = 0.21 (7% MeOH:EtOAc).

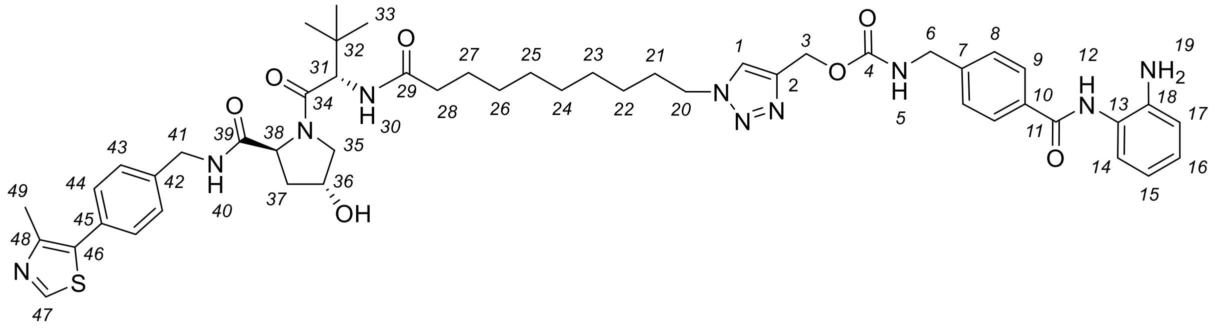

(1-(10-(((S)-1-((2S,4R)-4-Hydroxy-2-((4-(4-methylthiazol-5-yl)benzyl)carbamoyl)pyrrolidin-1-yl)-3,3-dimethyl-1-oxobutan-2-yl)amino)-10-oxodecyl)-1H-1,2,3-triazol-4-yl)methyl(4-((2-amino phenyl) carbamoyl)benzyl)carbamate (13)

TFA (0.2 mL) was added to a stirring solution of (1-(10-(((*S*)-1-((2*S*,4*R*)-4-hydroxy-2-((4-(4-methylthiazol-5-yl)benzyl)carbamoyl)pyrrolidin-1-yl)-3,3-dimethyl-1-oxobutan-2-yl)amino)-10-oxo decyl)-1H-1,2,3-triazol-4-yl)methyl(4-((2-((tert-butoxycarbonyl)amino)phenyl)carbamoyl)benzyl) carbamate (0.07 g, 0.07 mmol) in DCM (2 mL) and the resulting reaction mixture was stirred at room temperature overnight. The reaction was concentrated under reduced pressure to afford a yellow oil. The crude oil was dissolved in MeOH (2 mL), agitated in MP-carbonate resin (3.02 mmol/g loading capacity, 300 mg) for 3 h and filtered. Compound was lyophilised from H_2O:MeCN (1.5 mL 1:1) to provide the *title compound* as an off-white powder (0.05 g, 86%). ^{1}H NMR (400 MHz, MeOD-*d*$_4$) δ_H ppm 8.75 (1H, s, H^{47}), 7.85 (1H, s, H^1), 7.83 (2H, d, J = 8.0 Hz, H^9), 7.39-7.33 (2H, m, H^{44}), 7.32-7.27 (4H, m, $H^{8, 43}$), 7.09-7.07 (1H, m, H^{14}), 6.98-6.94 (1H, m, H^{16}), 6.80-6.78 (1H, m, H^{17}), 6.67-6.63 (1H, m, H^{15}), 5.06 (2H, s, H^3), 4.63 (1H, s, H^{31}), 4.59-4.47 (3H, m, $H^{36, 38, 41}$), 4.38-4.31 (5H, m, $H^{6, 20, 41'}$), 3.90-3.87 (1H, m, H^{35}), 3.80-3.76 (1H, m, $H^{35'}$), 2.46 (3H, s, H^{49}), 2.27-2.17 (3H, m, $H^{28, 37}$), 2.10-2.03 (1H, m, $H^{37'}$), 1.89-1.82 (2H, m, H^{21}), 1.60-1.52 (2H, m, H^{27}), 1.33-1.23 (10H, m, H^{22-26}), 1.03 (9H, s, H^{33}); ^{13}C NMR (101 MHz, MeOD-*d*$_4$) δ_C ppm 174.6 (C^{29}), 173.0 (C^{39}), 170.9 (C^{34}), 167.1 (C^{11}), 157.2 (C^4), 151.4 (C^{47}), 147.6 (C^{48}), 143.4 (C^{10}), 143.1 (C^2), 142.3 (C^{13}), 138.9 (C^{42}), 132.9 (C^7), 132.0 (C^{45}), 130.1 (C^{46}), 128.9 (C^{43}), 127.6 (C^9), 127.5 (C^{44}), 127.1 (C^{16}), 126.9 (C^8), 126.2 (C^{14}), 124.2 (C^1), 123.9 (C^{18}), 118.2 (C^{15}), 117.3 (C^{17}), 69.7 (C^{36}), 59.4 (C^{38}), 57.5 (C^{31}), 57.3 (C^3), 56.6 (C^{35}), 49.9 (C^{20}), 43.8 (C^6), 42.3 (C^{41}), 37.5 (C^{37}), 35.3 (C^{28}), 35.2 (C^{32}), 29.8 (C^{21}), 29.0-25.9 (C^{22-26}), 25.6 (C^{33}), 25.5 (C^{27}), 14.4 (C^{49}); *m/z* ($HRMS^+$) 971.4561 $[M+Na]^+$ ($C_{50}H_{64}N_{10}O_7SNa$ requires 971.4578).

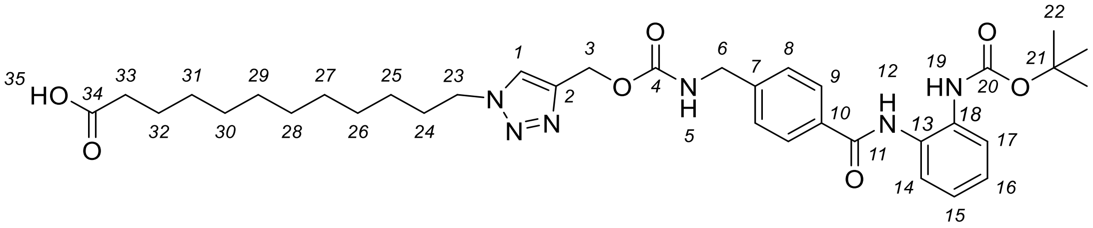

12-(4-((((4-((2-((Tert-butoxycarbonyl)amino)phenyl)carbamoyl)benzyl)carbamoyl)oxy)methyl)-1H-1,2,3-triazol-1-yl)dodecanoic acid (6d)

Tert-butyl-*N*-{2-[4-({[(prop-2-yn-1-yloxy)carbonyl]amino}methyl)benzamido]phenyl}carbamate (0.15 g, 0.34 mmol), 12-azidododecanoic acid (0.08 g, 0.34 mmol) and CuI (0.01 g, 0.10 mmol) were suspended in degassed DMF:MeCN (5 mL, 2:1) under an inert atmosphere. 2,6-Lutidine (0.06 g, 0.52 mmol) was added and the reaction stirred at room temperature for 16 h. The solvent was removed under reduced pressure and suspended in EtOAc (10 mL), washed with sat. $NaHCO_3$ (2 x 5 mL), brine (2 x 5 mL), the organic layers combined, dried over $MgSO_4$, filtered and the solvent removed under reduced pressure. The crude oil was purified by flash chromatography on silica (3:2 THF:Hexane) to provide the *title compound* as an off white solid (0.19 g, 81%). ^{1}H NMR (400 MHz, $CDCl_3$) δ_H ppm 9.21 (1H, s, H^{12}), 7.89 (2H, d, J = 8.0 Hz, H^9), 7.75 (1H, d, J = 7.0 Hz, H^{14}), 7.62 (1H, s, H^1), 7.32 (2H, J = 8.0 Hz, H^8), 7.26 (1H, d, J = 7.0 Hz, H^{17}), 7.22-7.12 (2H, m, $H^{15, 16}$), 7.01 (1H, s, H^{19}), 5.40 (1H, t, J = 6.5 Hz, H^5), 5.23 (2H, s, H^3), 4.40 (2H, d, J = 6.5 Hz, H^6), 4.33 (2H, t, J = 7.0 Hz, H^{23}), 2.32 (2H, t, J = 7.0 Hz, H^{33}), 1.88 (2H, tt, J = 7.0 Hz 7.0 Hz, H^{24}), 1.62 (2H, tt, J = 7.0 Hz 7.0 Hz, H^{32}), 1.50 (9H, s, H^{22}), 1.29-1.27 (8H, m, $H^{31, 30, 26, 25}$), 1.25-1.24 (6H, m, $H^{29, 28, 27}$); ^{13}C NMR (101 MHz, $CDCl_3$) δ_C ppm 178.2 (C^{34}), 165.3 (C^{11}), 156.3 (C^4), 154.7 (C^{20}), 143.2 (C^2), 143.1 (C^{10}), 142.5 (C^7), 130.8 (C^{18}), 130.0 (C^{13}), 127.7 (C^9), 127.4 (C^8), 125.9 ($C^{15, 16}$), 125.7 (C^{14}), 124.5 (C^{17}), 123.7 (C^1), 81.4 (C^{21}), 58.2 (C^3), 50.5 (C^{23}), 44.7 (C^6), 34.2 (C^{33}), 30.2 (C^{24}), 29.4-28.8 ($C^{31, 30, 29, 28, 27, 26, 25}$), 28.2 ($C^{22}$), 24.6 ($C^{32}$); *m/z* (HRMS$^+$) 665.3665 $[M+H]^+$ ($C_{35}H_{49}N_6O_7$ requires 665.3663); R_f = 0.28 (3:2 THF:Hexane).

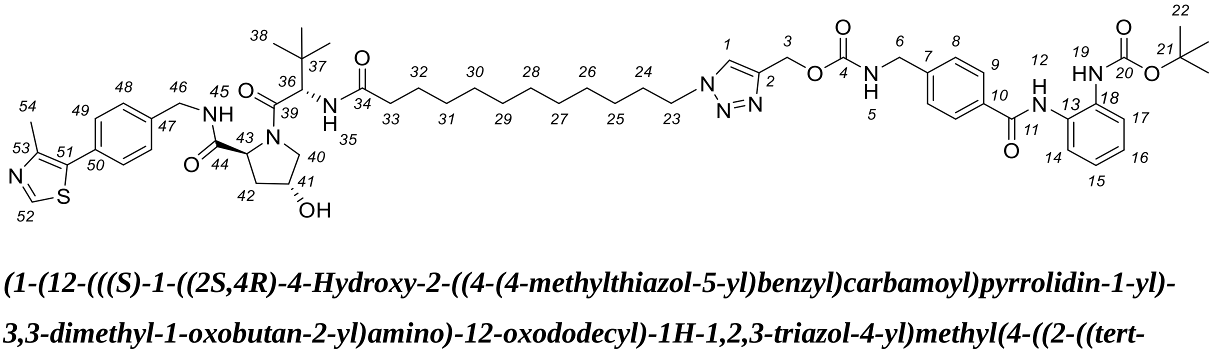

(1-(12-(((S)-1-((2S,4R)-4-Hydroxy-2-((4-(4-methylthiazol-5-yl)benzyl)carbamoyl)pyrrolidin-1-yl)-3,3-dimethyl-1-oxobutan-2-yl)amino)-12-oxododecyl)-1H-1,2,3-triazol-4-yl)methyl(4-((2-((tert-butoxy carbonyl)amino)phenyl)carbamoyl)benzyl)carbamate (8d)

12-(4-((((4-((2-((Tert-butoxycarbonyl)amino)phenyl)carbamoyl)benzyl)carbamoyl)oxy)methyl)-1H-1,2,3-triazol-1-yl)dodecanoic acid (0.08 g, 0.12 mmol), HATU (0.05 g, 0.13 mmol) and diisopropylethylamine (0.05 mL, 0.30 mmol) were dissolved in anhydrous DMF (1 mL), the reaction

mixture cooled to 0 °C and stirred for 15 mins. A solution of (4*R*)-3-methyl-L-valyl-4-hydroxy-*N*-[[4-(4-methyl-5-thiazolyl)phenyl]methyl]-L-prolinamide hydrochloride (0.05 g, 0.10 mmol) in anhydrous DMF (1 mL) was added slowly and the resulting solution was stirred at room temperature for 16 h. The solvent was removed under reduced pressure, redissolved in EtOAc (10 mL), washed with sat. $NaHCO_3$ (2 x 10 mL), brine (1 x 10 mL), organic layers combined, dried over $MgSO_4$, filtered and solvent removed under reduced pressure. The crude oil was purified using flash chromatography on silica (3-7% MeOH:DCM solvent gradient) to provide the *title compound* as a pale yellow oil (0.05 g, 38%). ^{1}H NMR (400 MHz, MeOD-d_4) δ_H ppm 8.85 (1H, s, H^{52}), 7.95 (1H, s, H^{1}), 7.92-7.90 (2H, d, J = 9.0 Hz, H^{9}), 7.61-7.59 (1H, m, H^{14}), 7.45-7.38 (7H, m, $H^{8, 17, 48, 49}$), 7.23-7.17 (2H, m, $H^{15, 16}$), 5.17 (2H, s, H^{3}), 4.62 (1H, s, H^{36}), 4.59-4.47 (3H, m, $H^{41, 43, 46}$), 4.38-4.31 (5H, m, $H^{6, 23, 46'}$), 3.90-3.88 (1H, m, H^{40}), 3.80-3.76 (1H, m, $H^{40'}$), 2.46 (3H, s, H^{54}), 2.31-2.17 (3H, m, $H^{33, 42}$), 2.10-2.04 (1H, m, $H^{42'}$), 1.89-1.83 (2H, m, H^{24}), 1.61-1.54 (2H, m, H^{32}), 1.48 (9H, s, H^{22}), 1.32-1.24 (14H, m, $H^{25, 26, 27, 28, 29, 30, 31}$), 1.02 (9H, s, H^{38}); ^{13}C NMR (101 MHz, MeOD-d_4) δ_C ppm 174.6 (C^{34}), 173.0 (C^{39}), 170.9 (C^{44}), 166.5 (C^{11}), 157.2 (C^{4}), 154.8 (C^{20}), 151.4 (C^{52}), 147.6 (C^{53}), 143.7 (C^{10}), 143.1 (C^{2}), 138.8 (C^{7}), 132.7 (C^{47}), 132.0 (C^{51}), 131.5 (C^{13}), 130.2 (C^{18}), 130.1 (C^{50}), 129.0 (C^{49}), 127.5 (C^{8}), 127.4 (C^{9}), 127.0 (C^{48}), 126.0 (C^{15}), 125.7 (C^{14}), 124.8 (C^{16}), 124.2 (C^{1}), 124.1 (C^{17}), 80.5 (C^{21}), 69.7 (C^{41}), 59.5 (C^{43}), 57.5 (C^{36}), 57.3 (C^{3}), 56.8 (C^{40}), 49.9 (C^{23}), 43.7 (C^{6}), 42.3 (C^{46}), 37.5 (C^{42}), 35.2 (C^{33}), 29.8 (C^{24}), 29.4 (C^{37}), 29.0-22.6 ($C^{25, 26, 27, 28, 29, 30, 31}$), 27.3 ($C^{22}$), 25.6 ($C^{38}$), 25.3 ($C^{32}$), 14.7 ($C^{54}$); *m/z* ($HRMS^+$) 1077.5618 $[M+H]^+$ ($C_{57}H_{77}N_{10}O_9S$ requires 1077.5596); R_f = 0.16 (7% MeOH:DCM).

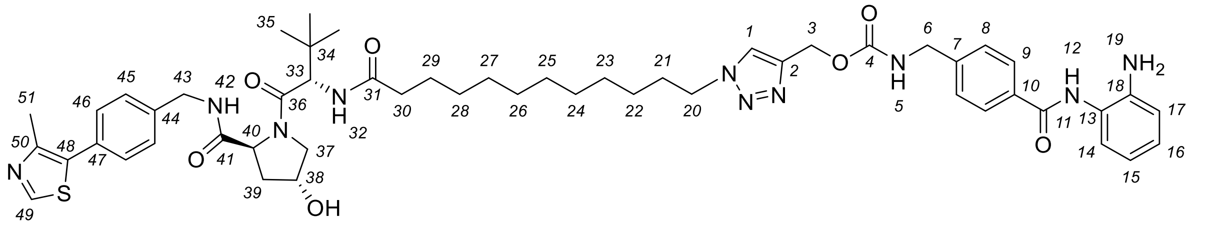

(1-(12-(((S)-1-((2S,4R)-4-Hydroxy-2-((4-(4-methylthiazol-5-yl)benzyl)carbamoyl)pyrrolidin-1-yl)-3,3-dimethyl-1-oxobutan-2-yl)amino)-12-oxododecyl)-1H-1,2,3-triazol-4-yl)methyl(4-((2-amino phenyl)carbamoyl)benzyl)carbamate (15)

TFA (0.2 mL) was added to a stirring solution of (1-(12-(((*S*)-1-((2*R*,4*R*)-4-hydroxy-2-((4-(4-methylthiazol-5-yl)benzyl)carbamoyl)pyrrolidin-1-yl)-3,3-dimethyl-1-oxobutan-2-yl)amino)-12-oxo dodecyl)-1H-1,2,3-triazol-4-yl)methyl(4-((2-((tert-butoxycarbonyl)amino)phenyl)carbamoyl)benzyl) carbamate (0.05 g, 0.05 mmol) in DCM (2 mL) and the resulting reaction mixture was stirred at room temperature overnight. The reaction was concentrated under reduced pressure to afford a yellow oil. The crude oil was dissolved in MeOH (2 mL), agitated in MP-carbonate resin (3.02 mmol/g loading capacity, 175 mg) for 3 h and filtered. Prior to biological evaluation the product was further purified by semi-preparative HPLC (Method 3). The product was lyophilised from H_2O:MeCN (1.5 mL, 1:1) to afford the *title compound* as an off white solid (0.03 g, 50%). ^{1}H NMR (400 MHz, MeOD-d_4) δ_H ppm

8.76 (1H, s, H^{49}), 7.86 (1H, s, H^{1}), 7.83 (2H, m, H^{9}), 7.36-7.29 (6H, m, $H^{8, 45, 46}$), 4.07-7.06 (1H, m, H^{14}), 6.98-6.94 (1H, m, H^{16}), 6.80-6.78 (1H, m, H^{17}), 6.68-6.63 (1H, m, H^{15}), 5.07 (2H, s, H^{3}), 4.52 (1H, s, H^{33}), 4.48-4.22 (8H, m, $H^{6, 20, 38, 40, 43}$), 3.80-3.77 (1H, m, H^{37}), 3.70-3.66 (1H, m, $H^{37'}$), 2.36 (3H, s, H^{51}), 2.25-2.07 (3H, m, $H^{30, 39}$), 2.00-1.93 (1H, m, $H^{39'}$), 1.80-1.73 (2H, m, H^{21}), 1.53-1.43 (2H, m, H^{29}), 1.24-1.13 (14H, m, $H^{22, 23, 24, 25, 26, 27, 28}$), 0.92 (9H, s, H^{35}); ^{13}C NMR (101 MHz, MeOD-d_4) δ_C ppm 174.6 (C^{31}), 173.0 (C^{41}), 170.9 (C^{36}), 167.1 (C^{11}), 157.2 (C^{4}), 151.4 (C^{48}), 147.6 (C^{49}), 143.4 (C^{7}), 143.1 (C^{2}), 142.3 (C^{13}), 138.8 (C^{47}), 132.9 (C^{43}), 130.1 (C^{46}), 129.0 (C^{10}), 128.9 (C^{45}), 127.9 (C^{15}), 127.6 (C^{9}), 127.5 (C^{44}), 127.3 (C^{17}), 127.1 (C^{16}), 126.9 (C^{8}), 126.2 (C^{14}), 124.1 (C^{1}), 123.9 (C^{18}), 118.2 (C^{50}), 69.6 (C^{38}), 59.4 (C^{40}), 57.5 (C^{33}), 57.3 (C^{3}), 56.6 (C^{37}), 49.9 (C^{20}), 43.7 (C^{6}), 37.5 (C^{39}), 35.3 (C^{30}), 35.2 (C^{34}), 29.8 (C^{21}), 29.0-25.4 ($C^{22, 23, 24, 25, 26, 27, 28}$), 25.6 ($C^{35}$), 25.5 ($C^{29}$), 14.4 ($C^{51}$); *m/z* (HRMS$^+$) 977.5051 $[M+H]^+$ ($C_{52}H_{69}N_{10}O_7S$ requires 977.5071); R_f = 0.16 (7% MeOH:DCM).

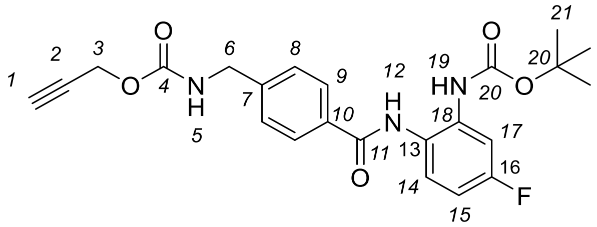

Prop-2-yn-1-yl(4-((2-((tert-butoxycarbonyl)amino)-4-fluorophenyl)carbamoyl)benzyl)carbamate (4a')

4-({[(Prop-2-yn-1-yloxy)carbonyl]amino}methyl)benzoic acid (0.36 g, 1.54 mmol) and HATU (0.88 g, 2.32 mmol) were dissolved in anhydrous DMF (5 mL) and stirred at room temperature for 1 h. Tert-butyl (2-amino-5-fluorophenyl)carbamate (0.35 g, 1.54 mmol), and diisopropylethylamine (0.53 mL, 3.09 mmol) were added and the reaction stirred at room temperature for 16 h. The solvent was removed under reduced pressure and suspended in EtOAc (20 mL), washed with sat. $NaHCO_3$ (2 x 20 mL), brine (20 mL), the organic layers combined, dried over $MgSO_4$, filtered and the solvent removed under reduced pressure. The crude oil was purified by flash chromatography on silica (Hexane:EtOAc 70:30-40:60 gradient) to provide the *title compound* as a blueish-grey oil (0.20 g, 23%). ^{1}H NMR (400 MHz, $CDCl_3$) δ_H ppm 8.89 (1H, s, H^{12}), 7.89 (2H, d, J = 8.0 Hz, H^{9}), 7.54 (1H, dd, J = 8.5 Hz, $^3J_{HF}$ 8.5 Hz, H^{15}), 7.36 (2H, d, J = 8.0 Hz, H^{8}), 7.16 (1H, dd, $^3J_{HF}$ 9.0 Hz, J = 2.0 Hz, H^{17}), 7.03 (1H, s, H^{19}), 6.86 (1H, ddd, J = 8.5 Hz 1.0 Hz, $^4J_{HF}$ 3.0 Hz, H^{14}), 5.30 (1H, t, J = 6.0 Hz, H^{5}), 4.73 (2H, d, J = 2.5 Hz, H^{3}), 4.44 (2H, d, J = 6.0 Hz, H^{6}), 2.49 (1H, t, J = 2.5 Hz, H^{1}), 1.50 (9H, s, H^{21}); ^{13}C NMR (101 MHz, $CDCl_3$) δ_C ppm 165.6 (C^{11}), 160.6 ($^1J_{CF}$ 246 Hz, C^{16}), 155.6 (C^{4}), 153.8 (C^{20}), 142.4 (C^{7}), 133.1 (C^{10}), 132.5 (C^{13}), 132.4 (C^{18}), 127.8 (C^{9}), 127.5 (C^{8}), 127.4 ($^2J_{CF}$ 10 Hz, C^{15}), 112.1 ($^2J_{CF}$ 22 Hz, H^{17}), 110.8 ($^2J_{CF}$ 26 Hz, C^{14}), 81.7 (C^{21}), 78.0 (C^{2}), 74.8 (C^{1}), 52.8 (C^{3}), 44.7 (C^{6}), 28.2 (C^{21}); ^{19}F NMR (376 MHz, $CDCl_3$) δ_F ppm -114.65 (1F, F^{16}); *m/z* (HRMS$^+$) 464.1584 $[M+Na]^+$ ($C_{23}H_{24}FN_6O_7Na$ requires 464.1598); R_f = 0.22 (60:40 Hexane:EtOAc).

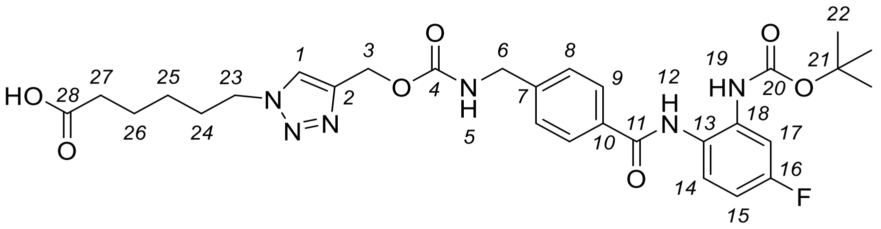

6-(4-((((4-((2-((Tert-butoxycarbonyl)amino)-4-fluorophenyl)carbamoyl)benzyl)carbamoyl)oxy) methyl) -1H-1,2,3-triazol-1-yl)hexanoic acid (6a')

Prop-2-yn-1-yl (4-((2-((tert-butoxycarbonyl)amino)-4-fluorophenyl)carbamoyl)benzyl) carbamate (0.12 g, 0.28 mmol), 6-azidohexanoic acid (0.04 g, 0.28 mmol) and CuI (0.01 g, 0.08 mmol) were suspended in degassed DMF:MeCN (5 mL, 2:1) under an inert atmosphere. 2,6-Lutidine (0.05 mL, 0.42 mmol) was added and the reaction stirred at room temperature for 16 h. The solvent was removed under reduced pressure and suspended in EtOAc (10 mL), washed with sat. $NaHCO_3$ (2 x 5 mL), brine (2 x 5 mL), the organic layers combined, dried over $MgSO_4$, filtered and the solvent removed under reduced pressure. The crude oil was purified by flash chromatography on silica (5-10%MeOH:DCM gradient) to provide the *title compound* as an off white solid (0.09 g, 55%). ^{1}H NMR (400 MHz, $CDCl_3$) δ_H ppm 8.82 (1H, bs, H^{12}), 7.80 (2H, d, J = 7.0 Hz, H^9), 7.57 (1H, s, H^1), 7.45 (1H, m, H^{14}), 7.23 (2H, d, J = 7.0 Hz, H^8), 7.14 (1H, dd, J = 2.8 Hz, $^3J_{HF}$ 10.0 Hz, H^{17}), 6.77 (1H, ddd, J = 8.0 Hz 3.5 Hz, $^3J_{HF}$ 9.0 Hz, H^{15}), 5.44 (1H, t, J = 6.5 Hz, H^5), 5.14 (2H, s, H^3), 4.35 (2H, d, J = 6.5 Hz, H^6), 4.28 (2H, t, J = 7.0 Hz, H^{23}), 2.22 (2H, t, J = 7.0 Hz, H^{27}), 1.83 (2H, quin, J = 7.0 Hz, H^{24}), 1.55 (2H, quin, J = 7.0 Hz, H^{26}), 1.42 (9H, s, H^{22}), 1.23 (2H, m, H^{25}); ^{13}C NMR (101 MHz, $CDCl_3$) δ_C ppm 176.2 (C^{28}), 164.9 (C^{11}), 164.4 (C^{20}), 160.6 (d, $^1J_{CF}$ 240 Hz, C^{16}), 156.5 (C^4), 143.3 (C^2), 142.7 (C^7), 133.0 (C^{10}), 127.8 (C^9), 127.3 (d, $^3J_{CF}$ 10 Hz, C^{14}) 127.2 (C^8), 125.3 (d, $^4J_{CF}$ 8.0 Hz, C^{13}), 125.1 (d, $^3J_{CF}$ 9.0 Hz, C^{18}), 124.0 (C^1), 111.9 (d, $^2J_{CF}$ 30 Hz, C^{15}), 110.7 (d, $^2J_{CF}$ 27 Hz, C^{17}), 81.8 (C^{21}), 58.0 (C^3), 50.0 (C^{23}), 44.5 (C^6), 33.4 (C^{27}), 29.8 (C^{24}), 28.2 (C^{22}), 25.5 (C^{25}), 23.8 (C^{26}); ^{19}F NMR (376 MHz, $CDCl_3$) δ_F ppm -114.5; *m/z* ($HRMS^+$) 621.2449 $[M+Na]^+$ ($C_{29}H_{35}FN_6O_7Na$ requires 621.2449); R_f = 0.44 (10% MeOH:DCM).

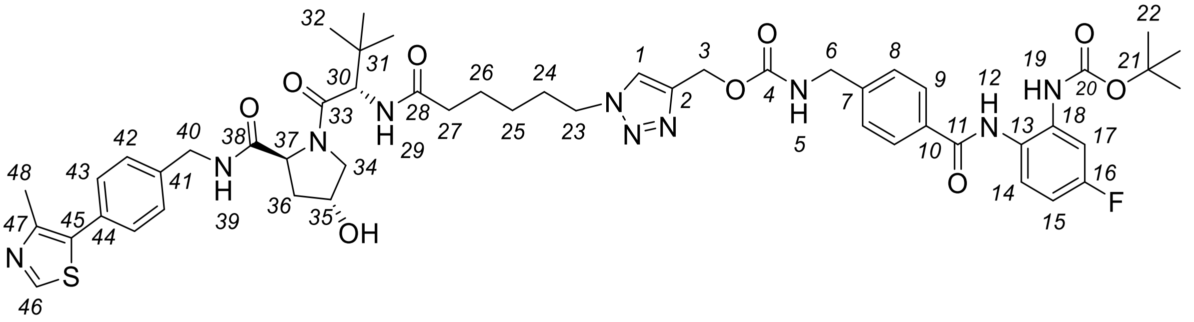

(1-(6-(((S)-1-((2S,4R)-4-Hydroxy-2-((4-(4-methylthiazol-5-yl)benzyl)carbamoyl)pyrrolidin-1-yl)-3,3-dimethyl-1-oxobutan-2-yl)amino)-6-oxohexyl)-1H-1,2,3-triazol-4-yl)methyl(4-((2-((tert-butoxy carbonyl)amino)-4-fluorophenyl)carbamoyl)benzyl)carbamate (8a')

6-(4-((((4-((2-((Tert-butoxycarbonyl)amino)-4-fluorophenyl)carbamoyl)benzyl)carbamoyl)oxy) methyl)-1H-1,2,3-triazol-1-yl)hexanoic acid (0.075 g, 0.12 mmol) and HATU (0.05 g, 0.13 mmol) were charged to a flask, dissolved in anhydrous DMF (2 mL) under an inert atmosphere and the solution cooled to 0 °C. Diisopropylethylamine (0.05 mL, 0.29 mmol) was added and the reaction stirred for 15 mins. (4*R*)-3-Methyl-L-valyl-4-hydroxy-*N*-[[4-(4-methyl-5-thiazolyl)phenyl]methyl]-L-prolinamide hydrochloride (0.05 g, 0.10 mmol) was dissolved in anhydrous DMF (1 mL) and added, the reaction allowed to warm to room temperature and stirred for 16 h. Solvent was removed under reduced pressure and redissolved in EtOAc (10 mL), extracted with saturated $NaHCO_3$ solution (2 x 10 mL), brine (2 x 10 mL), organic layers combined, dried over $MgSO_4$, filtered and solvent removed under reduced pressure. The crude oil was purified by flash chromatography on silica (2-8% MeOH:DCM solvent gradient) to provide the *title compound* as an off-white crystalline solid (0.06 g, 44%). ^{1}H NMR (400 MHz, MeOD-d_4) δ_H ppm 8.75 (1H, s, H^{46}), 7.86 (1H, s, H^{1}), 7.80 (2H, d, J = 8.5 Hz, H^{9}), 7.37-7.27 (8H, m, $H^{8, 14, 17, 42, 43}$), 6.79 (1H, ddd, J = 8.5 Hz 3.0 Hz, $^3J_{HF}$ 9.0 Hz, H^{15}), 5.05 (2H, s, H^{3}), 4.50 (1H, s, H^{30}), 4.48-4.36 (3H, m, $H^{35, 37, 40}$), 4.29-4.20 (5H, m, $H^{6, 23, 40'}$), 3.78-3.76 (1H, m, H^{34}), 3.69-3.65 (1H, m, $H^{34'}$), 2.35 (3H, s, H^{48}), 2.17-2.07 (3H, m, $H^{27, 36}$), 1.99-1.93 (1H, m, $H^{36'}$), 1.81-1.74 (2H, m, H^{24}), 1.56-1.48 (2H, m, H^{26}), 1.37 (9H, s, H^{22}), 1.22-1.15 (2H, m, H^{25}), 0.90 (9H, s, H^{32}); ^{13}C NMR (101 MHz, MeOD-d_4) δ_C ppm 174.2 (C^{28}), 173.0 (C^{38}), 171.6 (C^{33}), 167.1 (C^{11}), 160.8 (d, $^1J_{CF}$ 242 Hz, C^{16}), 157.5 (C^{4}), 151.5 (C^{46}), 150.4 (C^{20}), 147.8 (C^{47}), 143.8 (C^{7}), 143.2 (C^{2}), 138.9 (C^{41}), 134.2 (d, $^3J_{CF}$ 11 Hz, C^{18}), 132.9 (C^{10}), 132.5 (C^{45}), 132.0 (C^{44}), 128.9 (C^{43}), 127.8 (d, $^3J_{CF}$ 11 Hz, C^{14}), 127.6 (C^{42}), 127.5 (C^{9}), 127.0 (C^{8}), 125.0 (d, $^4J_{CF}$ 3.0 Hz, C^{13}), 124.3 (C^{1}), 110.6 (d, $^2J_{CF}$ 2 Hz, C^{17}), 109.5 (d, $^2J_{CF}$ 27 Hz, C^{15}), 80.7 (C^{21}), 69.7 (C^{35}), 59.4 (C^{37}), 57.6 (C^{30}), 57.3 (C^{3}), 56.6 (C^{34}), 49.8 (C^{23}), 43.8 (C^{6}), 42.2 (C^{40}), 37.5 (C^{36}), 35.2 (C^{31}), 34.9 (C^{27}), 29.5 (C^{24}), 27.2 (C^{22}), 25.7 (C^{32}), 25.6 (C^{25}), 24.8 (C^{26}), 14.7 (C^{48}); ^{19}F NMR (376 MHz, MeOD-d_4) δ_F ppm -116.4 (1F, F^{16}); *m/z* ($HRMS^+$) 1033.4385 $[M+Na]^+$ ($C_{51}H_{63}FN_{10}O_9SNa$ requires 1033.4382); R_f = 0.09 (8% MeOH:DCM).

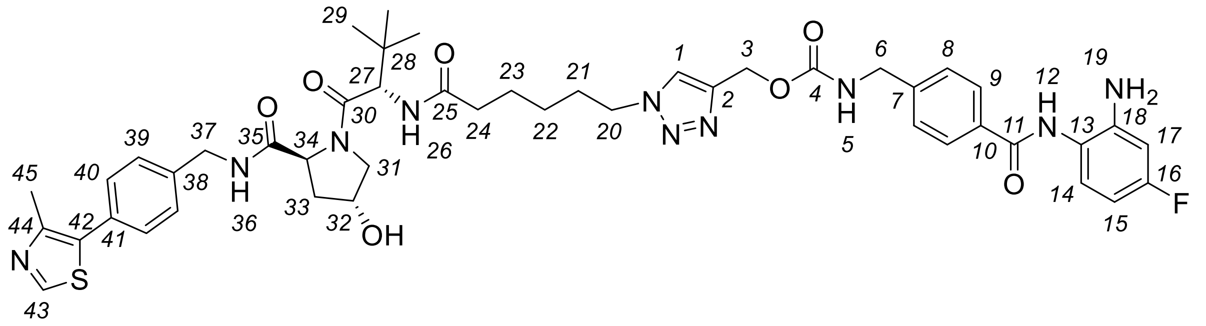

(1-(6-(((S)-1-((2S,4R)-4-Hydroxy-2-((4-(4-methylthiazol-5-yl)benzyl)carbamoyl)pyrrolidin-1-yl)-3,3-dimethyl-1-oxobutan-2-yl)amino)-6-oxohexyl)-1H-1,2,3-triazol-4-yl)methyl(4-((2-amino-4-fluorophenyl)carbamoyl)benzyl)carbamate (10)

TFA (0.2 mL) was added to a stirring solution of (1-(6-(((*S*)-1-((2*S*,4*R*)-4-hydroxy-2-((4-(4-methylthiazol-5-yl)benzyl)carbamoyl)pyrrolidin-1-yl)-3,3-dimethyl-1-oxobutan-2-yl)amino)-6-oxo

hexyl)-1*H*-1,2,3-triazol-4-yl)methyl(4-((2-((tert-butoxycarbonyl)amino)-4-fluorophenyl)carbamoyl) benzyl)carbamate (0.06 g, 0.06 mmol) in DCM (2 mL) and the resulting reaction mixture was stirred at room temperature overnight. The reaction was concentrated under reduced pressure to afford a yellow oil. The crude oil was dissolved in MeOH (2 mL), agitated in MP-carbonate resin (3.02 mmol/g loading capacity, 275 mg) for 3 h, filtered and solvent removed *en vacuo*. Prior to biological evaluation the product was further purified by semi-preparative HPLC (Method 1), the product lyophilised from solution to afford the *title compound* as an off white powder (0.04 g, 87%). ^{1}H NMR (400 MHz, MeOD-d_4) δ_H ppm 8.75 (1H, s, H^{43}), 7.86 (1H, s, H^{1}), 7.81 (2H, d, J = 7.5 Hz, H^{9}), 7.34-7.26 (6H, m, $H^{8, 39, 40}$), 7.00 (1H, dd, J = 8.5 Hz, $^4J_{HF}$ 6.0 Hz, H^{14}), 6.47 (1H, dd, J = 2.5 Hz, $^3J_{HF}$ 11 Hz, H^{17}), 6.30 (1H, ddd, J = 8.5 Hz 2.5 Hz, $^3J_{HF}$ 8.0 Hz, H^{15}), 5.04 (2H, s, H^{3}), 4.50 (1H, s, H^{27}), 4.49-4.36 (3H, m, $H^{32, 34, 37}$), 4.28-4.20 (5H, m, $H^{6, 20, 37'}$), 3.80-3.76 (1H, m, H^{31}), 3.69-3.64 (1H, m, $H^{31'}$), 2.35 (3H, s, H^{45}), 2.16-2.07 (3H, m, $H^{24, 33}$), 2.00-1.92 (1H, m, $H^{33'}$), 1.80-1.74 (2H, m, H^{21}), 1.54-1.48 (2H, m, H^{23}), 1.21-1.15 (2H, m, H^{22}), 0.91 (9H, s, H^{29}); ^{13}C NMR (101 MHz, MeOD-d_4) δ_C ppm 174.2 (C^{25}), 173.0 (C^{35}), 170.9 (C^{30}), 167.4 (C^{11}), 163.4 (d, $^1J_{CF}$ 240 Hz, C^{16}), 157.2 (C^{4}), 151.4 (C^{43}), 147.6 (C^{44}), 145.1 (d, $^3J_{CF}$ 12 Hz, H^{18}), 143.4 (C^{7}), 143.1 (C^{2}), 138.8 (C^{38}), 132.8 (C^{10}), 131.9 (C^{42}), 130.1 (C^{41}), 128.9 (C^{40}), 128.2 (C^{14}), 127.6 (C^{39}), 127.5 (C^{9}), 126.9 (C^{8}), 124.3 (C^{1}), 119.2 (d, $^4J_{CF}$ 2.5 Hz, C^{13}), 103.6 (d, $^3J_{CF}$ 23 Hz, C^{15}), 102.5 (d, $^3J_{CF}$ 20 Hz, C^{17}), 69.7 (C^{32}), 59.4 (C^{34}), 57.6 (C^{27}), 57.3 (C^{3}), 56.7 (C^{31}), 49.9 (C^{20}), 43.8 (C^{6}), 42.3 (C^{37}), 37.5 (C^{33}), 35.1 (C^{28}), 34.7 (C^{24}), 29.5 (C^{21}), 25.6 (C^{29}), 25.5 (C^{22}), 24.8 (C^{23}), 14.5 (C^{45}); ^{19}F NMR (376 MHz, MeOD-d_4) δ_F ppm -117.4 (1F, F^{16}); *m/z* (HRMS$^+$) 911.4038 [M+H]$^+$ ($C_{46}H_{56}FN_{10}O_7S$ requires 911.4038).

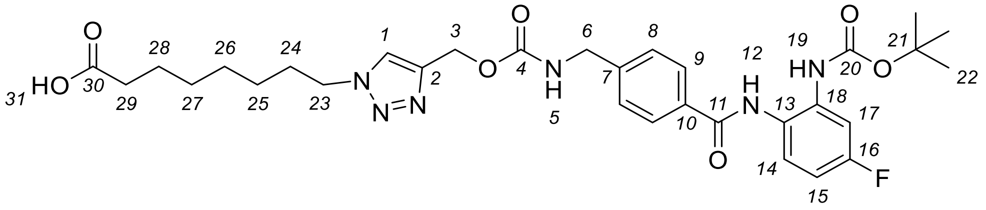

8-(4-((((4-((2-((Tert-butoxycarbonyl)amino)-4-fluorophenyl)carbamoyl)benzyl)carbamoyl)oxy) methyl)-1H-1,2,3-triazol-1-yl)octanoic acid (6b')

Prop-2-yn-1-yl (4-((2-((tert-butoxycarbonyl)amino)-4-fluorophenyl)carbamoyl)benzyl) carbamate (0.10 g, 0.23 mmol), 8-azidooctanoic acid (0.04 g, 0.23 mmol) and CuI (0.007 g, 0.07 mmol) were suspended in degassed DMF:MeCN (5 mL, 2:1) under an inert atmosphere. 2,6-Lutidine (0.05 mL, 0.46 mmol) was added and the reaction stirred at room temperature for 16 h. The solvent was removed under reduced pressure and suspended in EtOAc (10 mL), washed with sat. $NaHCO_3$ (2 x 5 mL), brine (2 x 5 mL), the organic layers combined, dried over $MgSO_4$, filtered and the solvent removed under reduced pressure. The crude oil was purified by flash chromatography on silica (3:2 THF:Hexane) to provide the *title compound* as an off white crystalline solid (0.07 g, 49%). ^{1}H NMR (400 MHz, $CDCl_3$) δ_H ppm 9.06 (1H, bs, H^{12}), 7.84 (2H, d, J = 7.0 Hz, H^{9}), 7.61 (1H, s, H^{1}), 7.46 (1H, m, H^{14}), 7.28 (2H,

d, J = 7.0 Hz, H^{8}), 7.22 (1H, m, H^{17}), 6.79 (1H, ddd, J = 8.0 Hz 3.0 Hz, $^{3}J_{HF}$ 8.0 Hz, H^{15}), 5.69 (1H, t, J = 6.0 Hz, H^{5}), 5.18 (2H, s, H^{3}), 4.38 (2H, d, J = 6.0 Hz, H^{6}), 4.31 (2H, t, J = 8.5 Hz, H^{23}), 2.28 (2H, t, J = 9.0 Hz, H^{29}), 1.88-1.84 (2H, m, H^{24}), 1.58-1.55 (2H, m, H^{28}), 1.47 (9H, s, H^{22}), 1.31-1.26 (6H, m, $H^{25, 26, 27}$); ^{13}C NMR (101 MHz, $CDCl_3$) δ_C ppm 177.6 (C^{30}), 166.0 (C^{11}), 160.6 (d, $^{1}J_{CF}$ 250 Hz, C^{16}), 156.6 (C^{4}), 148.6 (C^{20}), 143.1 (C^{2}), 142.8 (C^{7}). 132.9 (d, $^{3}J_{CF}$ 11 Hz, C^{18}), 132.8 (C^{10}), 127.8 (C^{9}), 127.4 (d, $^{3}J_{CF}$ 12 Hz, C^{14}), 127.3 (C^{8}), 125.4 (d, $^{4}J_{CF}$ 5.0 Hz, C^{13}), 123.8 (C^{1}), 111.8 (d, $^{2}J_{CF}$ 22 Hz, C^{15}), 110.7 (d, $^{2}J_{CF}$ 27 Hz, H^{17}), 81.8 (C^{21}), 58.1 (C^{3}), 50.3 (C^{23}), 44.7 (C^{6}), 33.8 (C^{29}), 29.8 (C^{24}), 28.6 (C^{27}), 28.4 (C^{26}), 28.2 (C^{22}), 26.1 (C^{25}), 24.4 (C^{28}); ^{19}F NMR (376 MHz, $CDCl_3$) δ_F ppm -114.5 (1F, F^{16}); *m/z* ($HRMS^+$) 649.2764 $[M+Na]^+$ ($C_{31}H_{39}N_6O_7FNa$ requires 649.2762); R_f = 0.23 (7% MeOH:DCM).

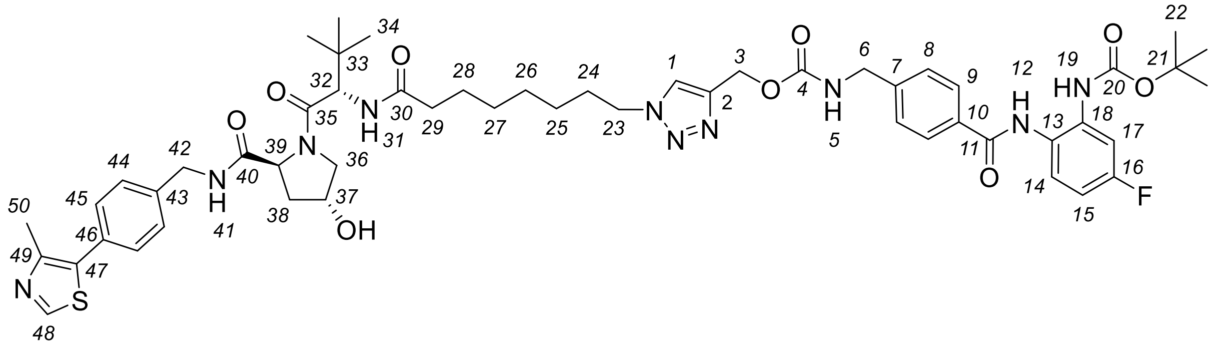

(1-(8-(((S)-1-((2S,4R)-4-Hydroxy-2-((4-(4-methylthiazol-5-yl)benzyl)carbamoyl)pyrrolidin-1-yl)-3,3-dimethyl-1-oxobutan-2-yl)amino)-8-oxooctyl)-1H-1,2,3-triazol-4-yl)methyl(4-((2-((tert-butoxy carbonyl)amino)-4-fluorophenyl)carbamoyl)benzyl)carbamate (8b')

8-(4-((((4-((2-((Tert-butoxycarbonyl)amino)-4-fluorophenyl)carbamoyl)benzyl)carbamoyl)oxy) methyl)-1H-1,2,3-triazol-1-yl)octanoic acid (0.07 g, 0.11 mmol), HATU (0.05 g, 0.12 mmol) and diisopropylethylamine (0.05 mL, 0.30 mmol) were dissolved in anhydrous DMF (1 mL), the reaction mixture cooled to 0 °C and stirred for 15 mins. A solution of (4*R*)-3-Methyl-L-valyl-4-hydroxy-*N*-[[4-(4-methyl-5-thiazolyl)phenyl]methyl]-L-prolinamide hydrochloride (0.05 g, 0.10 mmol) in anhydrous DMF (1 mL) was added slowly and the resulting solution was stirred at room temperature for 16 h. The solvent was removed under reduced pressure, dissolved in EtOAc (10 mL), washed with sat. $NaHCO_3$ (2 x 10 mL), brine (1 x 10 mL), organic layers combined, dried over $MgSO_4$, filtered and solvent removed under reduced pressure. The crude oil was purified using flash chromatography on silica (3-7% MeOH:DCM solvent gradient) to provide the *title compound* as a pale yellow oil (0.08 g, 68%). ^{1}H NMR (400 MHz, MeOD-d_4) δ_H ppm 8.75 (1H, s, H^{48}), 7.86 (1H, s, H^{1}), 7.81 (2H, d, J = 8.5 Hz, H^{9}), 7.38-7.28 (8H, m, $H^{8, 14, 17, 44, 45}$), 6.80 (1H, ddd, J = 8.5 Hz 3.0 Hz, $^{3}J_{HF}$ 9.0 Hz, H^{15}), 5.06 (2H, s, H^{3}), 4.50 (1H, s, H^{32}), 4.48-4.37 (3H, m, $H^{37, 39, 42}$), 4.30-4.22 (5H, m, $H^{6, 23, 42'}$), 3.80-3.76 (1H, m, H^{36}), 3.69-3.66 (1H, m, $H^{36'}$), 2.35 (3H, s, H^{50}), 2.16-2.07 (3H, m, $H^{29, 38}$), 2.00-1.93 (1H, m, $H^{38'}$), 1.80-1.74 (2H, m, H^{24}), 1.50-1.42 (2H, m, H^{28}), 1.37 (9H, s, H^{22}), 1.29-1.15 (6H, m, $H^{25, 26, 27}$), 0.91 (9H, s, H^{34}); ^{13}C NMR (101 MHz, MeOD-d_4) δ_C ppm 174.0 (C^{30}), 172.3 (C^{40}), 171.1 (C^{35}), 167.2 (C^{11}), 160.9 (d, $^{1}J_{CF}$

250 Hz, C^{16}), 157.2 (C^{4}), 151.4 (C^{48}), 147.6 (C^{49}), 143.8 (C^{7}), 143.1 (C^{2}), 141.5 (C^{20}), 138.9 (C^{43}), 134.3 (d, $^{3}J_{CF}$ 11 Hz, C^{18}), 132.5 (C^{10}), 132.0 (C^{47}), 130.1 (C^{46}), 128.9 (C^{45}), 127.7 (d, $^{3}J_{CF}$ 10 Hz, C^{14}), 127.6 (C^{8}), 127.5 (C^{9}), 126.9 (C^{44}), 125.0 (d, $^{4}J_{CF}$ 3.0 Hz, C^{13}), 124.2 (C^{1}), 110.6 (d, $^{2}J_{CF}$ 22 Hz, C^{15}), 109.5 (d, $^{2}J_{CF}$ 29 Hz, C^{17}), 80.5 (C^{21}), 69.7 (C^{37}), 59.4 (C^{39}), 57.5 (C^{32}), 57.3 (C^{3}), 56.5 (C^{36}), 50.1 (C^{23}), 43.7 (C^{6}), 42.4 (C^{42}), 37.6 (C^{38}), 35.2 (C^{29}), 35.1 (C^{33}), 29.7 (C^{24}), 28.5 (C^{27}), 28.2 (C^{26}), 27.2 (C^{22}), 26.6 (C^{34}), 25.9 (C^{25}), 25.3 (C^{28}), 14.4 (C^{50}); ^{19}F NMR (376 MHz, MeOD-*d4*) δ_F ppm -116.5 (1F, F^{16}); *m/z* (HRMS$^+$) 1039.4874 $[M+H]^+$ ($C_{53}H_{68}N_{10}O_9FS$ requires 1039.4875); R_f = 0.21 (7%MeOH:DCM).

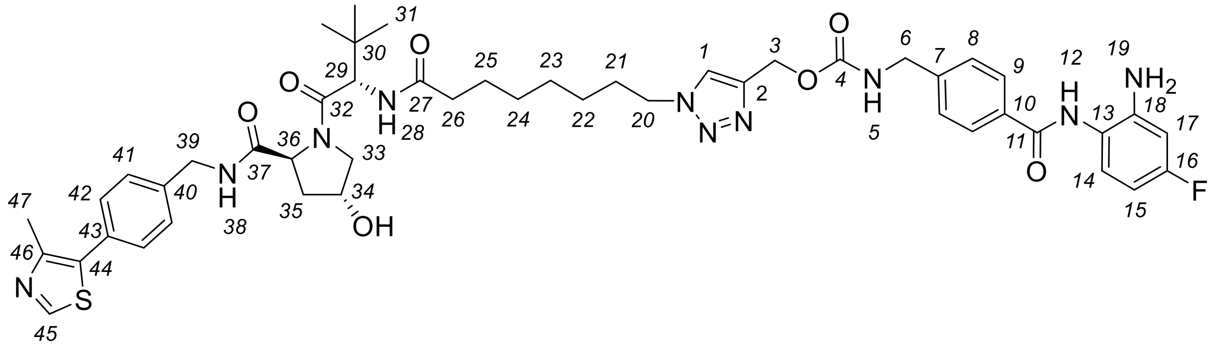

(1-(8-(((S)-1-((2S,4R)-4-Hydroxy-2-((4-(4-methylthiazol-5-yl)benzyl)carbamoyl)pyrrolidin-1-yl)-3,3-dimethyl-1-oxobutan-2-yl)amino)-8-oxooctyl)-1H-1,2,3-triazol-4-yl)methyl(4-((2-amino-4-fluorophenyl)carbamoyl)benzyl)carbamate (12)

TFA (0.2 mL) was added to a stirring solution of (1-(8-(((*S*)-1-((2*S*,4*R*)-4-hydroxy-2-((4-(4-methylthiazol-5-yl)benzyl)carbamoyl)pyrrolidin-1-yl)-3,3-dimethyl-1-oxobutan-2-yl)amino)-8-oxo octyl)-1H-1,2,3-triazol-4-yl)methyl(4-((2-((tert-butoxycarbonyl)amino)-4-fluorophenyl)carbamoyl) benzyl)carbamate (0.08 g, 0.08 mmol) in DCM (2 mL) and the resulting reaction mixture was stirred at room temperature overnight. The reaction was concentrated under reduced pressure to afford a yellow oil. The crude oil was dissolved in MeOH (2 mL), agitated in MP-carbonate resin (3.02 mmol/g loading capacity, 325 mg) for 3 h and filtered. The compound was lyophilised from H_2O:MeCN (1.5 mL, 1:1) to provide the *title compound* as an off white powder (0.05 g, 77%). ^{1}H NMR (400 MHz, MeOD-*d4*) δ_H ppm 8.75 (1H, s, H^{45}), 7.86 (1H, s, H^{1}), 7.82 (2H, d, *J* = 8.5 Hz, H^{9}), 7.35 (2H, d, *J* = 8.5 Hz, H^{8}), 7.31-7.27 (4H, m, $H^{41, 42}$), 7.00 (1H, dd, *J* = 8.5 Hz, $^{4}J_{HF}$ 6.0Hz, H^{14}), 6.48 (1H, dd, *J* = 3.0 Hz, $^{3}J_{HF}$ 10 Hz, H^{17}), 6.31 (1H, ddd, *J* = 8.5 Hz 3.0 Hz, $^{3}J_{HF}$ 9.0 Hz, H^{15}), 5.06 (2H, s, H^{3}), 4.51 (1H, s, H^{29}), 4.48-4.35 (3H, m, $H^{34,36, 39}$), 4.29-4.22 (5H, m, $H^{6, 20, 39'}$), 3.79-3.76 (1H, m, H^{33}), 3.69-3.65 (1H, m, $H^{33'}$), 2.35 (3H, s, H^{47}), 2.18-2.07 (3H, m, $H^{26, 35}$), 2.00-1.93 (1H, m, $H^{35'}$), 1.80-1.74 (2H, m, H^{21}), 1.50-1.42 (2H, m, H^{25}), 1.25-1.14 (6H, m, $H^{22, 23, 24}$), 0.91 (9H, s, H^{31}); ^{13}C NMR (101 MHz, MeOD-*d4*) δ_C ppm 174.8 (C^{27}), 173.2 (C^{37}), 171.0 (C^{32}), 167.6 (C^{11}), 162.6 (d, $^{1}J_{CF}$ 250 Hz, C^{16}), 157.2 (C^{4}), 151.4 (C^{45}), 147.6 (C^{46}), 145.1 (d, $^{3}J_{CF}$ 11 Hz, C^{18}), 143.4 (C^{7}), 143.2 (C^{2}), 138.8 (C^{40}), 132.8 (C^{44}), 132.1 (C^{43}), 132.0 (C^{10}), 128.9 (C^{42}), 128.1 (d, $^{3}J_{CF}$ 11 Hz, C^{14}), 127.6 (C^{9}), 127.5 (C^{8}),126.9 (C^{41}), 124.2 (C^{1}), 119.2 (d, $^{4}J_{CF}$ 2.5 Hz, C^{13}), 103.6 (d, $^{3}J_{CF}$ 23 Hz, C^{15}), 102.5 (d, $^{3}J_{CF}$ 26 Hz, C^{17}), 69.7 (C^{34}), 59.4 (C^{36}), 57.5 (C^{29}),

57.3 (C^{3}), 56.6 (C^{33}), 50.1 (C^{20}), 43.8 (C^{6}), 42.3 (C^{39}), 37.5 (C^{35}), 35.2 (C^{26}), 35.1 (C^{30}), 29.7 (C^{21}), 28.5 (C^{24}), 28.2 (C^{23}), 25.8 (C^{22}), 25.6 (C^{31}), 25.4 (C^{25}), 14.4 (C^{47}); ^{19}F NMR (376 MHz, MeOD-*d*$_4$) δ_F ppm -117.4 (1F, F^{16}); *m/z* (HRMS$^+$) 939.4318 [M+H]$^+$ ($C_{48}H_{60}N_{10}O_7FS$ requires 939.4351).

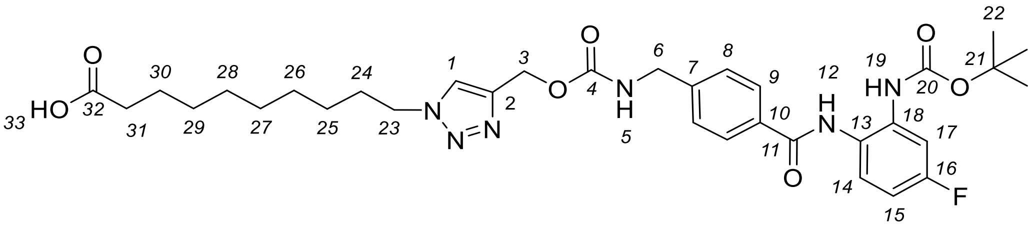

10-(4-((((4-((2-((Tert-butoxycarbonyl)amino)-4-fluorophenyl)carbamoyl)benzyl)carbamoyl)oxy) methyl)-1H-1,2,3-triazol-1-yl)decanoic acid (6c')

Prop-2-yn-1-yl (4-((2-((tert-butoxycarbonyl)amino)-4-fluorophenyl)carbamoyl)benzyl) carbamate (0.16 g, 0.35 mmol), 10-azidodecanoic acid (0.11 g, 0.51 mmol) and CuI (0.01 g, 0.10 mmol) were suspended in degassed DMF:MeCN (5 mL, 2:1) under an inert atmosphere. 2,6-Lutidine (0.06 mL, 0.52 mmol) was added and the reaction stirred at room temperature for 16 h. The solvent was removed under reduced pressure and suspended in EtOAc (10 mL), washed with sat. $NaHCO_3$ (2 x 5 mL), brine (2 x 5 mL), the organic layers combined, dried over $MgSO_4$, filtered and the solvent removed under reduced pressure. The crude oil was purified by flash chromatography on silica (4-7%MeOH:DCM gradient) to provide the *title compound* as an off white solid (0.20 g, 87%). ^{1}H NMR (400 MHz, $CDCl_3$) δ_H ppm 8.97 (1H, bs, H^{12}), 7.86 (2H, d, *J* = 7.0 Hz, H^{9}), 7.61 (1H, s, H^{1}), 7.50 (1H, dd, *J* = 6.5 Hz, $^4J_{HF}$ 8.5 Hz, H^{14}), 7.20 (1H, dd, *J* = 2.5 Hz, $^3J_{HF}$ 10 Hz, H^{17}), 7.31 (2H, d, *J* = 7.0 Hz, H^{8}), 6.82 (1H, ddd, *J* = 8.5 Hz 2.5 Hz, $^3J_{HF}$ 2.5 Hz, H^{15}), 5.50 (1H, t, *J* = 6.0 Hz, H^{5}), 5.20 (2H, s, H^{3}), 4.39 (2H, d, *J* = 6.0 Hz, H^{6}), 4.31 (2H, t, *J* = 6.5 Hz, H^{23}), 2.30 (2H, t, *J* = 6.5 Hz, H^{31}), 1.88-1.84 (2H, m, H^{24}), 1.62-1.54 (2H, m, H^{30}), 1.48 (9H, s, H^{22}), 1.32-1.22 (10H, m, H$^{25, 26, 27, 28, 29}$); ^{13}C NMR (101 MHz, $CDCl_3$) δ_C ppm 177.2 (C^{32}), 166.0 (C^{11}), 160.7 (d, $^1J_{CF}$ 246 Hz, C^{16}), 156.5 (C^{4}), 147.9 (C^{20}), 143.1 (C^{2}), 142.8 (C^{7}), 132.9 (d, $^3J_{CF}$ 10 Hz, C^{18}), 132.8 (C^{10}), 127.8 (C^{9}), 127.4 (d, $^3J_{CF}$ 11 Hz, C^{14}), 127.3 (C^{8}), 125.3 (d, $^4J_{CF}$ 7.0 Hz, C^{13}), 123.4 (C^{1}), 112.0 (d, $^2J_{CF}$ 22 Hz, C^{15}), 110.8 (d, $^2J_{CF}$ 27 Hz, C^{17}), 81.6 (C^{21}), 58.2 (C^{3}), 50.4 (C^{23}), 44.6 (C^{6}), 33.8 (C^{31}), 30.1 (C^{24}), 28.9-26.2 (C$^{25, 26, 27, 28, 29}$), 28.2 (C^{22}), 24.4 (C^{30}); ^{19}F NMR (376 MHz, $CDCl_3$) δ_F ppm -114.6 (1F, F^{16}); *m/z* (HRMS$^+$) 677.3079 [M+Na]$^+$ ($C_{33}H_{43}FN_6O_7Na$ requires 677.3075); R_f = 0.14 (7%MeOH:DCM).

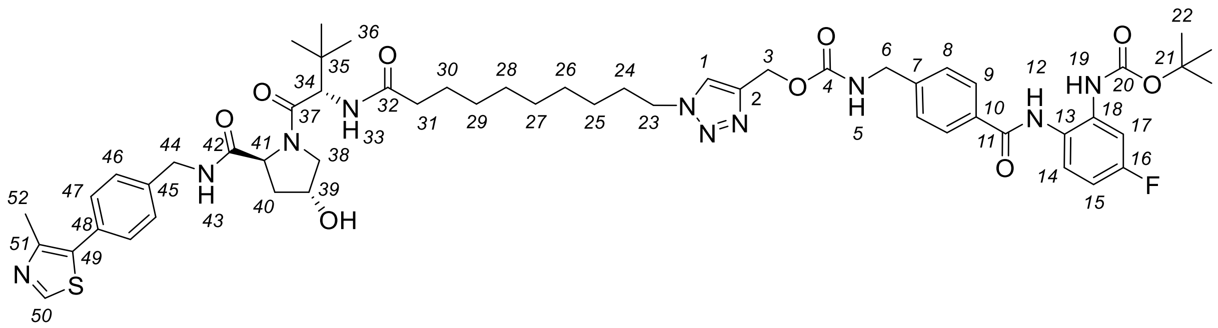

(1-(10-(((S)-1-((2S,4R)-4-Hydroxy-2-((4-(4-methylthiazol-5-yl)benzyl)carbamoyl)pyrrolidin-1-yl)-3,3-dimethyl-1-oxobutan-2-yl)amino)-10-oxodecyl)-1H-1,2,3-triazol-4-yl)methyl(4-((2-((tert-butoxy carbonyl)amino)-4-fluorophenyl)carbamoyl)benzyl)carbamate (8c')

10-(4-((((4-((2-((Tert-butoxycarbonyl)amino)-4-fluorophenyl)carbamoyl)benzyl)carbamoyl)oxy) methyl)-1*H*-1,2,3-triazol-1-yl)decanoic acid (0.07 g, 0.10 mmol) and HATU (0.05 g, 0.13 mmol) were charged to a flask, dissolved in anhydrous DMF (2 mL) under an inert atmosphere and the solution cooled to 0 °C. Diisopropylethylamine (0.05 mL, 0.29 mmol) was added and the reaction stirred for 15 mins. (4*R*)-3-Methyl-L-valyl-4-hydroxy-*N*-[[4-(4-methyl-5-thiazolyl)phenyl]methyl]-L-prolinamide hydrochloride (0.05 g, 0.10 mmol) was dissolved in anhydrous DMF (1 mL) and added, the reaction allowed to warm to room temperature and stirred for 16 h. Solvent was removed under reduced pressure and redissolved in EtOAc (10 mL), extracted with saturated $NaHCO_3$ solution (2 x 10 mL), brine (2 x 10 mL), organic layers combined, dried over $MgSO_4$, filtered and solvent removed under reduced pressure. The crude oil was purified by flash chromatography on silica (2-7% MeOH:DCM solvent gradient) to provide the *title compound* as a bright-yellow crystalline solid (0.06 g, 62%). ^{1}H NMR (400 MHz, MeOD-*d₄*) δ_H ppm 8.76 (1H, s, H^{50}), 7.86 (1H, s, H^{1}), 7.81 (2H, d, J = 9.0 Hz, H^9), 7.38-7.28 (8H, m, $H^{8, 14, 17, 46, 47}$), 6.80 (1H, ddd, J = 8.0 Hz 3.0 Hz, $^3J_{HF}$ 8.5 Hz, H^{15}), 5.06 (2H, s, H^3), 4.52 (1H, s, H^{34}), 4.48-4.37 (3H, m, $H^{39, 41, 44}$), 4.30-4.22 (5H, m, $H^{6, 23, 44'}$), 3.80-3.77 (1H, m, H^{38}), 3.70-3.66 (1H, m, $H^{38'}$), 2.36 (3H, s, H^{52}), 2.20-2.07 (3H, m, $H^{31, 40}$), 2.00-1.93 (1H, m, $H^{40'}$), 1.80-1.74 (2H, m, H^{24}, 1.50-1.43 (2H, m, H^{30}), 1.37 (9H, s, H^{22}), 1.21-1.14 (10H, m, $H^{25, 26, 27, 28, 29}$), 0.92 (9H, s, H^{36}); ^{13}C NMR (101 MHz, MeOD-*d₄*) δ_C ppm 174.6 (C^{32}), 173.0 (C^{42}), 170.9 (C^{37}), 167.1 (C^{11}), 160.7 (d, $^1J_{CF}$ 241 Hz, C^{16}), 157.1 (C^4), 155.2 (C^{20}), 151.5 (C^{50}), 147.6 (C^{51}), 143.8 (C^7), 143.1 (C^2), 138.8 (C^{45}), 134.3 (d, $^3J_{CF}$ 11 Hz, C^{18}), 132.5 (C^{10}), 132.0 (C^{49}), 130.0 (C^{48}), 128.9 (C^{47}), 127.7 (d, $^3J_{CF}$ 9.0 Hz, C^{14}), 127.6 (C^9), 127.5 (C^8), 126.9 (C^{46}), 125.0 ($^4J_{CF}$ 4.0 Hz, C^{13}), 124.2 (C^1), 109.5 (d, $^2J_{CF}$ 26 Hz, C^{17}), 101.6 (d, $^2J_{CF}$ 22 Hz, C^{15}), 80.7 (C^{21}), 69.7 (C^{39}), 59.4 (C^{41}), 57.5 (C^{34}), 57.3 (C^3), 56.6 (C^{38}), 50.0 (C^{23}), 43.8 (C^6), 42.3 (C^{44}), 37.5 (C^{40}), 35.2 (C^{31}), 35.1 (C^{35}), 29.8 (C^{24}), 28.9-25.9 ($C^{25, 26, 27, 28, 29}$), 27.2 (C^{22}), 25.6 (C^{36}), 25.5 (C^{30}), 14.4 (C^{52}); ^{19}F NMR (376 MHz, MeOD-*d₄*) δ_F ppm -116.6 (1F, F^{16}); *m/z* (HRMS$^+$) 1067.5145 $[M+H]^+$ ($C_{55}H_{72}FN_{10}O_9S$ requires 1067.5188); R_f = 0.20 (7%MeOH:EtOAc).

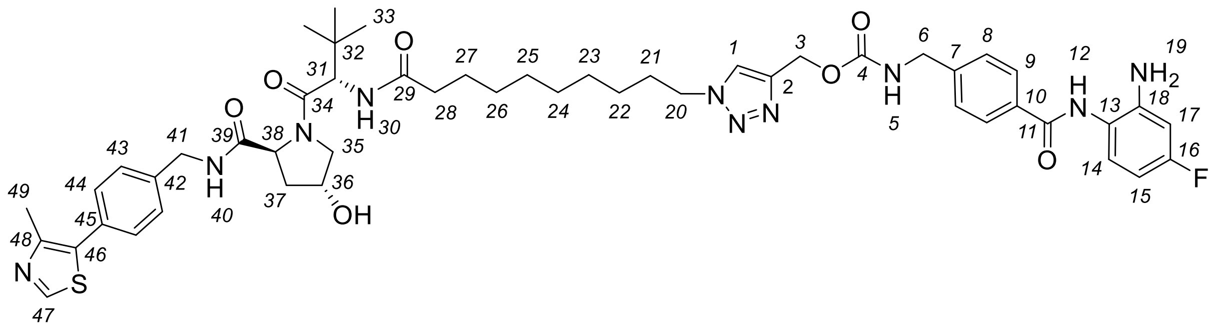

(1-(10-(((S)-1-((2S,4R)-4-Hydroxy-2-((4-(4-methylthiazol-5-yl)benzyl)carbamoyl)pyrrolidin-1-yl)-3,3-dimethyl-1-oxobutan-2-yl)amino)-10-oxodecyl)-1H-1,2,3-triazol-4-yl)methyl(4-((2-amino-4-fluorophenyl)carbamoyl)benzyl)carbamate (14)

TFA (0.2 mL) was added to a stirring solution of (1-(10-(((*S*)-1-((2*S*,4*R*)-4-hydroxy-2-((4-(4-methylthiazol-5-yl)benzyl)carbamoyl)pyrrolidin-1-yl)-3,3-dimethyl-1-oxobutan-2-yl)amino)-10-oxo decyl)-1H-1,2,3-triazol-4-yl)methyl(4-((2-((tert-butoxycarbonyl)amino)-4-fluorophenyl)carbamoyl) benzyl)carbamate (0.06 g, 0.06 mmol) in DCM (2 mL) and the resulting reaction mixture was stirred at room temperature overnight. The reaction was concentrated under reduced pressure to afford a yellow oil. The crude oil was dissolved in MeOH (2 mL), agitated in MP-carbonate resin (3.02 mmol/g loading capacity, 250 mg) for 3 h and filtered. The compound was lyophilised from H_2O:MeCN (1.5 mL, 1:1) to provide the *title compound* as an off white powder (0.06 g, 84%). ^{1}H NMR (400 MHz, MeOD-d_4) δ_H ppm 8.76 (1H, s, H^{47}), 7.87 (1H, s, H^{1}), 7.83 (2H, d, J = 8.0 Hz, H^9), 7.36-7.29 (6H, m, $H^{8, 43, 44}$), 7.01 (1H, dd, J = 8.0 Hz, $^4J_{HF}$ 6.0 Hz, H^{14}), 6.48 (1H, dd, J = 3.0 Hz, $^3J_{HF}$ 10 Hz, H^{17}), 6.31 (1H, ddd, J = 8.0 Hz 3.0 Hz, $^3J_{HF}$ 8.5 Hz, H^{15}), 5.07 (2H, s, H^3), 4.53 (1H, s, H^{31}), 4.49-4.37 (3H, m, $H^{36, 38, 41}$), 4.30-4.23 (5H, m, $H^{6, 20, 41'}$), 3.81-3.78 (1H, m, H^{35}), 3.71-3.67 (1H, m, $H^{35'}$), 2.37 (3H, s, H^{49}), 2.21-2.08 (3H, m, $H^{28, 37}$), 2.01-1.94 (1H, m, $H^{37'}$), 1.81-1.74 (2H, m, H^{21}), 1.51-1.43 (2H, m, H^{27}), 1.26-1.16 (10H, m, $H^{22, 23, 24, 25, 26}$), 0.92 (9H, s, H^{33}); ^{13}C NMR (101 MHz, MeOD-d_4) δ_C ppm 174.6 (C^{29}), 173.0 (C^{39})170.9 (C^{34}), 167.5 (C^{11}), 162.2 (d, $^1J_{CF}$ 237 Hz, C^{16}), 157.3 (C^4), 151.4 (C^{47}), 147.8 (C^{48}), 145.0 (d, $^3J_{CF}$ 11 Hz, C^{18}), 143.4 (C^7), 143.1 (C^2), 138.8 (C^{42}), 132.8 (C^{10}), 132.0 (C^{45}), 130.1 (C^{46}), 128.9 (C^{44}), 128.1 (d, $^3J_{CF}$ 11 Hz, C^{14}), 127.6 (C^9), 127.5 (C^8), 126.9 (C^{43}), 124.2 (C^1), 119.2 (d, $^4J_{CF}$ 2.0 Hz, C^{13}), 103.6 (d, $^2J_{CF}$ 23 Hz, C^{15}), 102.5 (d, $^2J_{CF}$ 25 Hz, C^{17}), 69.6 (C^{36}), 59.4 (C^{38}), 57.5 (C^{31}), 57.3 (C^3), 56.6 (C^{35}), 49.9 (C^{20}), 43.8 (C^6), 42.3 (C^{41}), 37.5 (C^{37}), 35.2 (C^{28}), 35.1 (C^{32}), 29.8 (C^{21}), 28.8-25.9 ($C^{22, 23, 24, 25, 26}$), 25.6 (C^{33}), 25.5 (C^{27}), 14.4 (C^{49}); ^{19}F NMR (376 MHz, MeOD-d_4) δ_F ppm -117.4 (1F, F^{16}); *m/z* (HRMS$^+$) 989.4492 $[M+Na]^+$ ($C_{50}H_{63}FN_{10}O_7SNa$ requires 989.4484).

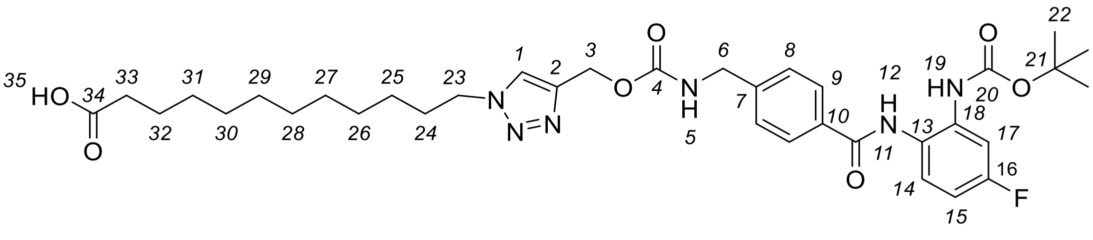

12-(4-((((4-((2-((Tert-butoxycarbonyl)amino)-4-fluorophenyl)carbamoyl)benzyl)carbamoyl)oxy) methyl)-1H-1,2,3-triazol-1-yl)dodecanoic acid (6d')

Prop-2-yn-1-yl (4-((2-((tert-butoxycarbonyl)amino)-4-fluorophenyl)carbamoyl)benzyl) carbamate (0.09 g, 0.21 mmol), 12-azidododecanoic acid (0.05 g, 0.21 mmol) and CuI (0.007 g, 0.06 mmol) were suspended in degassed DMF:MeCN (5 mL, 2:1) under an inert atmosphere. 2,6-Lutidine (0.04 mL, 0.31 mmol) was added and the reaction stirred at room temperature for 16 h. The solvent was removed under reduced pressure and suspended in EtOAc (10 mL), washed with sat. $NaHCO_3$ (2 x 5 mL), brine (2 x 5 mL), the organic layers combined, dried over $MgSO_4$, filtered and the solvent removed under reduced pressure. The crude oil was purified by flash chromatography on silica (40:60-60:40 THF:Hexane gradient) to provide the *title compound* as an off white solid (0.08 g, 57%). ^{1}H NMR (400 MHz, $CDCl_3$) δ_H ppm 8.94 (1H, s, H^{12}), 7.89-7.86 (2H, d, J = 8.0 Hz, H^9), 7.62 (1H, s, H^1), 7.55-7.52 (1H, dd, J = 8.5 Hz, $^3J_{HF}$ 8.5 Hz, H^{15}), 7.34-7.32 (2H, d, J = 8.0 Hz, H^8), 7.21-7.17 (1H, dd, $^3J_{HF}$ 9.0 Hz, J = 2.0 Hz, H^{17}), 6.98 (1H, s, H^{19}), 6.87-6.82 (1H, ddd, J = 8.5 Hz 1.0 Hz, $^4J_{HF}$ 3.0 Hz, H^{14}), 5.41 (1H, t, J = 6.5 Hz, H^5), 5.23 (2H, s, H^3), 4.40 (2H, d, J = 6.5 Hz, H^6), 4.33 (2H, t, J = 7.0 Hz, H^{23}), 2.32 (2H, t, J = 7.0 Hz, H^{33}), 1.88 (2H, tt, J = 7.0 Hz 7.0 Hz, H^{24}), 1.62 (2H, tt, J = 7.0 Hz 7.0 Hz, H^{32}), 1.50 (9H, s, H^{22}), 1.29-1.27 (8H, m, $H^{31, 30, 26, 25}$), 1.25-1.24 (6H, m, $H^{29, 28, 27}$); ^{13}C NMR (101 MHz, $CDCl_3$) δ_C ppm 178.3 (C^{34}), 166.1 (C^{11}), 161.7 (C^4), 160.6 ($^1J_{CF}$ 246 Hz, C^{16}), 154.1 (C^{20}), 143.1 (C^2),142.8 (C^7), 135.8 (C^{18}), 133.0 (C^{10}), 132.8 (C^{13}), 127.8 (C^9), 127.4 ($^2J_{CF}$ 10 Hz, C^{15}), 127.3 (C^8), 123.8 (C^1), 111.1 ($^2J_{CF}$ 22 Hz, H^{14}), 110.6 ($^2J_{CF}$ 26 Hz, C^{17}), 81.6 (C^{21}), 58.1 (C^3), 50.4 (C^{23}), 44.6 (C^6), 34.2 (C^{33}), 30.2 (C^{24}), 29.4-28.8 ($C^{31, 30, 29, 28, 27, 26, 25}$), 28.2 ($C^{22}$), 24.6 ($C^{32}$); ^{19}F NMR (376 MHz, $CDCl_3$) δ_F ppm -114.5; *m/z* ($HRMS^+$) 705.3386 $[M+Na]^+$ ($C_{35}H_{47}FN_6O_7Na$ requires 705.3388); R_f = 0.21 (50:50 Hexane:EtOAc).

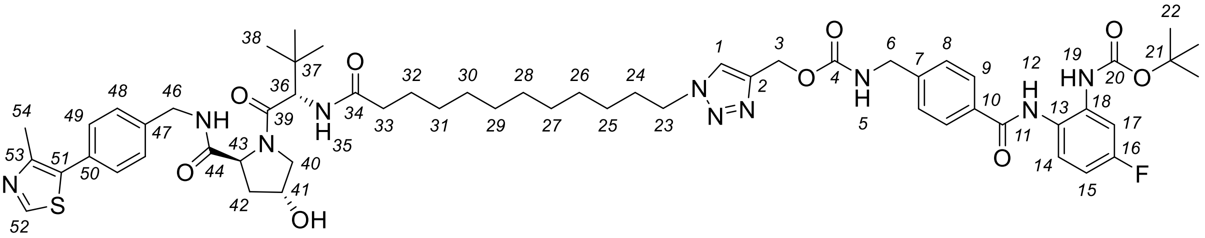

(1-(12-(((S)-1-((2S,4R)-4-Hydroxy-2-((4-(4-methylthiazol-5-yl)benzyl)carbamoyl)pyrrolidin-1-yl)-3,3-dimethyl-1-oxobutan-2-yl)amino)-12-oxododecyl)-1H-1,2,3-triazol-4-yl)methyl(4-((2-((tert-butoxy carbonyl)amino)-4-fluorophenyl)carbamoyl)benzyl)carbamate (8d')

12-(4-((((4-((2-((Tert-butoxycarbonyl)amino)-4-fluorophenyl)carbamoyl)benzyl)carbamoyl)oxy) methyl)-1H-1,2,3-triazol-1-yl)dodecanoic acid (0.08 g, 0.12 mmol), HATU (0.05 g, 0.13 mmol) and diisopropylethylamine (0.05 mL, 0.30 mmol) were dissolved in anhydrous DMF (1 mL), the reaction mixture cooled to 0 °C and stirred for 15 mins. A solution of (4*R*)-3-Methyl-L-valyl-4-hydroxy-*N*-[[4-(4-methyl-5-thiazolyl)phenyl]methyl]-L-prolinamide hydrochloride (0.05 g, 0.10 mmol) in anhydrous DMF (1 mL) was added slowly and the resulting solution was stirred at room temperature for 16 h. The

solvent was removed under reduced pressure, redissolved in EtOAc (10 mL), washed with sat. $NaHCO_3$ (2 x 10 mL), brine (1 x 10 mL), organic layers combined, dried over $MgSO_4$, filtered and solvent removed under reduced pressure. The crude oil was purified using flash chromatography on silica (3-7% MeOH:DCM solvent gradient) to provide the *title compound* as a pale yellow oil (0.04 g, 32%). ^{1}H NMR (400 MHz, MeOD-d_4) δ_H ppm 8.76 (1H, s, H^{52}), 7.86 (1H, s, H^{1}), 7.81 (2H, d, J = 9.0 Hz, H^9), 7.39-7.29 (8H, m, $H^{8, 14, 17, 48, 49}$), 6.81 (1H, ddd, J = 8.0 Hz 3.0 Hz, $^3J_{HF}$ 9.0 Hz, H^{15}), 5.08 (2H, s, H^3), 4.53 (1H, s, H^{36}), 4.48-4.26 (3H, m, $H^{41, 43, 46}$), 4.30-4.22 (5H, m, $H^{6, 23, 46'}$), 3.80-3.77 (1H, m, H^{40}), 3.71-3.67 (1H, m, $H^{40'}$), 2.36 (3H, s, H^{54}), 2.21-2.07 (3H, m, $H^{33, 42}$), 2.01-1.94 (1H, m, $H^{42'}$), 1.81-1.74 (2H, m, H^{24}), 1.56-1.43 (H^{32}), 1.38 (9H, s, H^{22}), 1.33-1.24 (14H, m, $H^{25, 26, 27, 28, 29, 30, 31}$), 0.92 (9H, s, H^{38}); ^{13}C NMR (101 MHz, MeOD-d_4) δ_C ppm 174.6 (C^{34}), 173.0 (C^{44}), 170.9 (C^{39}), 167.11 (C^{11}), 160.7 (d, $^1J_{CF}$ 241 Hz, C^{16}), 157.2 (C^4), 154.0 (C^{20}), 151.5 (C^{52}), 147.6 (C^{53}), 143.8 (C^7), 143.1 (C^2), 138.9 (C^{47}), 134.2 (d, $^3J_{CF}$ 10 Hz, H^{18}), 132.5 (C^{10}), 132.0 (C^{51}), 130.1 (C^{50}), 128.9 (C^{49}), 127.7 (d, $^3J_{CF}$ 9.0 Hz, C^{14}), 127.6 (C^9), 127.5 (C^8), 126.9 (C^{48}), 125.0 (d, $^4J_{CF}$ 3.5 Hz, C^{13}), 124.1 (C^1), 110.6 (d, $^2J_{CF}$ 22 Hz, C^{15}), 109.5 (d, $^2J_{CF}$ 26 Hz, C^{17}), 80.6 (C^{21}), 69.7 (C^{41}), 59.4 (C^{43}), 57.5 (C^{36}), 57.3 (C^3), 56.6 (C^{40}), 50.0 (C^{23}), 43.7 (C^6), 42.3 (C^{46}), 37.5 (C^{42}), 35.2 (C^{37}), 35.1 (C^{33}), 29.8 (C^{24}), 29.1-25.7 ($C^{25, 26, 27, 28, 29, 30, 31}$), 27.2 ($C^{22}$), 25.6 ($C^{38}$), 25.5 ($C^{32}$), 14.4 ($C^{54}$); ^{19}F NMR (376 MHz, MeOD-d_4) δ_F ppm -116.7 (1F, F^{16}); *m/z* (HRMS$^+$) 1095.5454 $[M+H]^+$ ($C_{57}H_{76}FN_{10}O_9S$ requires 1095.5501); R_f = 0.23 (7%MeOH:DCM).

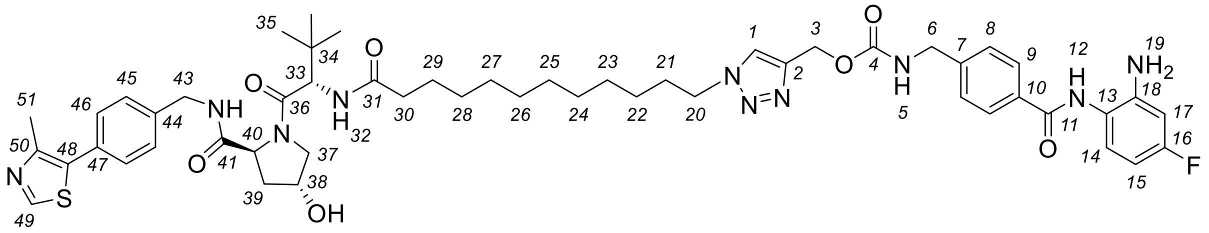

(1-(12-(((S)-1-((2S,4R)-4-Hydroxy-2-((4-(4-methylthiazol-5-yl)benzyl)carbamoyl)pyrrolidin-1-yl)-3,3-dimethyl-1-oxobutan-2-yl)amino)-12-oxododecyl)-1H-1,2,3-triazol-4-yl)methyl(4-((2-amino-4-fluorophenyl)carbamoyl)benzyl)carbamate (16)

TFA (0.2 mL) was added to a stirring solution of (1-(12-(((*S*)-1-((2*S*,4*R*)-4-hydroxy-2-((4-(4-methylthiazol-5-yl)benzyl)carbamoyl)pyrrolidin-1-yl)-3,3-dimethyl-1-oxobutan-2-yl)amino)-12-oxo dodecyl)-1H-1,2,3-triazol-4-yl)methyl(4-((2-((tert-butoxycarbonyl)amino)-4-fluorophenyl)carbamoyl) benzyl)carbamate (0.04 g, 0.04 mmol) in DCM (2 mL) and the resulting reaction mixture was stirred at room temperature overnight. The reaction was concentrated under reduced pressure to afford a yellow oil. The crude oil was dissolved in MeOH (2 mL), agitated in MP-carbonate resin (3.02 mmol/g loading capacity, 205 mg) for 3 h and filtered. Prior to biological evaluation the product was further purified by semi-preparative HPLC (Method 2), and the product lyophilised from H_2O:MeCN (1.5 mL, 1:1) to provide the *title compound* as an off-white powder (0.013 g, 34%). ^{1}H NMR (400 MHz, MeOD-d_4) δ_H ppm 8.77 (1H, s, H^{49}), 7.87 (1H, s, H^1), 7.83 (2H, d, J = 8.0 Hz, H^9), 7.37-7.30 (6H, m, $H^{8, 45, 46}$), 7.01

(1H, dd, J = 8.5 Hz, $^4J_{HF}$ 6.5 Hz, H^{14}), 6.49 (1H, dd, J = 2.5 Hz, $^3J_{HF}$ 11 Hz, H^{17}), 6.32 (1H, ddd, J = 9.0 Hz 2.5 Hz, $^3J_{HF}$ 9.0 Hz, H^{15}), 5.07 (2H, s, H^3), 4.53 (1H, s, H^{33}), 4.48-4.37 (3H, m, $H^{38, 40, 43}$), 4.30-4.22 (5H, m, $H^{6, 20, 43'}$), 3.80-3.77 (1H, m, H^{37}), 3.71-3.67 (1H, m, $H^{37'}$), 2.37 (3H, s, H^{51}), 2.22-2.07 (3H, m, $H^{30, 39}$), 2.00-1.94 (1H, m, $H^{39'}$), 1.82-1.74 (2H, m, H^{21}), 1.51-1.45 (2H, m, H^{29}), 1.23-1.15 (14H, m, $H^{22, 23, 24, 25, 26, 27, 28}$), 0.93 (9H, s, H^{35}); ^{13}C NMR (101 MHz, MeOD-d_4) δ_C ppm 174.6 (C^{31}), 173.0 (C^{41}), 170.9 (C^{36}), 167.3 (C^{11}), 162.2 (d, $^1J_{CF}$ 239 Hz, C^{16}), 157.2 (C^4) ,151.4 (C^{49}), 147.6 (C^{50}), 145.0 (d, $^3J_{CF}$ 11 Hz, C^{18}) 143.4 (C^7), 143.1 (C^2), 138.9 (C^{44}), 132.8 (C^{10}), 132.0 (C^{47}), 130.1 (C^{48}), 128.9 (C^{46}), 128.1 (d, $^3J_{CF}$ 12 Hz, C^{14}), 127.6 (C^9), 127.5 (C^8), 126.9 (C^{45}), 124.1 (C^1), 119.2 (d, $^4J_{CF}$ 4.0 Hz, C^{13}), 103.6 (d, $^2J_{CF}$ 24 Hz, C^{15}), 102.5 (d, $^2J_{CF}$ 27 Hz, C^{17}), 69.7 (C^{38}), 59.4 (C^{40}), 57.5 (C^{33}), 57.3 (C^3), 56.6 (C^{37}), 49.9 (C^{20}), 43.7 (C^6), 42.3 (C^{43}), 37.5 (C^{39}), 35.2 (C^{30}), 35.1 (C^{34}), 29.8 (C^{21}), 29.1-25.9 ($C^{22, 23, 24, 25, 26, 27, 28}$), 25.6 ($C^{35}$), 25.5 ($C^{29}$), 14.4 ($C^{51}$); ^{19}F NMR (376 MHz, MeOD-d_4) δ_F ppm -117.6 (1F, F^{16}); *m/z* (HRMS$^+$) 995.4969 $[M+H]^+$ ($C_{52}H_{68}FN_{10}O_7S$ requires 995.4977).

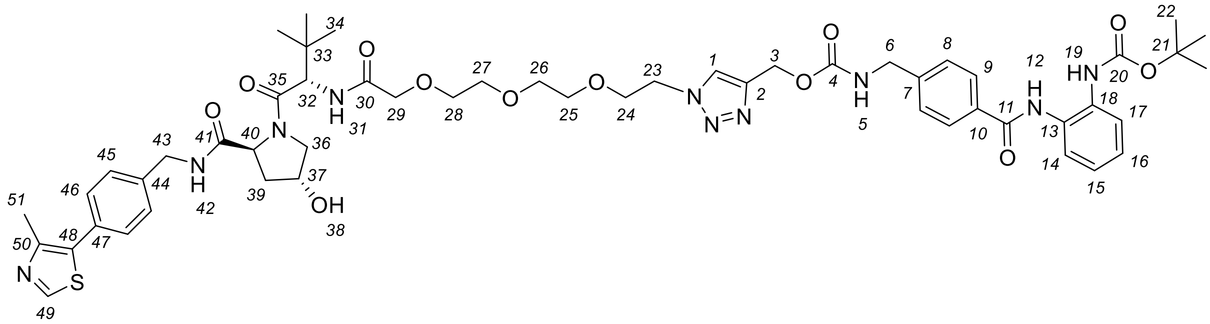

(1-((S)-13-((2S,4R)-4-Hydroxy-2-((4-(4-methylthiazol-5-yl)benzyl)carbamoyl)pyrrolidine-1-carbonyl)-14,14-dimethyl-11-oxo-3,6,9-trioxa-12-azapentadecyl)-1H-1,2,3-triazol-4-yl)methyl(4-((2-((tert-butoxycarbonyl)amino)phenyl)carbamoyl)benzyl)carbamate (20)

Tert-butyl-*N*-{2-[4-({[(prop-2-yn-1-yloxy)carbonyl]amino}methyl)benzamido]phenyl}carbamate (0.03 g, 0.06 mmol), (2*R*,4*R*)-1-((*S*)-14-azido-2-(tert-butyl)-4-oxo-6,9,12-trioxa-3-azatetradecanoyl)-4-hydroxy-*N*-(4-(4-methylthiazol-5-yl)benzyl)pyrrolidine-2-carboxamide (0.05 g, 0.08 mmol) and CuI (0.002 g, 0.02 mmol) were suspended in degassed DMF:MeCN (5 mL, 2:1) under an inert atmosphere. 2,6-Lutidine (0.01 mL, 0.09 mmol) was added and the reaction stirred at room temperature for 16 h. The solvent was removed under reduced pressure and suspended in EtOAc (10 mL), washed with sat. $NaHCO_3$ (2 x 10 mL), brine (2 x 10 mL), the organic layers combined, dried over $MgSO_4$, filtered and the solvent removed under reduced pressure. The crude oil was purified by flash chromatography on silica (5-10%MeOH:DCM gradient) to provide the *title compound* as an off white solid (0.07 g, 41%). ^{1}H NMR (400 MHz, (CD_3CN) δ_H ppm 9.16 (1H, s, H^{12}), 8.79 (1H, s, H^{49}), 7.91-7.88 (3H, m, $H^{1, 9}$), 7.62 (1H, s, H^{19}), 7.59-7.54 (2H, m, $H^{14, 17}$), 7.42-7.37 (6H, m, $H^{8, 45, 46}$), 7.32 (1H, s, H^{42}), 7.28-7.17 (3H, m, $H^{15, 16, 31}$), 6.41-6.38 (1H, m, H^5), 5.15 (2H, s, H^3), 4.60 (1H, d, J = 9.0 Hz, H^{32}), 4.52-4.42 (5H, m, $H^{23, 37, 40, 43}$), 4.36-4.27 (2H, m, $H^{6, 43}$), 3.94 (2H, m, H^{29}), 3.82 (2H, t, J = 6.0 Hz, H^{24}), 3.80-3.76 (1H, m, H^{36}), 3.68-3.65 (1H, m, $H^{36'}$), 3.62-3.55 (8H, m, $H^{25, 26, 27, 28}$), 2.47 (3H, s, H^{51}), 2.15-2.03 (2H, m, H^{39}),

1.48 (9H, s, H[22]), 0.97 (9H, s, H[34]); ^{13}C NMR (101 MHz, (CD_3CN) δ_C ppm 172.4 (C[41]), 170.9 (C[35]), 170.2 (C[30]), 166.4 (C[11]), 157.1 (C[4]), 154.6 (C[20]), 151.7 (C[49]), 148.7 (C[50]), 144.5 (C[2]), 142.4 (C[7]), 139.8 (C[44]), 133.8 (C[47]), 133.5 (C[48]), 132.4 (C[18]), 130.9 (C[10]), 130.6 (C[13]), 129.6 (C[46]), 128.3 (C[9]), 128.2 (C[45]), 127.8 (C[8]), 126.7 (C[16]), 126.3 (C[14]), 125.5 (C[1]), 125.3 (C[15]), 124.7 (C[17]), 81.1 (C[21]), 71.2-70.4 (C[25, 26, 27, 28]), 70.3 (C[37]), 70.2 (C[29]), 69.5 (C[24]), 68.6 (C[33]), 59.7 (C[40]), 58.3 (C[3]), 57.3 (C[36]), 57.1 (C[32]), 50.5 (C[23]), 44.6 (C[6]), 42.7 (C[43]), 37.9 (C[39]), 28.1 (C[22]), 26.4 (C[34]), 15.9 (C[51]); *m/z* (HRMS$^+$) 1091.4598 $[M+Na]^+$ ($C_{53}H_{68}N_{10}O_{12}SNa$ requires 1091.4637); R_f = 0.25 (10% MeOH:DCM).

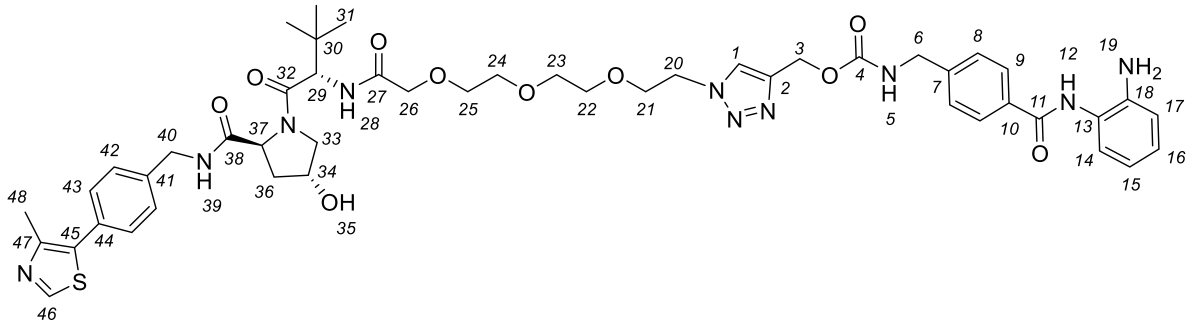

(1-((S)-13-((2S,4R)-4-Hydroxy-2-((4-(4-methylthiazol-5-yl)benzyl)carbamoyl)pyrrolidine-1-carbonyl)-14,14-dimethyl-11-oxo-3,6,9-trioxa-12-azapentadecyl)-1H-1,2,3-triazol-4-yl)methyl(4-((2-amino phenyl)carbamoyl)benzyl)carbamate (21)

TFA (0.2 mL) was added to a stirring solution of (1-((*S*)-13-((2*S*,4*R*)-4-hydroxy-2-((4-(4-methylthiazol-5-yl)benzyl)carbamoyl)pyrrolidine-1-carbonyl)-14,14-dimethyl-11-oxo-3,6,9-trioxa-12 -azapentadecyl)-1H-1,2,3-triazol-4-yl)methyl(4-((2-((tert-butoxycarbonyl)amino)phenyl)carbamoyl) benzyl)carbamate (0.03 g, 0.05 mmol) in DCM (2 mL) and the resulting reaction mixture was stirred at room temperature overnight. The reaction was concentrated under reduced pressure to afford a yellow oil. The crude oil was dissolved in MeOH (2 mL), agitated in MP-carbonate resin (3.02 mmol/g loading capacity, 175 mg) for 3 h and filtered. Prior to biological evaluation the product was further purified by semi-preparative HPLC (Method 1), the solution lyophilised to provide the *title compound* as an off-white solid (0.015 g, 67%). ^{1}H NMR (400 MHz, MeOD-d_4) δ_H ppm 8.88 (1H, s, H[46]), 8.05 (1H, s, H[1]), 7.95 (2H, d, *J* = 8.0 Hz, H[9]), 7.47-7.40 (6H, m, H[8, 42, 43]), 7.20 (1H, dd, *J* = 7.5 Hz 1.5 Hz, H[14]), 7.09 (1H, ddd, *J* = 8.0 Hz 7.5 Hz, 1.5 Hz, H[16]), 6.91 (1H, dd, *J* = 7.5 Hz 1.5 Hz, H[17]), 6.78 (1H, m, H[15]), 5.18 (2H, s, H[3]), 4.71 (1H, s, H[29]), 4.61-4.47 (5H, m, H[20 ,34, 37, 40]), 4.39-4.33 (3H, m, H[6, 40']), 4.02 (2H, m, H[26]), 3.91-3.85 (3H, m, H[21, 33]), 3.82-3.78 (1H, m, H[33']), 3.69-3.59 (8H, m, H[22, 23, 24, 25]), 2.35 (3H, s, H[48]), 2.26-2.20 (1H, m, H[36]), 2.12-2.05 (1H, m, H[36']), 0.92 (9H, s, H[31]); ^{13}C NMR (101 MHz, MeOD-d_4) δ_C ppm 172.9 (C[38]), 170.7 (C[32]), 170.3 (C[27]), 167.2 (C[11]), 157.2 (C[4]), 151.5 (C[46]), 147.7 (C[47]), 143.5 (C[7]), 143.1 (C[2]), 142.5 (C[18]), 138.9 (C[41]), 132.9 (C[10]), 132.0 (C[45]), 130.2 (C[44]), 128.9 (C[43]), 127.7 (C[9]), 127.6 (C[8]), 127.1 (C[14]), 126.9 (C[42]), 126.3 (C[16]), 125.2 (C[1]), 123.9 (C[13]), 118.2 (C[15]), 117.3 (C[17]), 70.9-70.0 (C[22, 23, 24, 25]), 69.7 (C[26]), 69.6 (C[34]), 68.8 (C[21]), 59.4 (C[37]), 57.3 (C[3]), 56.8 (C[29]), 56.7 (C[33]), 50.2

(C^{20}), 44.1 (C^{6}), 42.5 (C^{40}), 37.5 (C^{36}), 35.7 (C^{30}), 25.6 (C^{31}), 14.6 (C^{48}); *m/z* ($HRMS^{+}$) 969.4288 $[M+H]^{+}$ ($C_{48}H_{61}N_{10}O_{10}S$ requires 969.4293).

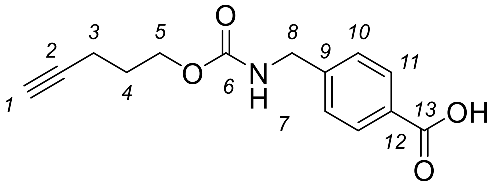

4-((((Pent-4-yn-1-yloxy)carbonyl)amino)methyl)benzoic acid (2)

4-Pentyn-1-ol (0.61 mL, 6.61 mmol) and carbonyldiimidazole (1.07 g, 6.61 mmol) were dissolved in THF (5 mL) and stirred at 10 °C for 1 h. 4-(Aminomethyl)benzoic acid (1.00 g, 6.61 mmol), DBU (0.98 mL, 6.61 mmol) and triethylamine (0.92 mL, 6.61 mmol) were suspended in THF (5 mL) and combined with the CDI-intermediate solution and the reaction stirred at room temperature for 16 h. The solvent was removed under reduced pressure and resuspended in H_2O (10 mL) and the solution was acidified with 1M HCl solution to pH 5. The resulting precipitate was collected by gravity filtration and air dried to provide the *title compound* as an off-white powder (1.19 g, 69%). ^{1}H NMR (400 MHz, DMSO-d_6) δ_H ppm 7.90 (2H, d, J = 8.0 Hz, H^{11}), 7.77 (1H, t, J = 6.0 Hz, H^{7}), 7.36 (2H, d, J = 8.0 Hz, H^{10}), 4.25 (2H, d, J = 6.0 Hz, H^{8}), 4.03 (2H, t, J = 8.0 Hz, H^{5}), 2.81 (1H, t, J = 3.0 Hz, H^{1}), 2.24 (2H, dt, J = 8.0 Hz 3.0 Hz, H^{3}), 1.74 (2H, quin, J = 8.0 Hz, H^{4}); ^{13}C NMR δ_C ppm (101 MHz, DMSO-d_6) 167.7 (C^{13}), 157.0 (C^{6}), 154.9 (C^{12}), 145.5 (C^{9}), 130.1 (C^{11}), 127.5 (C^{10}), 84.1 (C^{2}), 72.1 (C^{1}), 63.2 (C^{5}), 44.2 (C^{8}), 28.5 (C^{4}), 14.7 (C^{3}); *m/z* ($HRMS^{+}$) 262.1080 $[M+H]^{+}$ ($C_{14}H_{16}NO_4$ requires 262.1079);

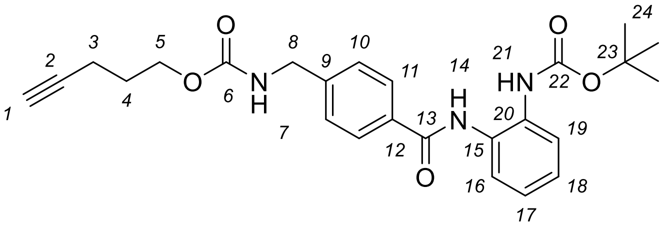

Pent-4-yn-1-yl (4-((2-((tert-butoxycarbonyl)amino)phenyl)carbamoyl)benzyl)carbamate (4b)

4-((((Pent-4-yn-1-yloxy)carbonyl)amino)methyl)benzoic acid (0.53 g, 2.01 mmol) and HATU (0.82 g, 2.16 mmol) were dissolved in anhydrous DMF (5 mL) and stirred at room temperature for 1 h. Tert-butyl N-(2-aminophenyl)carbamate (0.30 g, 1.44 mmol) and diisopropylethylamine (0.75 mL, 4.32 mmol) were added and the reaction stirred at room temperature for 16 h. The solvent was removed under reduced pressure and suspended in EtOAc (20 mL). The organic solution was washed with sat. $NaHCO_3$ (2 x 20 mL), brine (20 mL), the organic layers combined, dried over $MgSO_4$, filtered and the solvent removed under reduced pressure. The crude oil was purified by flash chromatography on silica (Hexane:EtOAc 40:60-50:50 gradient) to provide the *title compound* as a pale pink oil (0.79 g, 87%). ^{1}H NMR (400 MHz, $CDCl_3$) δ_H ppm 9.16 (1H, s, H^{14}), 7.91 (2H, d, J = 8.0 Hz, H^{11}), 7.77 (1H, d, J = 9.0 Hz, H^{16}), 7.35 (2H, d, J = 8.0 Hz, H^{10}), 7.23-7.12 (3H, m, H^{17-19}), 6.83 (1H, m, H^{21}), 5.14 (1H, bs,

H[7]), 4.41 (2H, d, J = 7.0 Hz, H[8]), 4.21 (2H, t, J = 6.5 Hz, H[5]), 2.28 (2H, m, H[3]), 1.96 (1H, t, J = 3.0 Hz, H[1]), 1.86 (2H, quin, J = 6.5 Hz, H[4]), 1.52 (9H, s, H[24]); ^{13}C NMR (101 MHz, $CDCl_3$) δ_C ppm 165.2 (C[13]), 156.5 (C[6]), 154.6 (C[22]), 142.6 (C[9]), 133.5 (C[12]), 130.9 (C[20]), 129.9 (C[15]), 127.8 (2C, C[11]), 127.5 (C[10]), 127.4-124.5 (C[17-19]), 125.7 (C[16]), 83.0 (C[2]), 81.7 (C[23]), 63.7 (C[5]), 44.6 (C[8]), 28.2 (C[24]), 27.9 (C[4]), 21.0 (C[1]), 15.2 (C[3]); *m/z* (HRMS$^+$) 452.2185 $[M+H]^+$ ($C_{25}H_{30}N_3O_5$ requires 452.2185); R_f = 0.37 (50:50 EtOAc:Hexane)

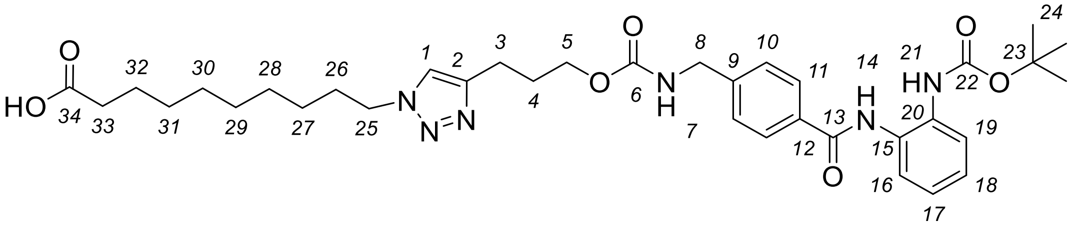

10-(4-(3-(((4-((2-((Tert-butoxycarbonyl)amino)phenyl)carbamoyl)benzyl)carbamoyl)oxy)propyl)-1H-1,2,3-triazol-1-yl)decanoic acid (6e)

Pent-4-yn-1-yl (4-((2-((tert-butoxycarbonyl)amino)phenyl)carbamoyl)benzyl)carbamate (0.22 g, 0.48 mmol), 10-azidodecanoic acid (0.12 g, 0.58 mmol) and CuI (0.02 g, 0.14 mmol) were suspended in degassed DMF:MeCN (5 mL, 2:1) under an inert atmosphere. 2,6-Lutidine (0.09 mL, 0.73 mmol) was added and the reaction stirred at room temperature for 16 h. The solvent was removed under reduced pressure and suspended in EtOAc (10 mL), washed with sat. $NaHCO_3$ (2 x 5 mL), brine (2 x 5 mL), the organic layers combined, dried over $MgSO_4$, filtered and the solvent removed under reduced pressure. The crude oil was purified by flash chromatography on silica (4-10% MeOH:DCM gradient) to provide the *title compound* as an off white solid (0.32 g, 54%). ^{1}H NMR (400 MHz, $CDCl_3$) δ_H ppm 9.21 (1H, s, H[14]), 7.90 (2H, d, J = 8.0 Hz, H[11]), 7.74 (1H, d, J = 7.5 Hz, H[16]), 7.34 (2H, d, J = 8.0 Hz, H[10]), 7.30 (1H, s, H[1]), 7.29-7.25 (1H, m, H[18]), 7.20-7.12 (2H, m, H[17, 19]), 7.00 (1H, bs, H[21]), 5.36 (1H, bs, H[7]), 4.40 (2H, d, J = 5.5 Hz, H[8]), 4.27 (2H, t, J = 7.0 Hz, H[25]), 4.12 (2H, t, J = 6.0 Hz, H[5]), 2.81-2.76 (2H, m, H[3]), 2.33-2.28 (2H, m, H[33]), 2.03-1.96 (2H, m, H[4]), 1.87-1.81 (2H, m, H[26]), 1.61-1.56 (2H, m, H[32]), 1.48 (9H, s, H[24]), 1.32-1.23 (10H, m, H[27-31]); ^{13}C NMR (101 MHz, $CDCl_3$) δ_C ppm 177.0 (C[34]), 165.6 (C[13]), 156.8 (C[6]), 154.8 (C[22]), 143.0 (C[9]), 142.8 (C[12]), 133.2 (C[2]), 130.8 (C[15]), 130.3 (C[20]), 127.8 (C[11]), 127.4 (C[10]), 125.9 (C[17]), 125.8 (C[19]), 125.7 (C[16]), 124.5 (C[18]), 120.9 (C[1]), 81.1 (C[23]), 64.1 (C[5]), 50.1 (C[25]), 44.5 (C[8]), 34.0 (C[33]), 30.2 (C[26]), 28.9-26.2 (C[27-31]), 28.3 (C[24]), 28.2 (C[4]), 24.6 (C[32]), 21.9 (C[3]); *m/z* (HRMS$^+$) 687.3451 $[M+Na]^+$ ($C_{35}H_{48}N_6O_7Na$ requires 687.3482); R_f = 0.23 (7% MeOH:DCM)

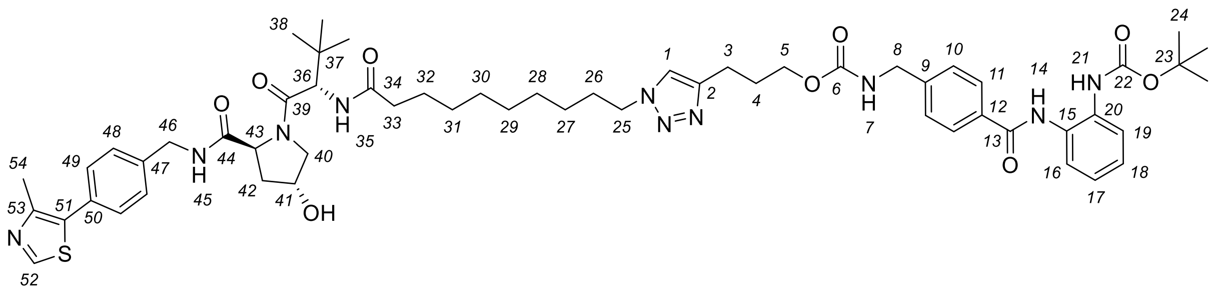

3-(1-(10-(((S)-1-((2S,4R)-4-Hydroxy-2-((4-(4-methylthiazol-5-yl)benzyl)carbamoyl)pyrrolidin-1-yl)-3,3-dimethyl-1-oxobutan-2-yl)amino)-10-oxodecyl)-1H-1,2,3-triazol-4-yl)propyl (4-((2-((tert-butoxy carbonyl)amino)phenyl)carbamoyl)benzyl)carbamate (8e)

10-(4-(3-(((4-((2-((Tert-butoxycarbonyl)amino)phenyl)carbamoyl)benzyl)carbamoyl)oxy)propyl)-1H-1,2,3-triazol-1-yl)decanoic acid (0.06 g, 0.09 mmol) and HATU (0.04 g, 0.11 mmol) and diisopropylethylamine (0.04 mL, 0.26 mmol) were dissolved in anhydrous DMF (1 mL), the reaction mixture cooled to 0 °C and stirred for 15 mins. A solution of (4*R*)-3-Methyl-L-valyl-4-hydroxy-*N*-[[4-(4-methyl-5-thiazolyl)phenyl]methyl]-L-prolinamide hydrochloride (0.04 g, 0.08 mmol) in anhydrous DMF (1 mL) was added slowly and the resulting solution was stirred at room temperature for 16 h. The solvent was removed under reduced pressure, redissolved in EtOAc (10 mL), washed with sat. $NaHCO_3$ (2 x 10 mL), brine (1 x 10 mL), organic layers combined, dried over $MgSO_4$, filtered and solvent removed under reduced pressure. The crude oil was purified using flash chromatography on silica (3-7% MeOH:DCM solvent gradient) to provide the *title compound* as a pale yellow oil (0.06 g, 57%). ^1H NMR (400 MHz, MeOD-d_4) δ_H ppm 8.86 (1H, s, H^{52}), 7.93 (2H, d, J = 8.5 Hz, H^{11}), 7.72 (1H, s, H^{1}), 7.60 (1H, d, J = 8.0 Hz, H^{16}), 7.46-7.38 (7H, m, $H^{10, 19, 48, 49}$), 7.24-7.17 (2H, m, $H^{17, 18}$), 4.64 (1H, s, H^{36}), 4.59-4.48 (3H, m, $H^{41, 43, 46}$), 4.37-4.30 (5H, m, $H^{8, 25, 46'}$), 4.11-4.08 (2H, m, H^{5}), 3.90-3.88 (1H, m, H^{40}), 3.80-3.76 (1H, m, $H^{40'}$), 2.78 (2H, m, H^{3}), 2.46 (3H, s, H^{54}), 2.30-2.17 (3H, m, $H^{33, 42}$), 2.10-2.03 (1H, m, $H^{42'}$), 2.02-1.96 (2H, m, H^{4}), 1.87-1.82 (2H, m, H^{26}), 1.61-1.54 (2H, m, H^{32}), 1.47 (9H, s, H^{24}), 1.32-1.24 (10H, m, $H^{27, 28, 29, 30, 31}$), 1.03 (9H, s, H^{38}); ^{13}C NMR (101 MHz, MeOD-d_4) δ_C ppm 174.6 (C^{34}), 173.0 (C^{44}), 170.9 (C^{39}), 166.3 (C^{13}), 157.8 (C^{6}), 154.8 (C^{22}), 151.48 (C^{52}), 147.6 (C^{53}), 146.7 (C^{2}), 144.0 (C^{9}), 138.9 (C^{47}), 132.0 (C^{50}), 131.6 (C^{20}), 130.2 (C^{15}), 130.1 (C^{51}), 129.0 (C^{49}), 127.5 (C^{10}), 127.4 (C^{11}), 127.0 (C^{48}), 126.0 (C^{16}), 125.9 (C^{12}), 125.7 (C^{17}), 124.8 (C^{18}), 124.1 (C^{19}), 121.88 (C^{1}), 80.2 (C^{23}), 69.7 (C^{41}), 63.6 (C^{5}), 59.4 (C^{43}), 57.6 (C^{36}), 56.6 (C^{40}), 49.8 (C^{25}), 43.7 (C^{8}), 42.4 (C^{46}), 37.5 (C^{42}), 35.2 (C^{33}), 29.8 (C^{26}), 28.6-25.7 ($C^{27, 28, 29, 30, 31}$), 28.5 (C^{4}), 27.2 (C^{24}) 26.0 (C^{32}), 25.6 (C^{38}), 25.5 (C^{37}), 21.5 (C^{3}), 14.5 (C^{54}); *m/z* ($HRMS^+$) 1099.5404 $[M+Na]^+$ ($C_{57}H_{76}N_{10}O_9SNa$ requires 1099.5415); R_f = 0.26 (10% MeOH:EtOAc).

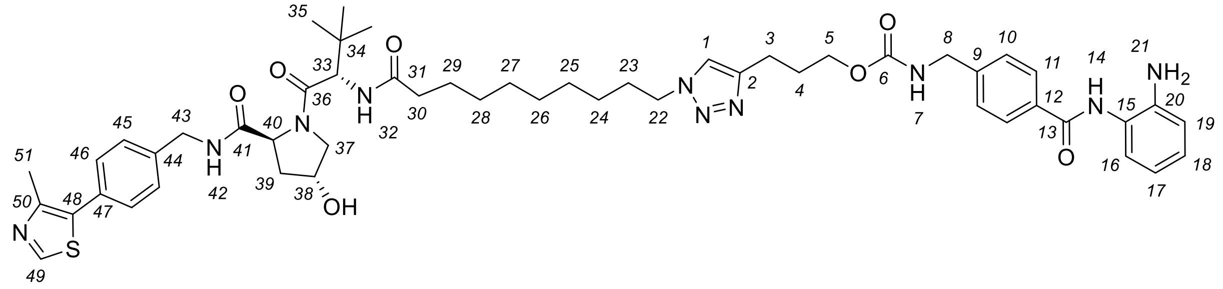

3-(1-(10-(((S)-1-((2S,4R)-4-Hydroxy-2-((4-(4-methylthiazol-5-yl)benzyl)carbamoyl)pyrrolidin-1-yl)-3,3-dimethyl-1-oxobutan-2-yl)amino)-10-oxodecyl)-1H-1,2,3-triazol-4-yl)propyl (4-((2-amino phenyl) carbamoyl)benzyl)carbamate (17)

TFA (0.2 mL) was added to a stirring solution of 3-(1-(10-(((*S*)-1-((2*S*,4*R*)-4-hydroxy-2-((4-(4-methylthiazol-5-yl)benzyl)carbamoyl)pyrrolidin-1-yl)-3,3-dimethyl-1-oxobutan-2-yl)amino)-10-oxo decyl)-1H-1,2,3-triazol-4-yl)propyl (4-((2-((tert-butoxycarbonyl)amino)phenyl)carbamoyl)benzyl) carbamate (0.06 g, 0.05 mmol) in DCM (2 mL) and the resulting reaction mixture was stirred at room temperature overnight. The reaction was concentrated under reduced pressure to afford a yellow oil. The crude oil was dissolved in MeOH (2 mL), agitated in MP-carbonate resin (3.02 mmol/g loading capacity, 310 mg) for 3 h and filtered. The crude product was lyophilised in H_2O:MeCN (3 mL, 2:1) to provide the *title compound* as a yellow powder (0.05 g, 92%). ^{1}H NMR (400 MHz, MeOD-d_4) δ_H ppm 8.75 (1H, s, H^{49}), 7.84 (2H, d, J = 8.0 Hz, H^{11}), 7.62 (1H, s, H^{1}), 7.35-7.28 (6H, m, $H^{10, 45, 46}$), 7.07 (1H, dd, J = 8.0 Hz 2.0 Hz, H^{16}), 6.96 (1H, ddd, J = 8.0 Hz 8.0 Hz 2.0 Hz, H^{18}), 6.79 (1H, dd, J = 8.0 Hz 2.0 Hz, H^{19}), 6.65 (1H, ddd, J = 8.0 Hz 8.0 Hz 2.0 Hz, H^{17}), 4.52 (1H, s, H^{33}), 4.48-4.37 (3H, m, $H^{38, 40, 43}$), 4.26-4.20 (5H, m, $H^{8, 22, 43'}$), 3.98 (2H, t, J = 6.5 Hz, H^{5}), 3.79-3.76 (1H, m, H^{37}), 3.69-3.66 (1H, m, $H^{37'}$), 2.67 (2H, t, J = 6.5 Hz, H^{3}), 2.35 (3H, s, H^{51}), 2.20-2.06 (3H, m, $H^{30, 39}$), 1.99-1.93 (1H, m, $H^{39'}$), 1.88 (2H, quin, J = 6.5 Hz, H^{4}), 1.77-1.71 (2H, m, H^{23}), 1.50-1.42 (2H, m, H^{29}), 1.21-1.15 (10H, m, $H^{24, 25, 26, 27, 28}$), 0.92 (9H, s, H^{35}); ^{13}C NMR (101 MHz, MeOD-d_4) δ_C ppm 174.4 (C^{31}), 173.0 (C^{41}), 170.9 (C^{36}), 167.3 (C^{13}), 157.8 (C^{6}), 151.6 (C^{49}), 147.6 (C^{50}), 146.9 (C^{2}), 143.6 (C^{9}), 142.3 (C^{20}), 138.8 (C^{44}), 132.9 (C^{47}), 132.0 (C^{48}), 130.1 (C^{12}), 127.7 (C^{11}), 127.6 (C^{46}), 127.5 (C^{10}), 127.1 (C^{18}), 126.9 (C^{45}), 126.2 (C^{16}), 123.9 (C^{15}), 121.9 (C^{1}), 118.2 (C^{17}), 117.3 (C^{19}), 69.6 (C^{38}), 63.6 (C^{5}), 59.4 (C^{40}), 57.6 (C^{33}), 56.6 (C^{37}), 49.7 (C^{22}), 43.7 (C^{8}), 42.3 (C^{43}), 37.5 (C^{39}), 36.2 (C^{34}), 35.2 (C^{30}), 29.8 (C^{23}), 29.0-26.0 ($C^{24, 25, 26, 27, 28}$), 28.5 (C^{4}), 25.7 (C^{35}), 25.5 (C^{29}), 21.2 (C^{3}), 14.4 (C^{51}); *m/z* (HRMS$^+$) 977.5063 $[M+H]^+$ ($C_{52}H_{69}N_{10}O_7S$ requires 977.5071).

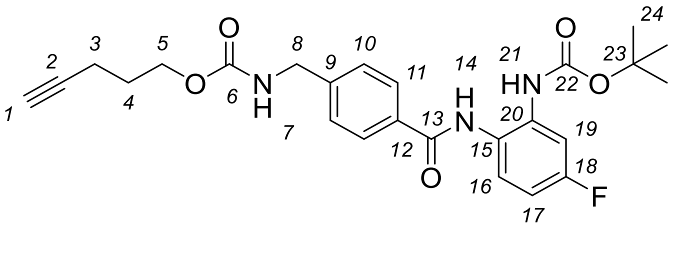

Pent-4-yn-1-yl(4-((2-((tert-butoxycarbonyl)amino)-4-fluorophenyl)carbamoyl)benzyl)carbamate (4b')

4-((((Pent-4-yn-1-yloxy)carbonyl)amino)methyl)benzoic acid (0.35 g, 1.33 mmol) and HATU (0.54 g, 1.43 mmol) were dissolved in anhydrous DMF (5 mL) and stirred at room temperature for 1 h. Tert-butyl N-(2-aminophenyl)carbamate Tert-butyl (2-amino-5-fluorophenyl)carbamate (0.21 g, 0.95 mmol) and diisopropylethylamine (0.50 mL, 2.86 mmol) were added and the reaction stirred at room temperature for 16 h. The solvent was removed under reduced pressure and suspended in EtOAc (20 mL). The organic solution was washed with sat. $NaHCO_3$ (2 x 20 mL), brine (20 mL), the organic layers combined, dried over $MgSO_4$, filtered and the solvent removed under reduced pressure. The crude oil was purified by flash chromatography on silica (EtOAc:Hexane 40:60-60:40 gradient) to provide the *title compound* as a pale pink oil (0.37 g, 84%). ^{1}H NMR (400 MHz, $CDCl_3$) δ_H ppm 8.84 (1H, s, H^{14}), 7.92 (2H, d, J = 8.0 Hz, H^{11}), 7.58 (1H, dd, J = 8.0 Hz, $^4J_{HF}$ 6.0 Hz, H^{16}), 7.40 (2H, d, J = 8.0 Hz, H^{10}), 7.20 (1H, dd, $^3J_{HF}$ 9.0 Hz, J = 2.0 Hz, H^{19}), 6.98 (1H, s, H^{21}), 6.90 (1H, ddd, J = 8.0 Hz 3.0 Hz, $^3J_{HF}$ 8.0 Hz, H^{17}), 5.14 (1H, bs, H^7), 4.45 (2H, J = 7.0 Hz, H^8), 4.24 (2H, t, J = 6.5 Hz, H^5), 2.31 (2H, m, H^3), 1.99 (1H, t, J = 2.5 Hz, H^1), 1.89 (2H, quin, J = 6.5 Hz, H^4), 1.53 (9H, s, H^{24}); ^{13}C NMR (101 MHz, $CDCl_3$) δ_C ppm 165.7 (C^{13}), 160.1 (d, $^1J_{CF}$ = 247 Hz, C^{18}), 156.6 (C^6), 142.9 (C^9), 133.8 (C^{22}), 133.0 (C^{12}), 132.4 (C^{15}), 127.8 (C^{11}), 127.5 (C^{10}), 127.3 (d, $^3J_{CF}$ = 8.3 Hz C^{16}), 125.8 (C^{20}), 112.2 (d, $^2J_{CF}$ = 22 Hz, H^{19}), 110.8 (d, $^2J_{CF}$ = 27 Hz, H^{17}), 82.6 (C^2), 81.6 (C^{23}), 63.8 (C^5), 44.3 (C^8), 28.3 (C^{24}), 27.9 (C^4), 21.1 (C^1), 15.3 (C^3); ^{19}F NMR (376 MHz, $CDCl_3$) δ_F ppm -114.6 (1F, F^{18}); *m/z* (HRMS$^+$) 492.1902 $[M+Na]^+$ ($C_{25}H_{28}FN_3O_5Na$ requires 492.1911); R_f = 0.38 (50:50 EtOAc:Hexane).

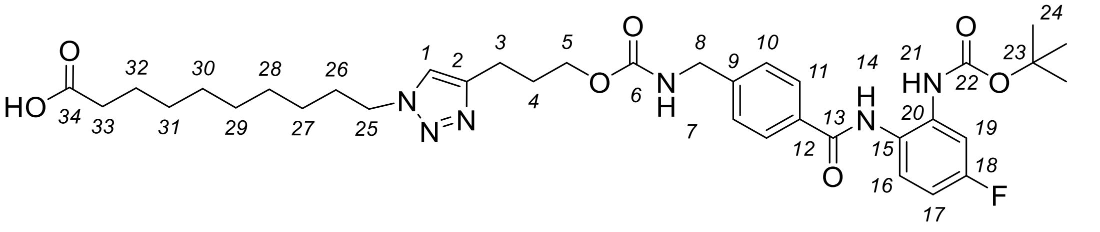

10-(4-(3-(((4-((2-((Tert-butoxycarbonyl)amino)-4-fluorophenyl)carbamoyl)benzyl)carbamoyl)oxy)-propyl)-1H-1,2,3-triazol-1-yl)decanoic acid (6e')

Pent-4-yn-1-yl(4-((2-((tert-butoxycarbonyl)amino)-4-fluorophenyl)carbamoyl)benzyl)carbamate (0.25 g, 0.53 mmol) and CuI (0.02 g, 0.16 mmol) were suspended in degassed DMF:MeCN (5 mL, 2:1) under an inert atmosphere. 2,6-Lutidine (0.09 mL, 0.80 mmol) was added and the reaction stirred at room temperature for 16 h. The solvent was removed under reduced pressure and suspended in EtOAc (10 mL), washed with sat. $NaHCO_3$ (2 x 5 mL), brine (2 x 5 mL), the organic layers combined, dried over $MgSO_4$, filtered and the solvent removed under reduced pressure. The crude oil was purified by flash chromatography on silica (3-10%MeOH:DCM gradient) to provide the *title compound* as an off white solid (0.18 g, 75%). ^{1}H NMR (400 MHz, $CDCl_3$) δ_H ppm 8.99 (1H, bs, H^{14}), 7.91 (2H, d, J = 8.0

Hz, H^{11}), 7.55-7.52 (1H, m, H^{16}), 7.37 (2H, d, J = 8.0 Hz, H^{10}), 7.33 (1H, s, H^{1}), 7.26-7.23 (1H, m, H^{19}), 6.88-6.84 (1H, m, H^{17}), 6.90 (1H, bs, H^{21}), 5.45 (1H, bs, H^{7}), 4.43 (2H, d, J = 6.0 Hz, H^{8}), 4.31 (2H, J = 7.0 Hz, H^{25}), 4.15 (2H, t, J = 7.0 Hz, H^{5}), 2.80 (2H, m, H^{3}), 2.32 (2H, t, J = 6.0 Hz, H^{33}), 2.04-1.98 (2H, m, H^{4}), 1.89-1.85 (2H, m, H^{26}), 1.64-1.57 (2H, m, H^{32}), 1.50 (9H, s, H^{24}), 1.33-1.25 (10H, m, H$^{27-31}$); ^{13}C NMR (101 MHz, $CDCl_3$) δ_C ppm 177.9 (C^{34}), 165.7 (C^{13}), 161.0 (d, $^{1}J_{CF}$ 280 Hz, C^{18}), 156.9 (C^{6}), 152.1 (C^{22}), 147.4 (C^{2}), 143.3 (C^{12}), 132.7 (C^{9}), 132.0 (C^{15}), 127.8 (C^{11}), 127.4 (C^{10}), 127.3 (d, $^{3}J_{CF}$ 40 Hz, C^{16}), 121.0 (C^{20}), 120.8 (C^{1}), 111.8 (d, $^{2}J_{CF}$ 100 Hz, C^{17}), 110.6 (d, $^{2}J_{CF}$ 84 Hz, C^{19}), 81.3 (C^{23}), 64.1 (C^{5}), 50.0 (C^{25}), 44.6 (C^{8}), 33.9 (C^{33}), 30.1 (C^{26}), 28.8-26.2 (C$^{27-31}$), 28.6 (C^{4}), 28.2 (C^{24}), 24.7 (C^{32}), 22.0 (C^{3}); ^{19}F NMR (376 MHz, $CDCl_3$) δ_F ppm -133.2 (1F, F^{18}); *m/z* (HRMS$^+$) 683.3568 $[M+H]^+$ ($C_{35}H_{48}FN_6O_7$ requires 683.3569); R_f = 0.27 (7% MeOH:DCM).

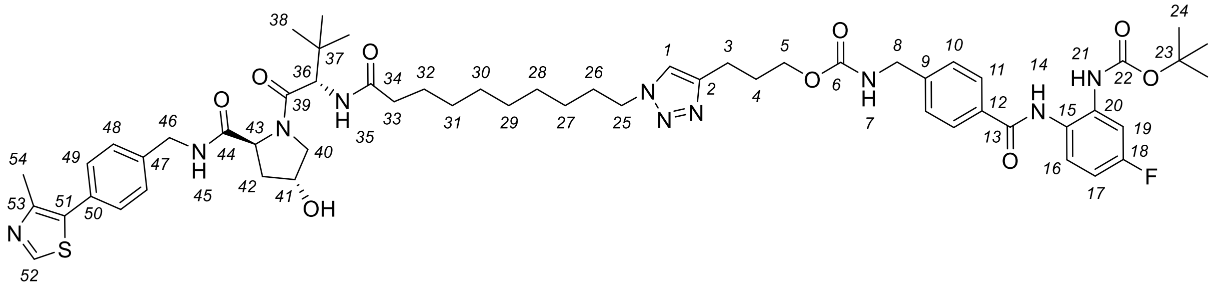

3-(1-(10-(((S)-1-((2S,4R)-4-Hydroxy-2-((4-(4-methylthiazol-5-yl)benzyl)carbamoyl)pyrrolidin-1-yl)-3,3-dimethyl-1-oxobutan-2-yl)amino)-10-oxodecyl)-1H-1,2,3-triazol-4-yl)propyl(4-((2-((tert-butoxy carbonyl)amino)-4-fluorophenyl)carbamoyl)benzyl)carbamate (8e')

10-(4-(3-(((4-((2-((Tert-butoxycarbonyl)amino)-4-fluorophenyl)carbamoyl)benzyl)carbamoyl)oxy)-propyl)-1H-1,2,3-triazol-1-yl)decanoic acid (0.07 g, 0.09 mmol) and HATU (0.04 g, 0.11 mmol) and diisopropylethylamine (0.05 mL, 0.26 mmol) were dissolved in anhydrous DMF (1 mL), the reaction mixture cooled to 0 °C and stirred for 15 mins. A solution of (4*R*)-3-Methyl-L-valyl-4-hydroxy-*N*-[[4-(4-methyl-5-thiazolyl)phenyl]methyl]-L-prolinamide hydrochloride (0.04 g, 0.08 mmol) in anhydrous DMF (1 mL) was added slowly and the resulting solution was stirred at room temperature for 16 h. The solvent was removed under reduced pressure, redissolved in EtOAc (10 mL), washed with sat. $NaHCO_3$ (2 x 10 mL), brine (1 x 10 mL), organic layers combined, dried over $MgSO_4$, filtered and solvent removed under reduced pressure. The crude oil was purified using flash chromatography on silica (2-10% MeOH:DCM solvent gradient) to provide the *title compound* as a pale yellow oil (0.06 g, 58%). ^{1}H NMR (400 MHz, MeOD-d_4) δ_H ppm 8.75 (1H, s, H^{52}), 7.81 (2H, d, J = 8.5 Hz, H^{11}), 7.61 (1H, s, H^{1}), 7.38-7.27 (8H, m, H$^{10, 16, 19, 48, 49}$), 6.79 (1H, ddd, J = 8.0 Hz 3.0 Hz, $^{3}J_{HF}$ 8.5 Hz, H^{17}), 4.52 (1H, s, H^{36}), 4.48-4.36 (3H, m, H$^{41, 43, 46}$), 4.26-4.18 (5H, m, H$^{8, 25, 46'}$), 3.98 (2H, t, J = 7.0 Hz, H^{5}), 3.80-3.77 (1H, m, H^{40}), 3.70-3.66 (1H, m, H$^{40'}$), 2.68 (2H, t, J = 7.0 Hz, H^{3}), 2.35 (3H, s, H^{54}), 2.20-2.07 (3H, m, H$^{33, 42}$), 2.00-1.93 (1H, m, H$^{42'}$), 1.88 (2H, quin, J = 7.0 Hz, H^{4}), 1.76-1.70 (2H, m, H^{26}), 1.50-1.43 (2H, m, H^{32}), 1.37 (9H, s, H^{24}), 1.22-1.13 (10H, m, H$^{27, 28, 29, 30, 31}$), 0.92 (9H, s, H^{38}); ^{13}C NMR (101 MHz,

MeOD-d_4) δ_C ppm 174.7 (C^{34}), 173.1 (C^{44}), 170.9 (C^{39}), 167.2 (C^{13}), 160.7 (d, $^1J_{CF}$ 241 Hz, C^{18}), 157.8 (C^6), 154.0 (C^{22}), 151.4 (C^{52}), 147.6 (C^{53}), 146.7 (C^2), 144.0 (C^9), 138.9 (C^{12}), 138.6 (C^{47}), 132.0 (C^{51}), 128.9 (C^{48}), 127.7 (d, $^3J_{CF}$ 9.4 Hz, C^{16}), 127.6 (C^{11}), 127.5 (C^{10}), 127.4 (C^{49}), 127.0 (C^{50}), 121.8 (C^1), 110.6 (d, $^4J_{CF}$ 10 Hz, C^{15}), 110.5 (d, $^2J_{CF}$ 23 Hz, C^{17}), 109.6 (d, $^3J_{CF}$ 28 z, H^{20}), 109.5 (d, $^2J_{CF}$ 30 Hz, H^{19}), 80.6 (C^{23}), 69.7 (C^{41}), 63.6 (C^5), 59.4 (C^{43}), 57.6 (C^{36}), 56.6 (C^{40}), 49.9 (C^{25}), 43.8 (C^8), 42.4 (C^{46}), 37.6 (C^{42}), 35.2 (C^{33}), 35.0 (C^{37}), 29.8 (C^{26}), 28.9-26.0 ($C^{27, 28, 29, 30, 31}$), 28.5 (C^4), 27.2 (C^{24}), 25.9 (C^{38}), 25.5 (C^{32}), 21.4 (C^3), 14.5 (C^{54}); ^{19}F NMR (376 MHz, $CDCl_3$) δ_F ppm -115.4 (1F, F^{18}); *m/z* (HRMS$^+$) 1095.5466 $[M+H]^+$ ($C_{57}H_{76}FN_{10}O_9S$ requires 1095.5501); R_f = 0.32 (10% MeOH:EtOAc).

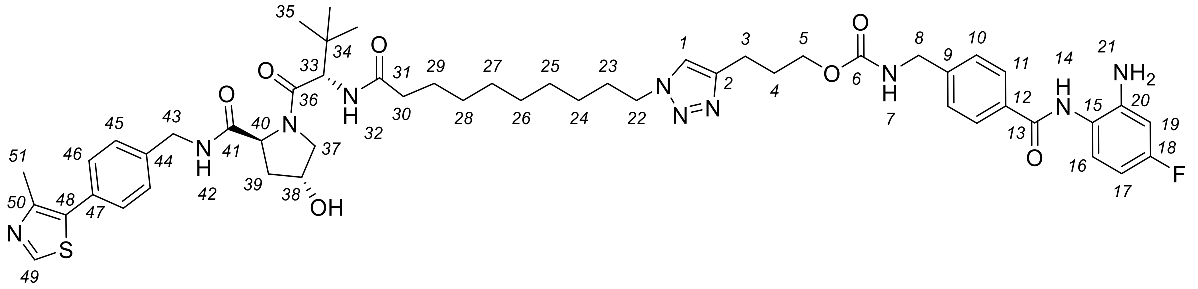

3-(1-(10-(((S)-1-((2S,4R)-4-Hydroxy-2-((4-(4-methylthiazol-5-yl)benzyl)carbamoyl)pyrrolidin-1-yl)-3,3-dimethyl-1-oxobutan-2-yl)amino)-10-oxodecyl)-1H-1,2,3-triazol-4-yl)propyl(4-((2-amino-4-fluoro phenyl)carbamoyl)benzyl)carbamate 18

TFA (0.2 mL) was added to a stirring solution of 3-(1-(10-(((*S*)-1-((2*S*,4*R*)-4-hydroxy-2-((4-(4-methylthiazol-5-yl)benzyl)carbamoyl)pyrrolidin-1-yl)-3,3-dimethyl-1-oxobutan-2-yl)amino)-10-oxo decyl)-1H-1,2,3-triazol-4-yl)propyl(4-((2-((tert-butoxycarbonyl)amino)-4-fluorophenyl)carbamoyl) benzyl)carbamate (0.06 g, 0.05 mmol) in DCM (2 mL) and the resulting reaction mixture was stirred at room temperature overnight. The reaction was concentrated under reduced pressure to afford a yellow oil. The crude oil was dissolved in MeOH (2 mL), agitated in MP-carbonate resin (3.02 mmol/g loading capacity, 300 mg) for 3 h and filtered. The crude product was lyophilised in H_2O:MeCN (3 mL, 2:1) to provide the *title compound* as a yellow powder (0.05 g, 92%). ^{1}H NMR (400 MHz, MeOD-d_4) δ_H ppm 8.76 (1H, s, H^{49}), 7.83 (2H, d, J = 9.0 Hz, H^{11}), 7.62 (1H, s, H^1), 7.35-7.27 (6H, m, $H^{10, 45, 46}$), 7.00 (1H, dd, J = 8.5 Hz, $^4J_{HF}$ 8.0 Hz, H^{16}), 6.47 (1H, dd, J = 2.5 Hz, ^3J = 10 Hz, H^{19}), 6.30 (1H, ddd, J = 8.5 Hz 3.0 Hz, $^3J_{HF}$ 8.5 Hz, H^{17}), 4.52 (1H, s, H^{33}), 4.48-4.36 (3H, m, $H^{38, 40, 43}$), 4.26-4.19 (5H, m, $H^{8, 22, 43'}$), 3.98 (2H, t, J = 6.0 Hz, H^5), 3.80-3.77 (1H, m, H^{37}), 3.70-3.66 (1H, m, $H^{37'}$), 2.67 (2H, t, J = 6.0 Hz, H^3), 2.35 (3H, s, H^{51}), 2.20-2.08 (3H, m, $H^{30, 39}$), 1.99-1.93 (1H, m, $H^{39'}$), 1.87 (2H, quin, J = 6.0 Hz, H^4), 1.77-1.74 (2H, m, H^{23}), 1.48-1.43 (2H, m, H^{29}), 1.19-1.14 (10H, m, $H^{24, 25, 26, 27, 28}$), 0.91 (9H, s, H^{35}); ^{13}C NMR (101 MHz, MeOD-d_4) δ_C ppm 174.7 (C^{31}), 173.3 (C^{41}), 171.0 (C^{36}), 167.5 (C^{13}), 162.3 ($^1J_{CF}$ 240 Hz, C^{18}), 157.8 (C^6), 151.5 (C^{49}), 147.6 (C^{50}), 146.6 (C^2), 145.1 (C^{20}), 143.7 (C^9), 138.9 (C^{12}), 138.8 (C^{47}), 132.0 (C^{44}), 128.9 (C^{10}), 128.2 ($^4J_{CF}$ = 10 Hz, C^{16}), 127.6 (C^{11}), 127.5 (C^{45}), 127.0 (C^{48}), 126.9 (C^{46}), 121.8 (C^1), 119.2 (C^{20}), 102.5 ($^3J_{CF}$ 26 Hz, C^{19}), 102.3 ($^3J_{CF}$ 30 Hz, C^{17}), 69.7 (C^{38}), 63.7

(C^{5}), 59.4 (C^{40}), 57.5 (C^{33}), 56.6 (C^{37}), 49.9 (C^{22}), 43.6 (C^{8}), 42.4 (C^{43}), 37.6 (C^{39}), 35.4 (C^{30}), 35.2 (C^{34}), 29.8 (C^{23}), 28.9-26.0 ($C^{24, 25, 26, 27, 28}$), 28.5 (C^{4}), 25.6 (C^{35}), 25.5 (C^{29}), 21.5 (C^{3}), 14.5 (C^{51}); ^{19}F NMR (376 MHz, $CDCl_3$) δ_F ppm -118.3 (1F, F^{18}); *m/z* ($HRMS^+$) 995.4973 $[M+H]^+$ ($C_{52}H_{68}FN_{10}O_7S$ requires 995.4977).

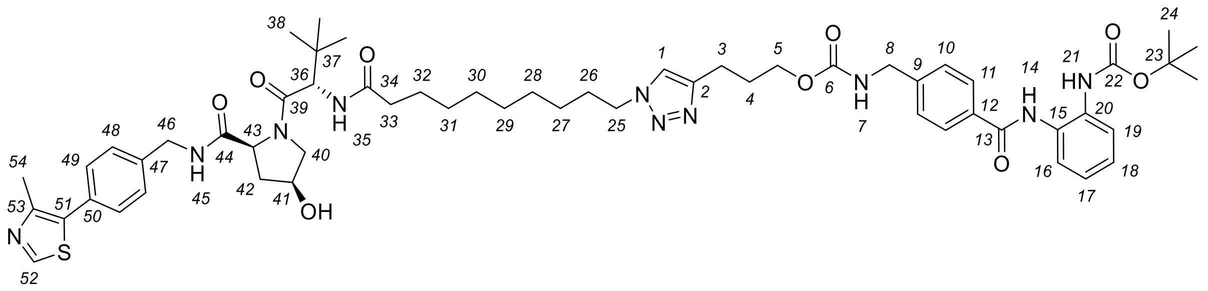

3-(1-(10-(((S)-1-((2S,4S)-4-Hydroxy-2-((4-(4-methylthiazol-5-yl)benzyl)carbamoyl)pyrrolidin-1-yl)-3,3-dimethyl-1-oxobutan-2-yl)amino)-10-oxodecyl)-1H-1,2,3-triazol-4-yl)propyl (4-((2-((tert-butoxy carbonyl)amino)phenyl)carbamoyl)benzyl)carbamate

10-(4-(3-(((4-((2-((Tert-butoxycarbonyl)amino)phenyl)carbamoyl)benzyl)carbamoyl)oxy)propyl)-1H-1,2,3-triazol-1-yl)decanoic acid (0.06 g, 0.08 mmol) and HATU (0.05 g, 0.12 mmol) were dissolved in DMF (anhydrous, 1 mL) and stirred at room temperature for 30 min. (2*S*,4*S*)-1-((*S*)-2-Amino-3,3-dimethylbutanoyl)-4-hydroxy-N-(4-(4-methylthiazol-5-yl)benzyl)pyrrolidine-2-carboxamide (0.04 g, 0.09 mmol) was dissolved in DMF (anhydrous, 2 mL) and added to the stirring mixture along with diisopropylethylamine (0.04 mL, 0.23 mmol) and the reaction stirred for 16 h at room temperature. The solvent was removed under reduced pressure and the crude dissolved in EtOAc (10 mL), washed with sat. NaHCO3 (2 x 10 mL), brine (2 x 10 mL) the organic layers were combined, dried over MgSO4, filtered and the solvent was removed under reduced pressure. The crude was purified on silica using a Biotage Isolera system (3-10% MeOH:EtOAc) to provide the *title compound* as an clear oil (0.04 g, 40%). ^{1}H NMR (400 MHz, MeOD-d_4) δ_H ppm 8.87 (1H, s, H^{52}), 7.94 (2H, d, *J* = 8.0 Hz, H^{11}), 7.73 (1H, s, H^{1}), 7.62 (1H, m, H^{16}), 7.46-7.39 (7H, m, $H^{10, 19, 48, 49}$), 7.25-7.18 (2H, m, $H^{17, 18}$), 4.56-4.50 (3H, m, $H^{36, 43, 46}$), 4.40-4.30 (6H, m, $H^{8, 25, 41, 46'}$), 4.11 (2H, t, *J* = 6.5 Hz, H^{5}), 4.06-4.02 (1H, m, H^{40}), 3.73-3.70 (1H, m, $H^{40'}$), 2.79 (2H, t, *J* = 6.5 Hz, H^{3}), 2.47 (3H, s, H^{54}), 2.45-2.37 (1H, m, H^{42}), 2.35-2.21 (2H, m, H^{33}), 2.05-1.96 (3H, m, $H^{4, 42'}$), 1.89-1.82 (2H, m, H^{26}), 1.61-1.54 (2H, m, H^{32}), 1.49 (9H, s, H^{24}), 1.33-1.25 (10H, m, $H^{27, 28, 29, 30, 31}$), 1.04 (9H, s, H^{38}); ^{13}C NMR (101 MHz, MeOD-d_4) 175.0 (C^{34}), 173.5 (C^{44}), 171.2 (C^{39}), 166.6 (C^{13}), 157.8 (C^{6}), 154.9 (C^{22}), 151.4 (C^{52}), 147.6 (C^{53}), 146.7 (C^{2}), 143.9 (C^{9}), 138.5 (C^{47}), 132.8 (C^{50}), 131.6 (C^{20}), 130.2 (C^{15}), 130.1 (C^{51}), 128.9 (C^{49}), 127.6 (C^{10}), 127.5 (C^{11}), 127.0 (C^{48}), 126.0 (C^{16}), 125.7 (C^{12}),121.8 (C^{1}), 125.6 (C^{17}), 124.8 (C^{18}), 124.2 (C^{19}), 80.3 (C^{23}), 70.2 (C^{41}), 63.7 (C^{5}), 59.6 (C^{43}), 57.9 (C^{36}), 56.3 (C^{40}), 49.9 (C^{25}), 43.7 (C^{8}), 42.5 (C^{46}), 36.5 (C^{42}), 35.1 (C^{33}), 34.6 (C^{37}), 29.8 (C^{26}), 28.9-26.1 ($C^{27, 28, 29, 30, 31}$), 28.5 (C^{4}), 27.3 (C^{24}), 25.6 (C^{38}), 25.5 (C^{32}), 21.3 (C^{3}), 14.4 (C^{54}); *m/z* ($HRMS^+$) 1077.5591 $[M+H]^+$ ($C_{57}H_{77}N_{10}O_9S$ requires 1077.5596).

(HRMS) m/z: for $C_{57}H_{77}N_{10}O_9S$, $[M+H]^+$ Calc. Mass = 1077.5596, found 1077.5591. For $C_{57}H_{77}N_{10}O_9SNa$, $[M+Na]^+$, Calc. Mass = 1099.5415, found 1099.5385.

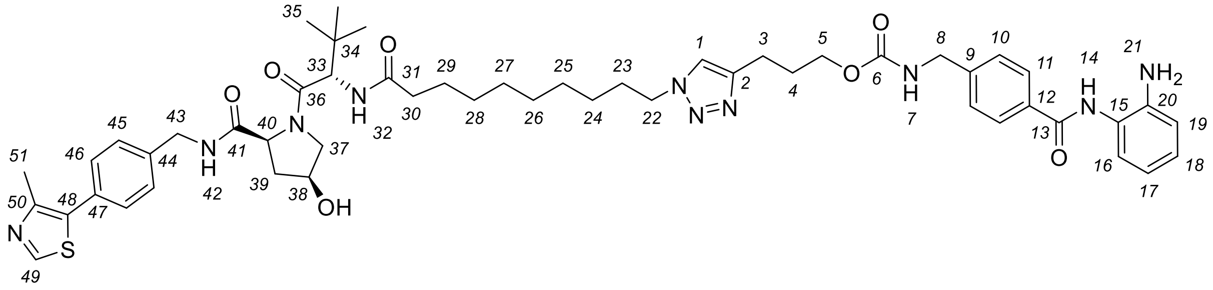

3-(1-(10-(((S)-1-((2S,4S)-4-Hydroxy-2-((4-(4-methylthiazol-5-yl)benzyl)carbamoyl)pyrrolidin-1-yl)-3,3-dimethyl-1-oxobutan-2-yl)amino)-10-oxodecyl)-1H-1,2,3-triazol-4-yl)propyl (4-((2-aminophenyl) carbamoyl)benzyl)carbamate (22)

TFA (0.2 mL) was added to a stirring solution of 3-(1-(10-(((*S*)-1-((2*S*,4*S*)-4-hydroxy-2-((4-(4-methylthiazol-5-yl)benzyl)carbamoyl)pyrrolidin-1-yl)-3,3-dimethyl-1-oxobutan-2-yl)amino)-10-oxo decyl)-1H-1,2,3-triazol-4-yl)propyl(4-((2-((tert-butoxycarbonyl)amino)phenyl)carbamoyl)benzyl) carbamate (0.04 g, 0.03 mmol) in DCM (2 mL) and mixture stirred at room temperature for 3 h. The reaction was concentrated under reduced pressure, dissolved in MeOH (2 mL) and agitated in MP-carbonate resin (3.02 mmol/g loading capacity, 200 mg) for 2 h. The crude was lyophilised in H_2O:MeCN (2 mL, 1:1) to afford the *title compound* as a white solid (0.02g, 55%). ^{1}H NMR (400 MHz, MeOD-d_4) δ_H ppm 8.76 (1H, s, H^{49}), 7.84 (2H, d, *J* = 8.0 Hz, H^{11}), 7.62 (1H, s, H^1), 7.35-7.28 (6H, m, $H^{10, 45, 46}$), 7.08 (1H, dd, *J* = 8.0 Hz 2.0 Hz, H^{16}), 6.96 (1H, ddd, *J* = 8.0 Hz 8.0 Hz, 2.0 Hz, H^{18}), 6.79 (1H, dd, *J* = 8.0 Hz 2.0 Hz, H^{19}), 6.65 (1H, m, H^{17}), 4.45-4.37 (3H, m, $H^{33, 40, 43}$), 4.28-4.21 (6H, m, $H^{8, 22, 38, 43'}$), 3.99 (2H, t, *J* = 6.5 Hz, H^5), 3.93-3.90 (1H, m, H^{37}), 3.60-3.56 (1H, m, $H^{37'}$), 2.68 (2H, t, *J* = 6.5 Hz, H^3), 2.36 (3H, s, H^{51}), 2.33-2.25 (1H, m, H^{39}), 2.21-2.07 (2H, m, H^{30}), 1.91-1.84 (3H, m, $H^{4, 39'}$), 1.79-1.73 (2H, m, H^{23}), 1.49-1.43 (2H, m, H^{29}), 1.21-1.15 (10H, m, $H^{24, 25, 26, 27, 28}$), 0.92 (9H, s, H^{35}); ^{13}C NMR (101 MHz, MeOD-d_4) 175.0 (C^{31}), 173.5 (C^{41}), 171.2 (C^{36}), 167.2 (C^{13}), 157.9 (C^6), 151.4 (C^{49}), 147.7 (C^{50}), 146.9 (C^2), 143.6 (C^9), 142.4 (C^{20}), 138.9 (C^{44}), 132.9 (C^{47}), 132.0 (C^{12}), 130.1 (C^{48}), 129.0 (C^{46}), 127.7 (C^{45}), 127.6 (C^{11}), 127.1 (C^{18}), 126.9 (C^{10}), 126.2 (C^{16}), 123.9 (C^{15}), 121.8 (C^1), 118.2 (C^{17}), 117.3 (C^{19}), 70.1 (C^{38}), 63.6 (C^5), 59.6 (C^{40}), 57.9 (C^{33}), 56.1 (C^{37}), 49.8 (C^{22}), 43.7 (C^8), 42.3 (C^{43}), 36.5 (C^{39}), 35.0 (C^{30}), 34.5 (C^{34}), 29.8 (C^{23}), 28.9-26.0 ($C^{24, 25, 26, 27, 28}$), 28.5 (C^4), 25.6 (C^{35}), 25.5 (C^{29}), 21.3 (C^3), 14.5 (C^{51}); *m/z* (HRMS$^+$) 977.5065 $[M+H]^+$ ($C_{52}H_{69}N_{10}O_7S$ requires 977.5071).

References

1 Y. Song, L. Dagil, L. Fairall, N. Robertson, M. Wu, T.J. Ragan, C.G. Savva, A. Saleh, N. Morone, M.B.A. Kunze, A.G. Jamieson, P.A. Cole, D.F. Hansen, and J.W.R. Schwabe, *Cell Reports* 2020, **30**, 2699-2711.

2 P. Watson, L. Fairall, G. Santos, and J.W.R. Schwabe, *Nature* 2012, **481** (7381), 335–340.

3 J.P. Smalley, I.M. Baker, W.A. Pytel, L.Y. Lin, K.J. Bowman, J. W.R. Schwabe, S.M. Cowley and J.T. Hodgkinson, *J. Med. Chem.,* 2022, **65**, 5642.

4 X. Wang, X. Jiang, S. Sun and Y. Liu, *RSC Adv.*, 2018, **8**, 16494–16502.

5 S. R. Nagarajan, B. Devadas, M. E. Zupec, S. K. Freeman, D. L. Brown, H. F. Lu, P. P. Mehta, N. S. Kishore, C. A. McWherter, D. P. Getman, J. I. Gordon and J. A. Sikorski, *J. Med. Chem.*, 1997, **40**, 1422–1438.

Appendix: ^{1}H NMR and ^{13}C NMR of Novel Compounds

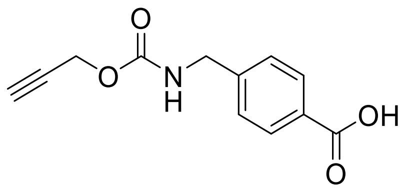

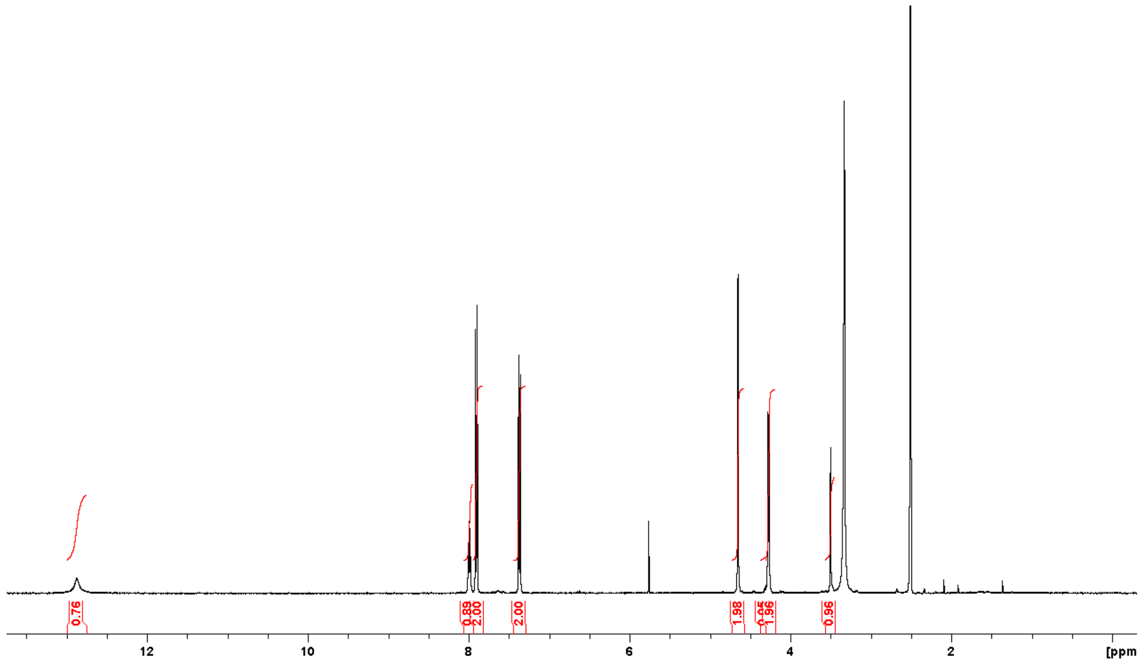

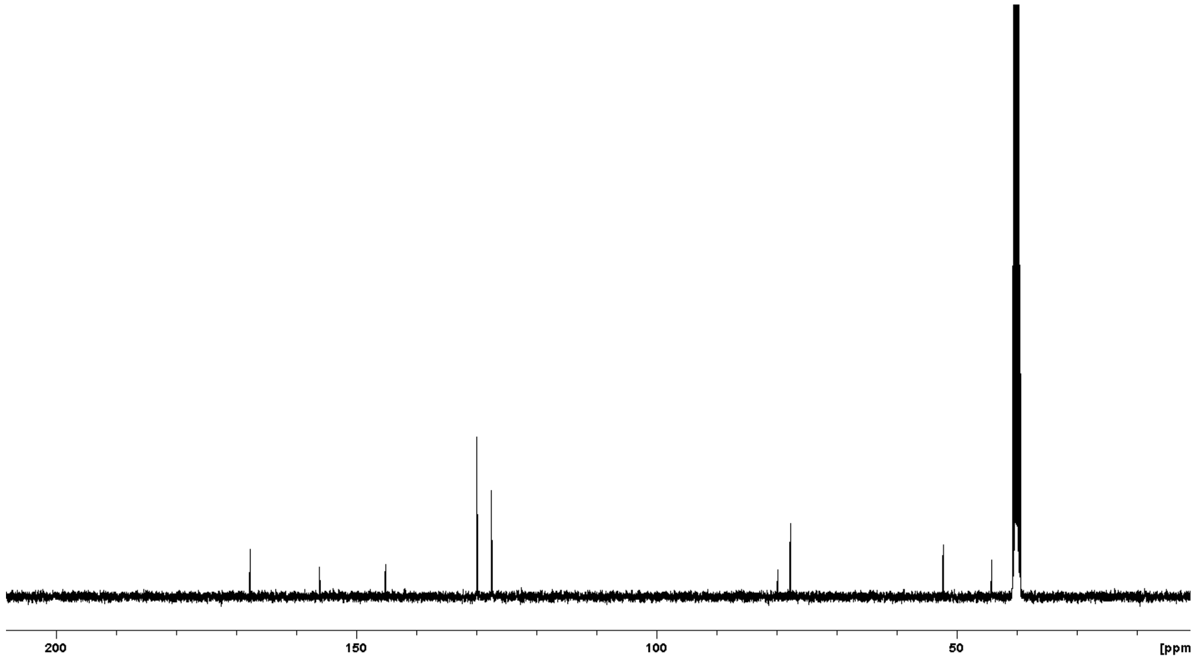

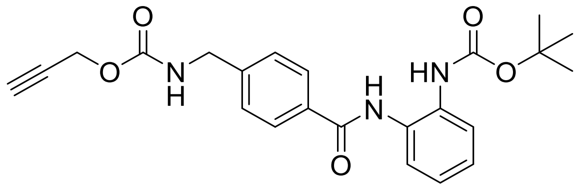

O
N
H
O
HN
O
H
N
O

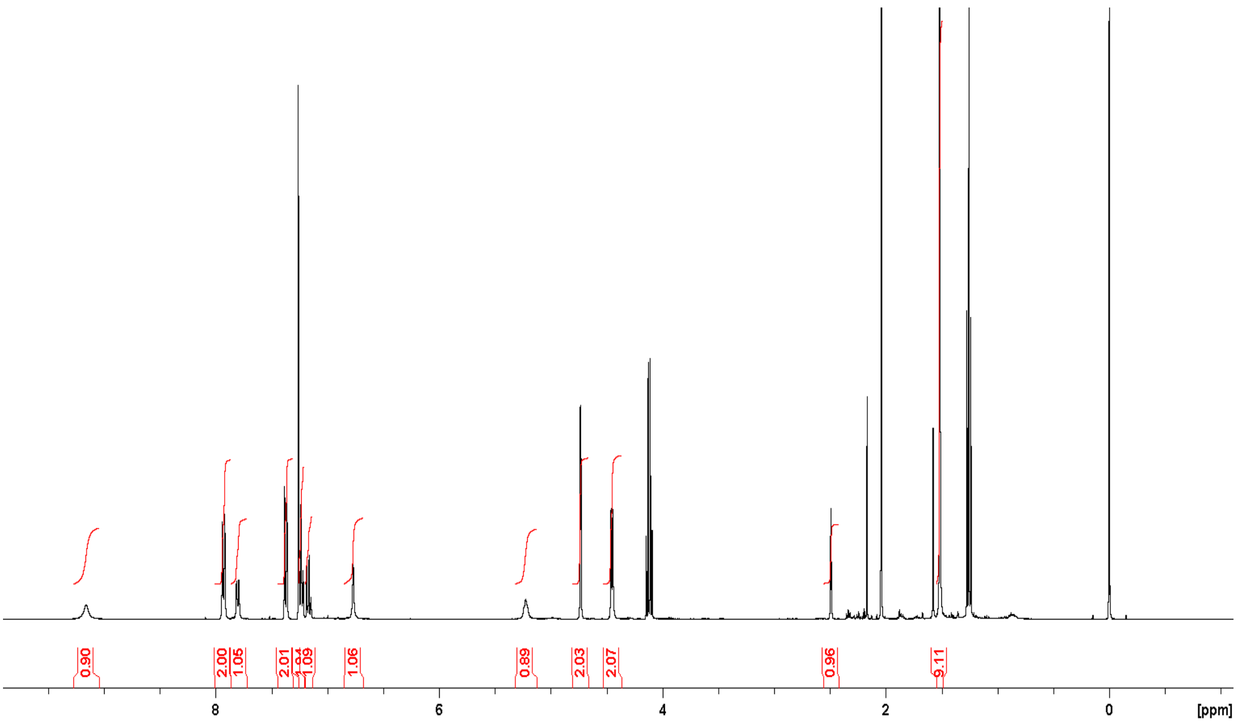

0.90
2.00
1.05
2.01
1.09
1.06
0.89
2.03
2.07
0.96
9.11
8
6
4
2
0
[ppm]

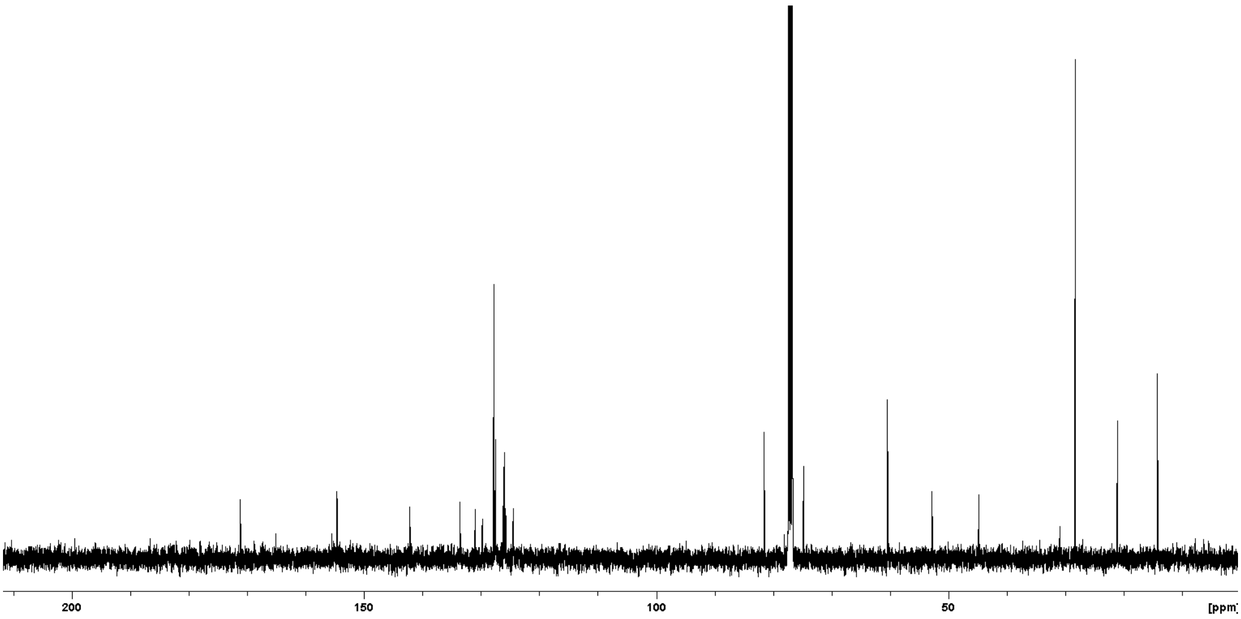

200
150
100
50
[ppm]

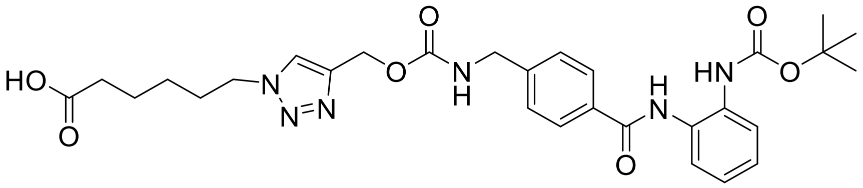

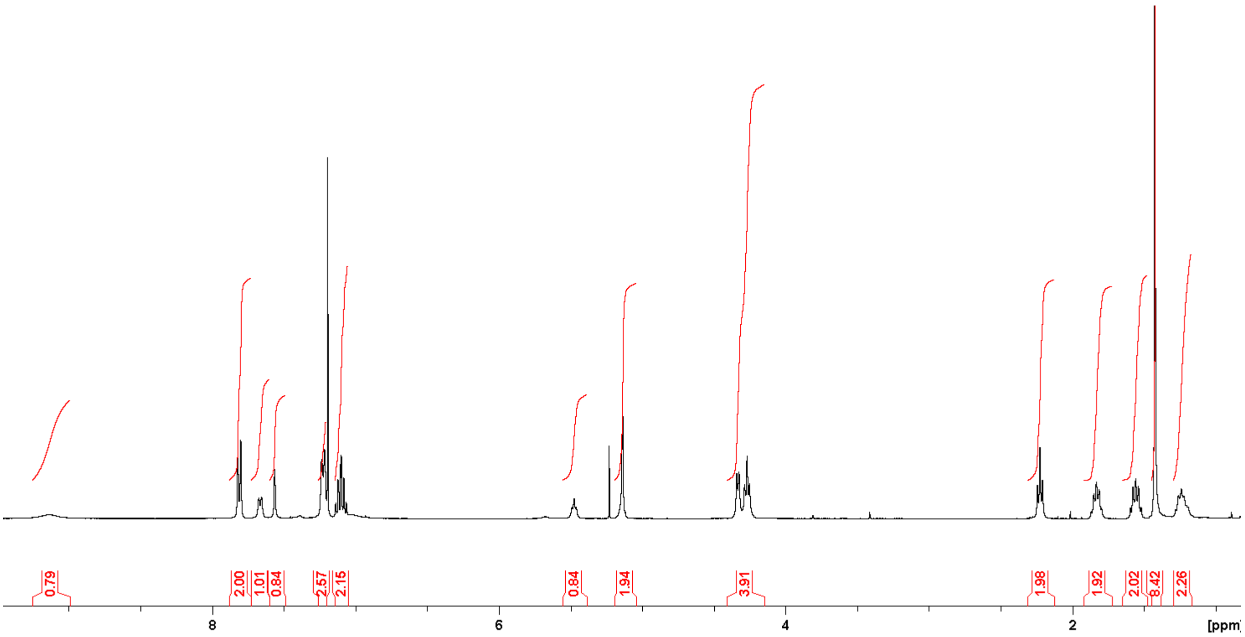
0.79
2.00
1.01
0.84
2.57
2.15
0.84
1.94
3.91
1.98
1.92
2.02
8.42
2.26
8
6
4
2
[ppm]

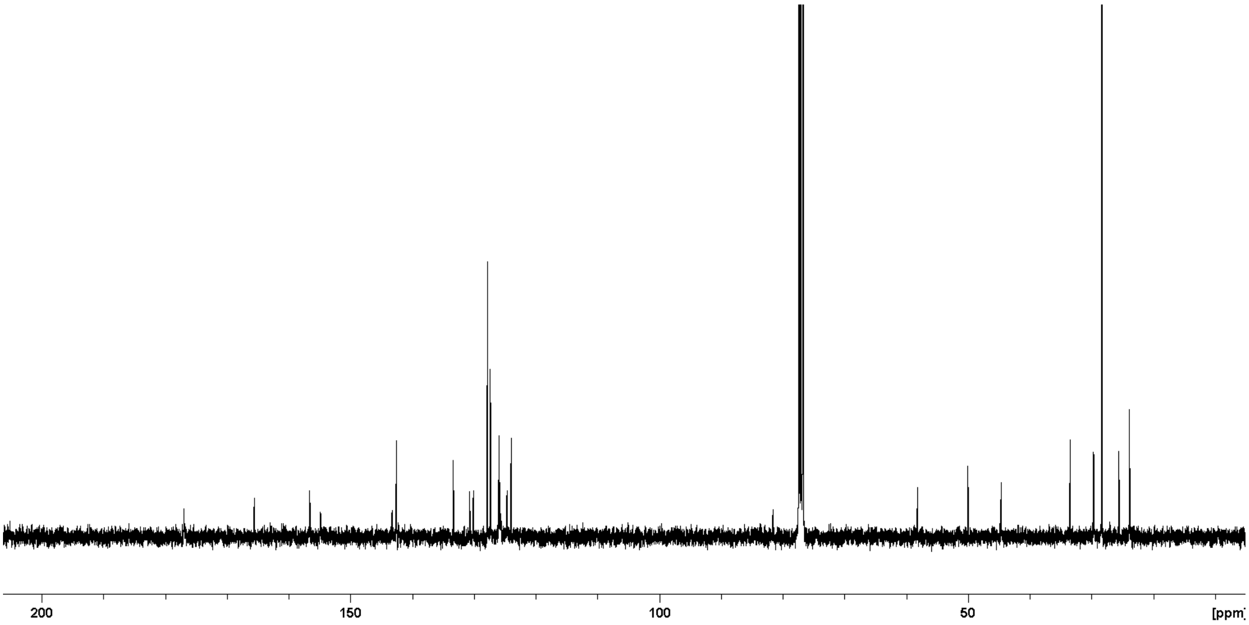
200
150
100
50
[ppm]

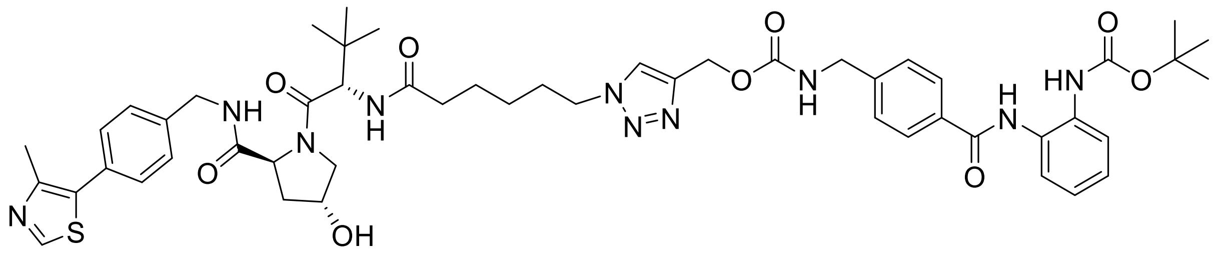

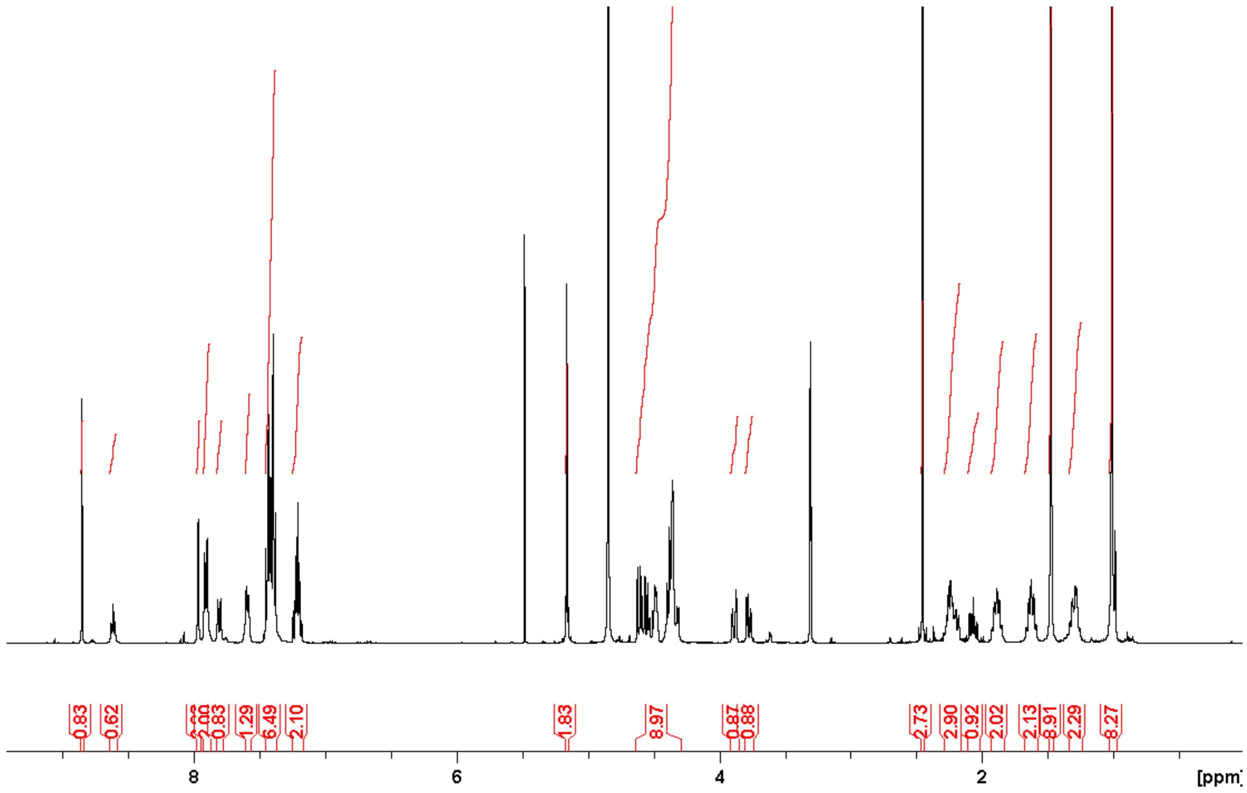
0.83
0.62
2.00
0.83
1.29
6.49
2.10
1.83
8.97
0.87
0.88
2.73
2.90
0.92
2.02
2.13
8.91
2.29
8.27
8
6
4
2
[ppm]

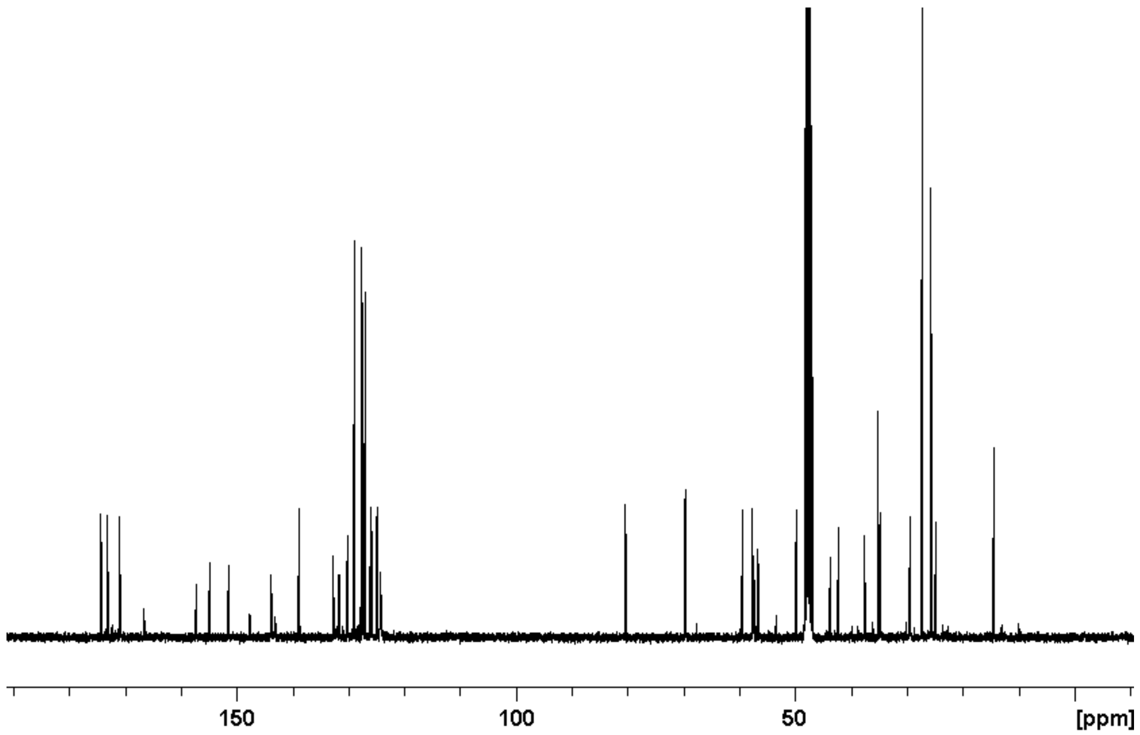
150
100
50
[ppm]

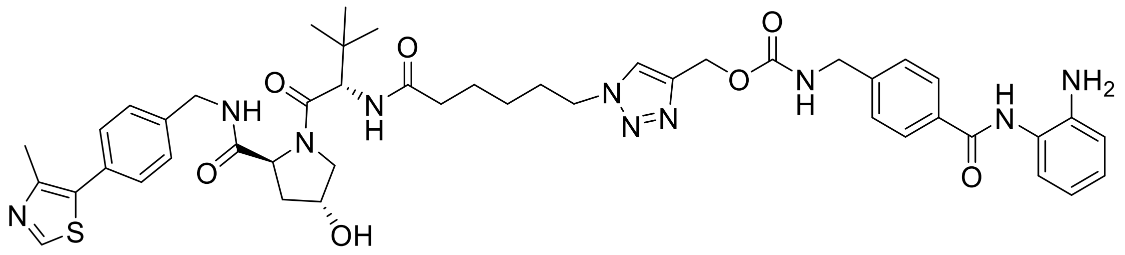

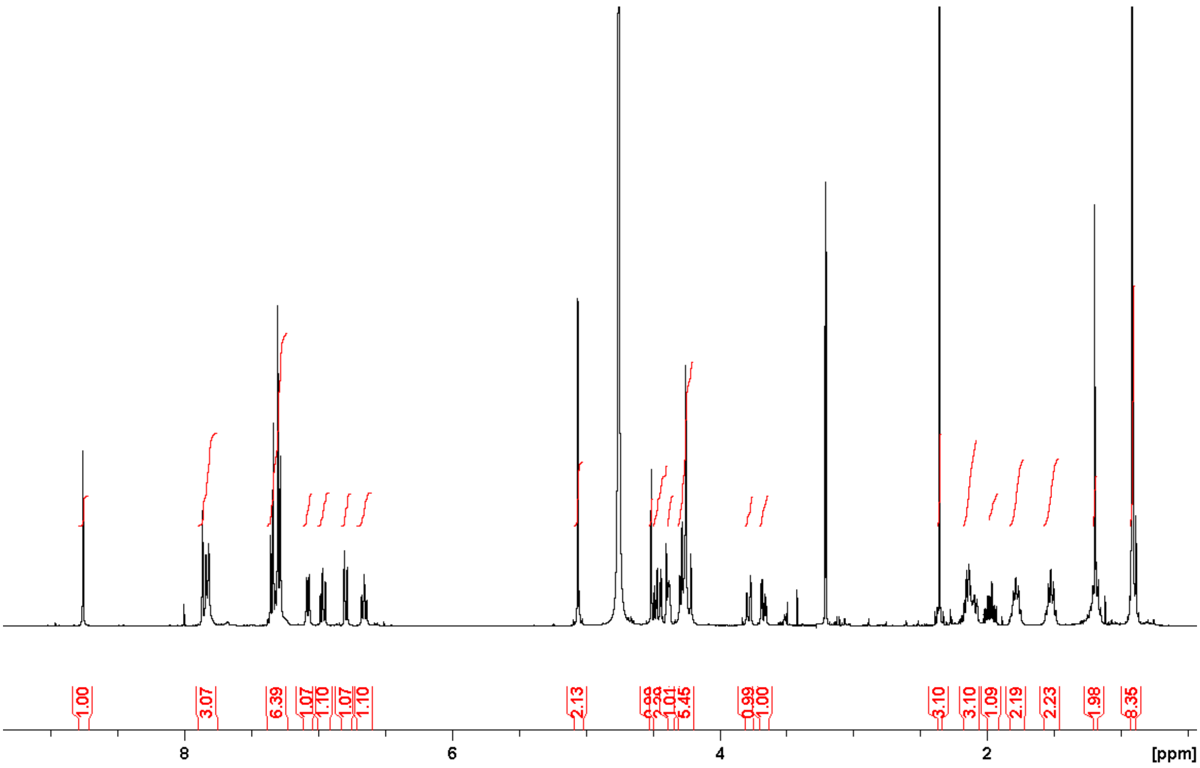
1.00
3.07
6.39
1.07
1.10
1.07
1.10
2.13
1.01
5.45
0.99
1.00
3.10
3.10
1.09
2.19
2.23
1.98
8.35
8
6
4
2
[ppm]

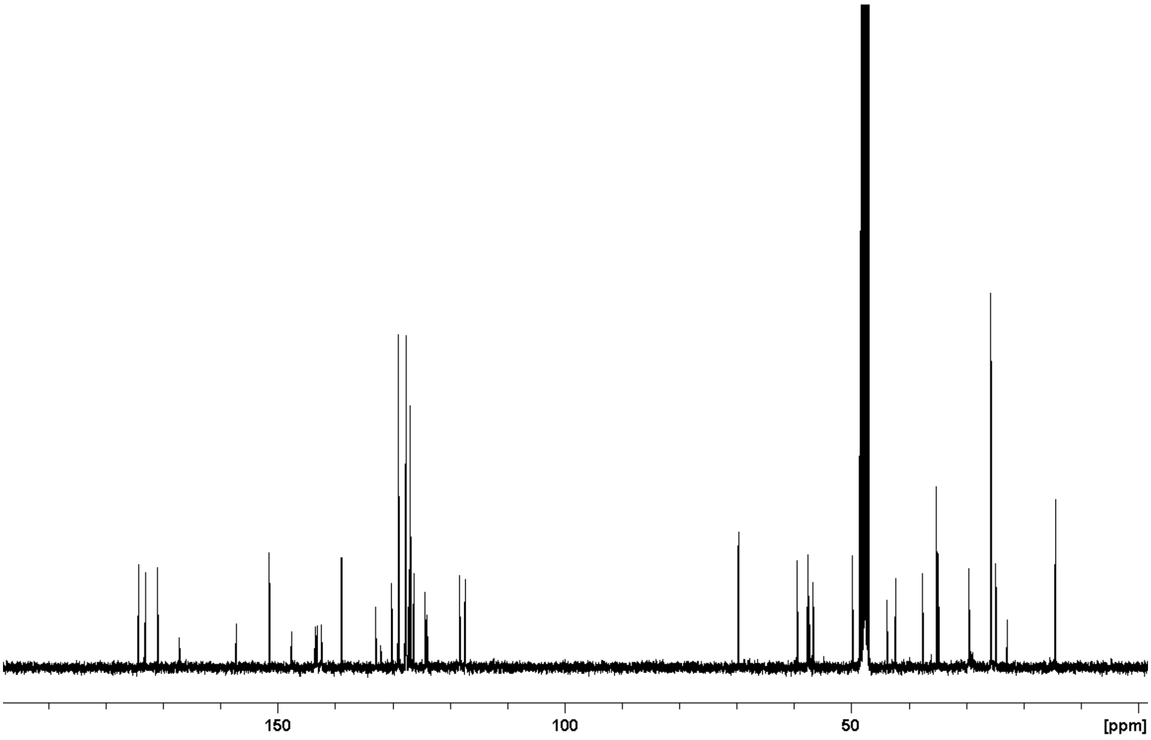
150
100
50
[ppm]

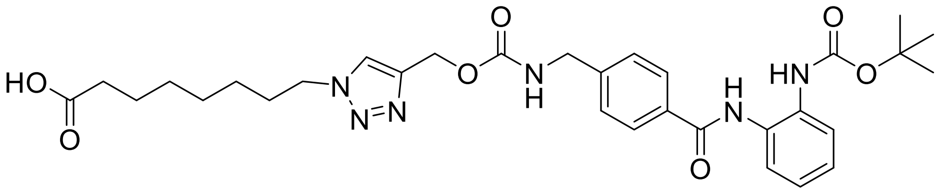

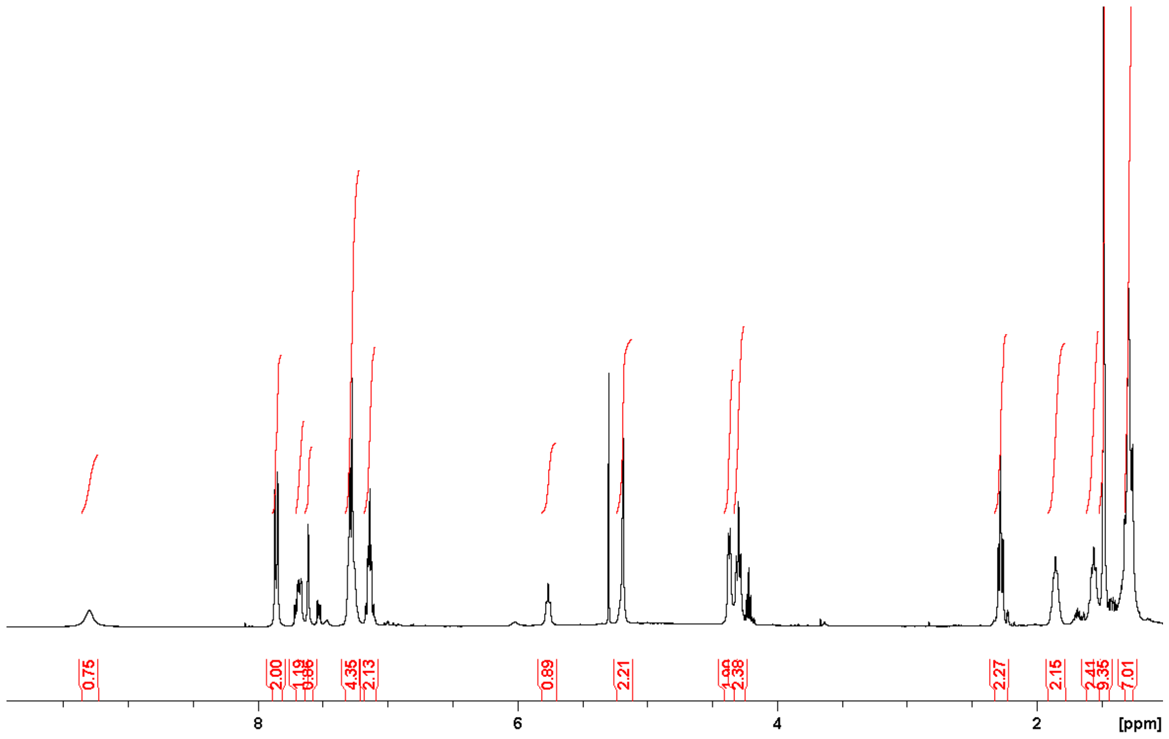

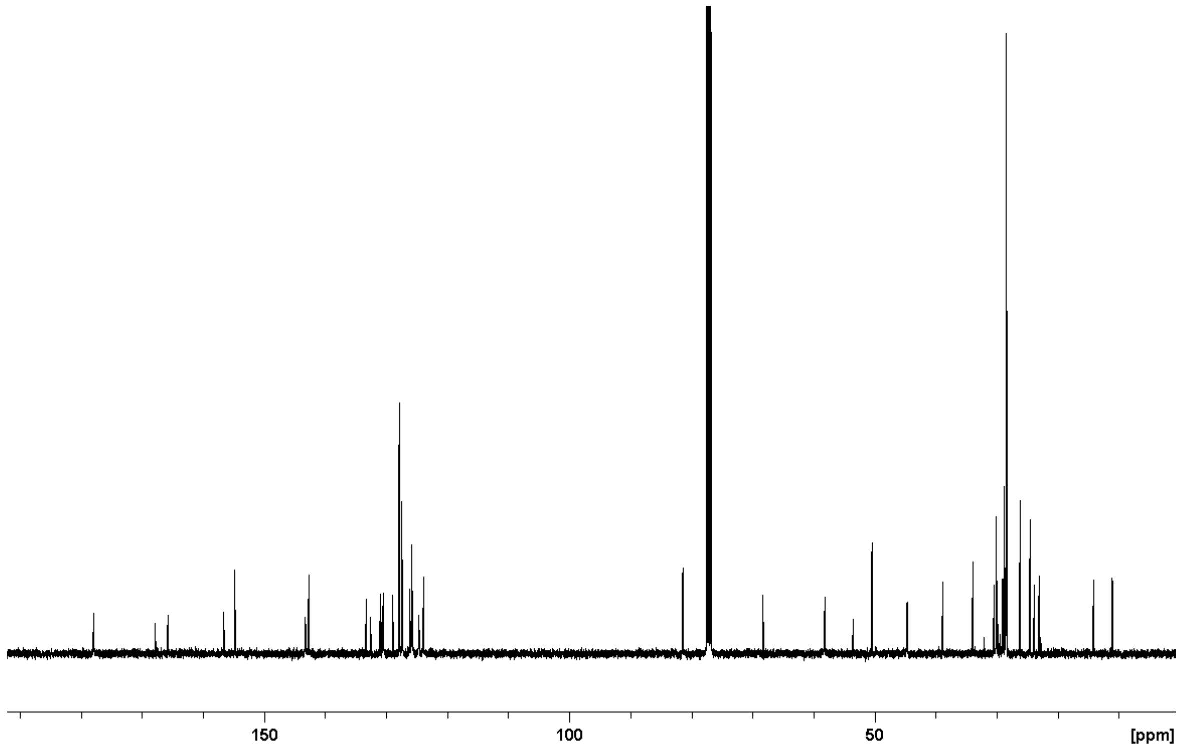

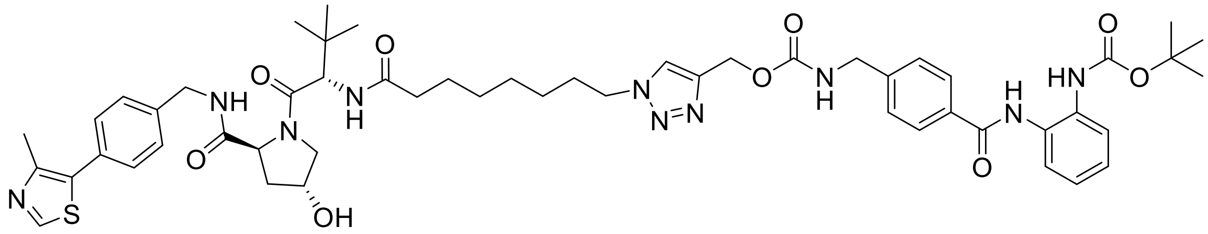

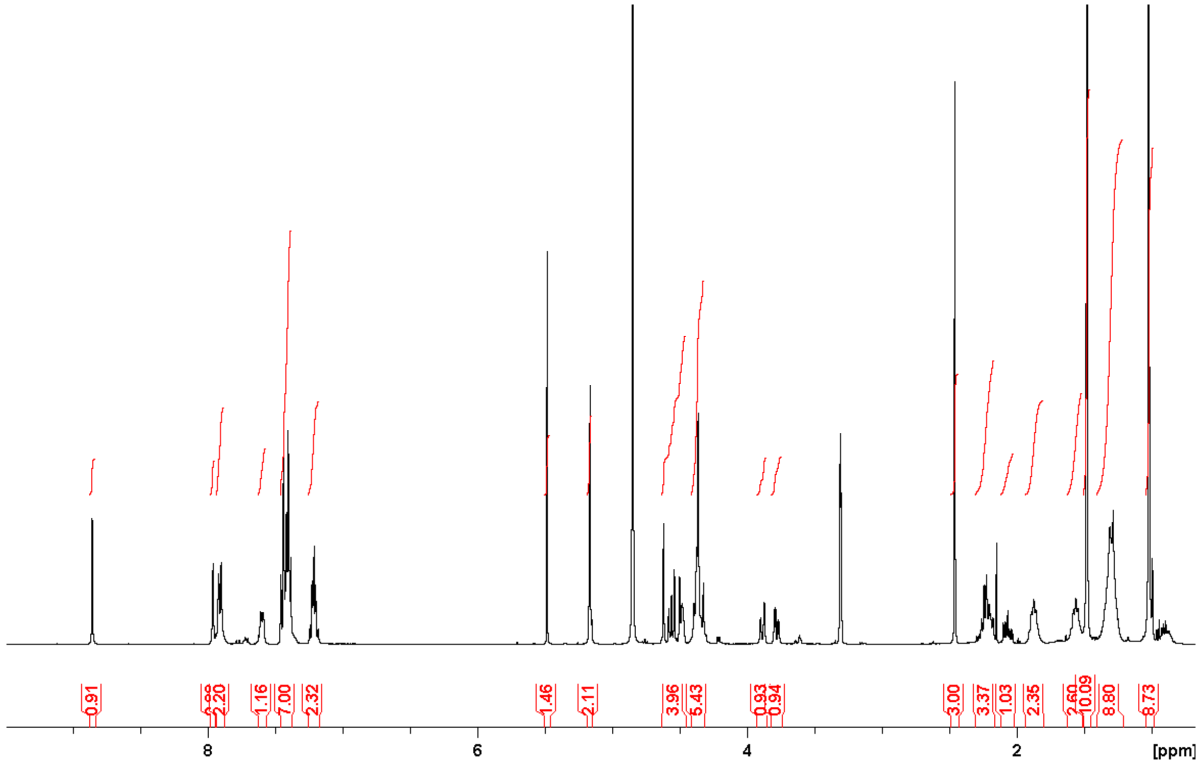
0.91
2.20
1.16
7.00
2.32
1.46
2.11
3.96
5.43
0.93
0.94
3.00
3.37
1.03
2.35
2.60
10.09
8.80
8.73
8
6
4
2
[ppm]

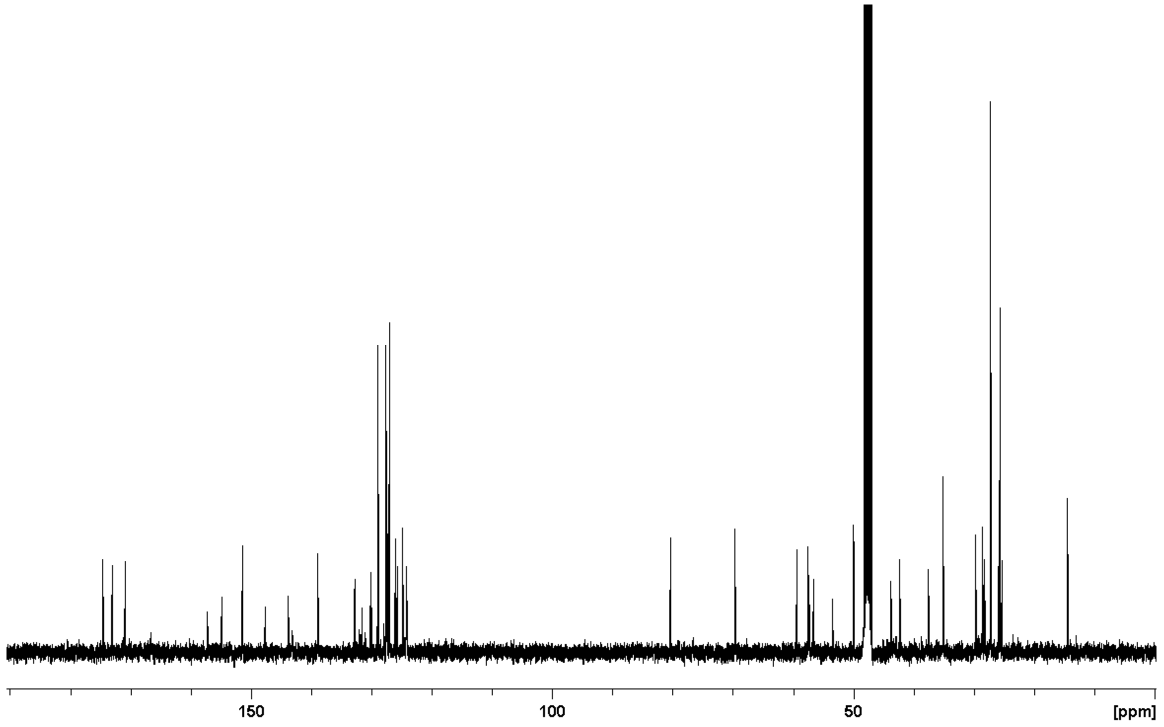
150
100
50
[ppm]

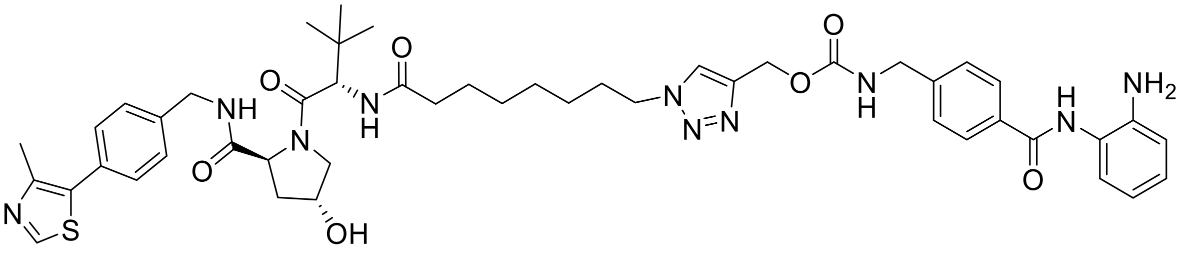
NH
O
N
S
N
OH
O
N
H
O
N
N=N
O
O
N
H
H
N
NH2
O

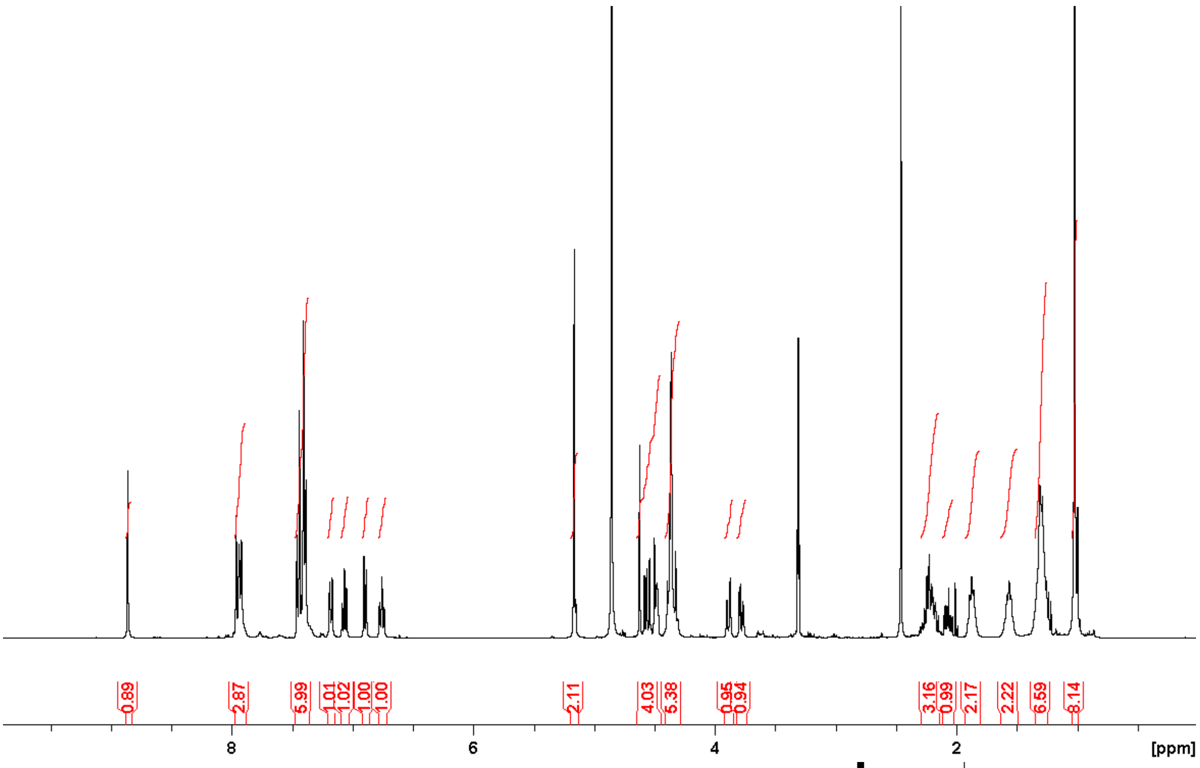
0.89
2.87
5.99
1.01
1.02
1.00
1.00
2.11
4.03
5.38
0.95
0.94
3.16
0.99
2.17
2.22
6.59
8.14
8
6
4
2
[ppm]

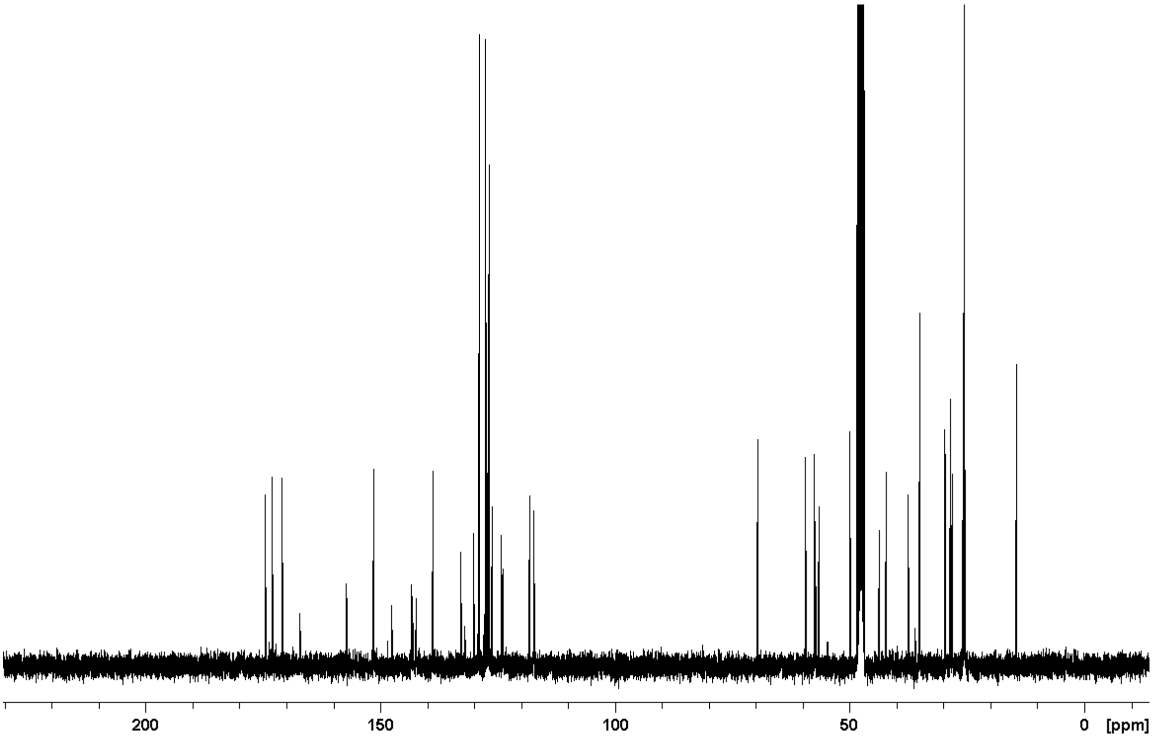
200
150
100
50
0
[ppm]

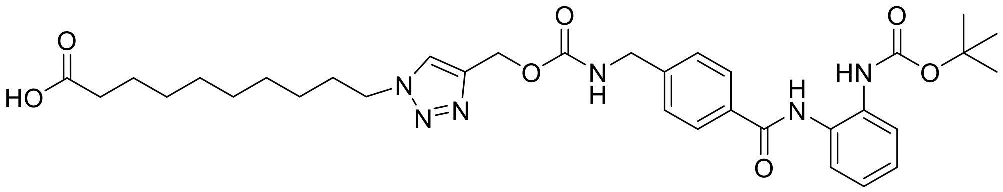
HO
O
N
N=N
O
O
N
H
H
N
O
HN
O
O

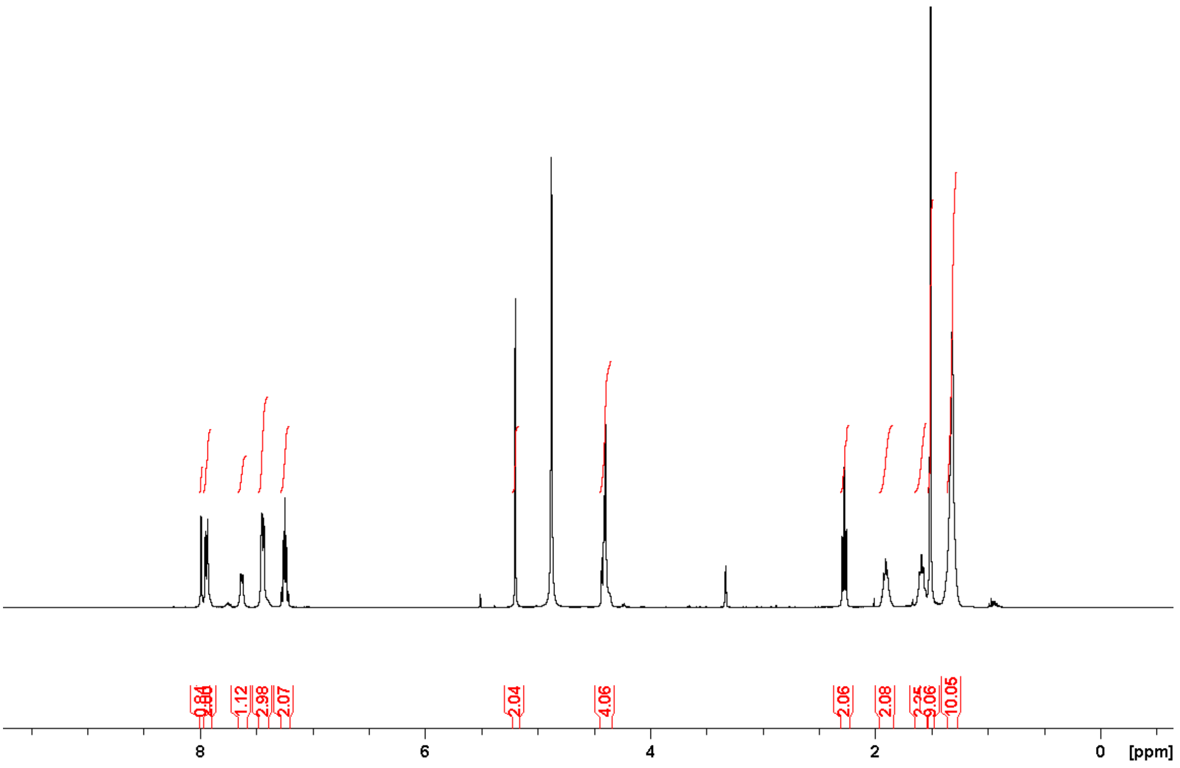
8
6
4
2
0
[ppm]

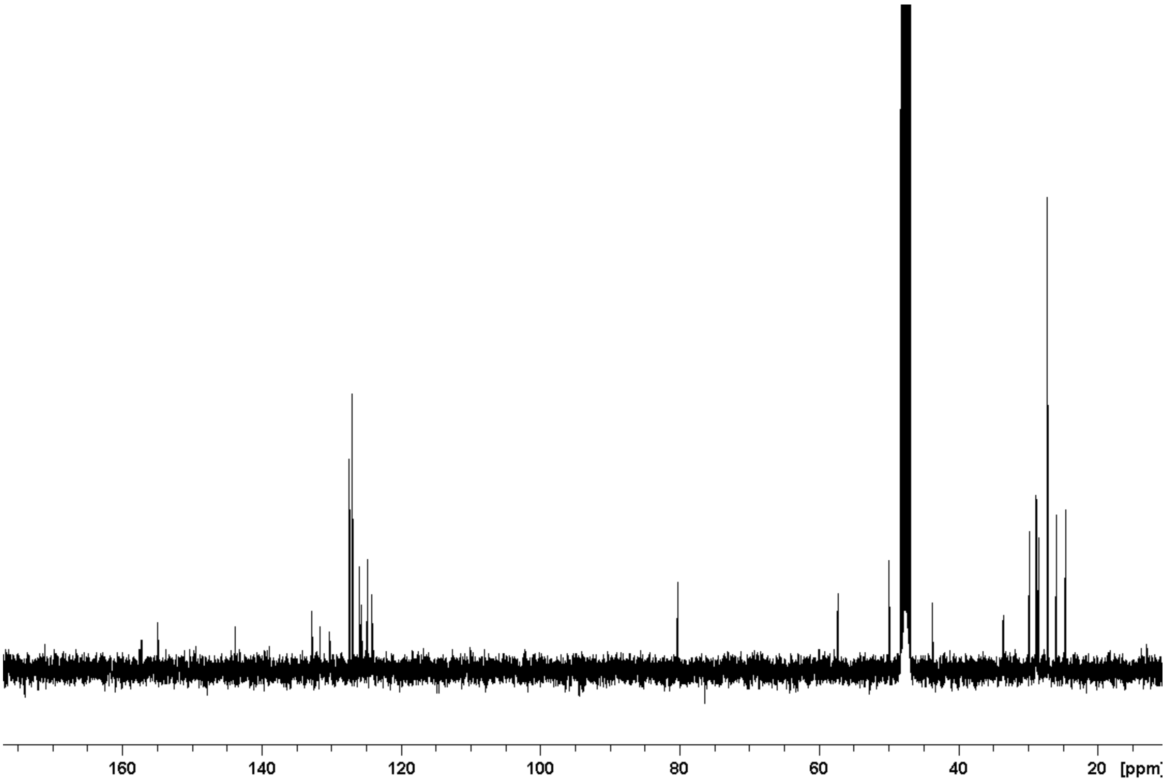
160
140
120
100
80
60
40
20
[ppm]

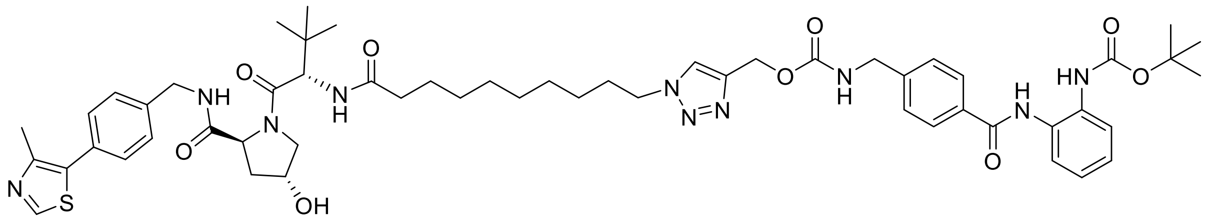
N
S
NH
O
O
N
OH
H
N
O
N
N=N
O
O
N
H
H
N
O
HN
O
O

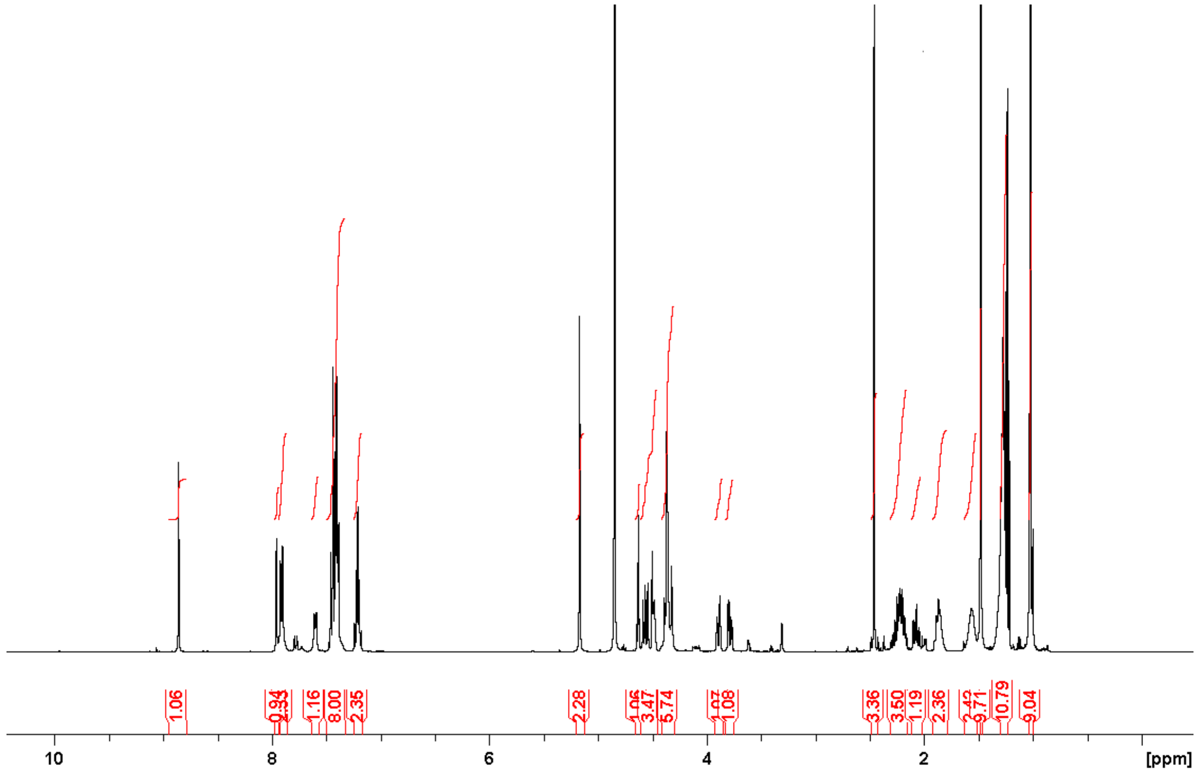
1.06
0.94
2.33
1.16
8.00
2.35
2.28
1.06
3.47
5.74
1.07
1.08
3.36
3.50
1.19
2.36
2.42
9.71
10.79
9.04
10
8
6
4
2
[ppm]

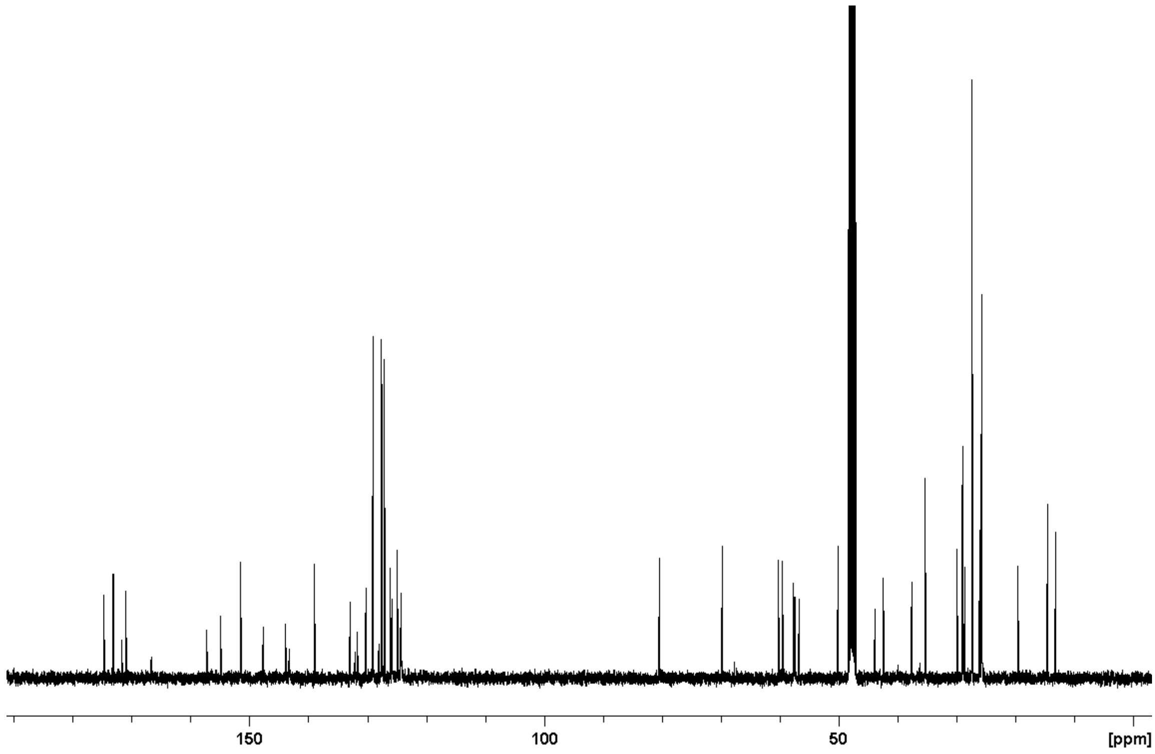
150
100
50
[ppm]

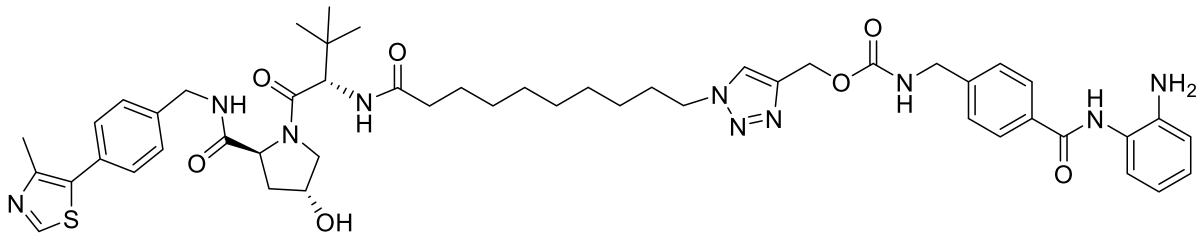

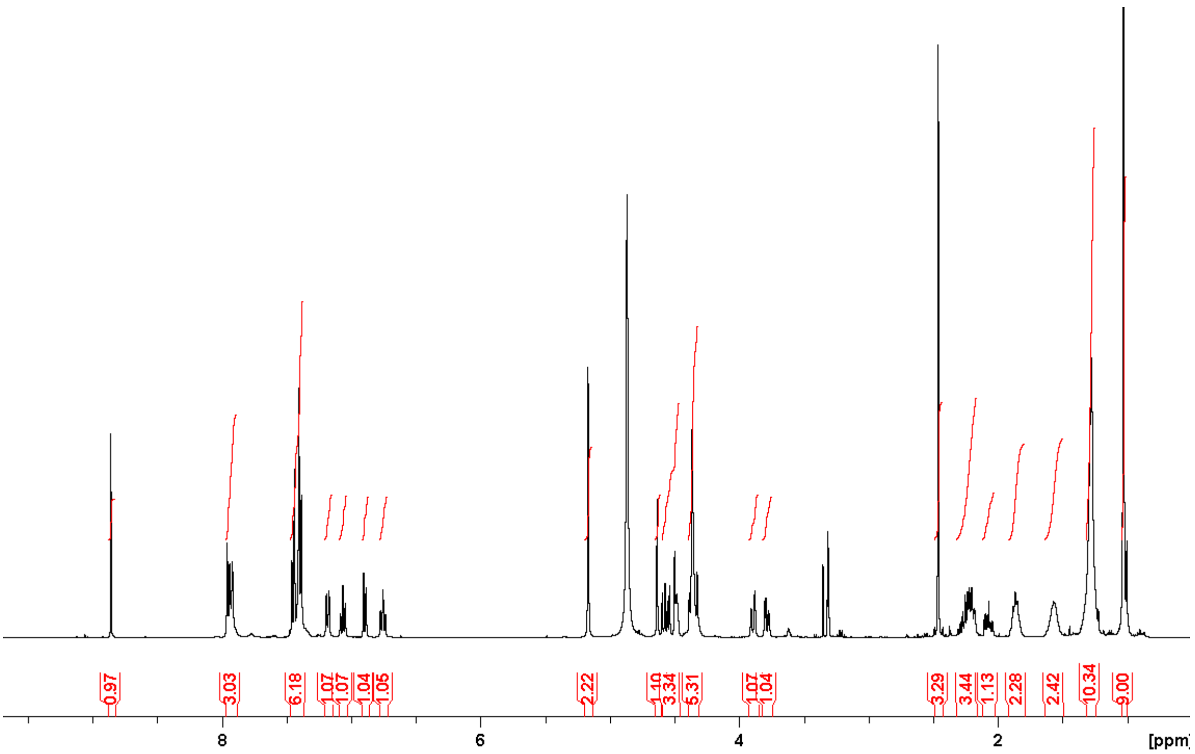
0.97
3.03
6.18
1.07
1.07
1.04
1.05
2.22
1.10
3.34
5.31
1.07
1.04
3.29
3.44
1.13
2.28
2.42
10.34
9.00
8
6
4
2
[ppm]

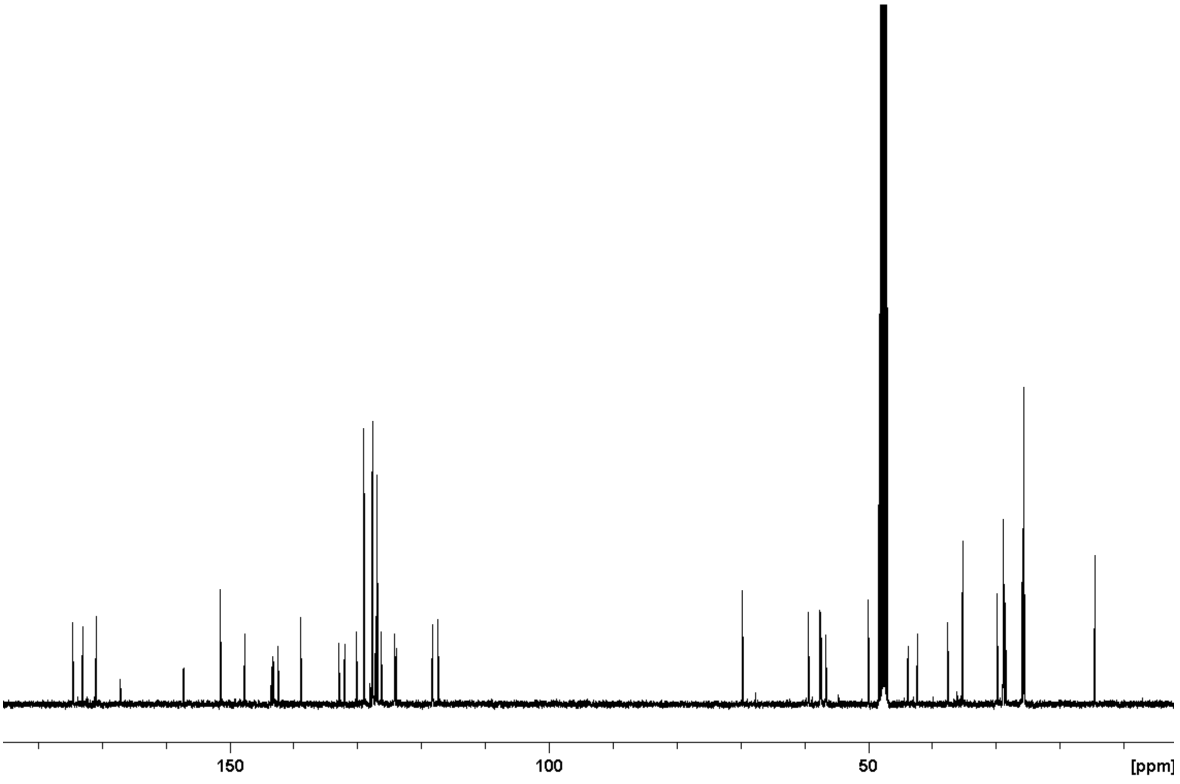
150
100
50
[ppm]

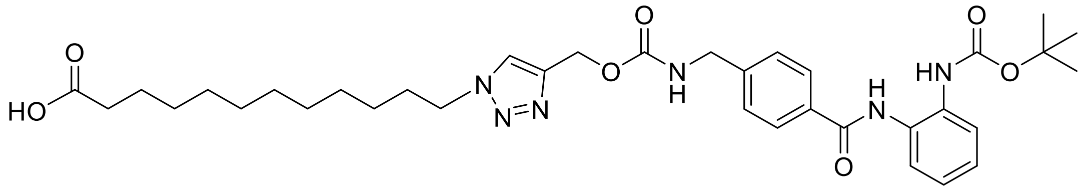
HO
O
N
N
N
O
O
N
H
H
N
O
HN
O
O

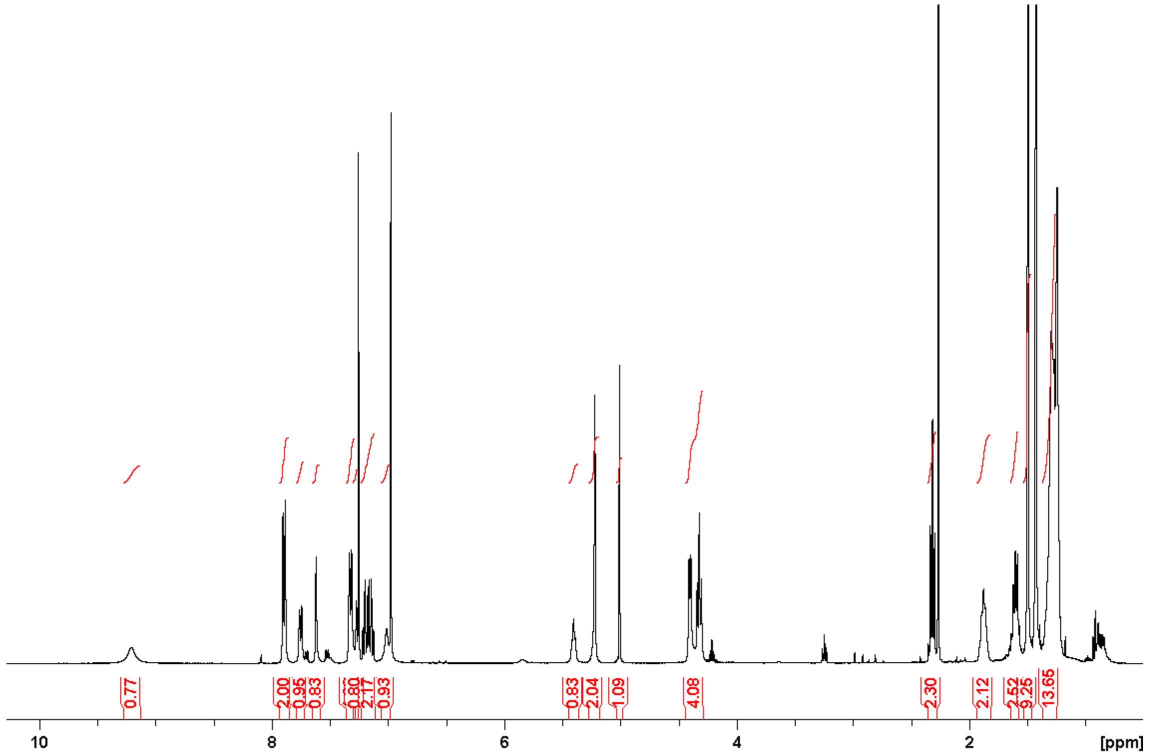
0.77
2.00
0.95
0.83
2.17
0.93
0.83
2.04
1.09
4.08
2.30
2.12
2.52
9.25
13.65
10
8
6
4
2
[ppm]

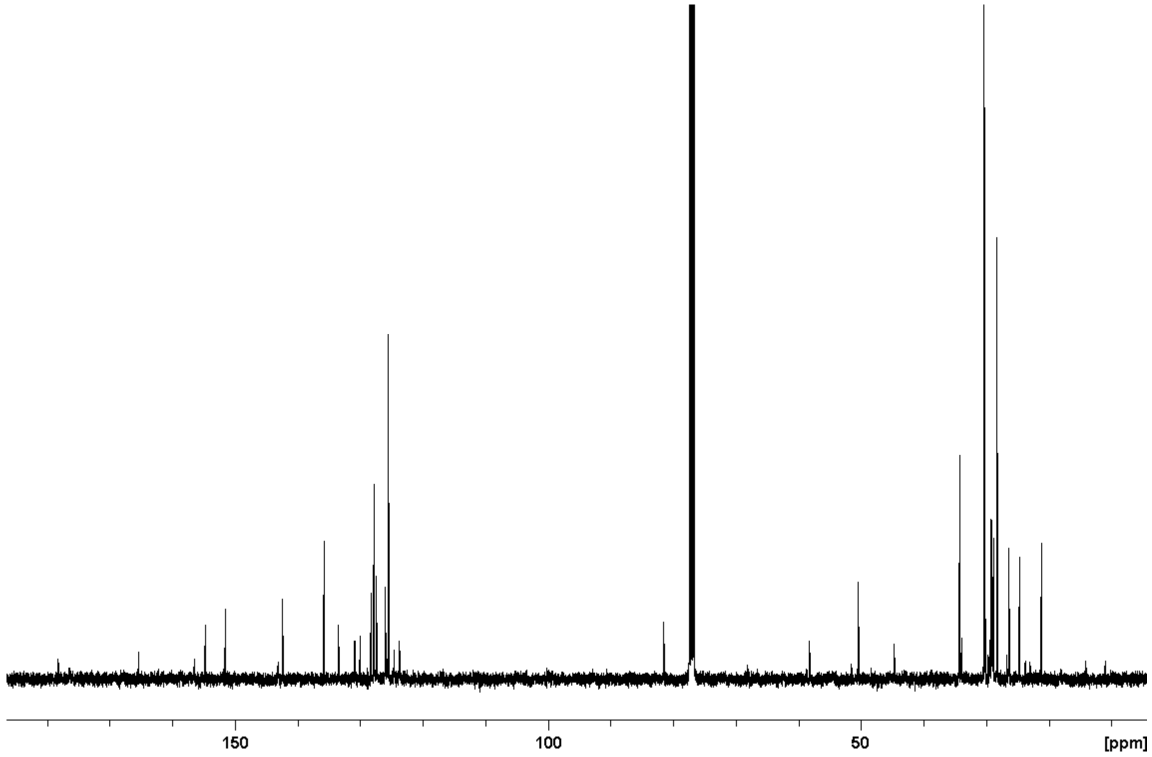
150
100
50
[ppm]

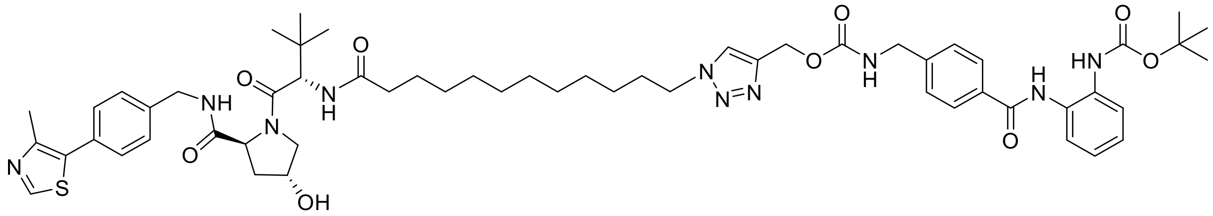

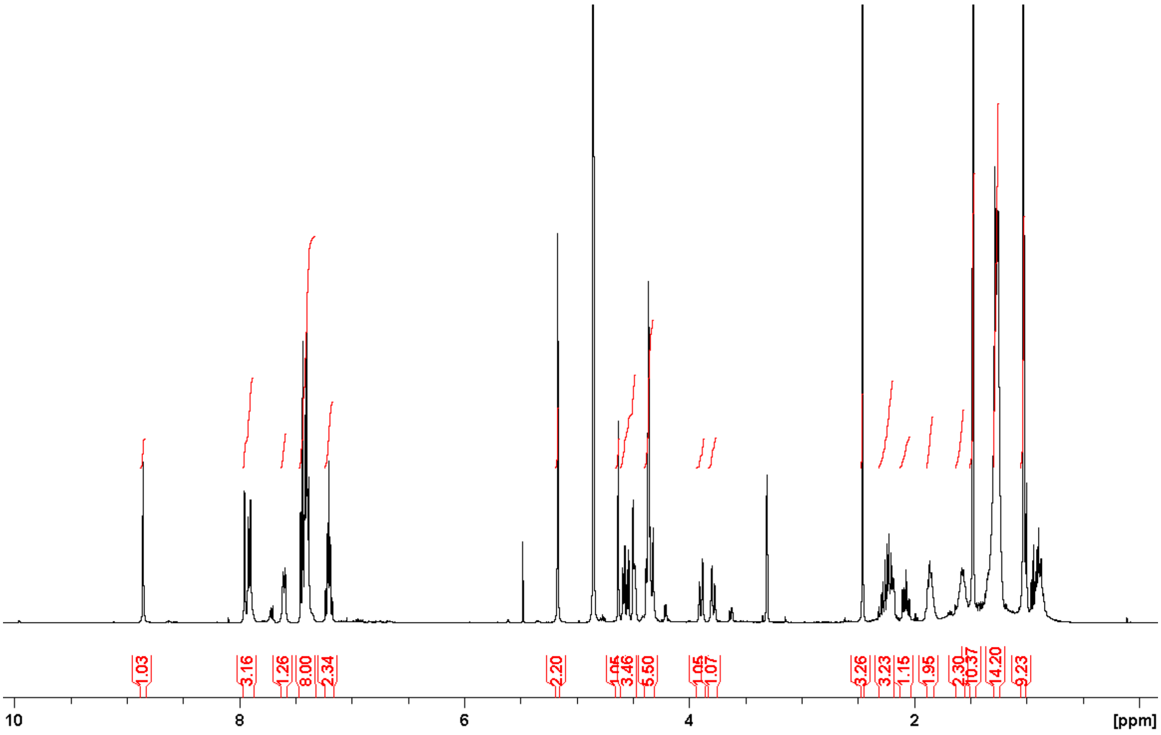
1.03
3.16
1.26
8.00
2.34
2.20
1.05
3.46
5.50
1.05
1.07
3.26
3.23
1.15
1.95
2.30
10.37
14.20
9.23
10
8
6
4
2
[ppm]

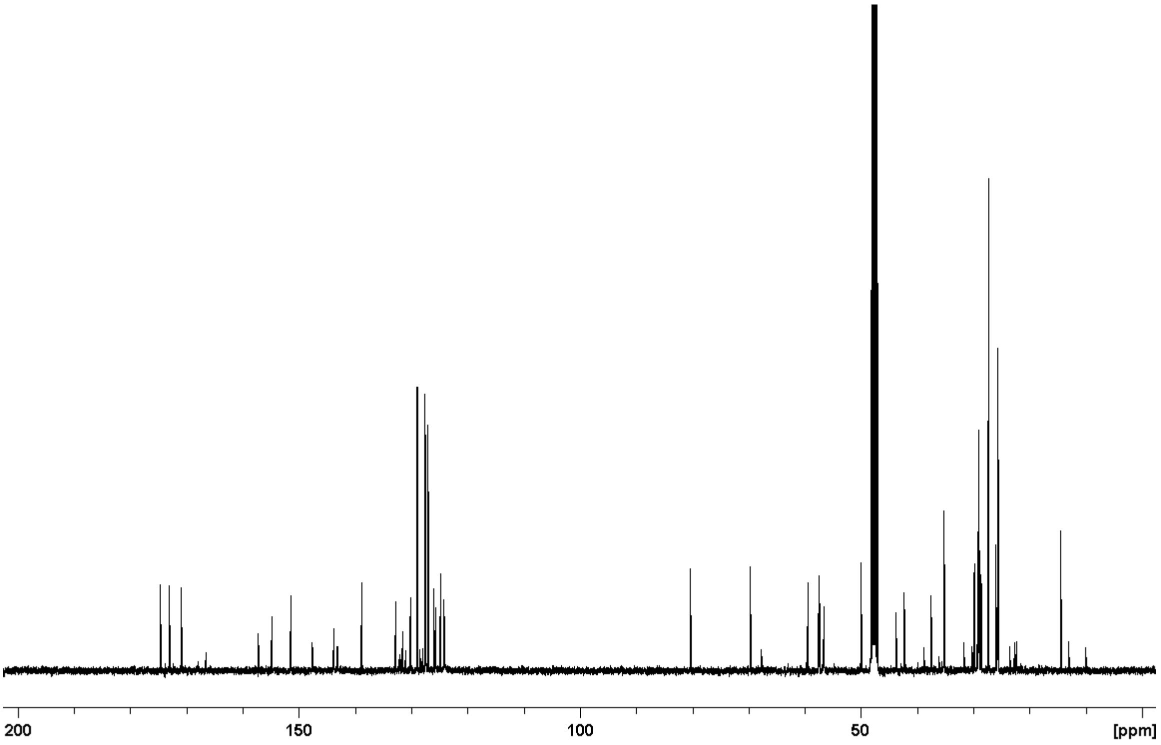
200
150
100
50
[ppm]

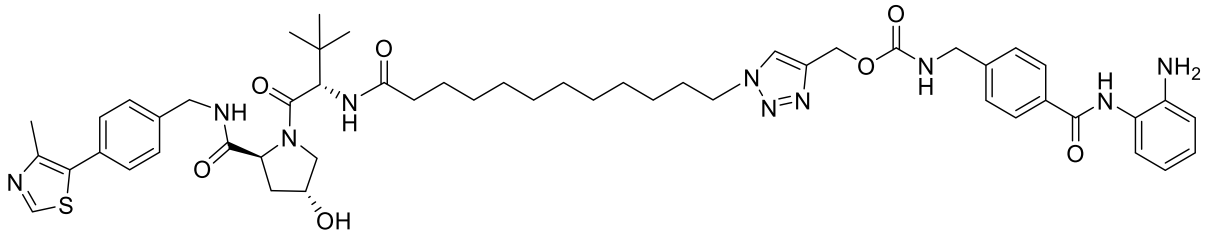

NH
N
O
O
OH
H
N
N
N
N
O
O
N
H
NH2
H
N
S

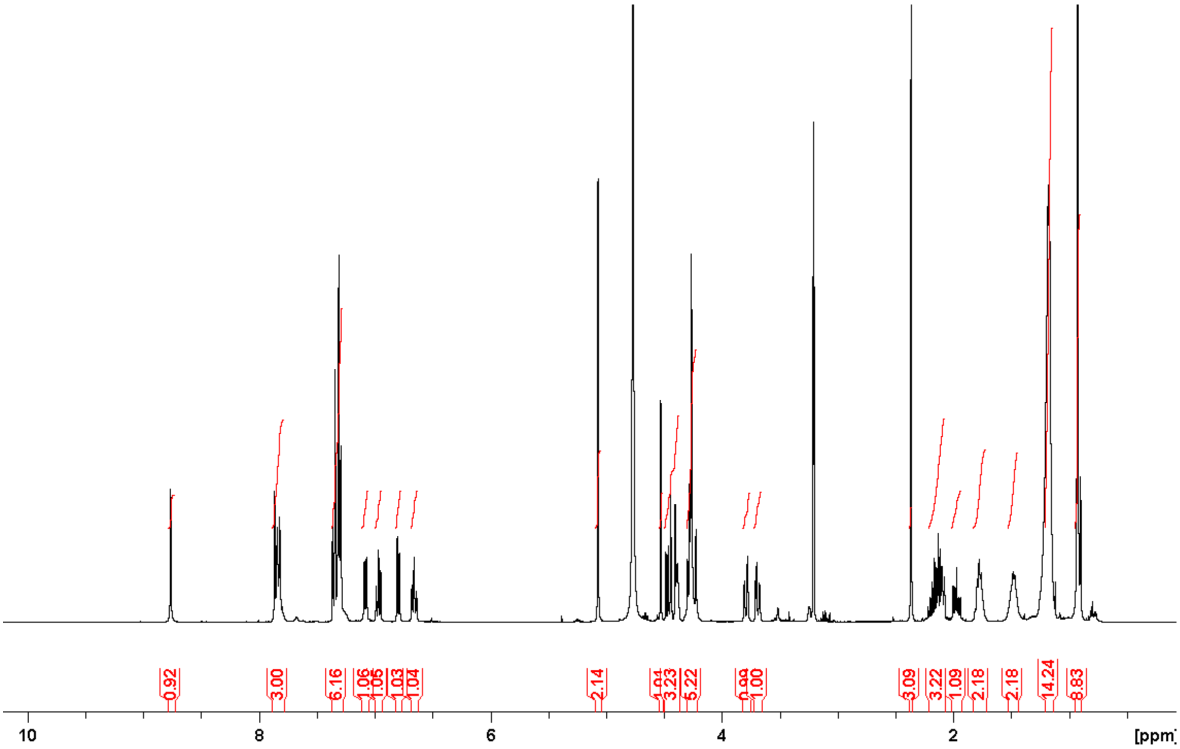

0.92
3.00
6.16
1.06
1.05
1.03
1.04
2.14
3.23
5.22
1.00
3.09
3.22
1.09
2.18
2.18
14.24
8.83
10
8
6
4
2
[ppm]

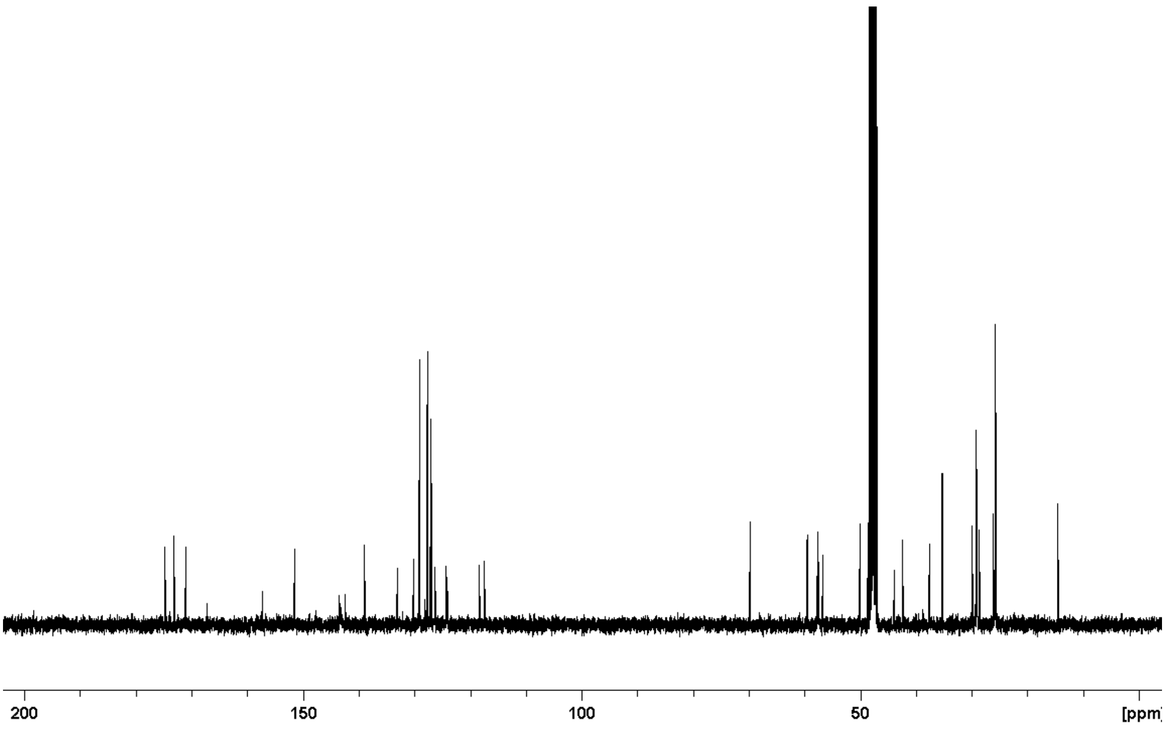

200
150
100
50
[ppm]

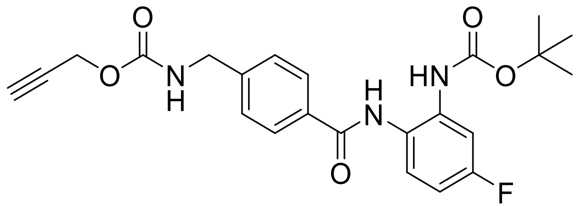

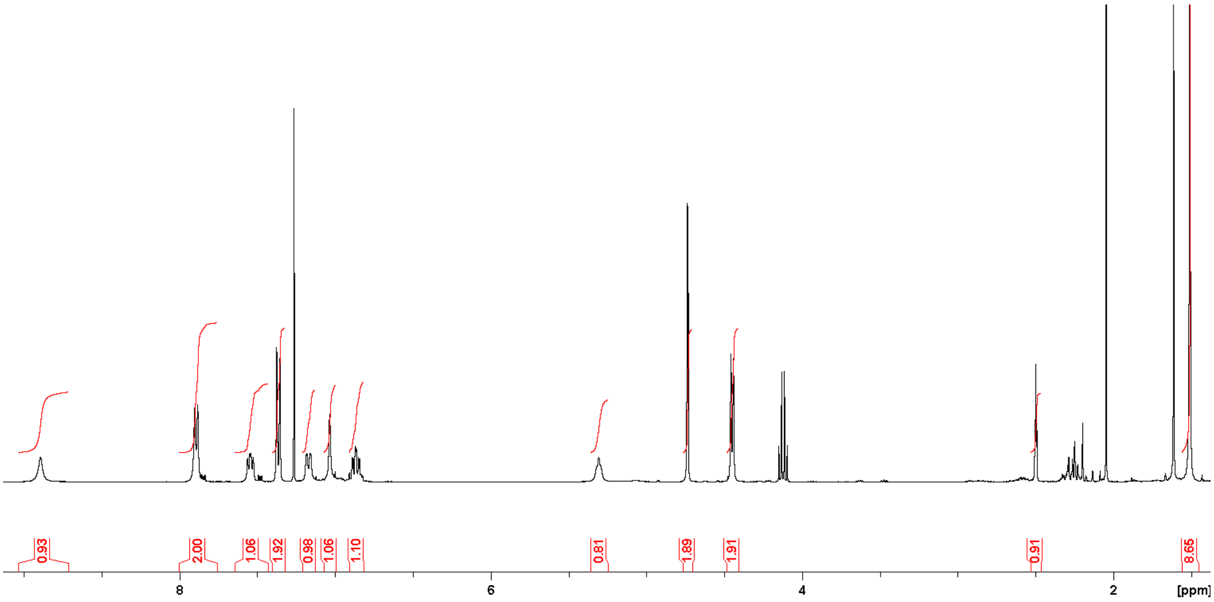

0.93
2.00
1.06
1.92
0.98
1.06
1.10
0.81
1.89
1.91
0.91
8.65
8
6
4
2
[ppm]

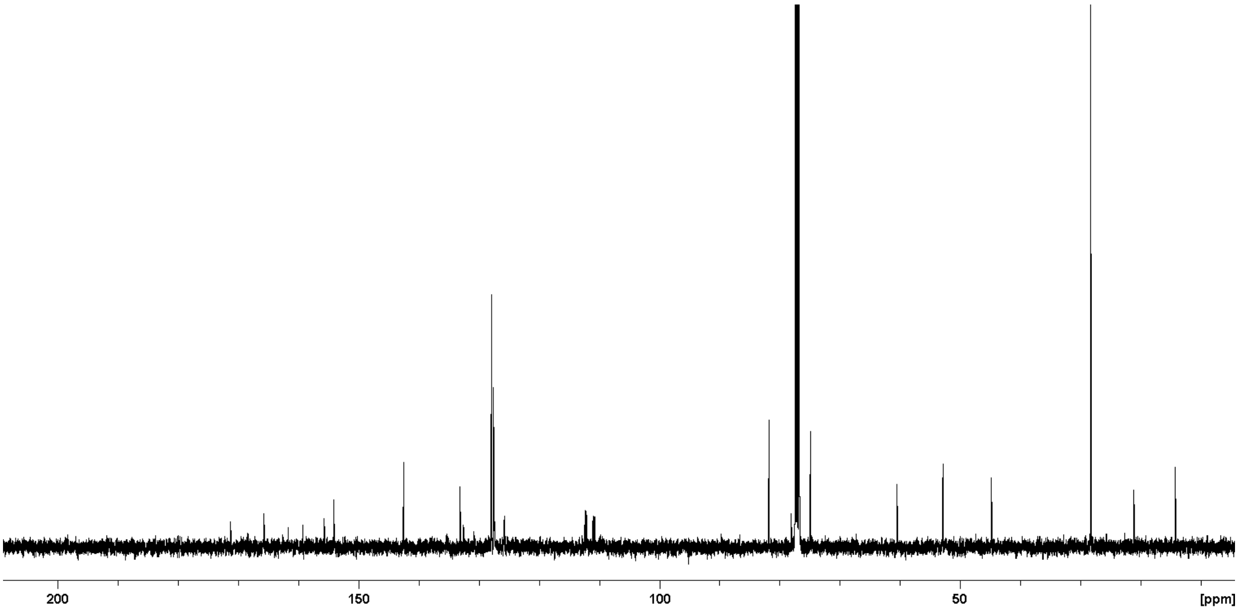

200
150
100
50
[ppm]

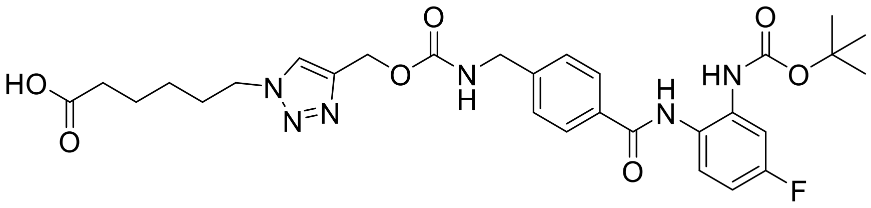
HO
O
N
N=N
O
O
N
H
H
N
O
HN
O
O
F

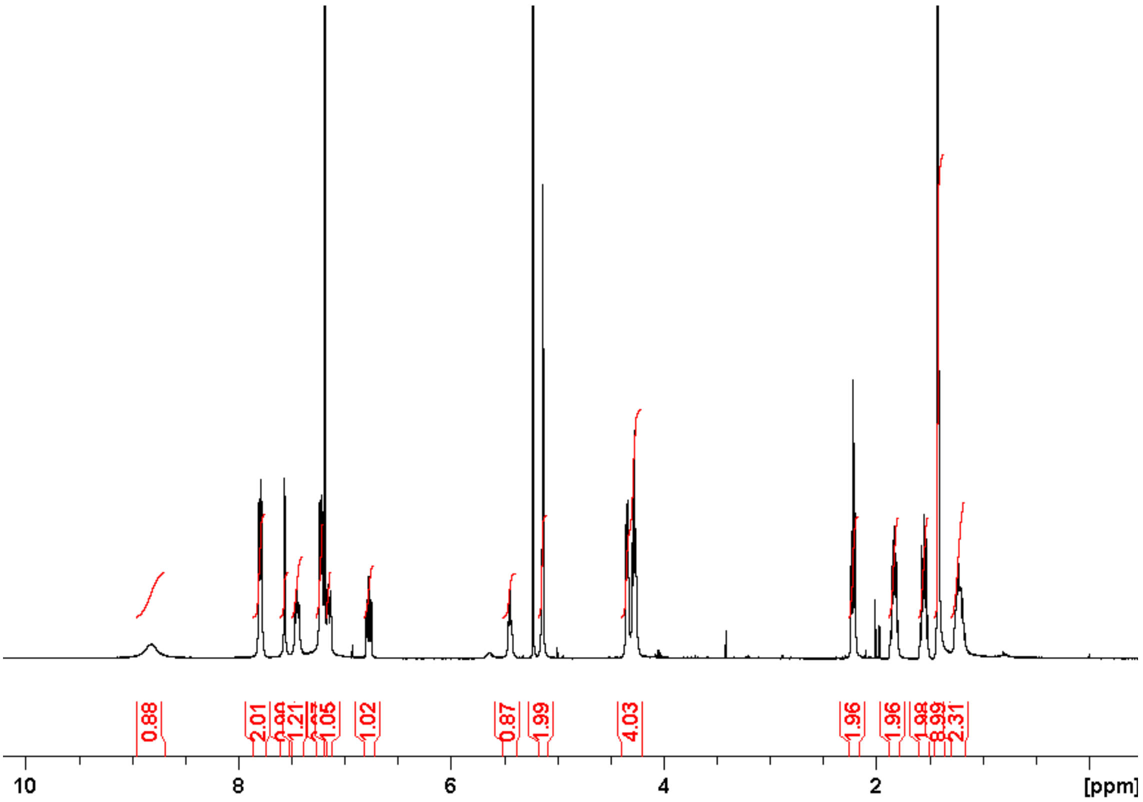
0.88
2.01
1.21
1.05
1.02
0.87
1.99
4.03
1.96
1.96
8.99
2.31
10
8
6
4
2
[ppm]

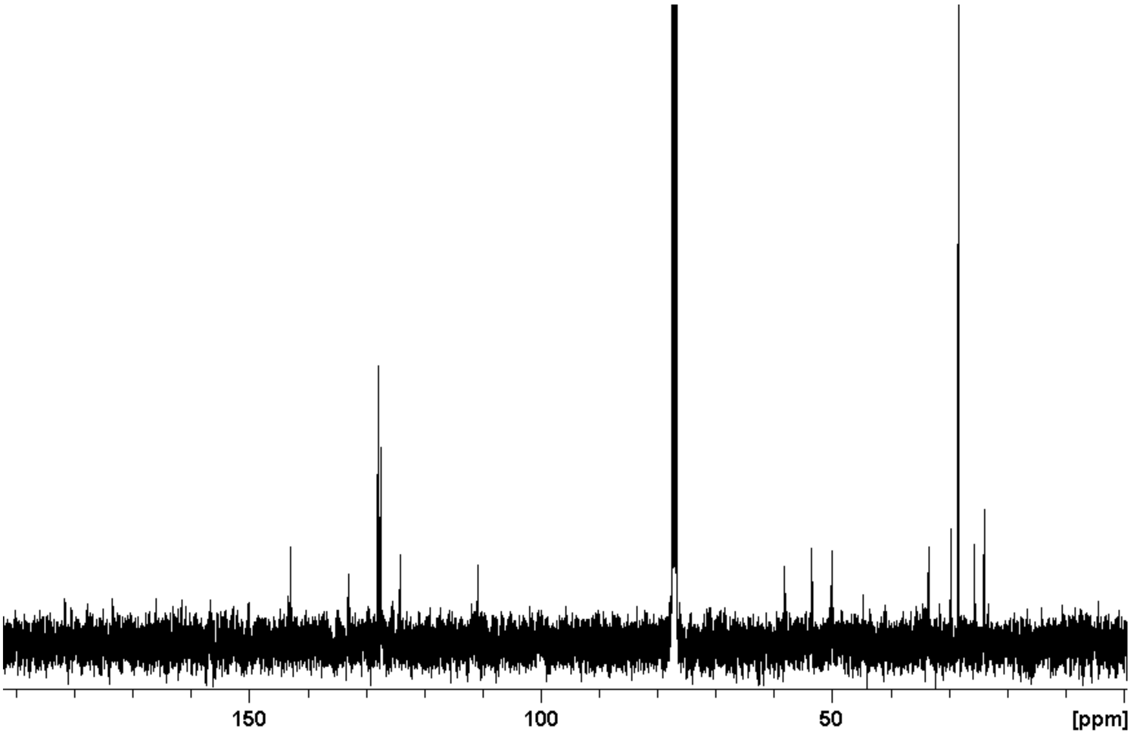
150
100
50
[ppm]

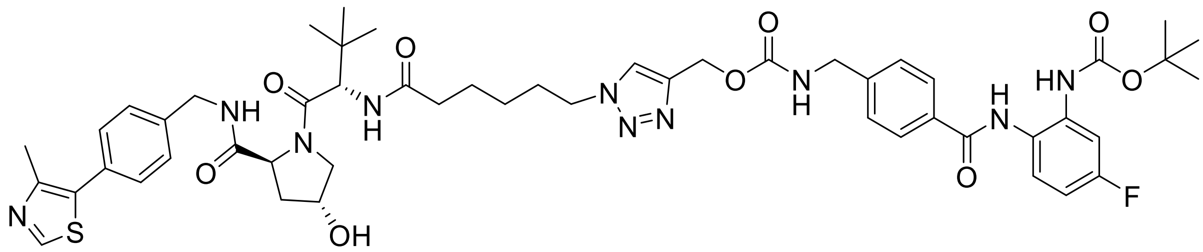

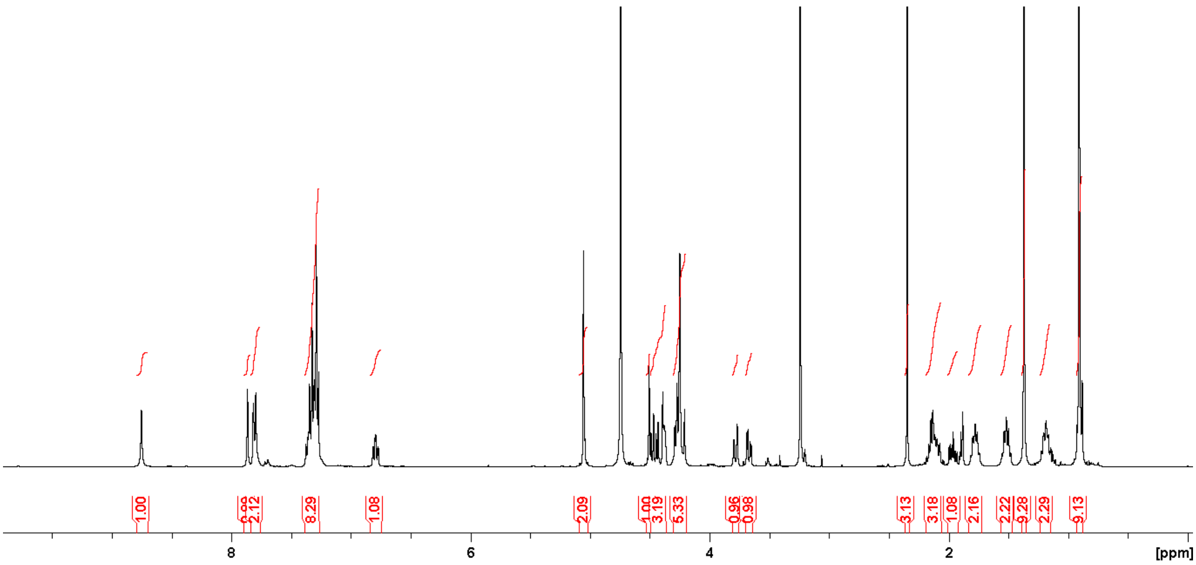
1.00
2.12
8.29
1.08
2.09
1.00
3.19
6.33
0.96
0.98
3.13
3.19
1.08
2.16
2.22
9.28
2.29
9.13
8
6
4
2
[ppm]

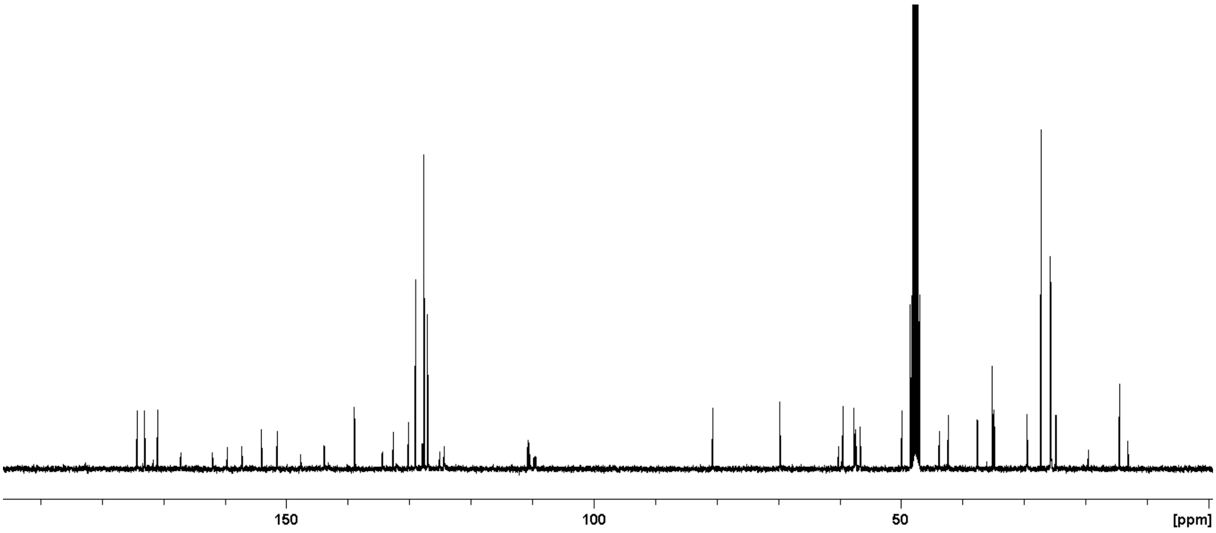
150
100
50
[ppm]

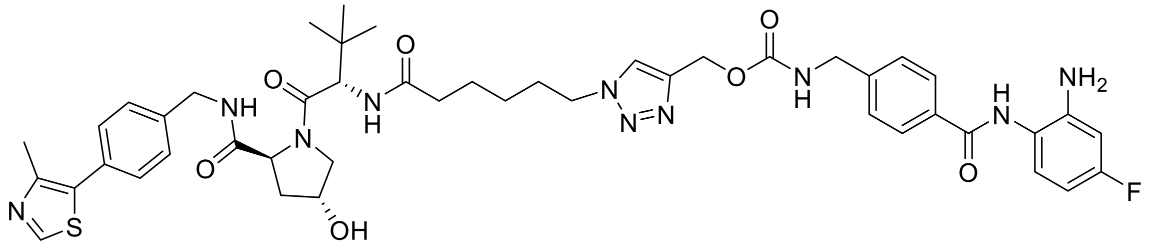

O
O
NH
N
H
N
O
N
N N
O
O
N
H
H
N
NH2
O
F
N
S
OH

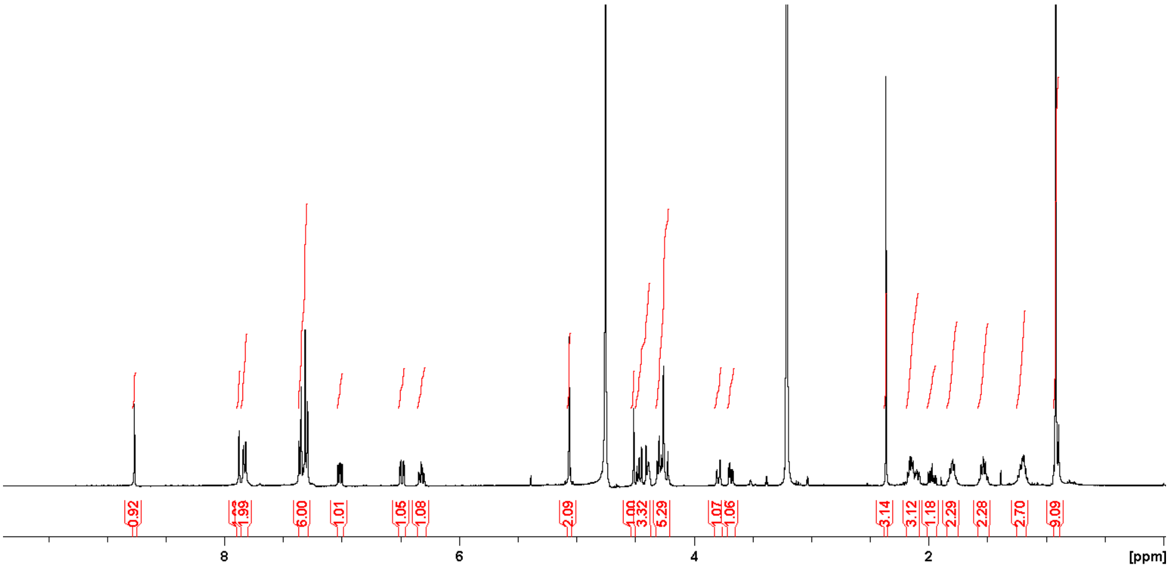

0.92
1.99
6.00
1.01
1.05
1.08
2.09
1.00
3.32
5.29
1.07
1.06
3.14
3.12
1.18
2.29
2.28
2.70
9.09
8
6
4
2
[ppm]

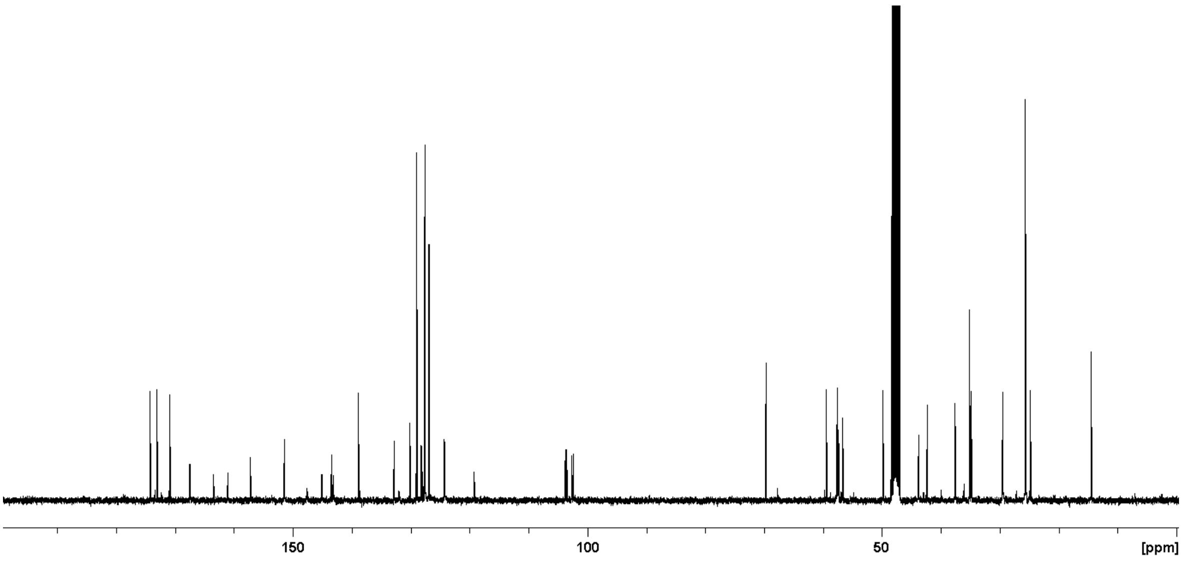

150
100
50
[ppm]

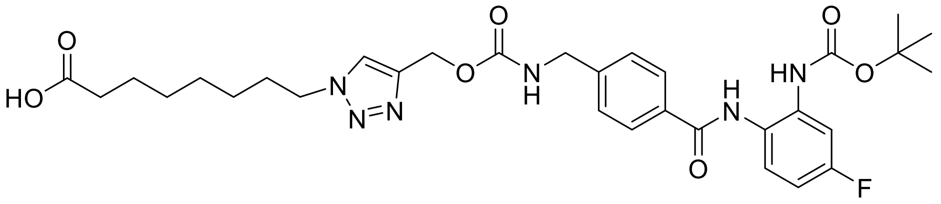

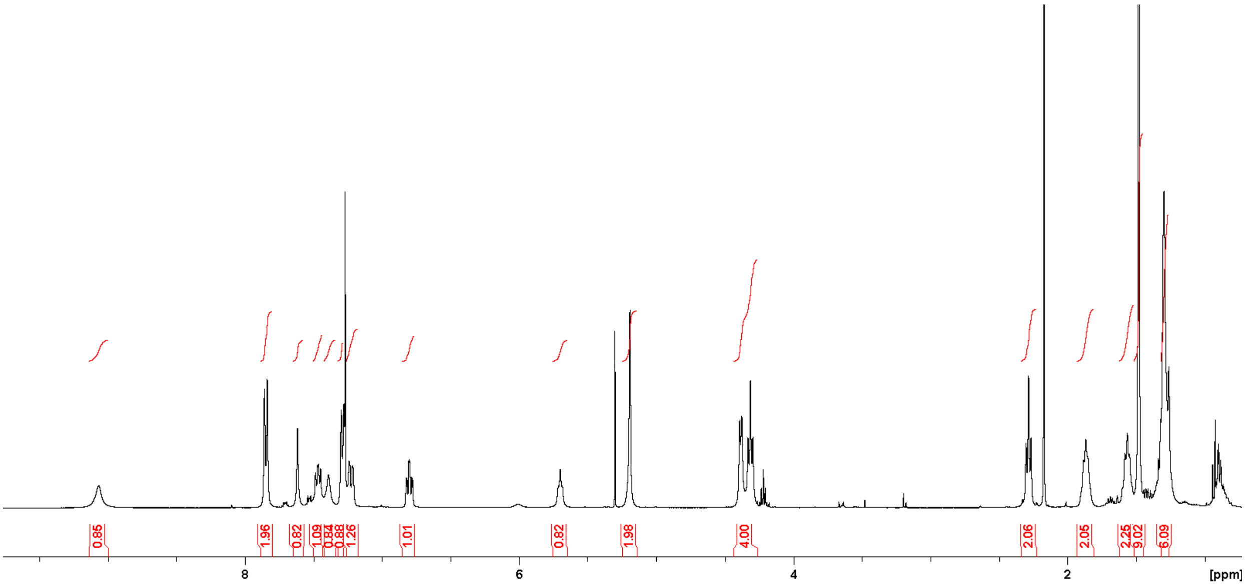
8
6
4
2
[ppm]

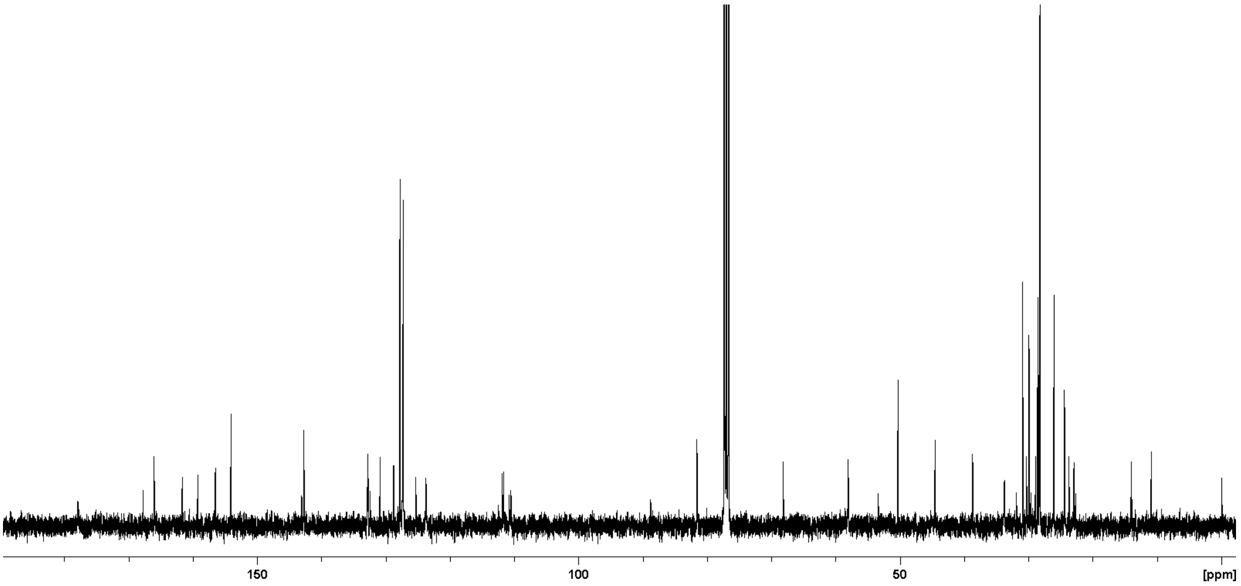
150
100
50
[ppm]

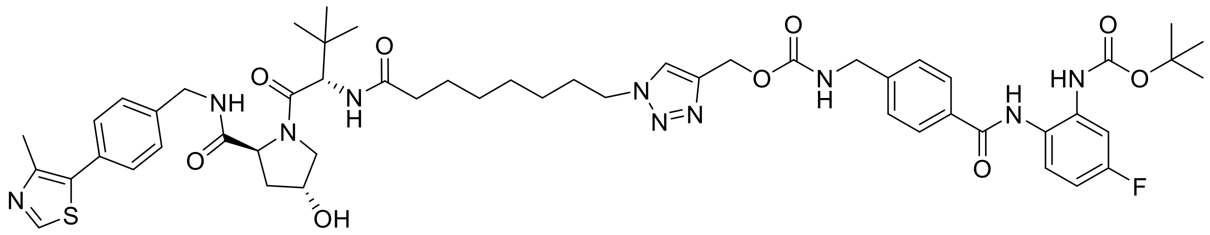

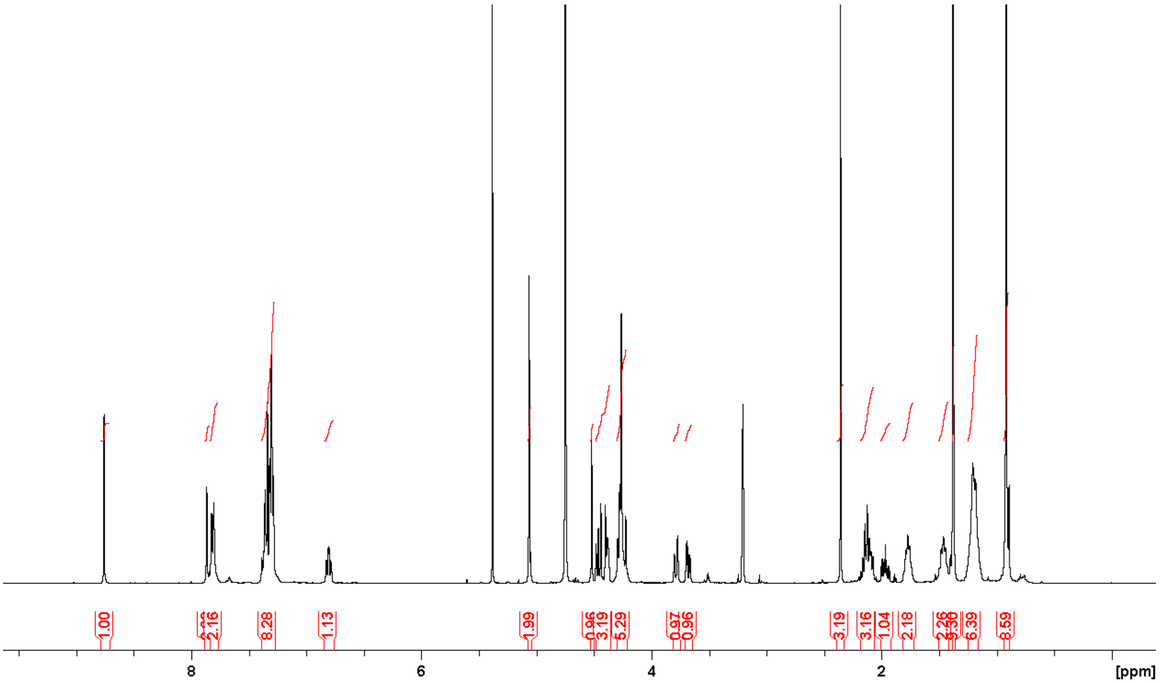
1.00
2.16
8.28
1.13
1.99
0.96
3.19
5.29
0.97
0.96
3.19
3.16
1.04
2.18
6.39
8.59
8
6
4
2
[ppm]

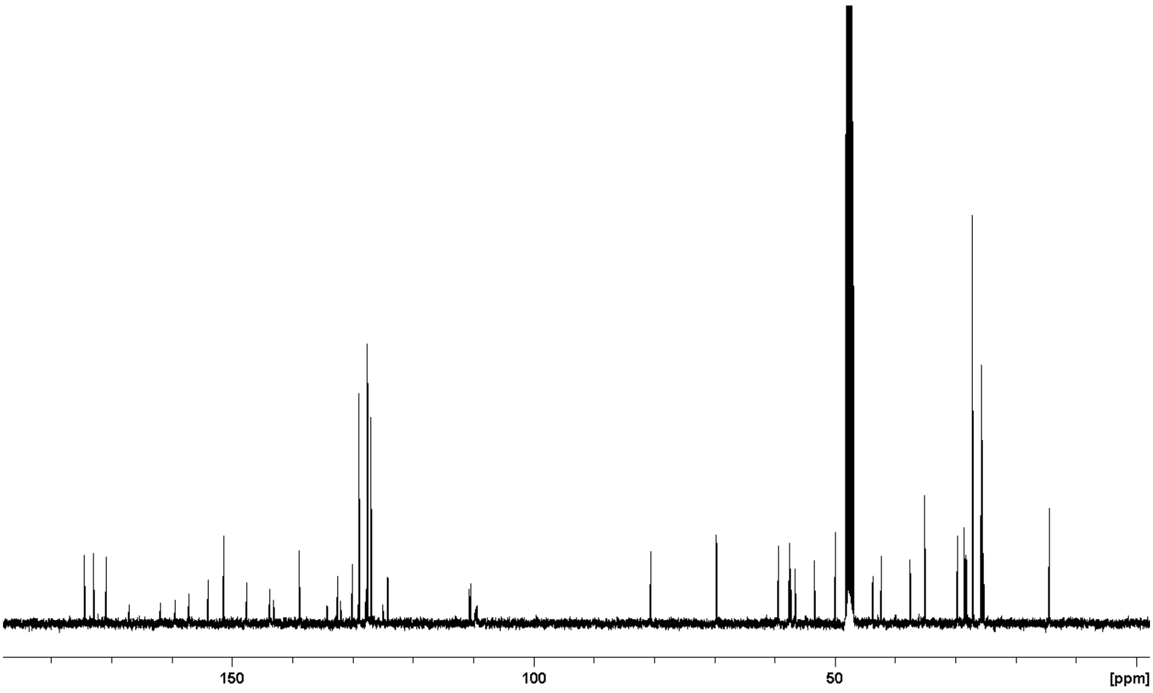
150
100
50
[ppm]

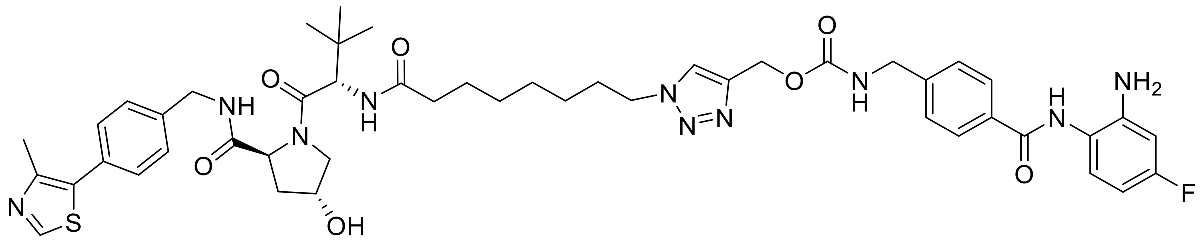
NH
N
O
O
OH
N
S
O
N
H
N
N=N
O
O
N
H
H
N
NH2
O
F

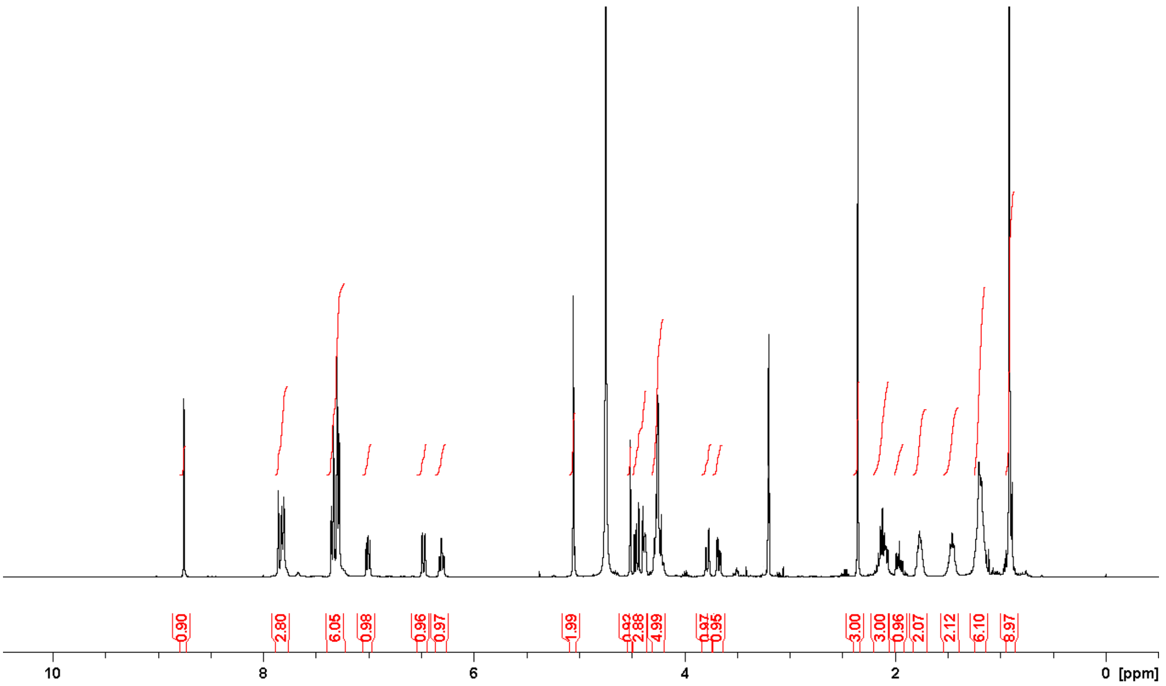
0.90
2.80
6.05
0.98
0.96
0.97
1.99
0.92
2.88
4.99
0.97
0.95
3.00
3.00
0.96
2.07
2.12
6.10
8.97
10
8
6
4
2
0 [ppm]

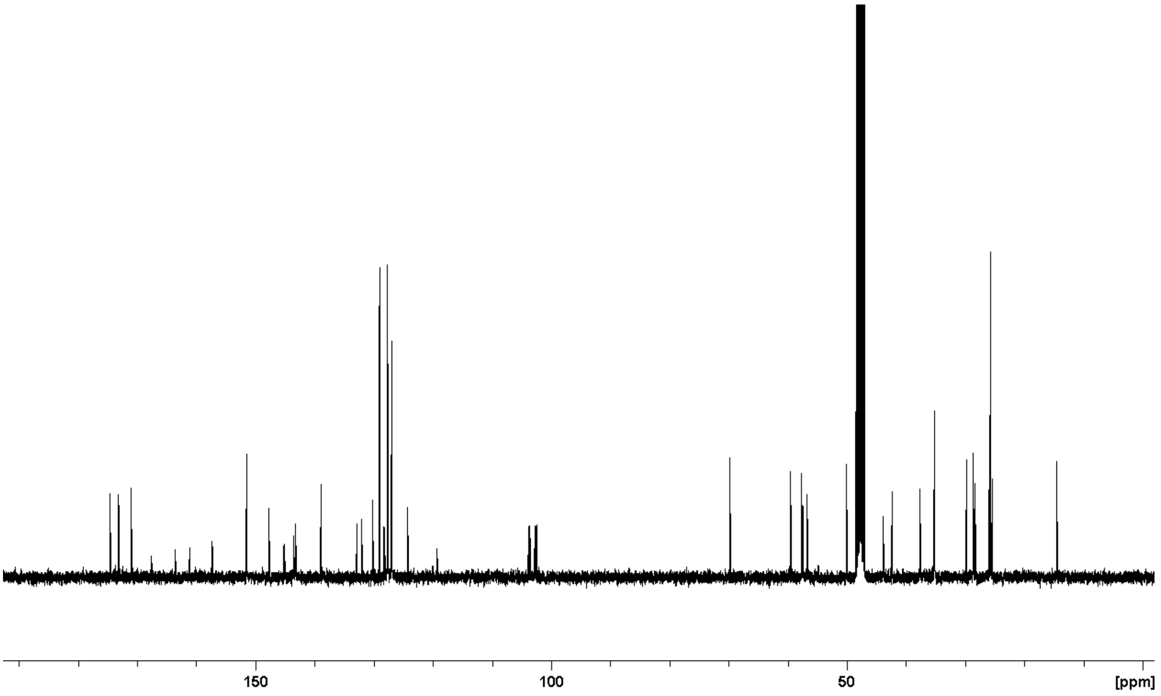
150
100
50
[ppm]

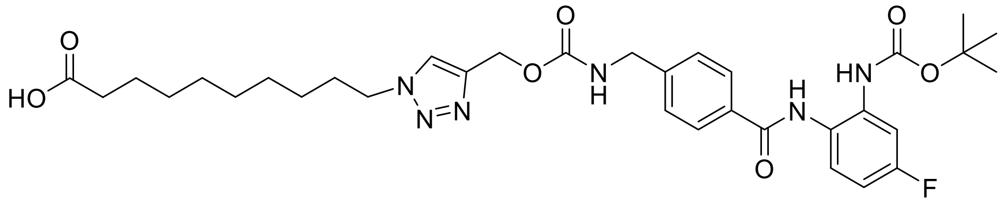

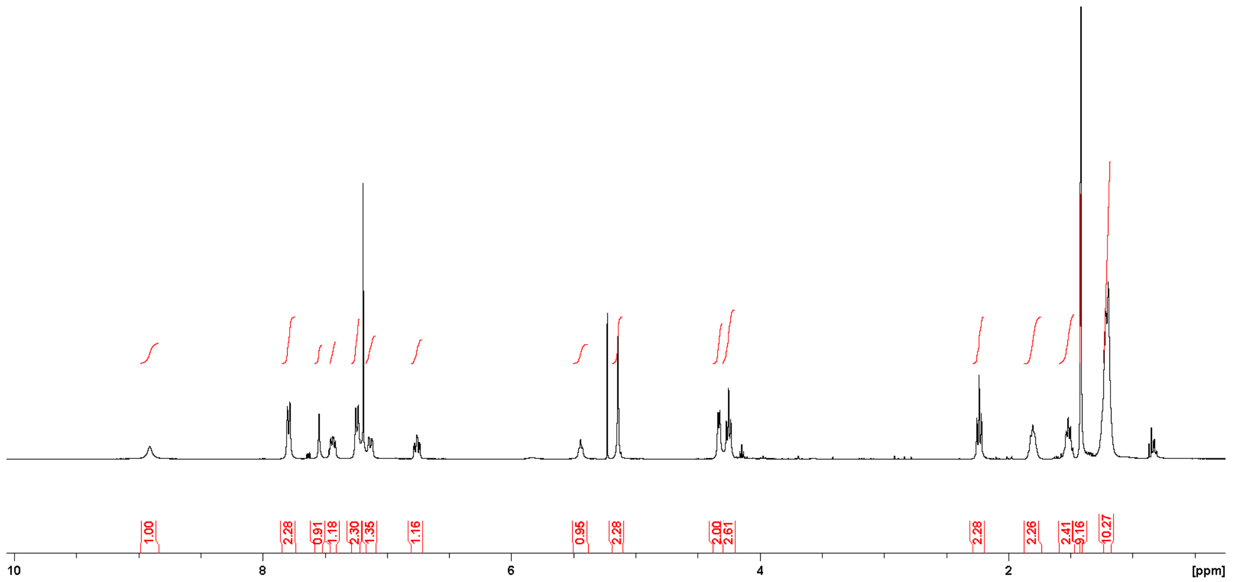

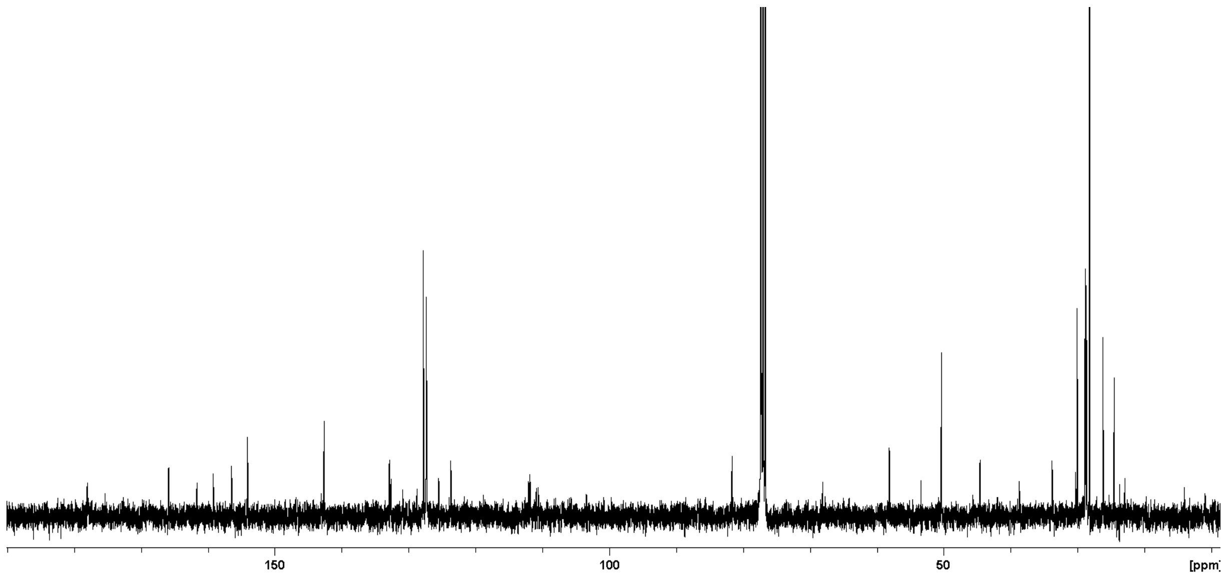

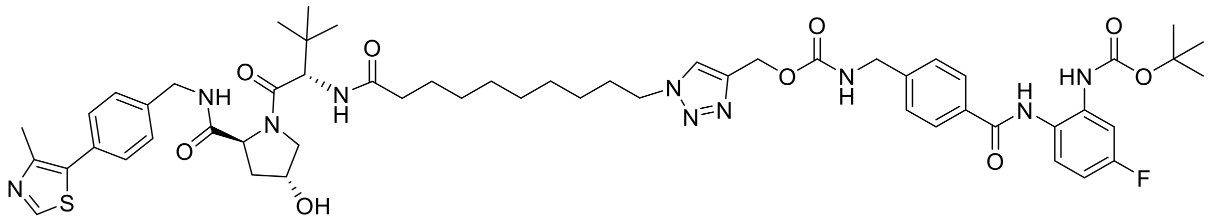

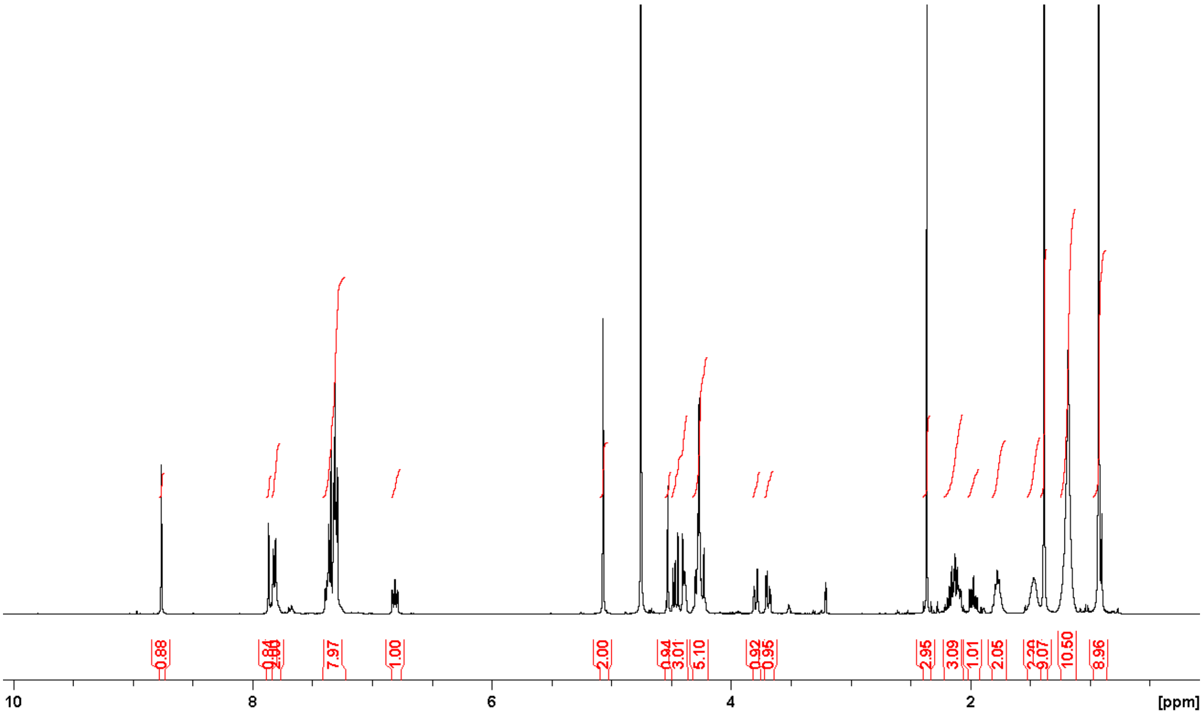

0.88
7.97
1.00
2.00
3.01
5.10
0.92
0.95
2.95
3.09
1.01
2.05
9.07
10.50
8.96
10
8
6
4
2
[ppm]

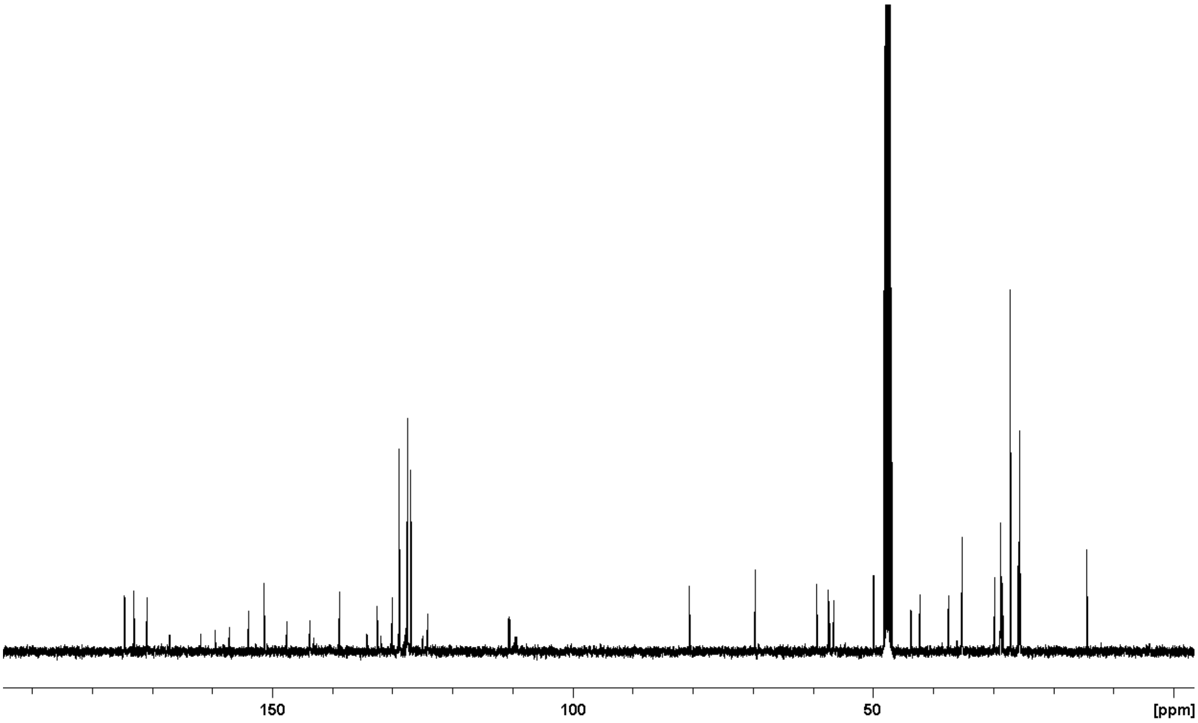

150
100
50
[ppm]

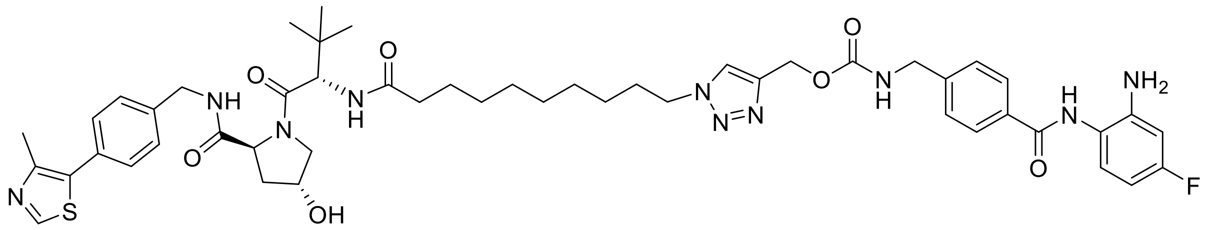
NH
O
N
H
O
N
OH
N
S
N
N=N
O
O
N
H
H
N
NH2
O
F

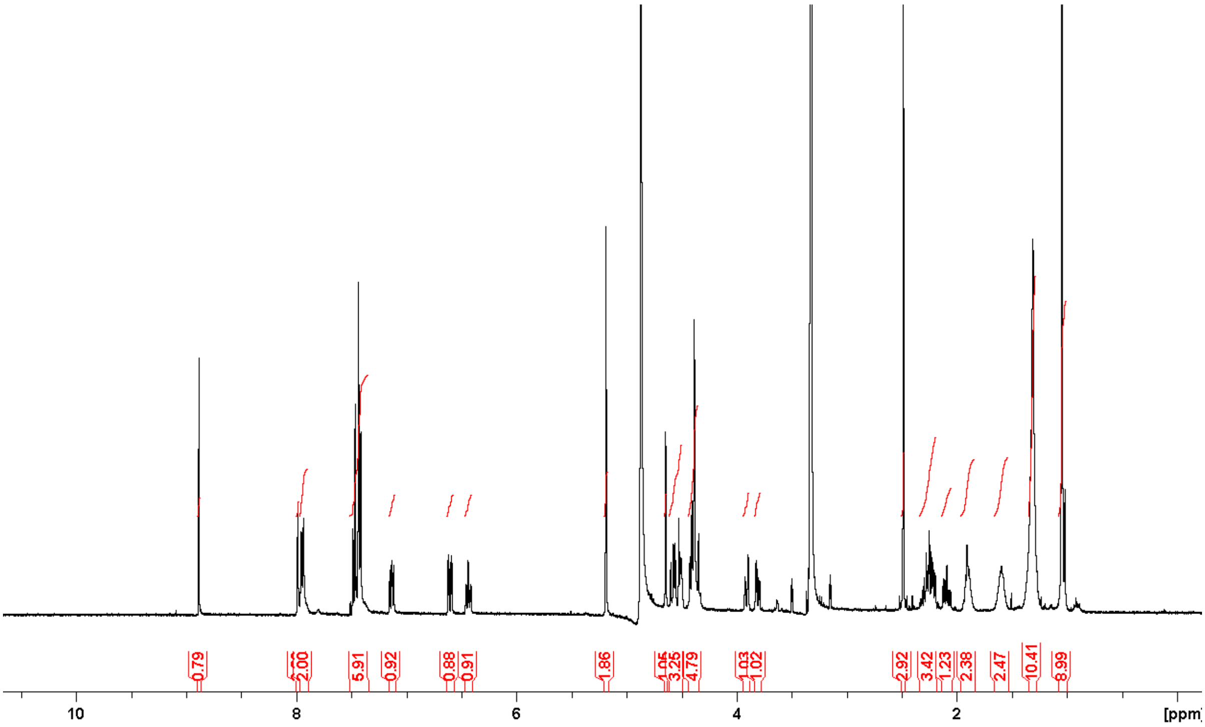
0.79
2.00
5.91
0.92
0.88
0.91
1.86
1.06
3.25
4.79
1.03
1.02
2.92
3.42
1.23
2.38
2.47
10.41
8.99
10
8
6
4
2
[ppm]

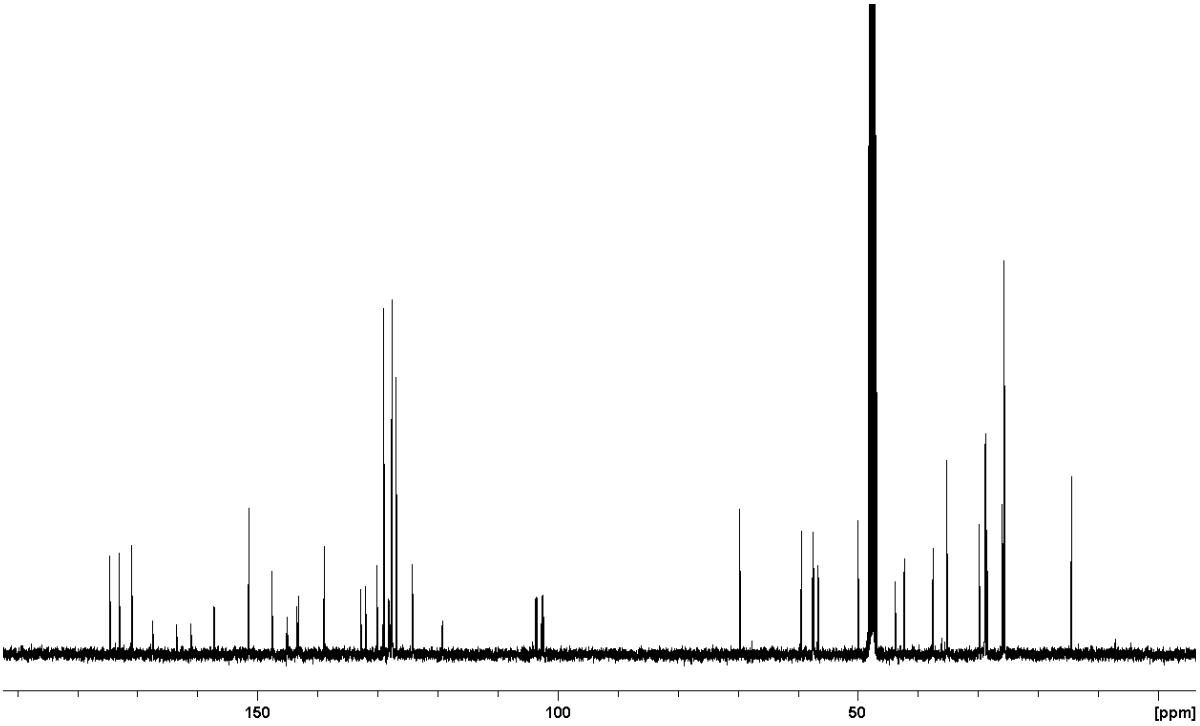
150
100
50
[ppm]

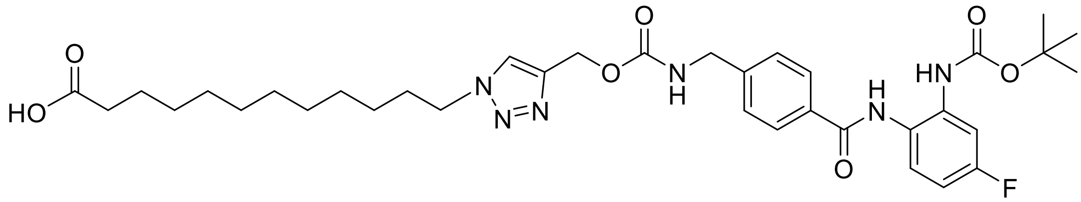
HO
O
N
N=N
O
N
H
O
H
N
O
HN
O
O
F

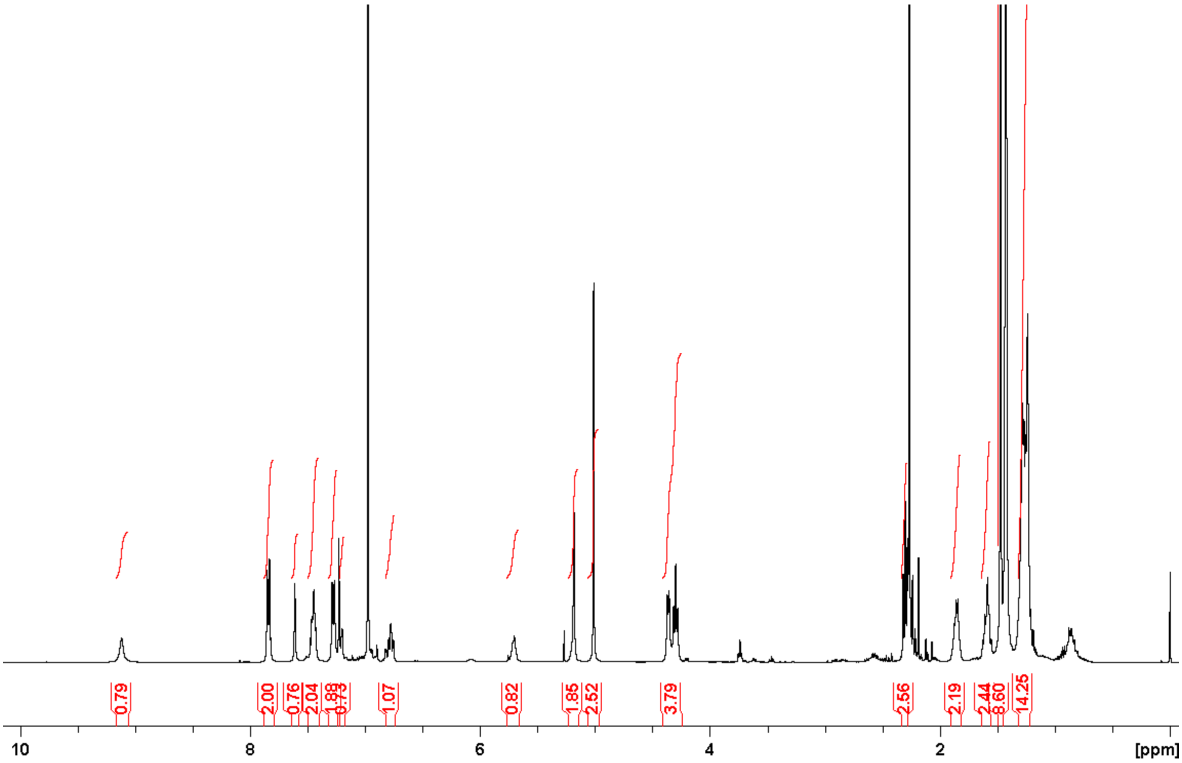
0.79
2.00
0.76
2.04
1.98
0.73
1.07
0.82
1.85
2.52
3.79
2.56
2.19
2.44
8.60
14.25
10
8
6
4
2
[ppm]

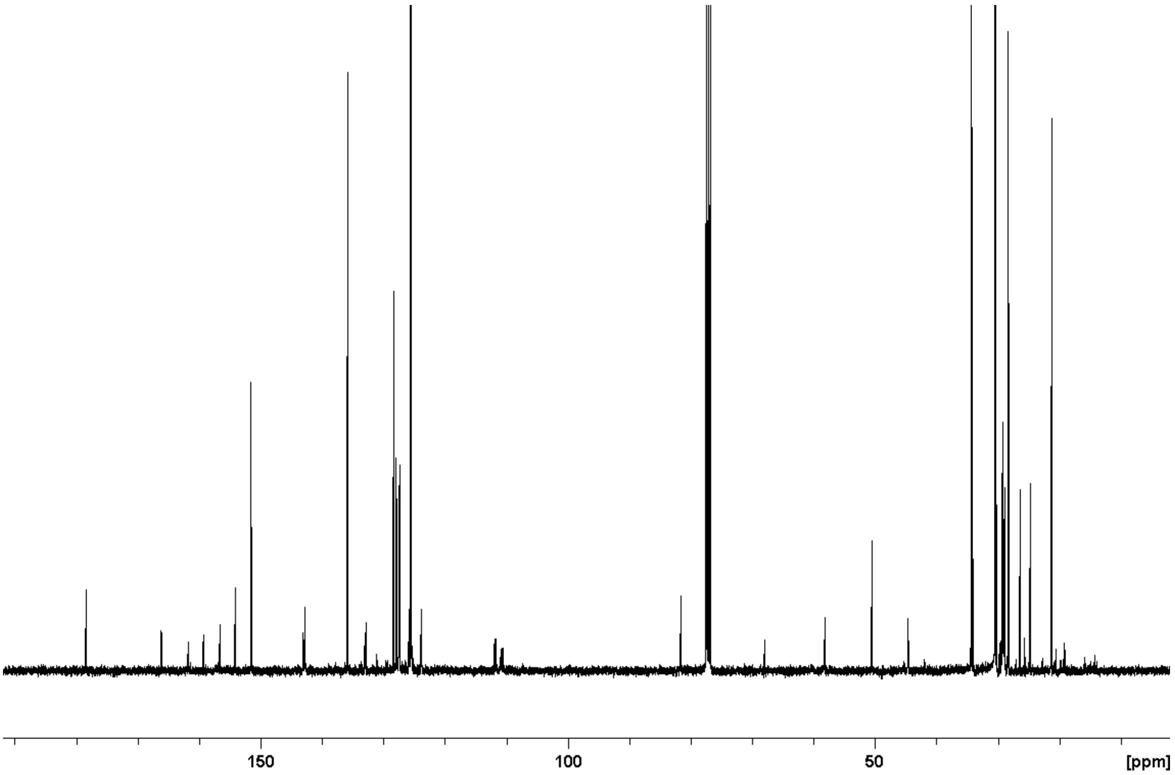
150
100
50
[ppm]

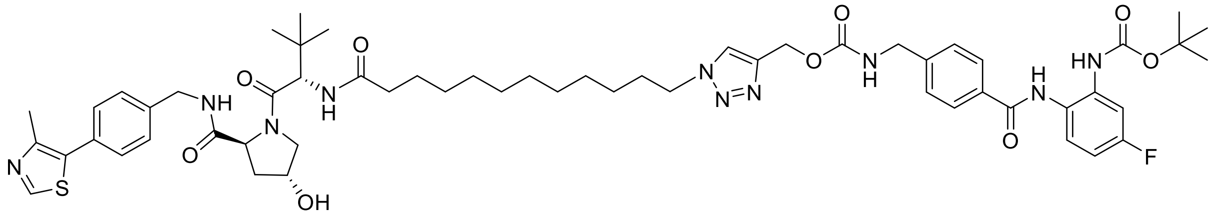

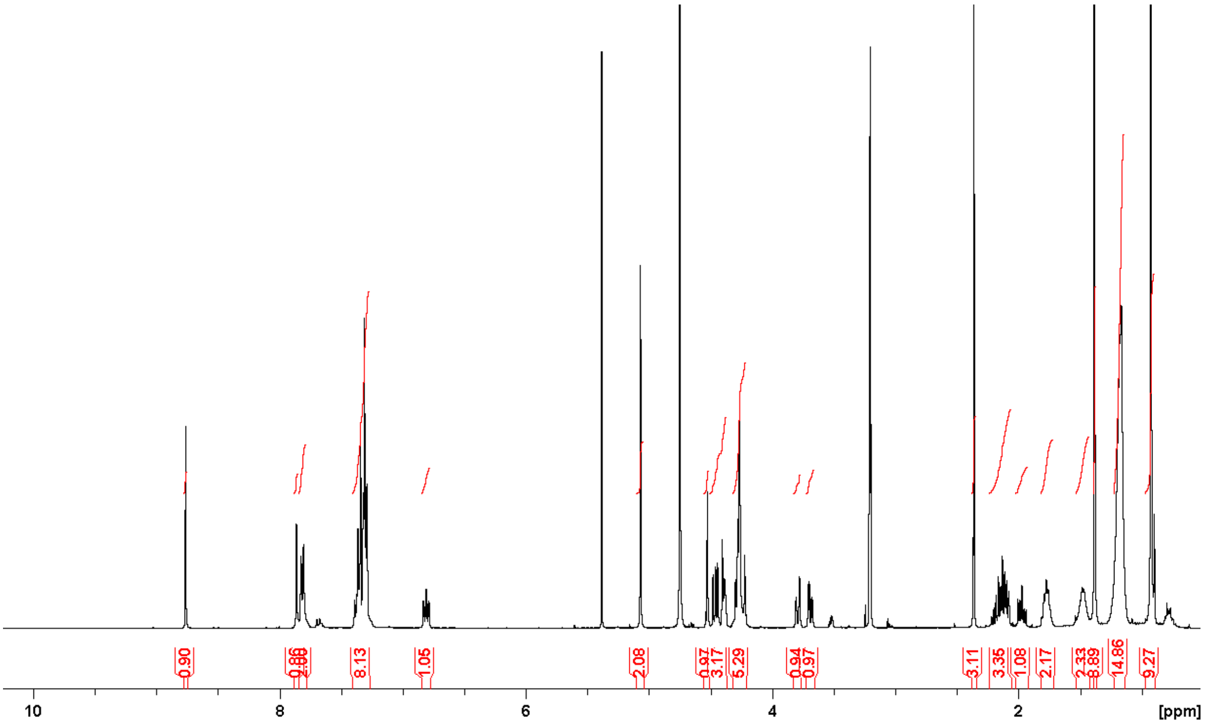
10
8
6
4
2
[ppm]

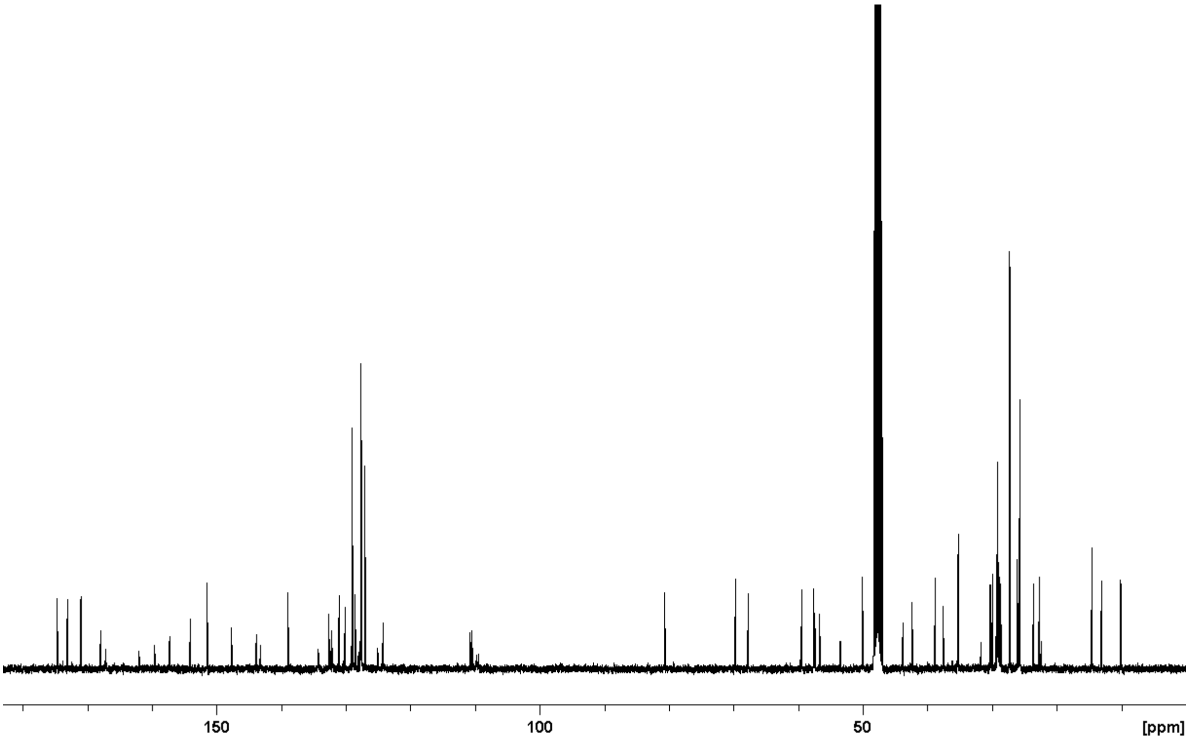
150
100
50
[ppm]

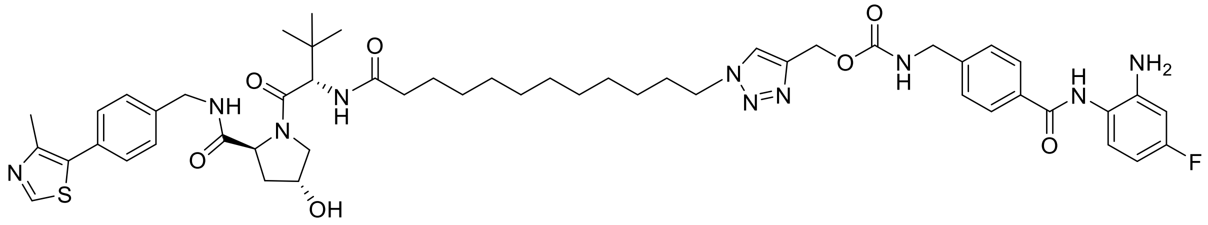

NH
O
N
OH
H
N
O
N
N=N
O
O
N
H
H
N
NH2
O
F
N
S

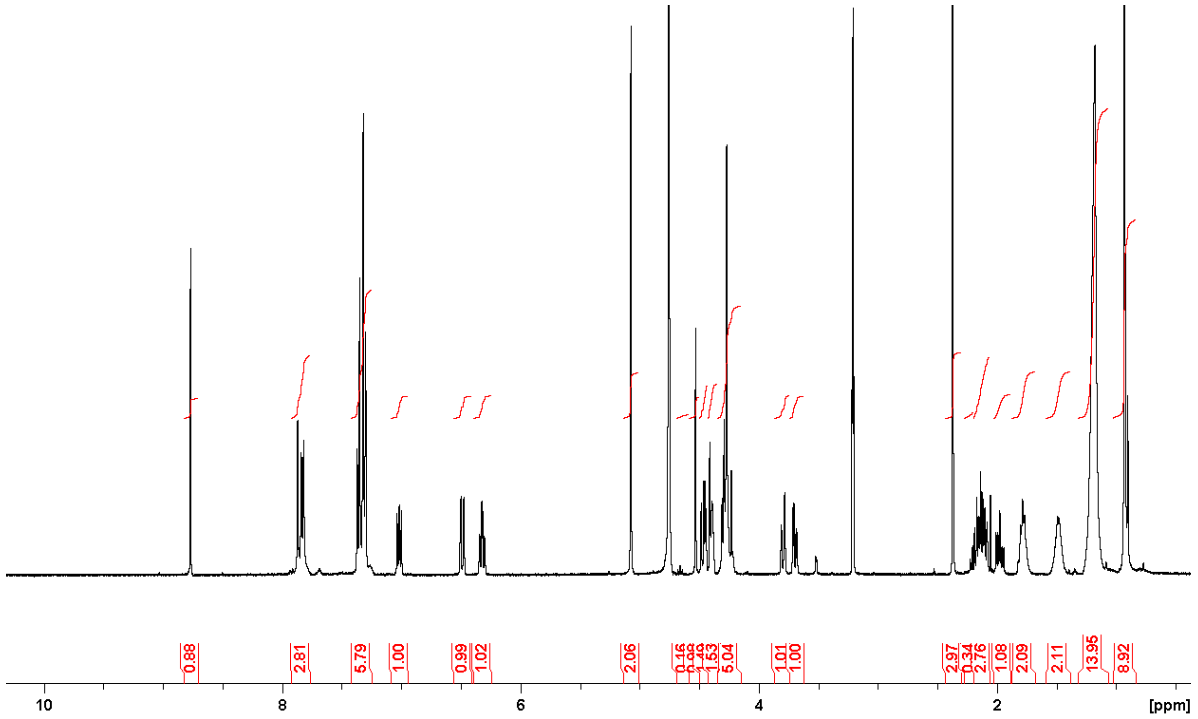

0.88
2.81
5.79
1.00
0.99
1.02
2.06
5.04
1.01
1.00
2.97
2.76
1.08
2.09
2.11
13.95
8.92
10
8
6
4
2
[ppm]

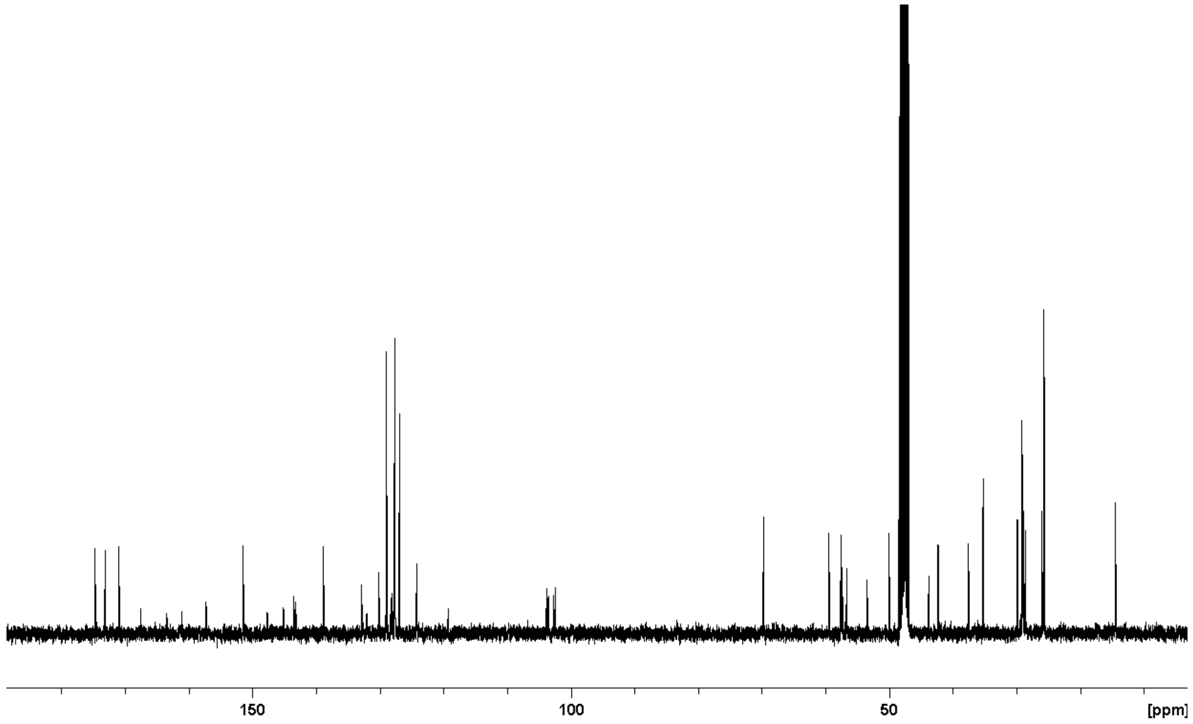

150
100
50
[ppm]

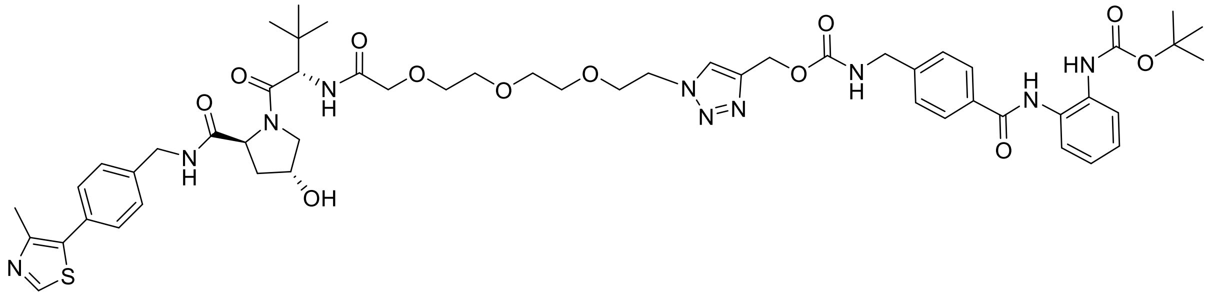

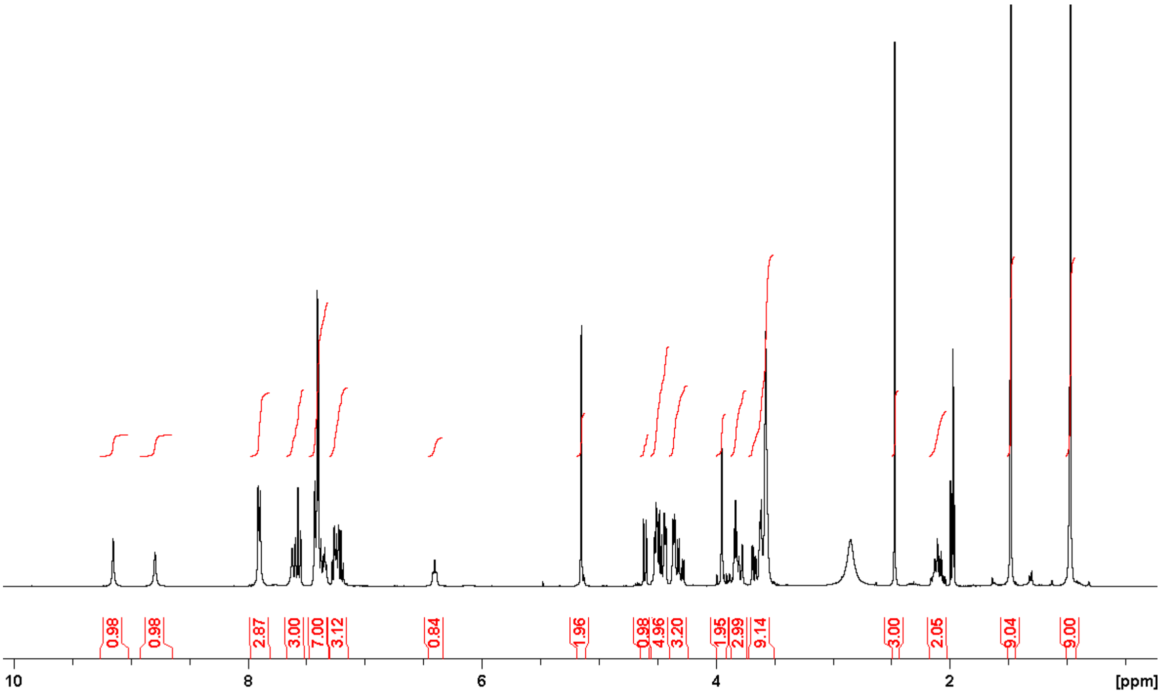
0.98
0.98
2.87
3.00
7.00
3.12
0.84
1.96
0.98
4.96
3.20
1.95
2.99
9.14
3.00
2.05
9.04
9.00
10
8
6
4
2
[ppm]

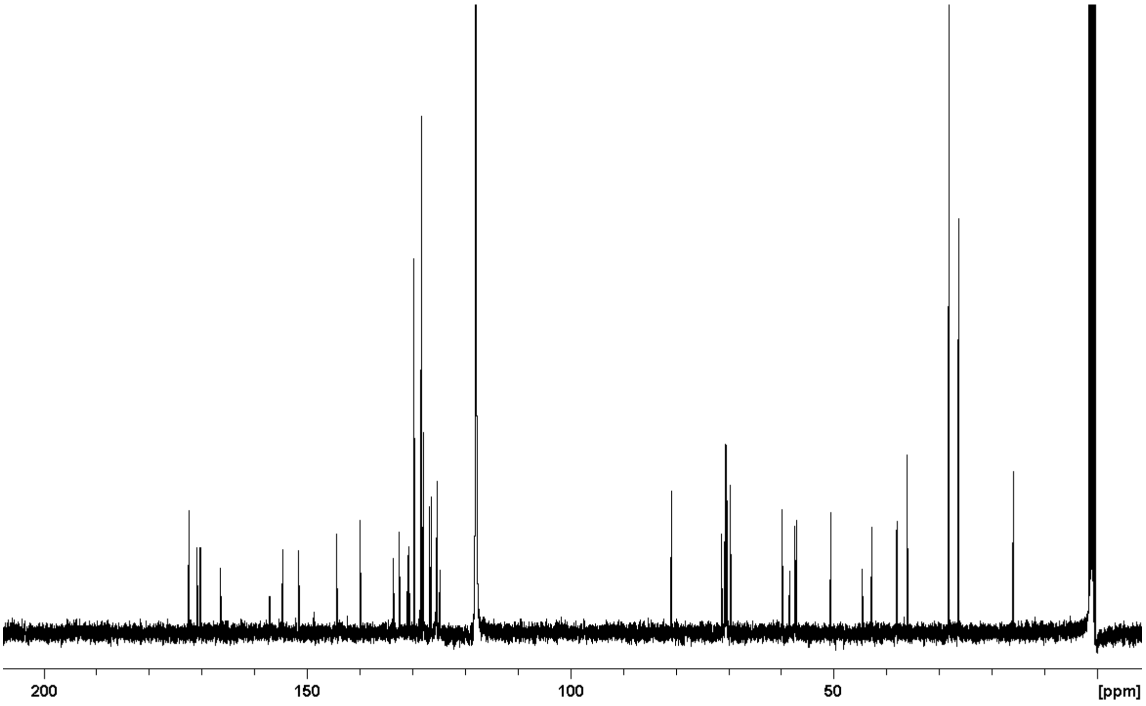
200
150
100
50
[ppm]

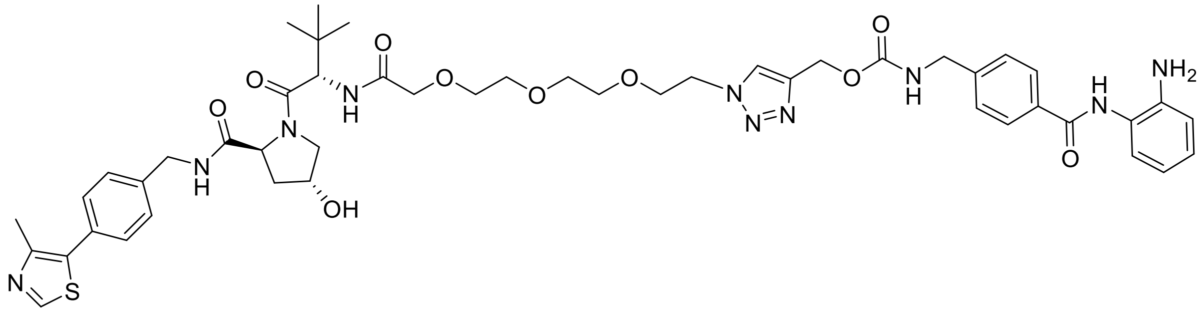
O
N
H
OH
N
S
NH2
N
N=N

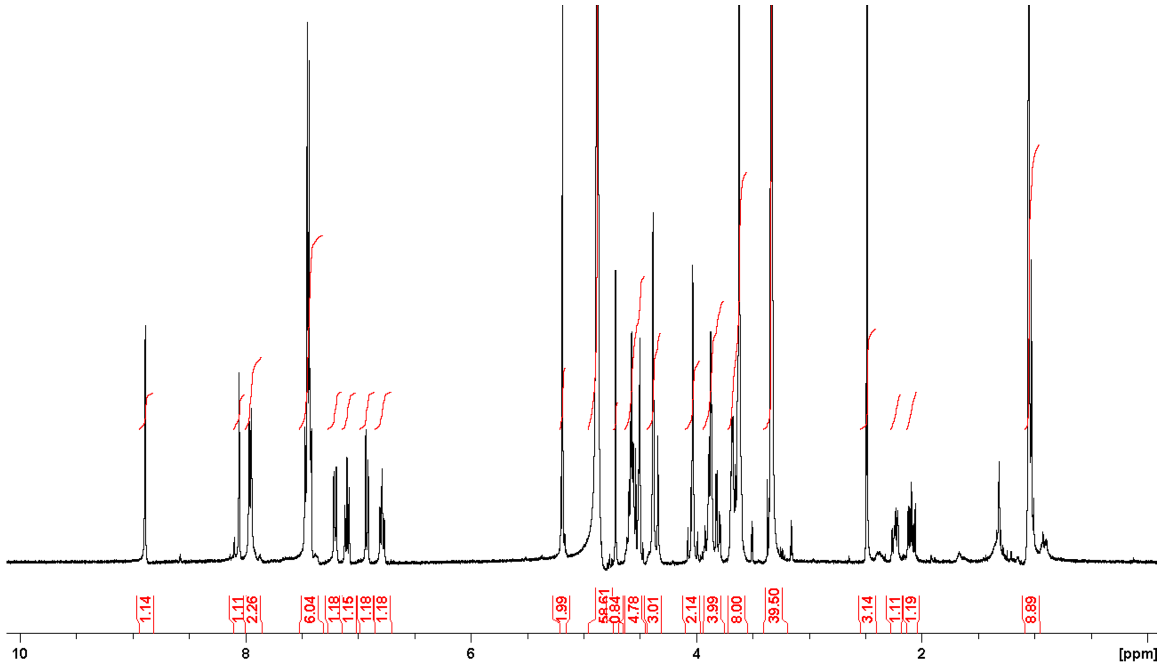
1.14
1.11
2.26
6.04
1.18
1.15
1.18
1.18
1.99
59.61
0.84
4.78
3.01
2.14
3.99
8.00
39.50
3.14
1.11
1.19
8.89
10
8
6
4
2
[ppm]

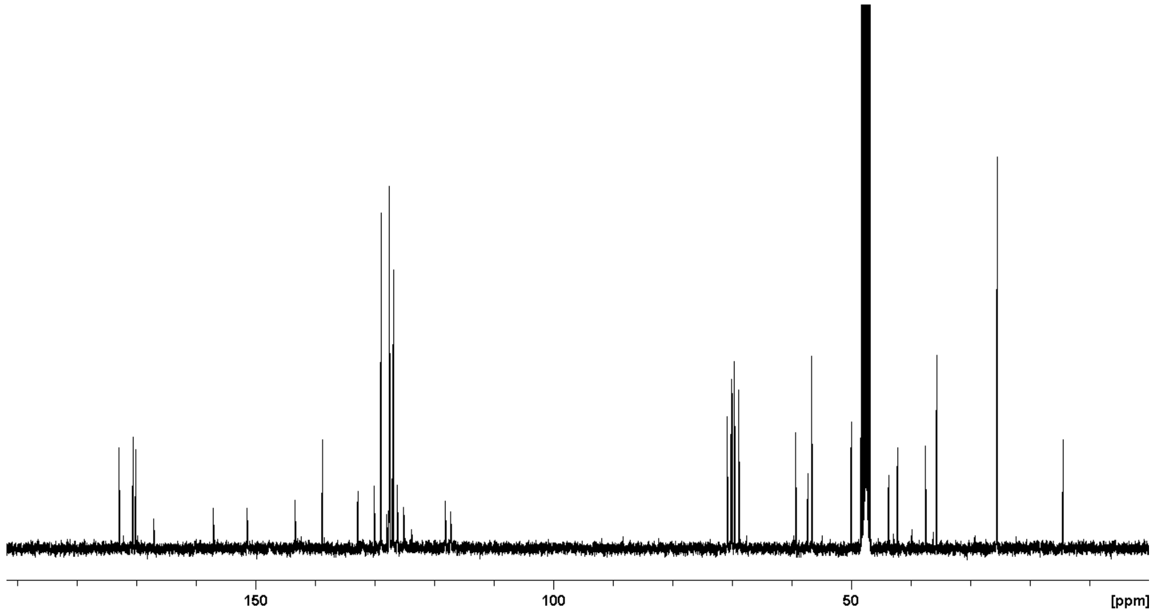
150
100
50
[ppm]

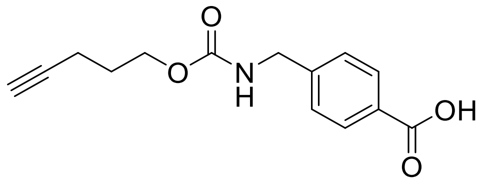
O
O
N
H
OH
O

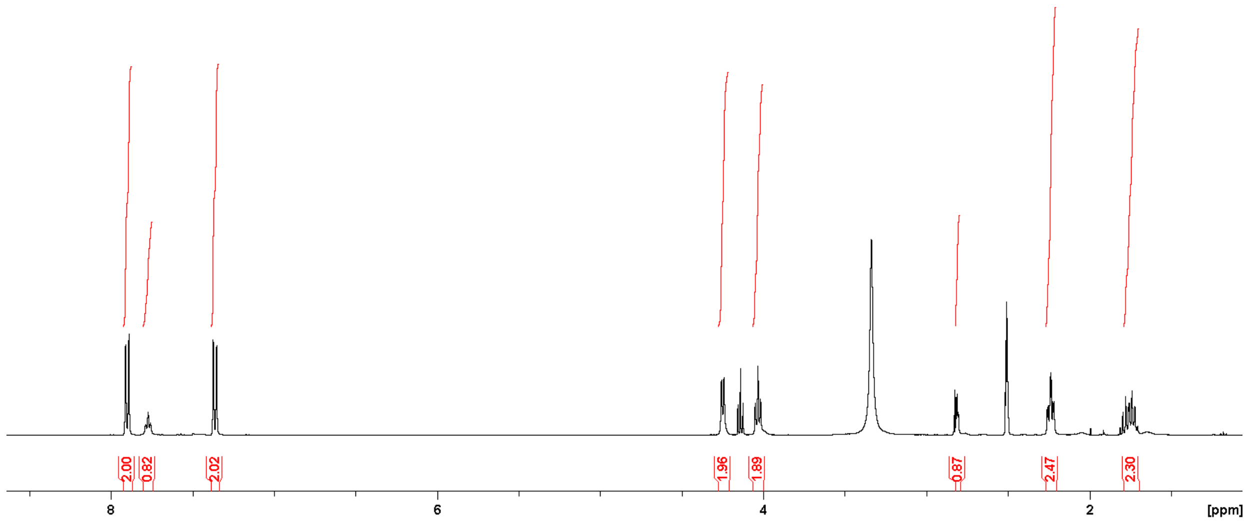
2.00
0.82
2.02
1.96
1.89
0.87
2.47
2.30
8
6
4
2
[ppm]

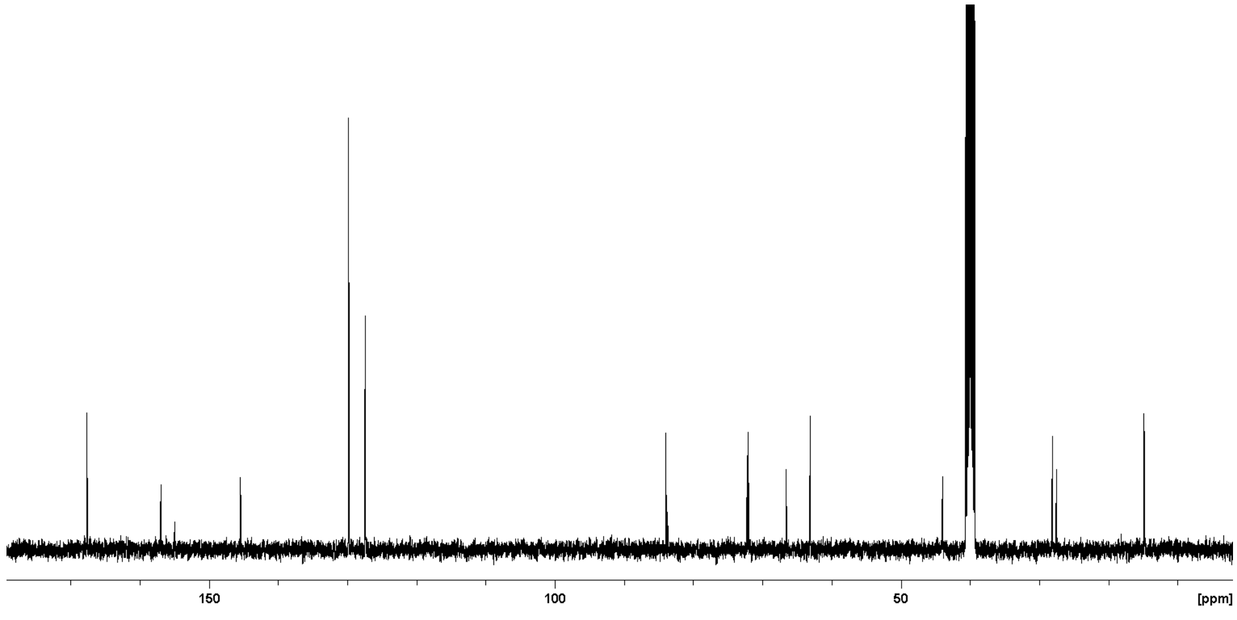
150
100
50
[ppm]

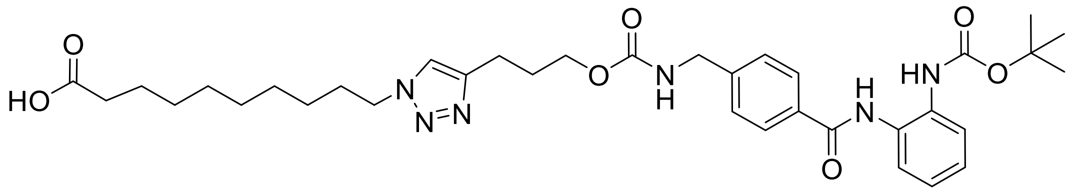

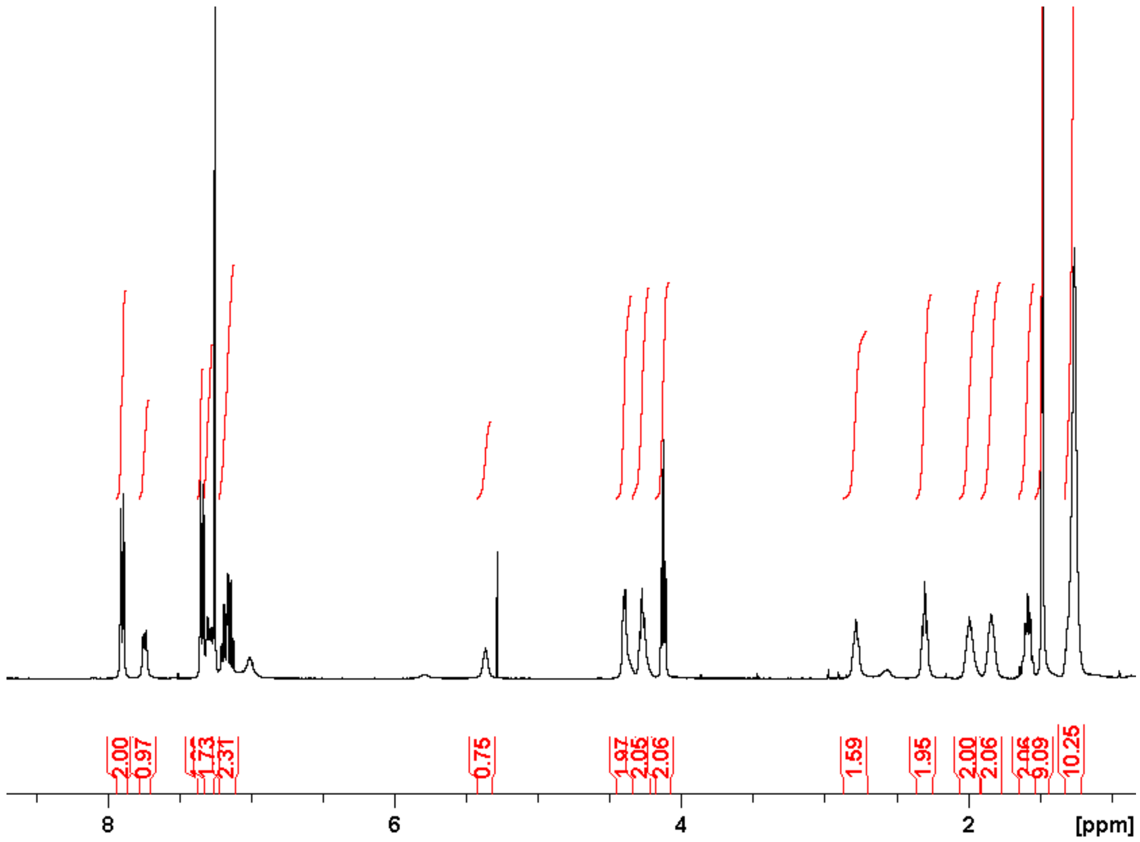
2.00
0.97
1.73
2.31
0.75
1.97
2.05
2.06
1.59
1.95
2.00
2.06
9.09
10.25
8
6
4
2
[ppm]

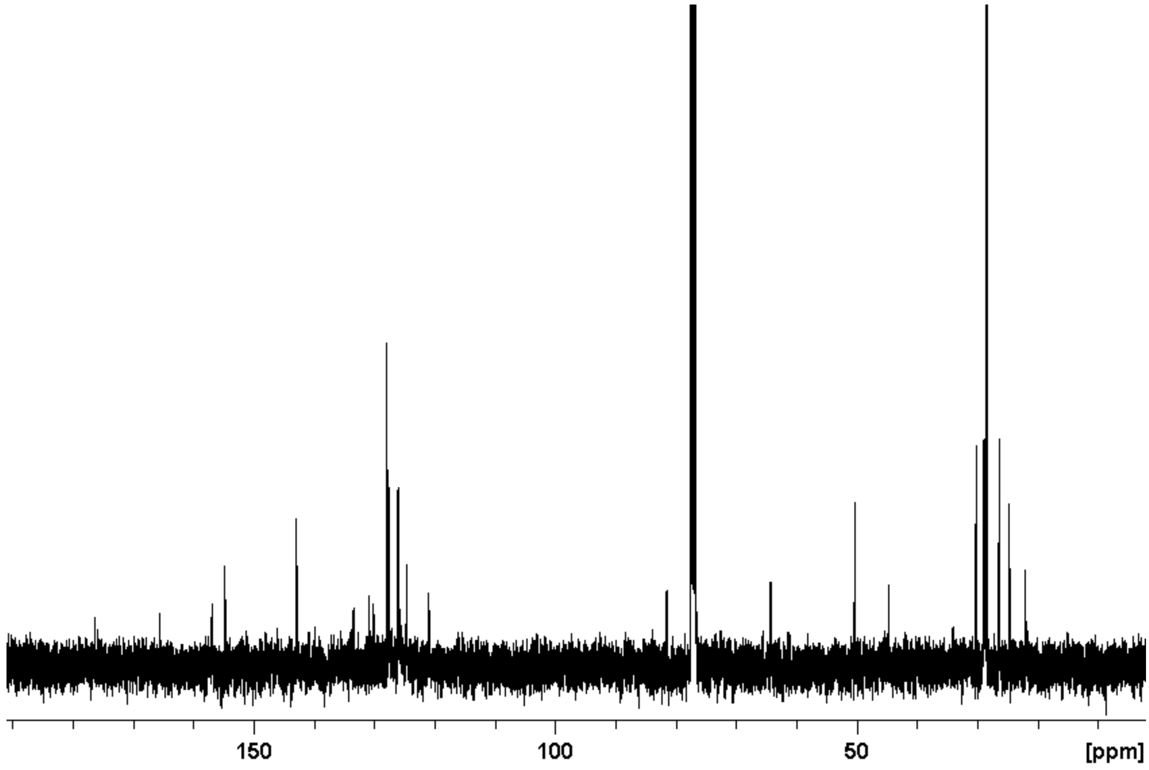
150
100
50
[ppm]

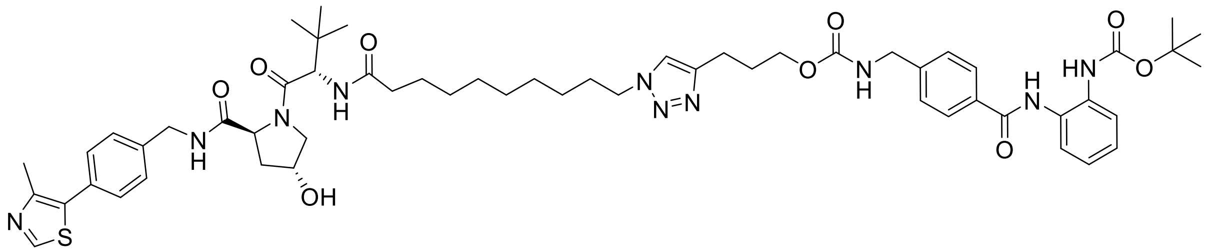

O
N
H
N
OH
S
HN
O

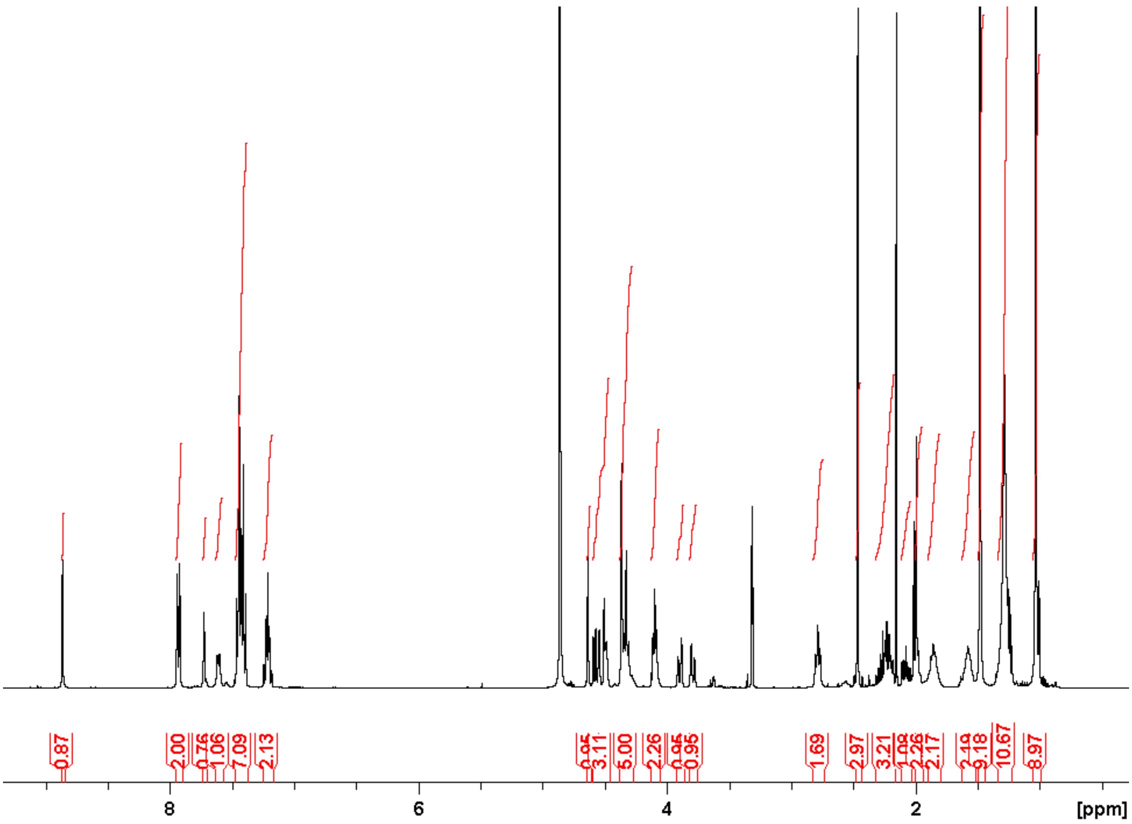

8
6
4
2
[ppm]

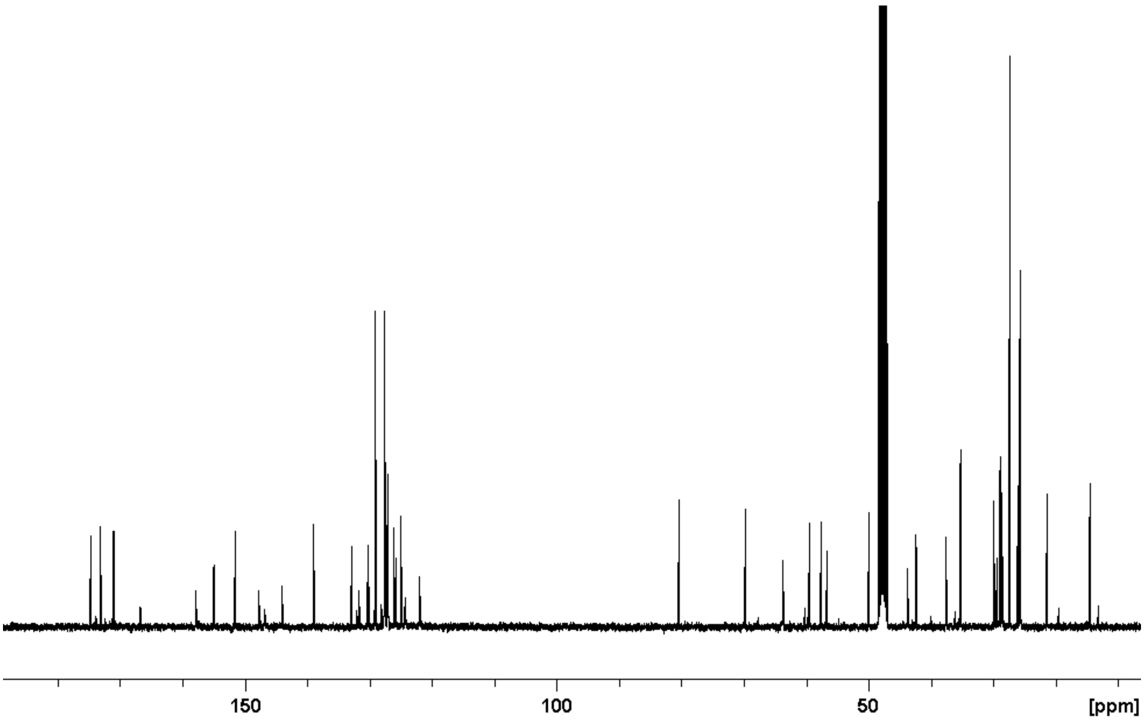

150
100
50
[ppm]

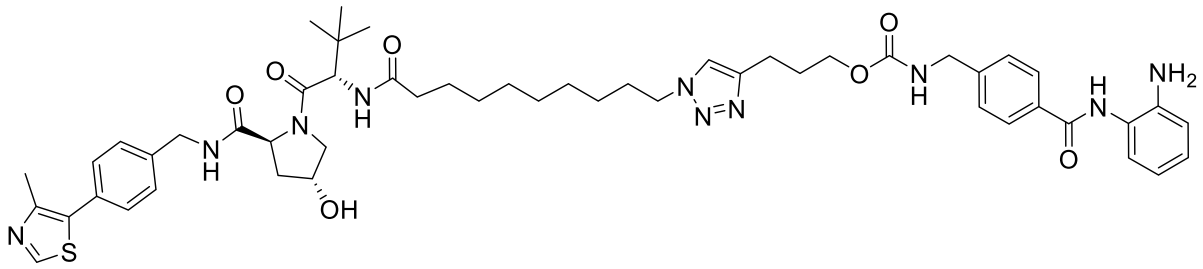

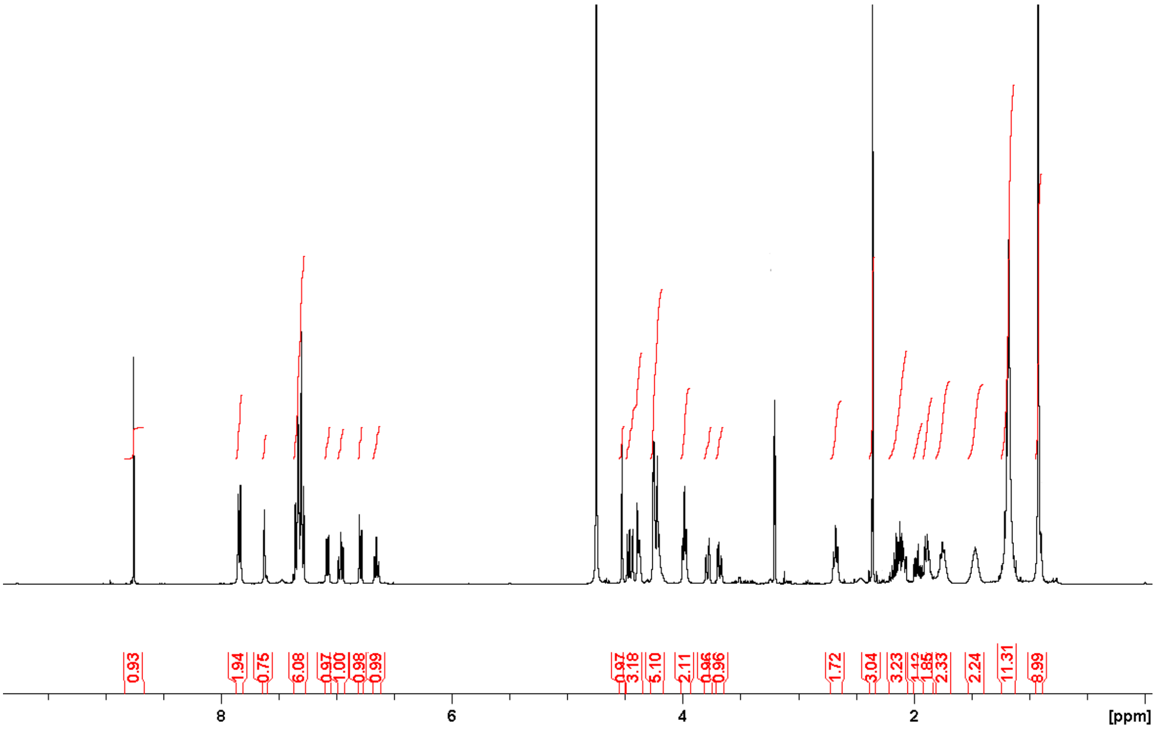

0.93
1.94
0.75
6.08
0.97
1.00
0.98
0.99
3.18
5.10
2.11
0.96
0.96
1.72
3.04
3.23
1.85
2.33
2.24
11.31
8.99
8
6
4
2
[ppm]

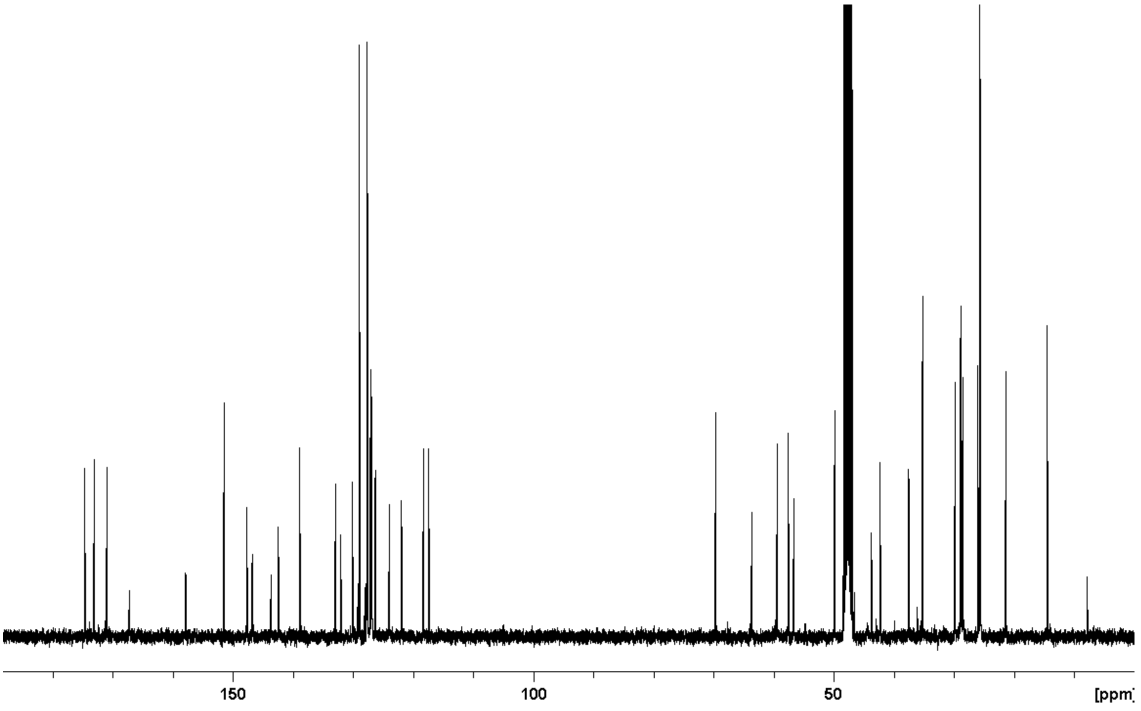

150
100
50
[ppm]

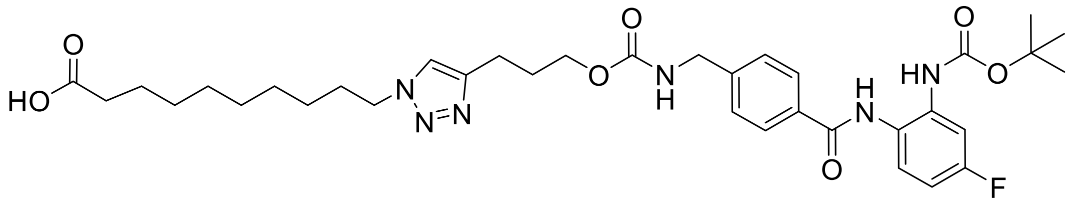

HO
O
N
N
N
O
O
N
H
H
N
O
HN
O
O
F

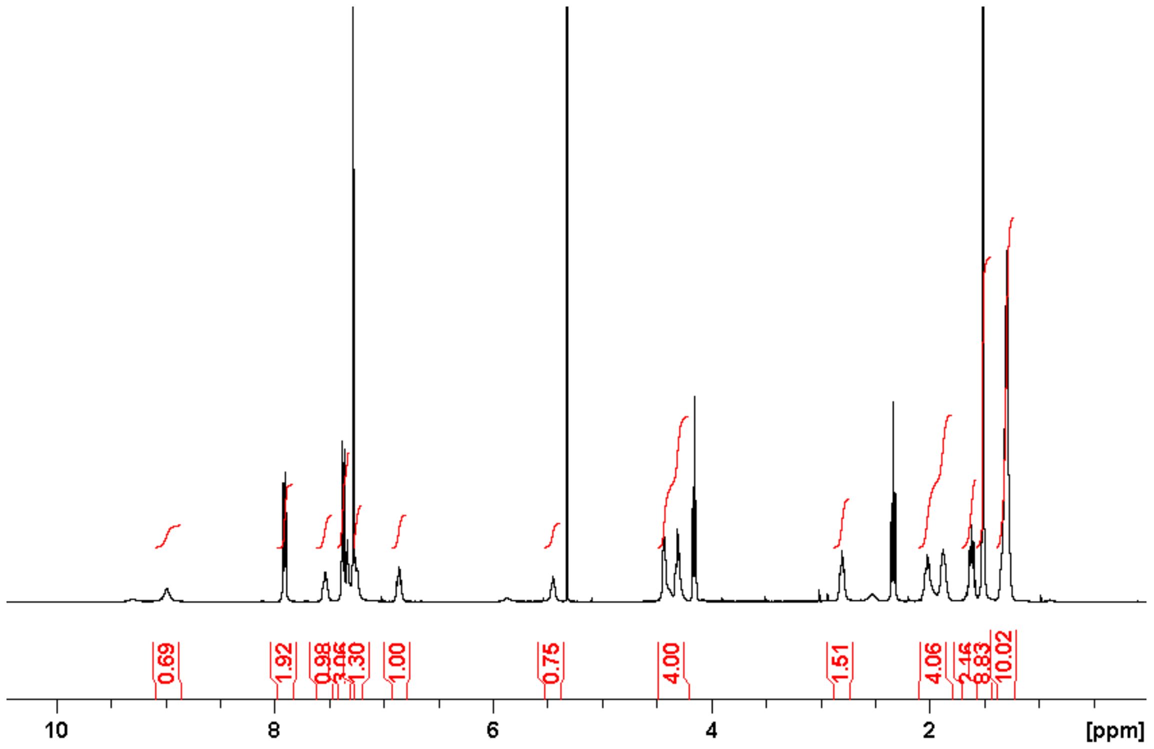

0.69
1.92
0.98
3.06
1.30
1.00
0.75
4.00
1.51
4.06
2.46
8.83
10.02
10
8
6
4
2
[ppm]

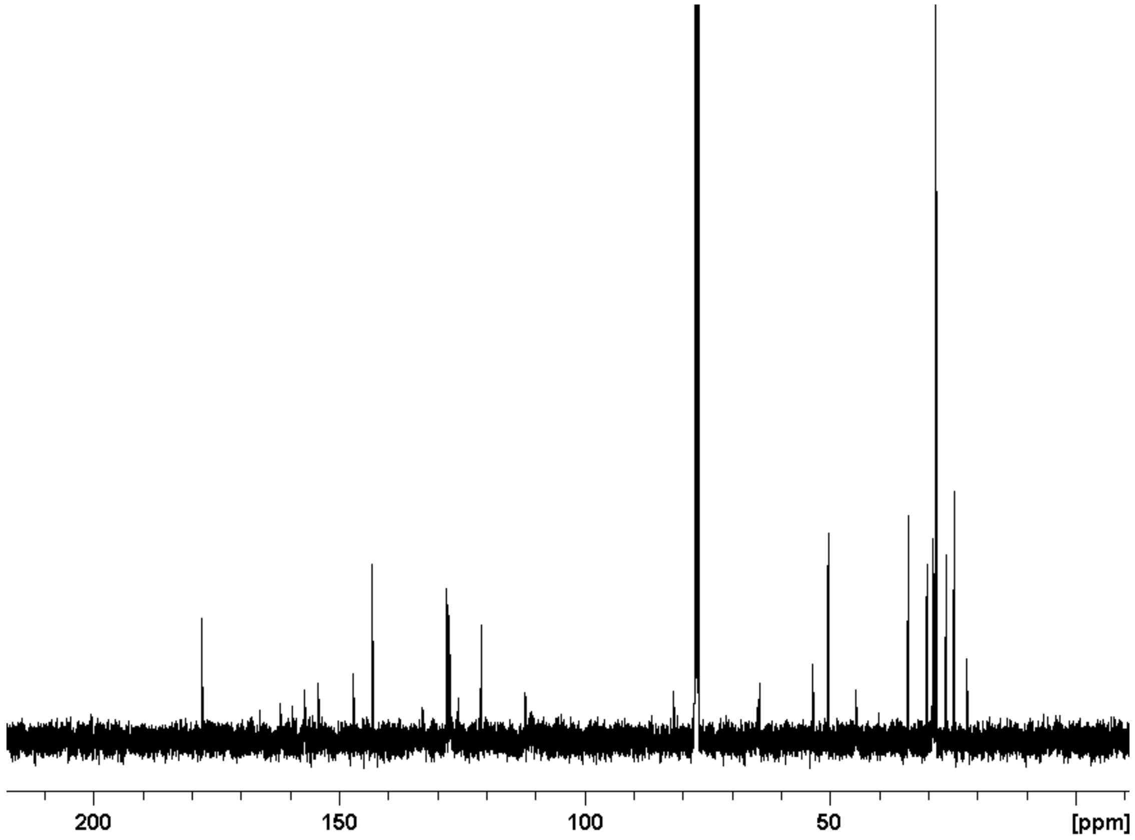

200
150
100
50
[ppm]

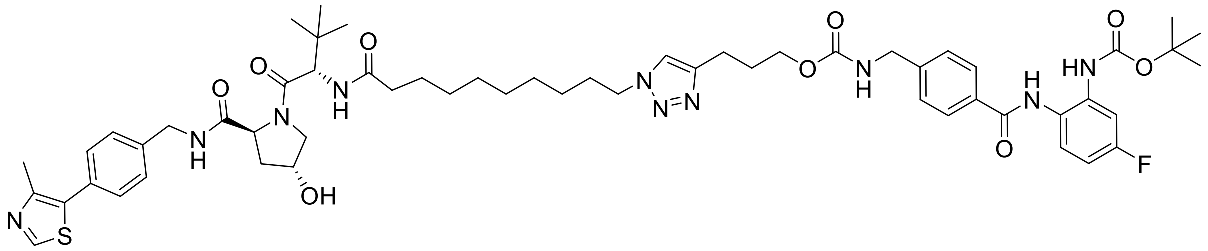

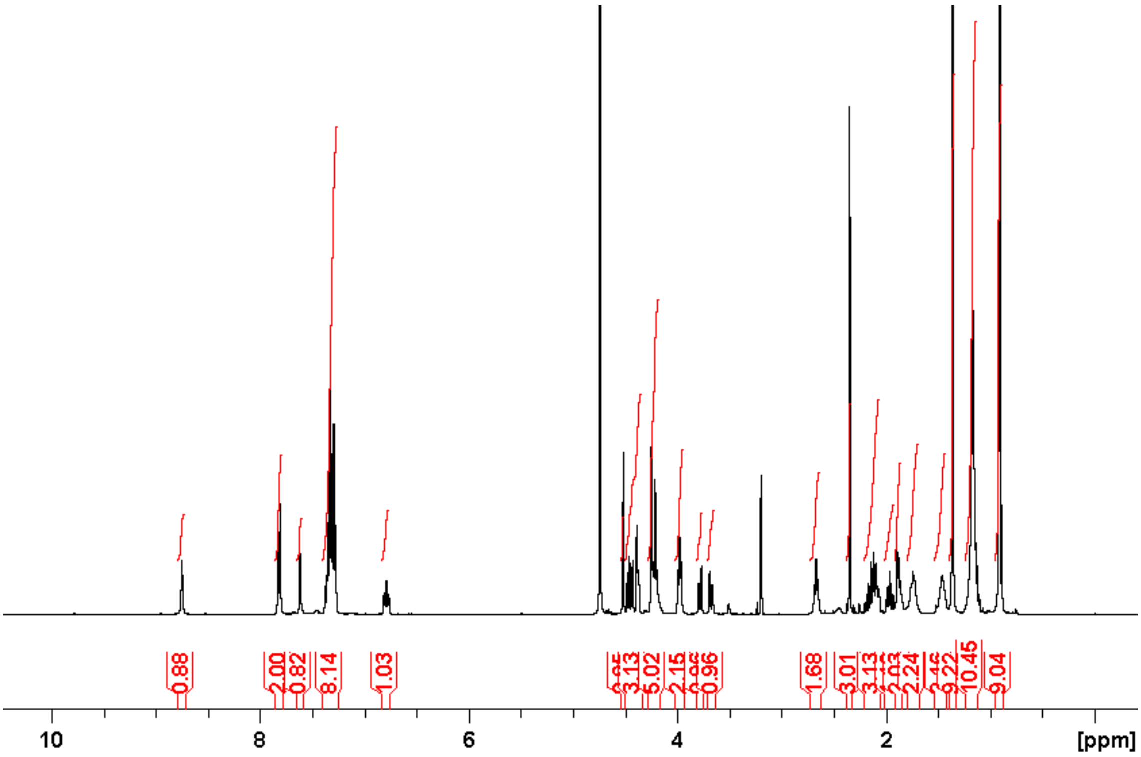
0.88
2.00
0.82
8.14
1.03
3.13
5.02
2.15
0.96
1.68
3.01
3.13
2.24
9.22
10.45
9.04
10
8
6
4
2
[ppm]

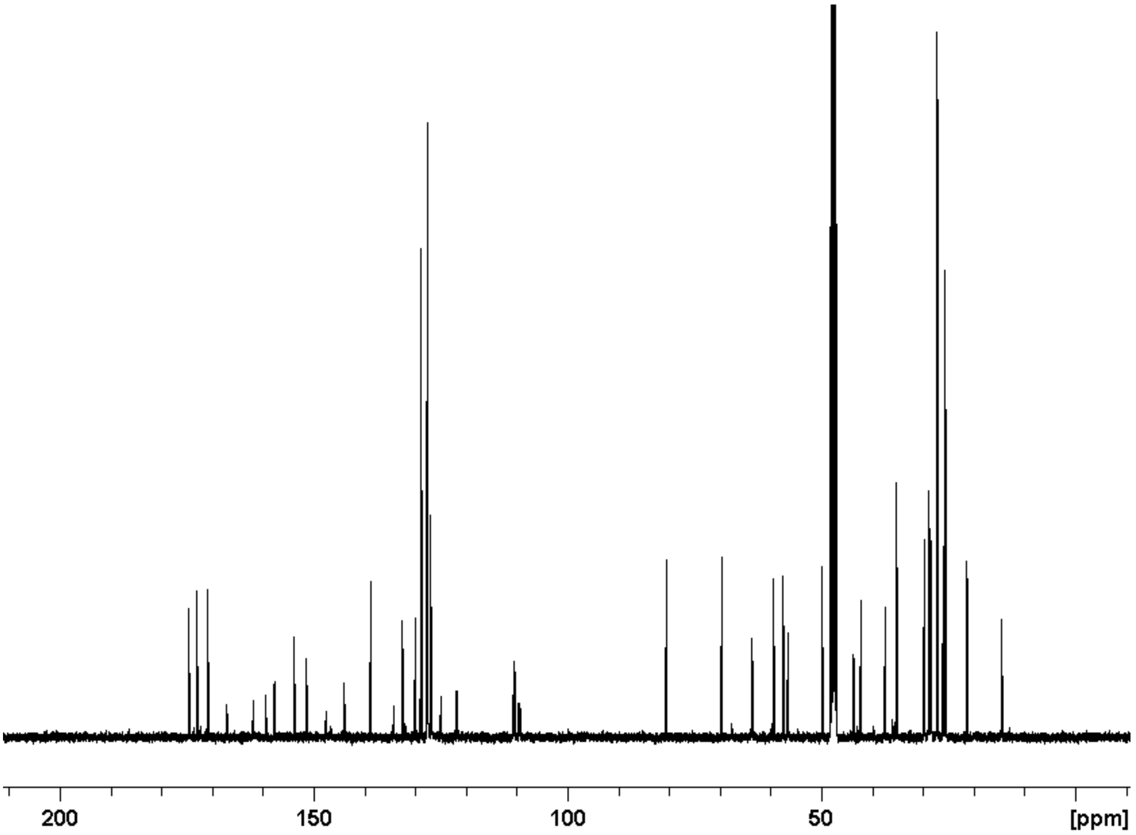
200
150
100
50
[ppm]

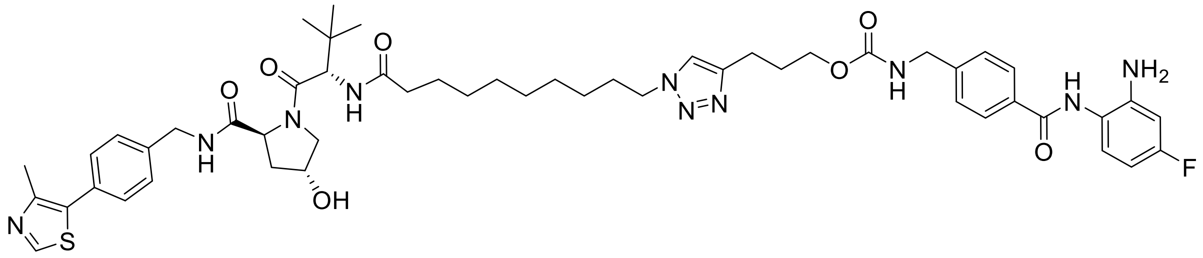
O
O
N
H
N
OH
N
S
N
N=N
NH2
F

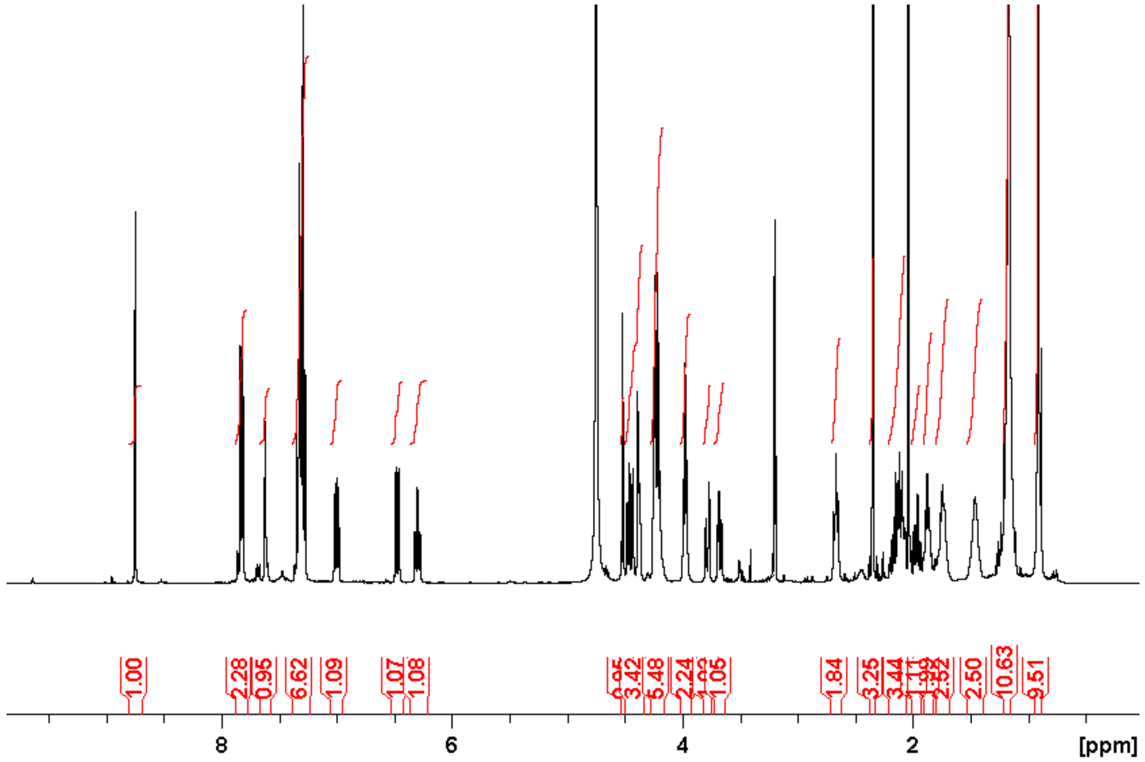
1.00
2.28
0.95
6.62
1.09
1.07
1.08
3.42
5.48
2.24
1.05
1.84
3.25
3.44
2.52
2.50
10.63
9.51
8
6
4
2
[ppm]

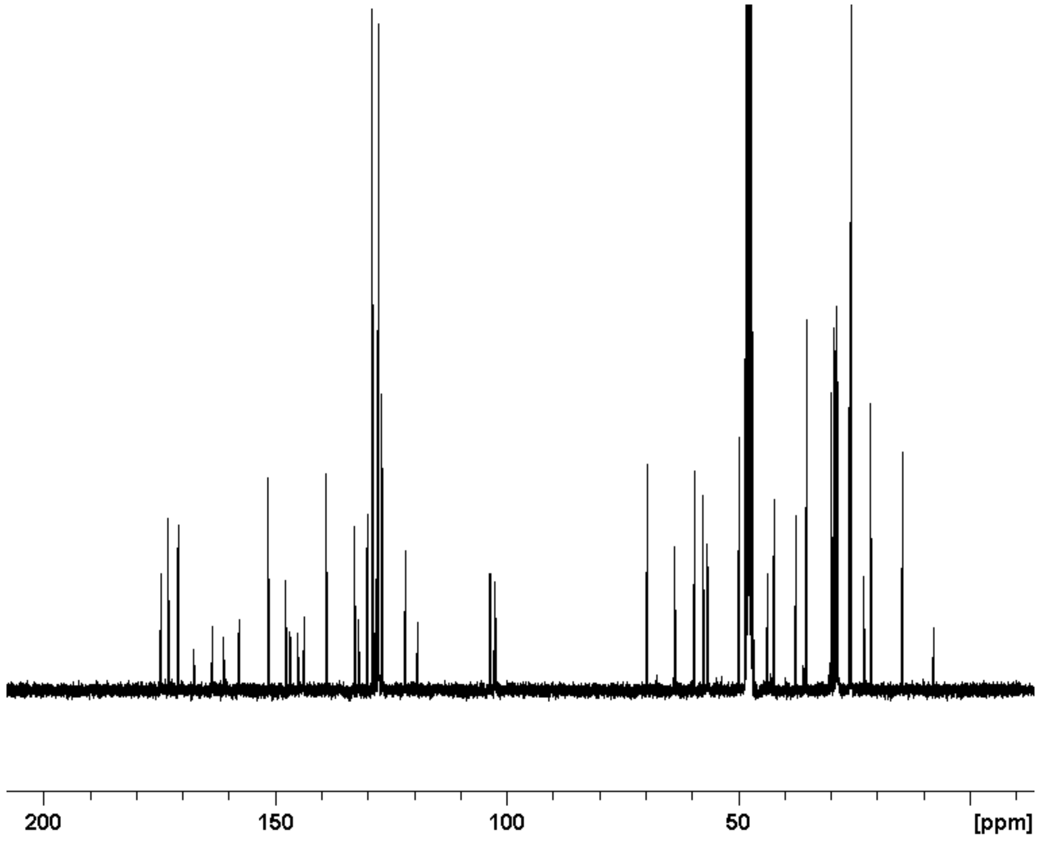
200
150
100
50
[ppm]

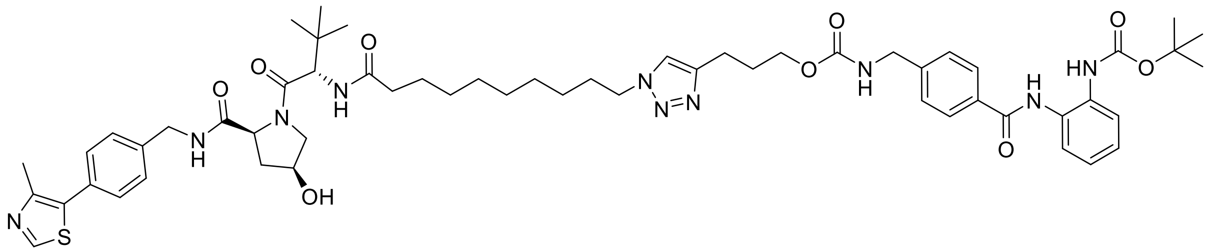

O
N
H
OH
S
HN

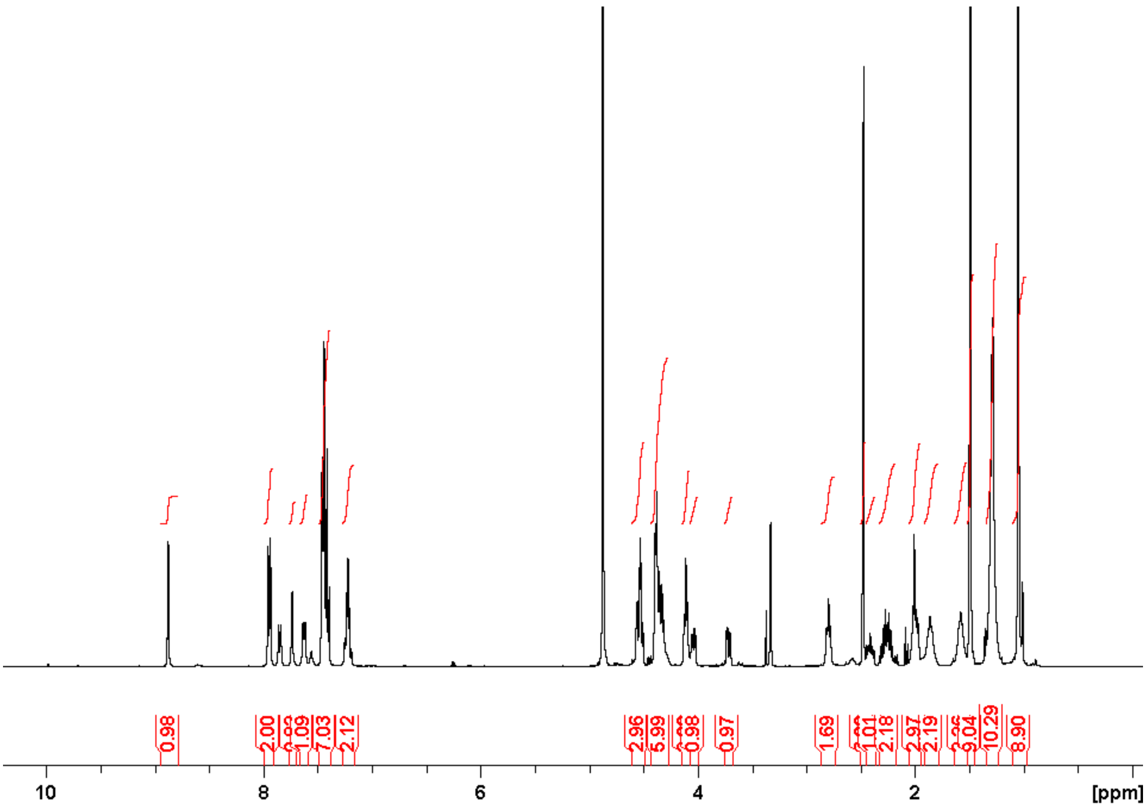

0.98
2.00
1.09
7.03
2.12
2.96
5.99
0.98
0.97
1.69
1.01
2.18
2.97
2.19
9.04
10.29
8.90
10
8
6
4
2
[ppm]

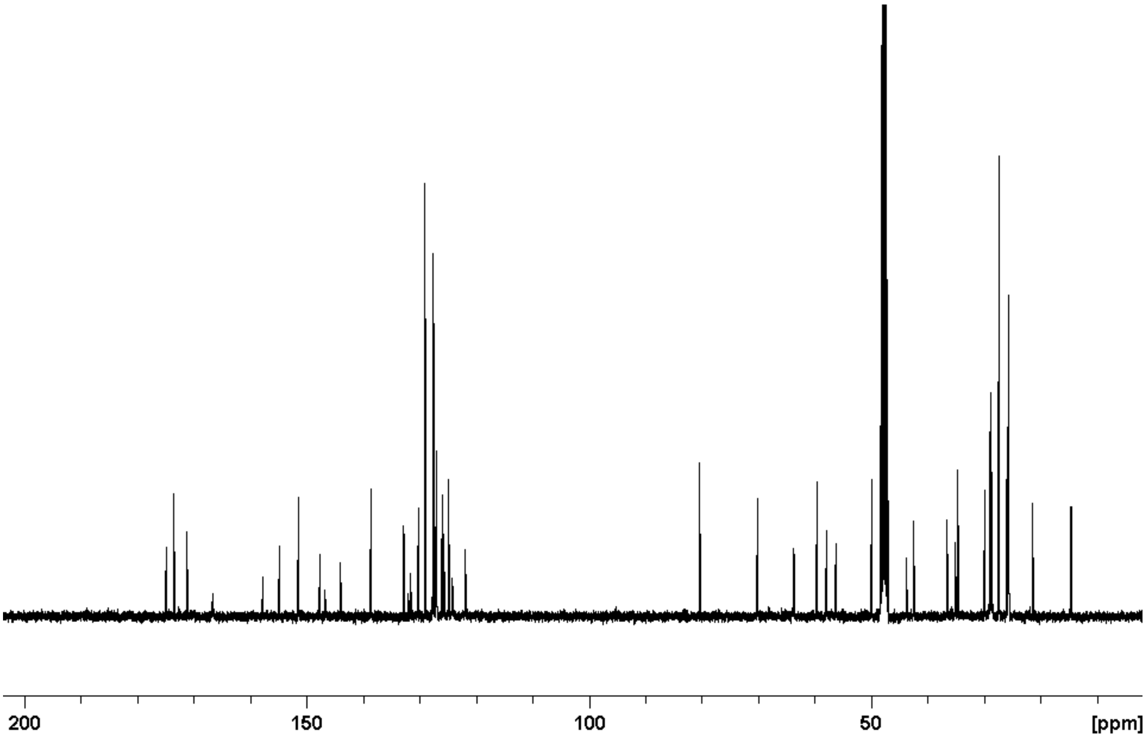

200
150
100
50
[ppm]

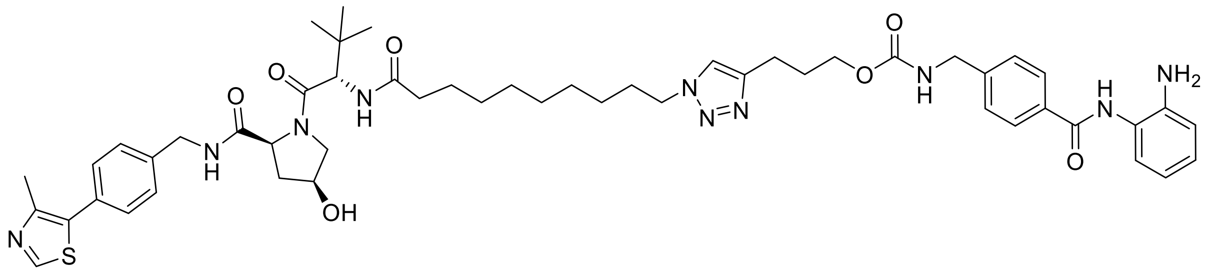
NH2
OH
N
S
O
N
H
O
O
N
H
N
N=N
O
O
N
H
H
N
O

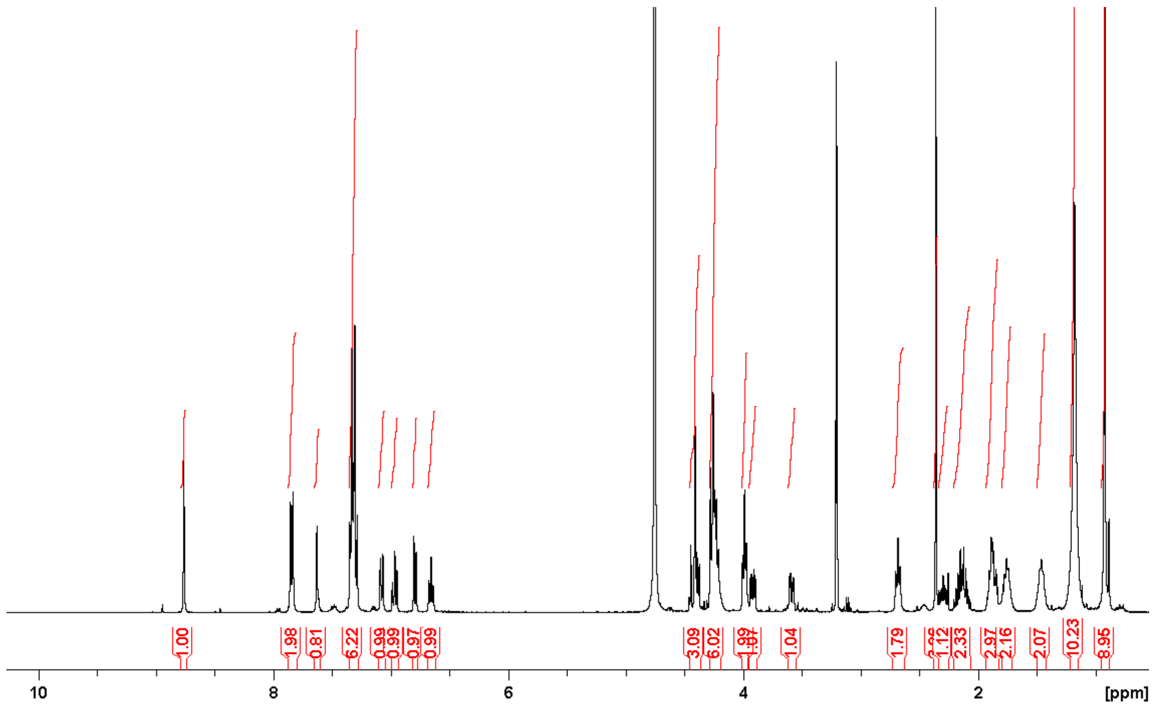
1.00
1.98
0.81
6.22
0.99
0.99
0.97
0.99
3.09
6.02
1.99
1.04
1.79
1.12
2.33
2.97
2.16
2.07
10.23
8.95
10
8
6
4
2
[ppm]

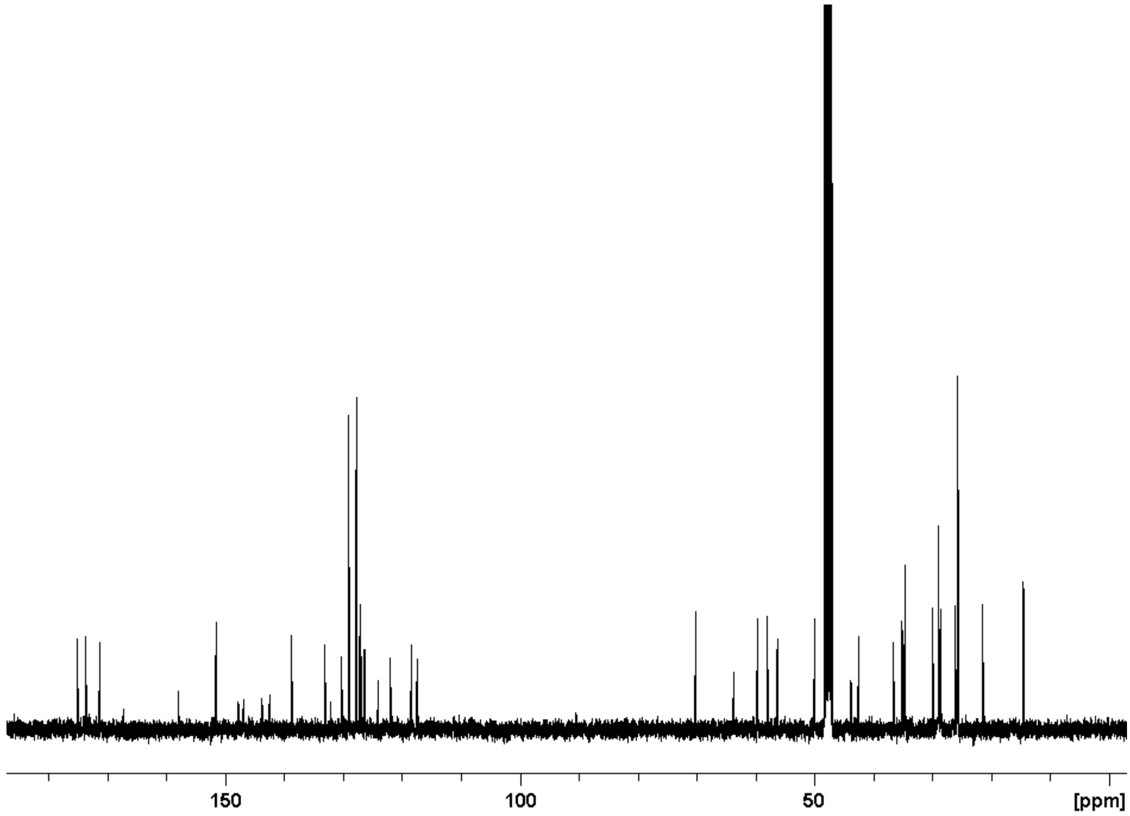
150
100
50
[ppm]
